# Cerebellocerebral connectivity predicts body mass index: a new open-source Python-based framework for connectome-based predictive modeling

**DOI:** 10.1093/gigascience/giaf010

**Published:** 2025-03-12

**Authors:** Tobias Bachmann, Karsten Mueller, Simon N A Kusnezow, Matthias L Schroeter, Paolo Piaggi, Christopher M Weise

**Affiliations:** Department of Neurology, University of Leipzig Medical Center, Leipzig 04103, Germany; Max Planck Institute for Human Cognitive and Brain Sciences, Leipzig 04103, Germany; Department of Neurology, First Faculty of Medicine and General University Hospital in Prague, Prague 12108, Czech Republic; Department of Neurology, University of Halle Medical Center, Halle 06102, Germany; Max Planck Institute for Human Cognitive and Brain Sciences, Leipzig 04103, Germany; Department of Information Engineering, University of Pisa, Pisa 56122, Italy; Department of Neurology, University of Halle Medical Center, Halle 06102, Germany

**Keywords:** connectome-based predictive modeling, functional magnetic resonance imaging (fMRI), Python, Human Connectome Project (HCP), cerebellum, BMI

## Abstract

**Background:**

The cerebellum is one of the major central nervous structures consistently altered in obesity. Its role in higher cognitive function, parts of which are affected by obesity, is mediated through projections to and from the cerebral cortex. We therefore investigated the relationship between body mass index (BMI) and cerebellocerebral connectivity.

**Methods:**

We utilized the Human Connectome Project’s Young Adults dataset, including functional magnetic resonance imaging (fMRI) and behavioral data, to perform connectome-based predictive modeling (CPM) restricted to cerebellocerebral connectivity of resting-state fMRI and task-based fMRI. We developed a Python-based open-source framework to perform CPM, a data-driven technique with built-in cross-validation to establish brain−behavior relationships. Significance was assessed with permutation analysis.

**Results:**

We found that (i) cerebellocerebral connectivity predicted BMI, (ii) task-general cerebellocerebral connectivity predicted BMI more reliably than resting-state fMRI and individual task-based fMRI separately, (iii) predictive networks derived this way overlapped with established functional brain networks (namely, frontoparietal networks, the somatomotor network, the salience network, and the default mode network), and (iv) we found there was an inverse overlap between networks predictive of BMI and networks predictive of cognitive measures adversely affected by overweight/obesity.

**Conclusions:**

Our results suggest obesity-specific alterations in cerebellocerebral connectivity, specifically with regard to task execution. With brain areas and brain networks relevant to task performance implicated, these alterations seem to reflect a neurobiological substrate for task performance adversely affected by obesity.

Key Points:

- Cerebellocerebral connectivity predicts body mass index (BMI).
- Task-general cerebellocerebral connectivity most reliably predicts BMI.
- Predictive networks derived this way overlap with established functional brain networks.
- There is an inverse overlap between networks predictive of BMI and networks predictive of measures adversely affected by overweight/obesity (i.e., positive predictive networks overlapped with negative predictive networks and vice versa).

## Background

The prevalence of overweight has increased substantially over the past decades. Globally, around 2 billion people can be classified as individuals with overweight or obesity as defined by a body mass index (BMI) of ≥25 or ≥30 kg/m^2^, respectively [1, 2]. Being not only numerous but a major risk factor of noncommunicable disease, a considerable amount of research on these conditions exists. Etiological considerations try to conceptualize overweight and obesity as a result of a behavior not adapted to fairly new obesogenic environments [3]. From a neuroscientific perspective, behavior is a manifestation of brain activity, and it therefore stands to reason that obesity is not only associated with (i) specific behavioral characteristics but also (ii) specific neuroanatomical characteristics.

Research on behavior associated with overweight, especially in the field of neuroimaging, has prominently been focusing on impulse control and reward processing [4–7]. Studies have also consistently demonstrated impairments in executive function in individuals with overweight and, more pronounced, individuals with obesity [8–10].

Following these concepts, most research on the neurobiological substrate of overweight has been concerned with cerebral cortical and subcortical regions thought to be involved in impulse control and affective regulation. Less light has been shed on the contribution of the cerebellum, even though it has consistently been demonstrated as functionally altered in individuals with overweight or obesity compared to individuals of nonpathological weight. In fact, recent meta-analyses count the cerebellum among 3 structures most robustly associated with obesity-related measurements [11, 12].

The cerebellum is an organized collection of a vast number of neurons commonly thought to be involved primarily in voluntary motor control. This model of cerebellar function dates back to the 19th century, when observations in animals and humans suffering from structural deficits (i.e., lesions or dys-/agenesis) of the cerebellum led to the formulation of a cerebellar syndrome with a set of core (motor) features, which is still very much in clinical use today: a combination of ataxia (dysmetria of the extremities and disturbance of balance and gait), dysarthria, and oculomotor abnormalities (most notably nystagmus). (For a comprehensive overview of the history of the study of the cerebellum, see [13].) In the late 20th century and more emphatically in the 21st century, it has been, on the grounds of consolidating evidence, argued that the cerebellum does, in fact, play a role in a number of nonmotor functions (i.e., higher-cortical functions like cognition, executive function, and language but also emotion, affect, and behavior) [14–20]. Of clinical importance, the corresponding symptoms of disturbances of nonmotor cerebellar function constitute a complementary cerebellar syndrome: cerebellar cognitive affective syndrome (CCAS).

Like the cerebellum’s motor functions, these nonmotor functions are thought to be put into effect through modulation of information of cerebral origin (for a comprehensive discussion, see the Discussion section). This mechanism is reflected in the cerebellum’s involvement in cerebral networks and in the existence of cerebellocerebral networks [22–25] as its neurobiological basis. Functional data suggest that most functional cerebellar units are indeed connected to nonmotor areas of the cerebral cortex (i.e., association areas [26]), while on a regional level, domain-specific activation of cerebellar regions justifies extending the functional topography of the cerebellum to nonmotor domains (for a comprehensive overview, see [27–29]).

Therefore, we aimed to investigate BMI-dependent cerebellocerebral networks with a focus on their role in behavioral function. To do so, we applied connectome-based predictive modeling (CPM; for the principal study, see [30]), a protocol for establishing relationships between brain functional connectivity and neuroimaging-independent measures (e.g., anthropometric or behavioral measures), which offers several advantages in comparison to more common approaches (e.g., seed-based methods). Constitutive advantages of CPM are (i) being data-driven and (ii) applying cross-validation. Regarding (i), no assumptions other than restricting connections as informed by our hypothesis (predictability of BMI by cerebellocerebral networks) were imposed on the data. By separating training and test datasets, cross-validation mitigates the problem of overfitting data: the performance of the model is evaluated on how well it performs on unseen data (for details, see the Methods section).

Taking into account the capabilities of CPM, our basic proposition of overweight- and obesity-specific behavioral alterations being mediated by cerebellar dysfunction can then be qualified. Thus, we hypothesized that cerebellocerebral connectivity, determined by resting-state and task-based functional magnetic resonance imaging (rsfMRI and tfMRI, respectively), is predictive of BMI in the context of CPM. We further hypothesized that tfMRI is more predictive of BMI than rsfMRI since executive function and therefore task performance seem to be more tangibly affected by overweight/obesity. As executive function is a general prerequisite for task execution, we speculated that tfMRI can task-independently be used to predict BMI. Finally, we evaluate if there is an overlap of predictive networks for BMI, as determined by CPM, with established functional brain networks and/or with predictive networks for cognitive and behavioral measures altered in overweight/obesity.

## Data Description

Regarding imaging data, for both rsfMRI and tfMRI (see the Methods section for details) we used the Human Connectome Project’s (HCP’s) 1200 Subjects preprocessed release. The HCP developed what became known as the HCP-style approach to neuroimaging data, an acquisition and processing protocol based on a set of principles (“tenets”) guided by new insights gleaned from technical and analytical progress [31].

Raw high-resolution imaging data were acquired using a customized MRI scanner with anatomically and physiologically informed parameters, which for fMRI at 3T (the field strength we used) translates to a relatively fine-grained 2-mm isotropic spatial resolution. Multiband pulse sequences were used with a multiband factor of 8 and a short repetition time (TR) of 0.72 seconds. Echo time (TE) equaled 33 ms, and the flip angle (FA) was calculated at $52^{\circ }$ to match the Ernst angle. Seventy-two slices per brain were acquired with left–right and right–left phase encoding directions and an asymmetric acquisition matrix to help with distortion-related losses. Also noteworthy is the long overall acquisition time (e.g., approximately 1 hour of combined rsfMRI data for each subject).

To preserve as much signal as possible and make use of the high-quality raw data in downstream analyses, only minimal preprocessing was applied, notably restraining from unnecessary spatial smoothing and temporal filtering. While a comprehensive and detailed description has been published elsewhere [32], we provide a concise description of the steps involved. In a first step, correction for distortions related to gradient nonlinearity (which is more pronounced in the HCP’s scanner setup) was applied with a FreeSurfer software package. The FSL software’s FLIRT method was then used to correct for head motion. Grand-mean intensity normalization was performed on the fMRI time series.

One of the keystones of fMRI studies is reliable intersubject comparability, which requires translating a subject’s physical space into a common standard space (“registering”). The HCP addressed this fundamental issue via multimodal registering—that is, using a variety of imaging modalities to reliably and automatically identify anatomical or functional landmarks in each subject’s 3-dimensional (3D) data and align them accordingly in what they call grayordinate space, a derivate of Montreal Neurological Institute (MNI) space, in which only matter of interest (i.e., gray matter) is preserved. Building on work described in [33], Glasser et al. [34] developed a multimodal and mapped areal feature–based (dubbed “MSMAll”) registration method, which uses myelin maps, resting-state brain networks, visuotopic maps, and a subcortical region of interest for intersubject alignment (see the supplemental methods of [34] for implementation details; for a discussion of the merits of multimodal registration in the context of high-resolution imaging data and why reliable registration is paramount to neuroimaging studies, see [35]).

Using the specially developed CIFTI file format [32, 36], the cortex is represented as a 2-dimensional (2D) surface and subcortical structures as 3D volumes, reflecting the inherent spatial properties of the dualist nature of human gray matter. This has profound practical consequences. First, unfolding the cortical surface improves spatial localization by avoiding bleeding of signal into geographically close but functionally distinct regions of neighboring sulci. Second, surface-based methods, again, aid intersubject comparability by abstracting from intersubject variability of cortical folding patterns. For our own analysis, individual subjects’ CIFTI files registered as discussed above (i.e., via “MSMAll”) constituted our starting point.

## Methods

**Figure 1:**
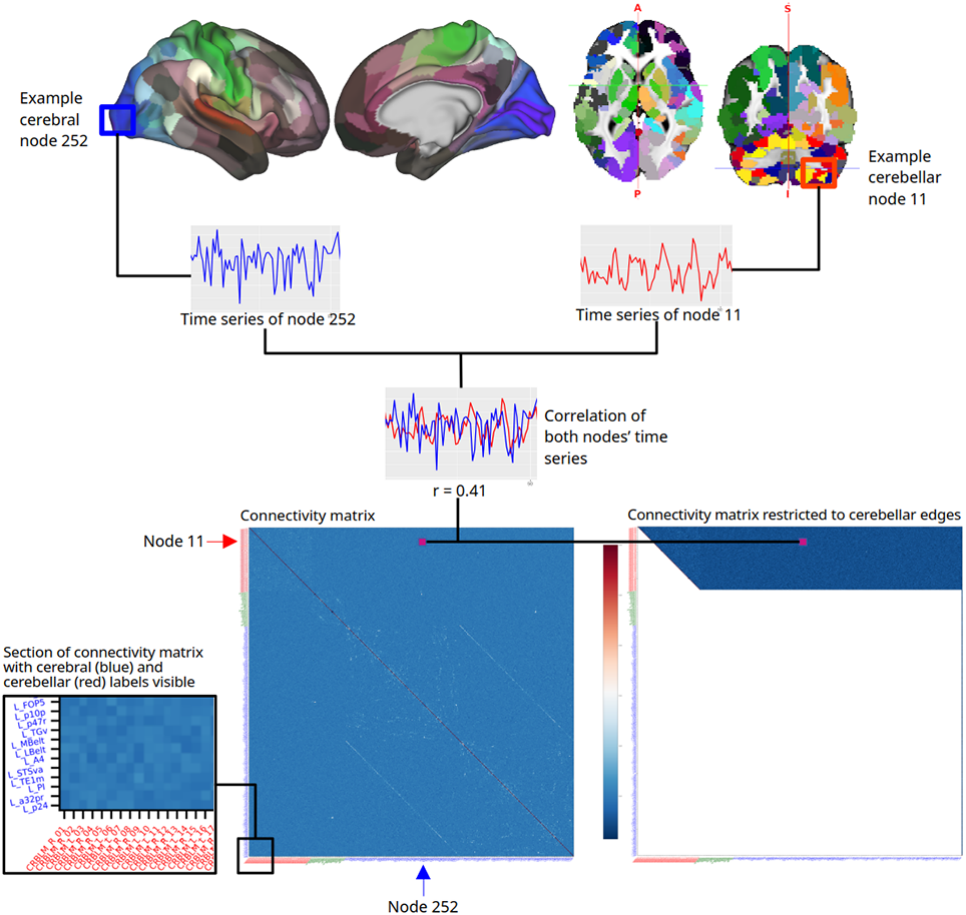
Flowchart depicting parcellated correlation analysis. The top row consists of our combined cerebrocortical–subcortical–cerebellar parcellation. A short time-series graph of exemplary nodes 11 and 247 is shown, the correlation of which, numerically represented by a value denoted with the letter r, makes up part of a connectivity matrix. The bottom-left panel shows an example connectivity matrix of a single subject. Cerebellar parcels are marked red, subcortical green, and cortical blue. The bottom-right panel shows the same connectivity matrix reduced to connections of interest.

We opted for parcellated analysis as opposed to voxel-based analysis. Parcels consist of a collection of geographically and ideally functionally related voxels. As such, parcels not only save (computing) time and (memory) space and improve intersubject comparability and statistical sensitivity [34, 35]. They also represent functional integration hubs upon which brain function is built [37]. Trying to be as data-driven as feasible and being interested in functional connectivity, our parcels are functionally informed demarcations within the HCP data set. By combining 3 separately published parcellations, we were able to create a detailed whole-brain functional parcellation model covering the cerebral cortex, subcortical structures, and the cerebellum. For the cerebral cortex, we used the HCP-MMP1.0 (Human Connectome Project Multi-Modal Parcellation version 1.0) by Glasser et al. [34]. They delineated 180 parcels per hemisphere (360 in total) by using the overlap of 4 areal feature maps, one for each modality (cortical thickness, relative myelin content, tfMRI, rsfMRI). Subcortical parcels were provided by Tian et al. [38]. They relied on subcortical-to-cortical connectivity derived from the HCP’s rsfMRI data to delineate 27 subcortical parcels per hemisphere (54 in total) along connectivity gradients (i.e., sufficiently stark changes in functional connectivity). Finally, cerebellar parcels came from an HCP-based study that clustered neighboring cerebellar voxels into 100 parcels by means of similarity of their rsfMRI time series [39]. Combining the aforementioned parcellations gave us 513 parcels in total (see Fig. 1). Our combined parcellation along with other auxiliary data and the entire code used in our study is publicly available (see below).

Using our parcels as nodes, we first extracted time series from the MSMall CIFTI files. For resting state, we used HCP Connectome Workbench software’s command-line application to average time series per parcel. Supplying the individual parcels as regions of interest, tfMRI was prepared with Python code based on the HCP pipelines script collection. With these time series, we calculated connectivity matrices for each subject using the Python package nilearn’s ([40]; nilearn is based heavily on scikit-learn [41]) ConnectivityMeasure class (see bottom-left panel of Fig. 1). For tfMRI, as opposed to more conventional partial correlation, we opted for tangent space–based connectivity matrices, which use a Riemannian manifold transformation [42, 43], as they were shown to be more sensitive to intersubject differences. Comparing different processing methods for CPM, Dadi et al. [44] found tangent-based parametrization and parcellations based on functional connectivity data to perform best. For a more general comparison, which also resulted in a recommendation for tangent space, see the work of Pervaiz et al. [45]. We were able to confirm these reports by producing better predictions following these recommendations. Nilearn’s implementation of Ledoit-Wolf’s [46] shrinkage estimator was used as a regularization technique.

As we are interested in cerebellocerebral connections only, we purged our matrices of connections of noninterest (see bottom-right panel of Fig. 1). Aside from limiting our connections of interest as informed by our hypothesis, no further anatomical assumptions were imposed on the data. These correlation-of-interest (COI) matrices (in the parlance of graph theory, which will be used later on, correlations represent “edges” between “nodes,” i.e., parcels) served as the basis for computing predictive networks in a CPM analysis.

### Connectome-based predictive modeling

**Figure 2:**
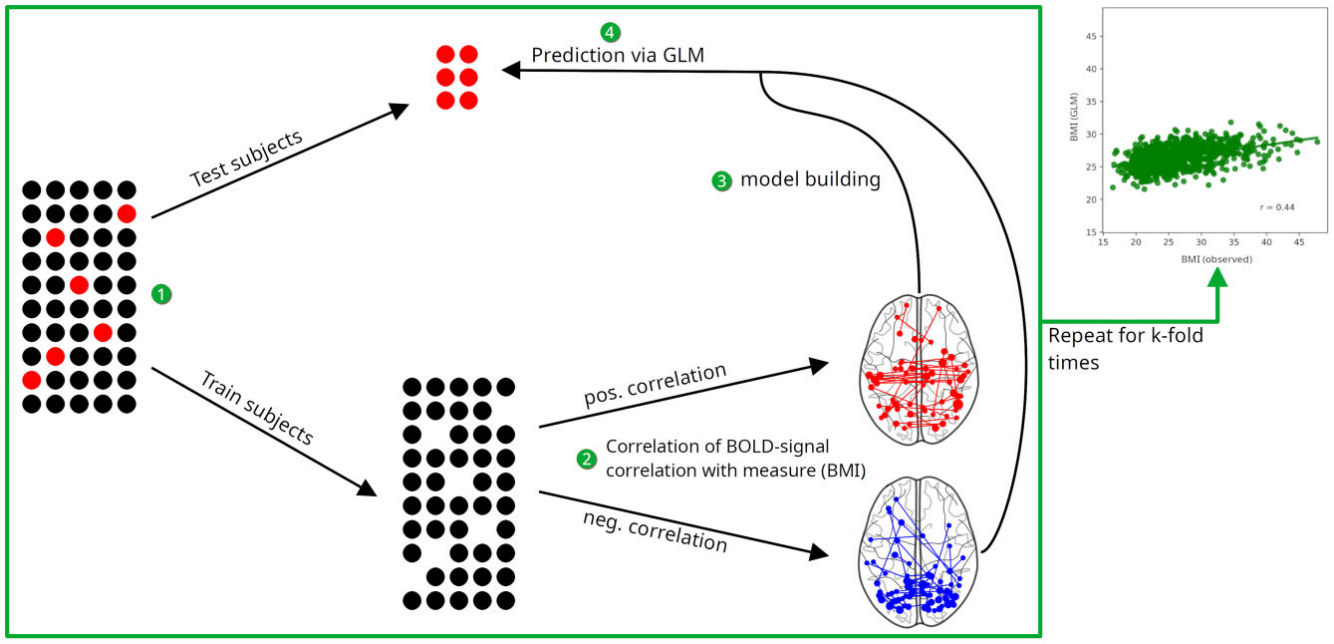
Flowchart depicting the steps involved in CPM. BOLD: blood-oxygen-level dependent; GLM: general linear model.

CPM consists of several steps (see Fig. 2): (i) subjects are randomly divided in a “train” and a “test” population, with their respective sizes being determined by the number of folds *k* (i.e., pairs of train and test populations). For illustration purposes: An extreme number of folds (*n* − 1) would correspond to the leave-one-out method, where, for each fold, a single subject constitutes the test population. We opted for *k* = 128, as a middle ground is reported to provide the most solid results [47], which is consistent with our own experience.

The train population’s connectivity matrices are then used to (ii) correlate their edges’ weight, that is, the strength of connections between nodes/parcels with the respective subject’s variable of interest (e.g., BMI). At this stage, nuisance variables (gender, age, ethnicity) were regressed out. This yielded 2 separate networks, one of positively correlated edges and one of negatively correlated edges. To improve signal-to-noise ratio, only those edges passing a *p* threshold of 0.05 were put through to the next stage, which is (iii) model building (note that *p* thresholding is just a means to select for “meaningful” edges and is not related to the statistical significance of our results, which is established later through permutation analysis). The HCP included a high number of twins (and subjects with other forms of close biological relation) in their study population. As both BMI [48, 49] and functional connectivity [50–54] are influenced by genetics, we took care to remove siblings from each fold before model building, thus ensuring that closely related subjects did not predict each other’s BMI.

The positive and negative networks of the train population were fitted into linear models describing the relation of brain area connections to the brain-external measure (BMI) within that population; combining the negative and positive network models, a general linear model (GLM) was built. Finally, these models, trained on the train population, were used to (iv) predict the test population’s BMI. This process was repeated for *k*-fold times, so that all subjects were test subjects once and the entire population’s BMI therefore predicted. Finally, Pearson’s *r* for population-level correlation of predicted with observed values was calculated.

### Task-based functional magnetic resonance imaging

Besides task-free (i.e., rsfMRI) data, the HCP offers tfMRI session data. The tasks performed by subjects tap into different domains of cognitive and affective function. For a summary of tasks and their rationale, see [55]; concise explanations of the tasks are provided in the following paragraphs.

The emotion task contained 2 conditions: fear and neutral. Subjects were presented pictures with fearful or angry faces (“fear” condition) or shapes (“neutral” condition) at the bottom of a screen and had to decide whether faces or shapes (respectively) at the top of the screen matched [55, 56].

The gambling task was designed to tap into incentive processing. Participants had to guess if the value of a card was less or more than 5 and would win 1 US dollar if correct and lose one if wrong [55, 57]. Accordingly, the gambling task was split into 2 conditions: loss and win.

The language task consisted of the “story” condition testing semantic understanding and, for comparison, of the “math” condition, where participants had to solve arithmetic tasks [55, 58].

For the relational processing task, participants had to decide whether pairs of objects differed along the same dimensions (i.e., shape or texture) or if an object matched other objects with regard to a specified dimension; these different subtasks amounted to the “relational” and “match” condition, respectively [55, 59].

During the social cognition task, based on Frith–Happé animations designed to test participants’ theory of mind, geometrical objects were displayed interacting (“mental” condition) or moving randomly (“random” condition), and participants had to decide whether movement of those objects represented social interaction [55, 60, 61].

The working memory task is a variant of N-back tasks. Subjects were presented with pictures of faces, places, body parts, and tools in the 0-back and 2-back fashion [55]. As we were more interested in working memory as an executive function subdomain than in the localizing function of different picture categories [62], we combined all 0-back runs and all 2-back runs into 2 respective conditions.

To address our hypothesis of task-independent predictability of BMI, we proceeded to (i) average connectivity matrices of task conditions, resulting in a single connectivity matrix per task (averaging was done per subject; i.e., the connectivity matrix serving as input for CPM for subject X was calculated by averaging the connectivity matrices, e.g., the “loss” and “win” condition of the gambling task of that subject), and (ii) finally combine and average all tasks (i.e., adding all task-specific connectivity matrices per subject and dividing the resulting cumulative matrices by the number of tasks).

### Statistical significance

To assess statistical significance of our results (i.e., the correlation of BMI as predicted by our GLM with observed BMI) while avoiding the pitfalls of parametric testing [63], we used permutation analysis, which consisted of repeatedly performing CPM after permuting BMI within the test population. The number of permutations was chosen as to establish significance at *P*  $\le$ 0.001 (*P* equals the proportion of permutations equal to or greater than our true prediction) after correcting for multiple comparisons (we took a conservative approach with 10,000 permutations for the task-based analysis and 2,000 permutations for the resting state–based analysis, which satisfies conservative correction methods like Bonferroni’s). For a graphical representation of the results, see Supplementary Figure S13. Permutation analysis is a powerful, albeit computationally expensive, method. As a means to deal with computational load, our analysis code is able to make use of parallel and distributed (as in multimachine) computing, to which end we rely on the Ray framework [64] in its Python-based incarnation.

### Overlap with networks predictive of related measures

To further explore our theory of a predictive network–determining relationship of cerebellar nonmotor function and BMI’s negative association with the latter, we ventured to analyze the overlap between networks predictive of BMI, on one hand, and networks predictive of other measures, on the other hand. We first calculated Pearson’s *r* for BMI and measures of interest, that is, measures of executive function (Wisconsin Card Sorting Test and Eriksen flanker task), general cognition (Penn Matrix Reasoning Test), and reward-related self-regulation (delay discounting; for a description of these measures, their acquisition, and usage in the context of the HCP, see [55, 65]).

For exploratory purposes, we then performed CPM (with averaged tfMRI, as it yielded the most promising results in our primary analysis) on measures of interest, that is, measures of executive function (Wisconsin Card Sorting Test and Eriksen flanker task), general cognition (Penn Matrix Reasoning Test), and reward-related self-regulation (delay discounting; for a description of these measures, their acquisition, and usage in the context of the HCP, see [55, 65]). We finally compared the resulting predictive networks with those of our primary analysis by multiplying masks of connectivity matrices of significant edges, thus creating a connectivity matrix describing an overlap network. Ranking of nodes was based on their weighted degrees averaged over overlapping networks (see Supplementary Section S2 for details).

## Results

### Population

Characteristics of our study population are summarized in Table 1. We only included subjects for whom all needed data were available, which included BMI for all subjects and respective neuroimaging data for each of the fMRI modalities. Therefore, the number of subjects differed between fMRI modalities and ranged from 999 (resting-state fMRI) to 1,077 (gambling task). The number of subjects with complete data for all tasks, which could thus be included in our combined task analysis (see below), was 999. Demographic variables were very similar between groups; they all contained more female than male participants and were predominantly white (∼75%). Based on BMI, more than half of our subjects were individuals with overweight or obesity, a quarter were in the normal weight range, and a small minority (∼1.5%) were underweight. Median BMI was in the lower overweight range.

**Table 1:** Table of summary population statistics

		Task-based fMRI					
Characteristic	rsfMRI, *n* = 999	Emotion, *n* = 1,041	Gambling, *n* = 1,077	Language, *n* = 1,007	Relational, *n* = 1,034	Social, *n* = 1,042	Working memory, *n* = 1,074
Age	29 (26, 32)	29 (26, 32)	29 (26, 32)	29 (26, 32)	29 (26, 32)	29 (26, 32)	29 (26, 32)
Gender							
Female	532 (53%)	558 (54%)	581 (54%)	540 (54%)	553 (53%)	559 (54%)	582 (54%)
Male	467 (47%)	483 (46%)	496 (46%)	467 (46%)	481 (47%)	483 (46%)	492 (46%)
Ethnicity							
Indigenous	2 (0.2%)	2 (0.2%)	2 (0.2%)	2 (0.2%)	2 (0.2%)	2 (0.2%)	2 (0.2%)
Asian/Pacific	63 (6.3%)	64 (6.1%)	64 (5.9%)	61 (6.1%)	64 (6.2%)	64 (6.1%)	64 (6.0%)
Black	139 (14%)	145 (14%)	155 (14%)	141 (14%)	144 (14%)	145 (14%)	154 (14%)
More than 1	24 (2.4%)	24 (2.3%)	29 (2.7%)	23 (2.3%)	23 (2.2%)	23 (2.2%)	29 (2.7%)
Unknown	17 (1.7%)	16 (1.5%)	18 (1.7%)	13 (1.3%)	16 (1.5%)	16 (1.5%)	18 (1.7%)
White	754 (75%)	790 (76%)	809 (75%)	767 (76%)	785 (76%)	792 (76%)	807 (75%)
BMI	25.4 (22.8, 29.1)	25.4 (22.8, 29.1)	25.5 (22.8, 29.2)	25.4 (22.8, 29.2)	25.4 (22.8, 29.2)	25.4 (22.8, 29.1)	25.5 (22.9, 29.2)
Weight group							
Normal weight	426 (43%)	447 (43%)	454 (42%)	434 (43%)	442 (43%)	448 (43%)	450 (42%)
Obesity	233 (23%)	241 (23%)	255 (24%)	230 (23%)	242 (23%)	240 (23%)	256 (24%)
Overweight	326 (33%)	337 (32%)	351 (33%)	327 (32%)	334 (32%)	338 (32%)	351 (33%)
Underweight	14 (1.4%)	16 (1.5%)	17 (1.6%)	16 (1.6%)	16 (1.5%)	16 (1.5%)	17 (1.6%)

Values are presented as median (interquartile range) or *n* (%).

### Resting-state functional connectivity of parcels

**Figure 3:**
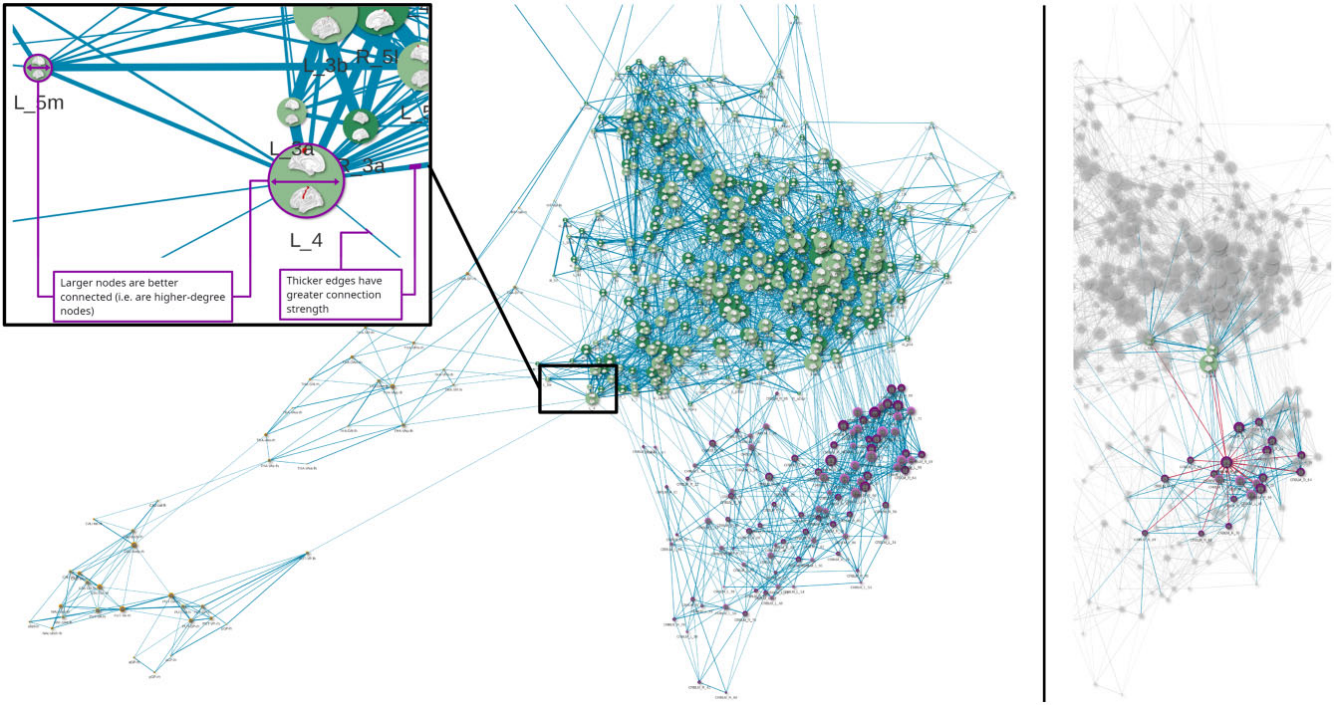
Screenshots of an interactive web browser–based visualization of resting-state functional MRI connectivity between all parcels; cerebral parcels are colored green, cerebellar parcels violet, and subcortical parcels orange (brighter and darker hues denote left and right hemispheres, respectively). The right panel shows primary (red) and secondary (blue) connections of 1 selected cerebellar parcel.

Taking rsfMRI time series from our whole-brain parcellation as a starting point and using visNetwork [66], an R [67] package, we are able to provide an interactive overview of functional connectivity between parcels (see Fig. 3 for illustration; the interactive webpage is available in GigaDB [see Data Availability section]—note that no anatomical information was supplied to the algorithm seemingly “grouping” cerebral, cerebellar, and subcortical parcels; the spatial proximity of the respective group’s parcels to each other is solely a function of their greater interconnectedness).

### CPM

Our primary measure was Pearson’s *r* describing the relationship between observed (i.e., recorded by the HCP) and predicted (by our GLM) BMI. While this correlation is the most direct measure of predictive success, it is not informative with regard to the neurobiological substrate of brain–behavior relationships. Of neuroscientific interest are specific predictive networks and the nodes and edges they consist of. (i) For visual inspection, we plotted networks on a schematic brain (see Results figures) and provide interactive web-based plots for our main results (available in GigaDB, see Data Availability section). (ii) We plotted the most powerfully predictive edges one by one. (iii) To reach a better understanding of cerebral regions involved, we sorted cerebral nodes according to their relevance in the respective networks; our concept of relevance here makes use of the graph-theoretical notion of a weighted degree (i.e., the sum of the weights of all edges a node is connected by). We finally compared these weightiest nodes to established brain networks.

The following presentation of our results is of a descriptive nature; we explore functional implications in the Discussion section.

#### rsfMRI-based CPM

**Figure 4:**
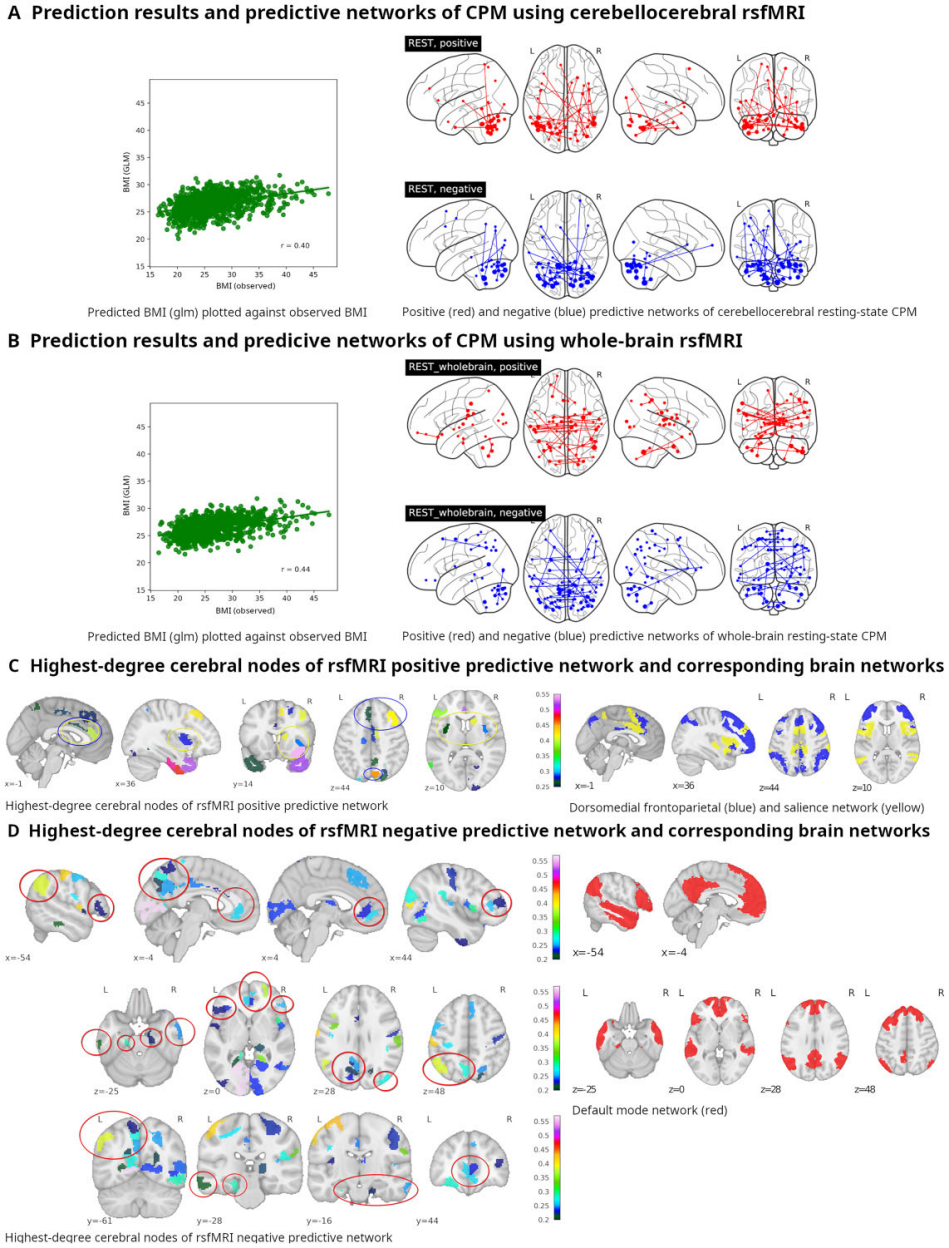
(A, B) Left panel: Plot of observed body mass index vs. predicted body mass index (*p* for Pearson’s $r \le 0.001$). Right panel: Plots of positive and negative predictive networks onto a glass brain. (C) Left panel: Slices showing cerebral nodes of the resting-state *positive* predictive network with nodes participating in the dorsomedial frontoparietal network (dmFPN) and salience network (SN) encircled in blue and yellow, respectively. Right panel: Plots of established brain networks associated with our resting-state *positive* predictive network; slices at strategic locations show networks 6 and 4 of Yeo et al.'s [21] 7-network parcellation (corresponding to the dmFPN, blue, and the SN, yellow). Coordinates are MNI coordinates. Colors and numbers on colorbars indicate weighted degrees. (D) Left panel: Slices showing cerebral nodes of our resting-state *negative* predictive network. Coordinates are MNI coordinates. Colors and numbers on colorbars indicate weighted degrees. Right panel: Slices plotting network 7 of Yeo et al.’s 7-network parcellation ^a^, which corresponds to the default mode network associated with our resting-state *negative* predictive network. L: left; R: right.^a^For the established brain networks, data were downloaded from https://surfer.nmr.mgh.harvard.edu/fswiki/CorticalParcellation_Yeo2011 and plotted with nilearn (filename was Yeo2011_17Networks_MNI152_FreeSurferConformed1mm_LiberalMask.nii.gz for the 17 network parcellation and Yeo2011_7Networks_MNI152_FreeSurferConformed1mm_LiberalMask.nii.gz for the 7 network parcellation, both contained in Yeo_JNeurophysiol11_MNI152.zip.)

Performing CPM with rsfMRI data yielded a modest correlation of observed with predicted BMI (*r* = 0.44; results were statistically significant, *P* ≤ 0.001; see Supplementary Fig. S13). Intracerebellar connections dominated both negative and positive networks, the former more than the latter. The positive predictive network’s highest-degree nodes notably included bitemporal nodes (temporal poles and ventromedial areas of the temporal poles), the right insular cortex and the left cingulate cortex, and biprefrontal and biparietal areas (see Fig. 4). The left V1 reached the top spot in the negative predictive networks’ list of best connected nodes, which also featured as the only subcortical region in a thalamic subdivision.

As an interesting side note, whole-brain rsfMRI CPM, as opposed to rsfMRI CPM restricted to cerebellocerebral and cerebellocerebellar connections, performed only slightly better in predicting subjects’ BMI (*r* = 0.44 vs. 0.40; see A vs. B in Fig. 4).

#### tfMRI-based CPM

tfMRI sessions consisted of several conditions per task group (e.g., 0-back and 2-back conditions for the working memory task). Designed by the HCP for this purpose, we tried to utilize these constellations of conditions by subtracting connectivity matrices of the more-general condition (e.g., 0-back) from more-specific conditions (e.g., 2-back) to allow for capturing task-specific activations as opposed to overarching task-general activations. Besides 2-back vs. 0-back conditions with working memory, we analyzed story vs. math conditions in language, relational vs. match conditions in the relational task, and the theory-of-mind condition vs. random conditions in the social task. Interestingly, predicted-observed correlations with task contrasts were generally low and not significant.

**Figure 5:**
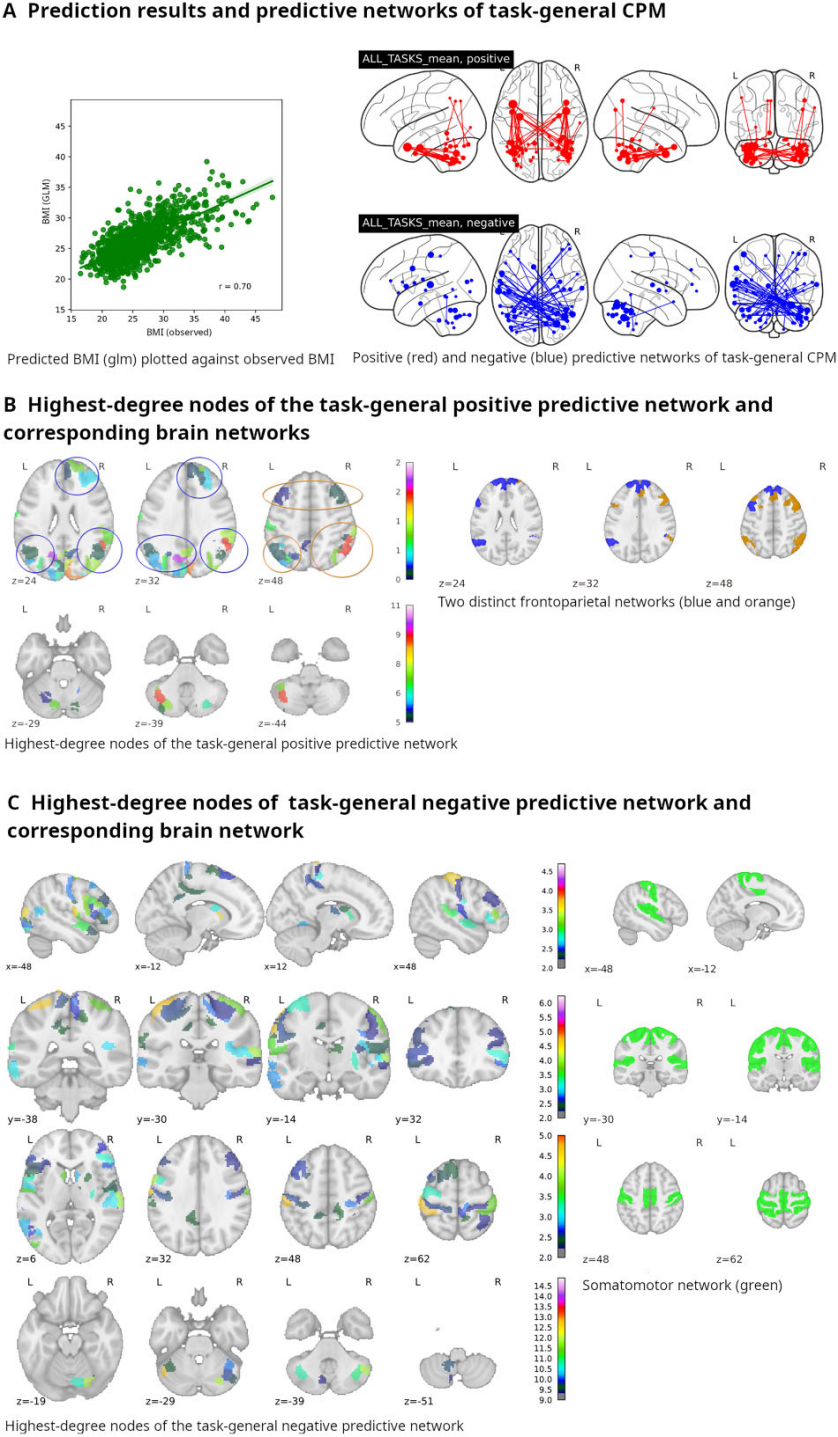
(A) Left panel: Plot of observed body mass index vs. predicted body mass index (*p* for Pearson’s *r*  $\le 0.001$). Right panel: Plots of positive and negative predictive networks onto a glass brain. (B) Left panel: Slices showing cerebral (top panel) and cerebellar (bottom panel) nodes of the task-general *positive* predictive network involved, among other things, in 2 established frontoparietal networks (encircled in blue and orange, respectively). Coordinates are MNI coordinates. Colors and numbers on colorbars indicate weighted degrees. Note that preferential distribution in the right cerebral hemisphere corresponds to a preferential distribution in the left cerebellar hemisphere. Right panel: Plots of aforementioned established frontoparietal networks associated with our task-general *positive* predictive network; slices at strategic locations show networks 13 and 17 of Yeo et al.’s [21] 17-network parcellation$^{\rm a}$. (C) Left panel: Slices showing cerebral (top 3 panels) and cerebellar nodes (bottom panel) of the task-general *negative* predictive network. Coordinates are MNI coordinates. Colors and numbers on colorbars indicate weighted degrees. Right panel: Slices plotting the established somatomotor network (SMN) associated with our task-general *negative* predictive network at strategic locations, plotted with data from Yeo et al.’s (2011) 7-network parcellation, where the SMN corresponds to network 2$^{\rm a}$. L: left; R: right.

While specific predictive networks for individual tasks could be identified, correlation was more pronounced the more general the task data were prepared. Task groups (i.e., averaged task conditions) yielded markedly better results than individual tasks and task contrasts, while task-general activation (i.e., all task conditions averaged) outperformed both in predicting BMI (*r* = 0.70, *P* ≤ 0.001; see Fig. 5). A rather detailed description of individual task results and their respective plots can be found in Supplementary Section S1. In the following section, we will concentrate on (i.e., plot, list, and discuss) the most relevant 5% (by weighted degree) of cerebral nodes of the task-general predictive networks; the number of cerebellar nodes to concentrate on was decided by visual determination of a degree threshold via inspection of their degree distribution (see the bar plots in Supplementary Fig. S7).

The *positive* predictive network was clearly dominated by ipsilateral and contralateral symmetric cerebellar projections to just a few temporopolar nodes, namely (adopting the names provided by Glasser et al. [34]) (i) dorsal and (ii) ventral area TG (see also [68]) and (iii) perirhinal cortex (see Table 2 for further details). Cerebellar nodes involved in the positive predictive network were also symmetrically located in the lateral posterior hemispheres, where they tended to be more ventrally located; only 1 intercerebellar edge was among the top 100 (ordered by edge strength), but a number of intracerebellar edges within the respective lateral posterior cerebellar hemispheres were (also symmetrical). The 5 most predictive cerebellocerebral edges connected left cerebellar node 86, its contralateral counterpart node 49, and adjacent node 4 to contralateral temporal nodes. Interestingly, these cerebellar nodes were all located in crus VIIb (see Discussion section).

**Table 2:** List of top 30 (by weighted degree) cerebral nodes in the positive predictive network for all tasks combined (averaged). Area names denote either Brodmann areas (numerical) or follow von Economo and Kosinas’s letter system (for a concise overview, see [68]). Cerebral divisions for cortical parcels by Glasser et al.’s [34] parcellation are based on its Neuroanatomical Supplementary Results as compiled by [69].

Node	Degree	Label	Name	Cerebral division	MNI coordinates
325	4.99839	R_TGv	Right Area TG Ventral	Lateral Temporal	39.81 $-$0.81 $-$42.01
455	4.9941	L_PeEc	Left Perirhinal Ectorhinal Cortex	Medial Temporal	$-$ 29.14 $-$9.22 $-$32.93
464	4.81841	L_TGd	Left Area TG Dorsal	Lateral Temporal	$-$ 39.32 9.55 $-$31.94
275	4.31887	R_PeEc	Right Perirhinal Ectorhinal Cortex	Medial Temporal	29.4 $-$7.66 $-$33.54
505	3.90656	L_TGv	Left Area TG Ventral	Lateral Temporal	$-$ 41.2 −2.21 $-$41.38
284	3.00586	R_TGd	Right Area TG Dorsal	Lateral Temporal	39.95 11.8 $-$31.73
304	2.63506	R_PGs	Right Area PGs	Inferior Parietal	45.35 −64.99 37.45
168	2.59669	R_POS2	Right Parieto-Occipital Sulcus Area 2	Posterior Cingulate	12.41 $-$68.35 38.55
348	2.32532	L_POS2	Left Parieto-Occipital Sulcus Area 2	Posterior Cingulate	$-$ 9.23 $-$69.57 36.83
302	2.17232	R_PFm	Right Area PFm Complex	Inferior Parietal	51.34 $-$47.96 40.5
158	2.06219	R_V3	Right Third Visual Area	Early Visual	20.69 −86.43 8.49
465	2.05809	L_TE1a	Left Area TE1 Anterior	Lateral Temporal	−58.78 $-$9.83 $-$20.57
451	2.00351	L_EC	Left Entorhinal Cortex	Medial Temporal	−21.06 $-$14.52 $-$28.18
466	1.89598	L_TE1p	Left Area TE1 Posterior	Lateral Temporal	−58.29 $-$46.77 $-$9.99
496	1.77366	L_VVC	Left Ventral Visual Complex	Ventral Stream Visual	$-$ 30.93 $-$52.31 $-$16.89
157	1.75576	R_V2	Right Second Visual Area	Early Visual	13.04 −78.5 5.59
225	1.72395	R_10d	Right Area 10d	Orbital and Polar Frontal	10.29 64.82 6.67
363	1.71058	L_7m	Left Area 7m	Posterior Cingulate	$-$ 4.57 −61.87 36.73
301	1.69895	R_PF	Right Area PF Complex	Inferior Parietal	58.54 $-$31.42 35.7
271	1.69591	R_EC	Right Entorhinal Cortex	Medial Temporal	21.61 $-$14.02 $-$27.89
296	1.69274	R_PGp	Right Area PGp	Inferior Parietal	42.77 −75.44 23.8
299	1.6117	R_IP0	Right Area Intraparietal 0	Inferior Parietal	34.4 $-$72.07 30.14
183	1.58747	R_7m	Right Area 7m	Posterior Cingulate	5.57 −61.75 36.66
298	1.56293	R_IP1	Right Area Intraparietal 1	Inferior Parietal	35.85 $-$62.69 42.61
508	1.55809	L_A4	Left Auditory 4 Complex	Auditory Association	−60.62 $-$24.47 7.55
240	1.54291	R_9a	Right Area 9 Anterior	Dorsolateral Prefrontal	17.26 59.18 21.32
288	1.52408	R_TF	Right Area TF	Medial Temporal	42.23 $-$21.44 −27.25
178	1.51844	R_PSL	Right Perisylvian Language Area	Temporo-Parieto-Occipital Junction	60.55 $-$37.3 24.57
478	1.46096	L_IP1	Left Area Intraparietal 1	Inferior Parietal	−30.49 $-$65.54 42.52
287	1.44772	R_TE2a	Right Area TE2 Anterior	Lateral Temporal	55.13 $-$18.49 $-$27.3

MNI: Montreal Neurological Institute.

The *negative* predictive network, on the other hand, proved to be less concentrated and dominated by just a few nodes. Contralateral edges prevailed (not a single ipsilateral edge passed the threshold), with the right cerebellum contributing weightier (by weighted degree) nodes. Interestingly, the edge with the most predictive power connected a left cerebellar node to a subdivision of the right putamen. Other than that, the weightiest edges connected right cerebellar nodes with temporal nodes (but excluding the poles and including bilateral auditory area 4), followed by parietal nodes and intercerebellar connections, which were far more numerous than in the positive predictive network (see Table 3 for further details). It is worth noting that the cerebellar constituents of the most predictive edges were again located in crus VII (left hemisphere: node 99, right hemisphere: nodes 20, 24, 70, and 96).

**Table 3:** List of top 30 (by weighted degree) cerebral nodes in the negative predictive network for all tasks combined (averaged). Area names denote either Brodmann areas (numerical) or follow von Economo and Kosinas’s letter system (for a concise overview, see [68]).

Node	Degree	Label	Name	Cerebral division	MNI coordinates
508	6.24397	L_A4	Left Auditory 4 Complex	Auditory Association	−60.62 $-$24.47 7.55
384	4.66822	L_1	Left Area 1	Somatosensory and Motor	$-$ 44.99 −25.26 52.28
389	4.09919	L_6v	Left Ventral Area 6	Premotor	$-$ 55.79 1.93 31.74
328	4.00675	R_A4	Right Auditory 4 Complex	Auditory Association	63.44 $-$20.25 7.24
204	3.65353	R_1	Right Area 1	Somatosensory and Motor	45.89 −22.03 52.67
143	3.61732	CAU-VA-lh	Left Ventroanterior Caudate	Caudate Nucleus	$-$ 8.49 11.35 4.96
492	3.58627	L_LO3	Left Area Lateral Occipital 3	MT+ Complex and Neighboring Visual Areas	$-$ 42.01 $-$79.06 11.72
433	3.57763	L_OP4	Left Area OP4/PV	Posterior Opercular	$-$ 55.9 −13.38 15.43
361	3.32351	L_STV	Left Superior Temporal Visual Area	Temporo-Parieto-Occipital Junction	$-$ 57.46 $-$47.33 17.28
387	3.19566	L_6d	Left Dorsal Area 6	Premotor	$-$ 32.02 $-$13.25 62.94
411	3.19286	L_6r	Left Rostral Area 6	Premotor	$-$ 50.68 7.0 18.08
114	3.17489	PUT-VP-rh	Right Ventroposterior Putamen	Putamen	30.73 $-$10.67 $-$0.64
235	3.14016	R_IFSa	Right Area IFSa	Inferior Frontal	45.2 38.3 8.08
414	3.10309	L_IFSp	Left Area IFSp	Inferior Frontal	$-$ 43.96 22.44 21.72
277	3.05388	R_PBelt	Right ParaBelt Complex	Early Auditory	58.2 $-$19.32 8.4
466	3.04505	L_TE1p	Left Area TE1 Posterior	Lateral Temporal	−58.29 $-$46.77 $-$9.99
432	3.00455	L_43	Left Area 43	Posterior Opercular	$-$ 56.12 −1.03 9.93
255	2.97411	R_OP2-3	Right Area OP2-3/VS	Posterior Opercular	38.28 $-$16.01 18.61
257	2.96739	R_RI	Right Retroinsular Cortex	Early Auditory	43.04 $-$29.43 17.69
440	2.96613	L_TA2	Left Area TA2	Auditory Association	$-$ 51.57 0.9 $-$4.87
412	2.96198	L_IFJa	Left Area IFJa	Inferior Frontal	$-$ 42.34 13.25 25.61
327	2.95945	R_LBelt	Right Lateral Belt Complex	Early Auditory	50.27 $-$24.46 10.32
177	2.89554	R_A1	Right Primary Auditory Cortex	Early Auditory	44.82 $-$21.49 9.69
335	2.88416	L_MST	Left Medial Superior Temporal Area	MT+ Complex and Neighboring Visual Areas	$-$ 43.71 $-$68.77 7.23
116	2.87957	CAU-VA-rh	Right Ventroanterior Caudate	Caudate Nucleus	9.87 11.89 5.04
260	2.84924	R_TA2	Right Area TA2	Auditory Association	51.39 1.0 $-$5.42
470	2.83976	L_PHT	Left Area PHT	Lateral Temporal	$-$ 55.16 −57.57 0.6
458	2.81946	L_A5	Left Auditory 5 Complex	Auditory Association	−59.57 $-$17.43 $-$0.75
465	2.79938	L_TE1a	Left Area TE1 Anterior	Lateral Temporal	−58.78 $-$9.83 $-$20.57
355	2.77208	L_PIT	Left Posterior Inferotemporal Complex	Ventral Stream Visual	$-$ 38.25 $-$81.59 $-$11.24

IFJ: inferior frontal junction; IFS: inferior frontal sulcus; MNI: Montreal Neurological Institute.

Statistical significance was assessed with permutation testing; for a graphical representation, see Supplementary Fig. S13.

### Overlap analysis

**Figure 6:**
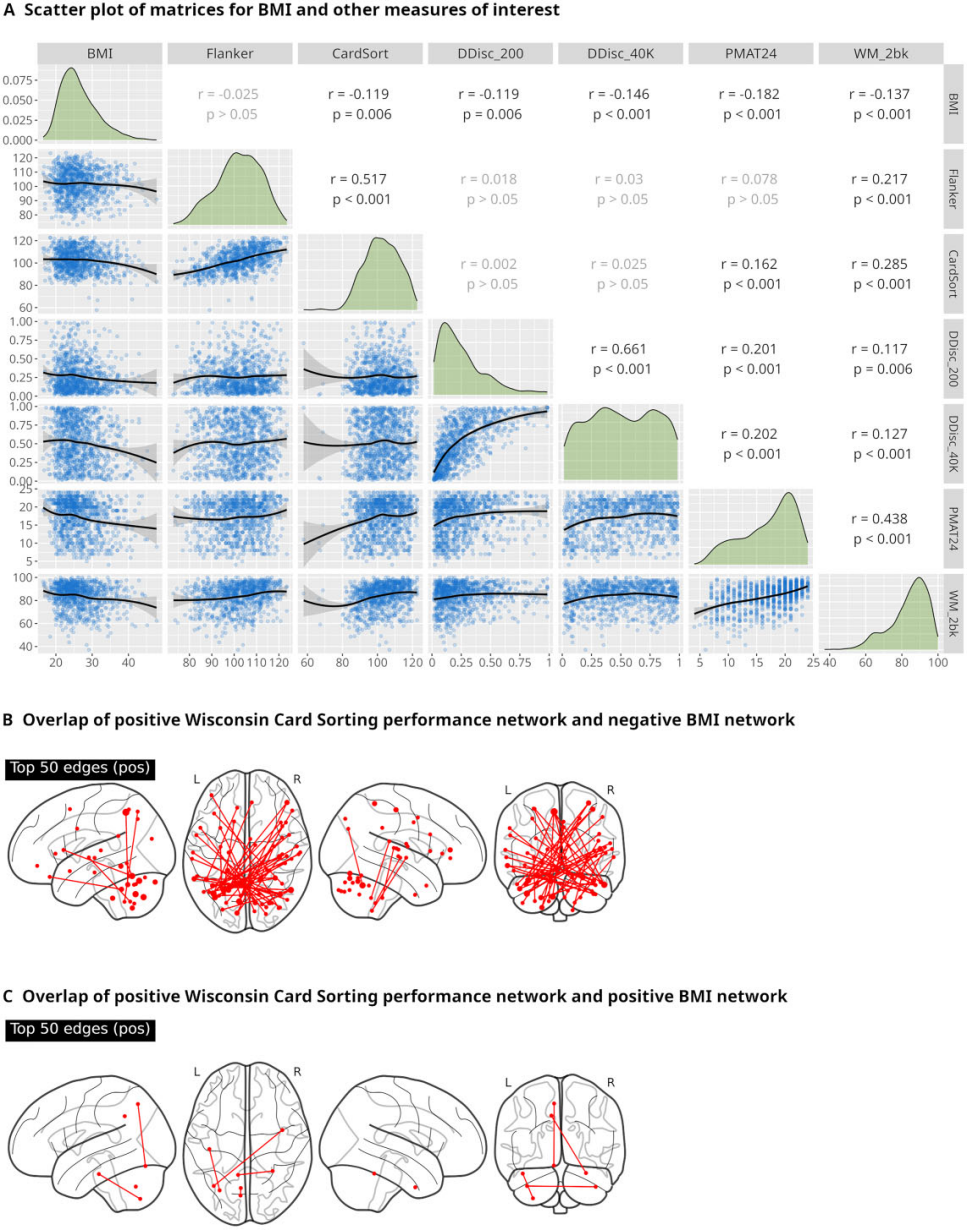
(A) Scatterplot of matrices visualizing Pearson correlation for BMI and other measures of interest. Diagonally, a density plot of the measures is shown; the lower triangle depicts scatter plots of the respective measures, while the upper triangle denotes Pearson’s *r* and Bonferroni-corrected *P* values as determined by permutation testing (the code used to replace ggplot2’s [79] built-in parametric test function along with an example call used to generate image A of this figure is part of hcp-suite and can be found in our repository (see below) in hcp-suite/utils/add_perm_P.R.) as none of the measures showed a normal distribution in our sample (as seen in the density plots and verified with Shapiro–Wilk normality testing). CardSort: age-adjusted results for Wisconsin Card Sorting Test; DDisc_200/40K: area under the curve for delay discounting (200 $ and 40000 $ conditions, respectively); Flanker: age-adjusted results for flanker task; PMAT24: correct responses in the Penn Matrix Reasoning Test; WM_2bk: accuracy in the working memory 2-back task. (B) Example overlap between the network *positively* predicting Wisconsin card sorting performance and the network *negatively* predicting BMI. (C) Example overlap (or lack thereof) between the network *positively* predicting Wisconsin card sorting performance and the network *positively* predicting BMI.

As expected, BMI was negatively associated with measures of executive and cognitive function, with the Penn Matrix Reasoning Test showing the strongest negative association and the flanker task the least (the latter is also the only measure where statistical significance was not reached; see Fig. 6 for details).

Crucially, a significant overlap between networks predictive for BMI and other measures of interest was only observed when comparing positive predictive networks for BMI with negative predictive networks of the other measures (and vice versa; see Fig. 6 for an example). This is in line with our theory, which predicts this kind of inverse relationship. An alternative explanation would be that this observation is simply a consequence of the inverse relationship of BMI with the other measures. However, correlations between BMI and the other measures are weak and significant only due to the large number of subjects, though. We find it therefore more plausible to attribute the inverse overlap to the predictive networks being functionally distinct. Consequently, positive predictive networks for BMI overlapping with negative predictive networks of the other measures had high-ranking frontoparietal nodes reminiscent of frontoparietal executive networks, and negative predictive networks for BMI overlapping with positive predictive networks of the other measures tended to have high-degree somatomotor cortex nodes.

## Discussion

Using CPM, we aimed to investigate whether and to which extent cerebellocerebral connectivity predicts BMI. Both tfMRI and rsfMRI yielded networks positively and negatively predictive of BMI. In the following sections, we will provide an in-depth discussion and interpretation of our results (i.e., of important nodes within the networks and of the networks as networks). We will start with the rsfMRI-based positive and negative predictive networks, followed by the tfMRI-based positive and negative predictive networks.

### Resting-state CPM

CPM with cerebellocerebral connections based on rsfMRI data as opposed to tfMRI yielded markedly worse predictions of BMI. It is interesting to note, though, that whole-brain CPM with rsfMRI data performed not much better (Pearson’s *r* = 0.40 vs. 0.44, respectively), which confirms the cerebellum’s prominence with respect to obesity-related brain changes noted in literature.

We will first discuss cerebellar nodes, then move on to discussing cerebral nodes of the positive and negative predictive network separately, where we will again focus on established brain networks that were recognizable in our results.

**Figure 7:**
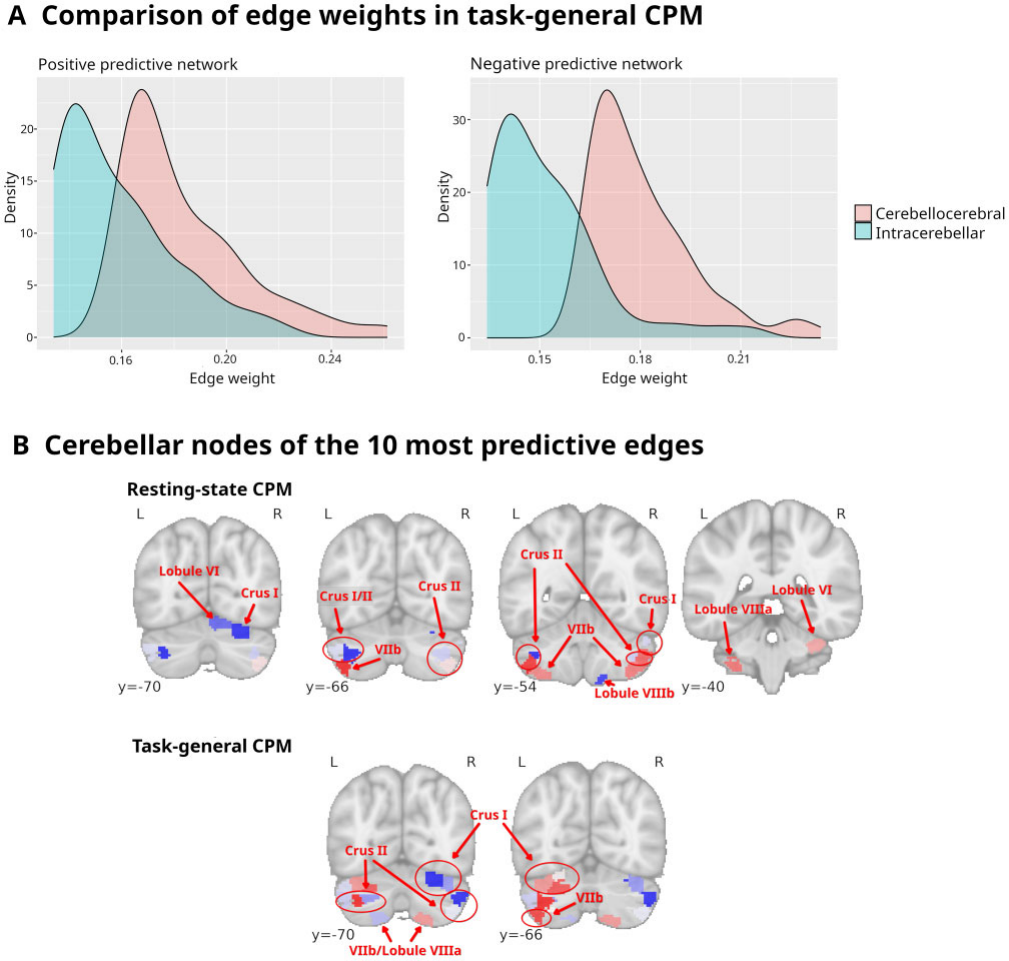
(A) Comparison of weights of cerebellocerebral and intracerebellar edges of the positive (left) and negative (right) predictive network for task-general connectome-based predictive modeling. (B) Cerebellar nodes of the 10 most predictive edges of connectome-based predictive modeling of task-based functional magnetic resonance imaging (fMRI) (top) or resting-state fMRI (bottom). Nodes of positive predictive networks are colored in red and nodes of negative predictive networks in blue. Locations of nodes with regard to cerebellar lobules are annotated. Coordinates are MNI coordinates.

While in the case of tfMRI, cerebellocerebral edges made up most predictive edges in the positive and negative predictive networks, with only a few intercerebellar connections making the list, this ratio was reversed for rsfMRI. On the other hand, cerebellar nodes in the rsfMRI predictive networks were geographically constrained to a much higher degree, with most nodes being directly adjacent to each other (see Fig. 7B).

#### Resting-state positive predictive network

The rsfMRI positive predictive network’s best-connected cerebral nodes contained the dorsal-anterior portion of the right insula and the left anterior cingulate cortex (see Fig. 4), corresponding to key nodes of the salience network (for an overview, see [70]). Also included was the left caudate nucleus, which belongs to subcortical areas associated with the salience network. Interestingly, evidence in psychiatric disease implicates the caudate nucleus and its contribution to the salience network (SN) in (impaired) cognitive flexibility and inhibitory control [71, 72], which, along with functional connectivity of the caudate nucleus, tends to be also impaired in individuals with obesity [73, 74].

Apart from the SN, the rsfMRI positive predictive network’s cerebral nodes are similar to the ones for tfMRI, which are most conspicuous for the prominence of the temporal poles and ventromedial temporal lobes. Less pronounced but nonetheless interesting is another executive network partially overlapping with the frontoparietal networks (FPNs; aka “central executive network”) discussed above. Distinct from that network is the emergence of the anterior to middle cingulate cortex and the medial superior parietal cortex as hubs (the network we refer to here corresponds most closely to the green cluster [75] labeled the dorsomedial frontoparietal network [dM-FPN]). Being part of the executive network family, this network is also implicated in executive functions (EFs).

#### Resting-state negative predictive network

Within the cerebral faction of the rsfMRI negative predictive network, we found a pattern of nodes reflecting virtually all key clusters of the default mode network (for an overview of the default mode network, see [76]): bilateral orbital frontal cortices, medial prefrontal and adjacent anterior cingulate cortices, lateral temporal cortices, inferior parietal cortices, posterior cingulate, and parahippocampal cortices (see Fig. 4). Most famous for being active in rest and downregulated when a task is performed [77], the default mode network has been implicated in various conditions. Of relevance to the individual-level neuroscientific view on the obesity complex, a major study on the topic of impulsivity has shown the default mode network’s co-correlation with motor planning areas to be associated with impulsivity in juvenile offenders and developing youth [78].

Moving away from networks, one of the notable nodes being well connected in the rsfMRI negative predictive network is the auditory cortex. We will address the auditory cortex as a multimodal integration hub in the section discussing the tfMRI negative predictive network.

### Task-based CPM

After averaging connectivity matrices for different task conditions, CPM yielded generally good results, differing only marginally in their range from *r* = 0.59 (emotion task) to *r* = 0.61 (relational task) to *r* = 0.62 (language, social, and working memory task), see Supplementary Section S1. Improving prediction by averaging instead of just appending condition time series (thus keeping the number of data points the same while leveling out task-specific correlations) suggests that there are indeed task-general networks at play.

The discussion of the results of task-based CPM will therefore focus on averaged task data (“task general”). We will first discuss general aspects of our results, highlighting specific cerebellar nodes, whose connections were found to be most predictive of BMI, and continue with a per-network discussion with an emphasis on cerebral nodes and their participation in cerebral networks.

While we discovered distinct networks in each single-task CPM, when considering the analysis of combined tasks, a pattern was vindicated, which had been indicated in the individual task analyses: the negative predictive network was considerably stronger overall (with regard to edge strength and weighted degree), with its cerebellocerebral and cerebellocerebellar connections overwhelmingly connecting contralateral nodes. In contrast, the positive predictive network had more and stronger ipsilateral connections. In the positive predictive network, the distribution of nodes across cerebellar hemispheres was numerically balanced with one hemisphere mirroring the other; judging by weighted degree, though, left cerebellar nodes ruled superior. Cerebellar nodes in the negative predictive network, on the other hand, were predominantly (number-wise and degree-wise) found in the right hemisphere, which is also reflected in their comparative weighted degrees. With regard to the anatomical distribution of cerebral nodes involved in our predictive networks, temporal poles and entorhinal and perirhinal cortices featured prominently in the positive predictive network, while they were conspicuously absent in the negative network (see Table 3 and Table 2). Other regions present in both networks notably included the lateral and medial prefrontal cortices as well as superior parietal areas. Interestingly, cerebellocerebral edges tended to rank higher than intracerebellar edges among those with the highest predictive power (see Fig. 7), supporting the notion of cerebellar function being mediated through its involvement in cerebral networks.

Suprathreshold cerebellar nodes in both networks were almost exclusively located in the posterior hemispheres. This can be interpreted in light of the cerebellum’s functional division. A coarse division based on functional associations can delineate a motor cerebellum (anterior hemispheres), a limbic cerebellum (vermis and adjacent regions, the paravermis), and a cognitive cerebellum (posterior hemispheres). The posterior cerebellum’s dominant role in cognitive processes was confirmed in lesion studies [80], and comparative anatomy lends support to the notion of the posterior hemispheres contributing to higher cognition, as they are the phylogenetically youngest part and seem to have expanded alongside cerebral regions relevant to cognitive functions associated with modern humans [81–84].

In fact, most nodes of the 10 most predictive edges were located in lobule VII and all of them in lobules VI, VII, and VIII (see Fig. 7). Gross-anatomically, the cerebellum can be divided into 2 lobes (anterior and posterior) and 10 lobules, I to X. While lobules I to IV correspond to the anterior lobe, lobules VI to IX are part of the posterior lobe; lobule VII is subdivided into crus I, crus II, and lobule VIIb. In line with the general tendency of our results, lobule VII specifically (and, to a lesser degree, lateral lobule VI) is considered the principal lobule of the cognitive-affective cerebellum, with regions of lobule VIII also implicated [29, 85, 86]. Note that the only node from the positive predictive network not located in lobule VII is located in lobule VIIIa, which is adjacent to lobule VII; this might reflect a meaningful discrepancy between structural-anatomical and functional divisions. The bulk of lobule VIII, on the other hand, forms part of the motor cerebellum.

This notion of functional specialization of cerebellar regions is borne out by recent conceptualizations. Multiple lines of neuroimaging evidence converge on multiple representations of nonmotor functions (task-negative [i.e., DMN-related] and task-positive [i.e., EF-related and attention-related]). Within this division, lobule VI and crus I form the first nonmotor representation, and crus II forms the second nonmotor representation [22, 87]. As mentioned, crus I and crus II are part of lobule VII, and most of our most relevant nodes are found in these cerebellar regions. It is worth noting that functional divisions do not conform to structural divisions in lobes and lobules, which has been confirmed by another recent major study by King et al. [85]. It is not surprising, then, that the nodes found to be most relevant in our study do not strictly adhere to structural boundaries like lobules and crurae.

In a recent meta-review concerned with the cerebellum’s role in appetite control, crus I and lobule VI were highlighted as being consistently altered, a finding the authors explained by pointing out these cerebellar regions’ association with executive and emotional control [88]. There is direct evidence for BMI-associated activation of crus I and lobule VI in response to food cues, with the increased activation of these cerebellar areas being reversible after leptin replacement–associated BMI reduction in leptin-deficient individuals [89]. Both the latter study and the aforementioned metareview by Sader et al. [88] recognized an asymmetry in crus I and lobule VI activation favoring the left cerebellum, which can also be seen in our results (for the positive predictive network, as expected; see Fig. 7B).

Lobule VI and crus I are also implicated in reward processing, which in the context of our analysis resonates with the view of obesity as an addiction-like state with pathological food craving [90]. For instance, greater functional connectivity of bilateral lobule VI with the ventral tegmental area, an important reward-processing center, in obese women in response to energy-dense food cues was reported [91]. In a study on regular cocaine users, cocaine and food cues led to a similar pattern of activation predominantly in lobule VI and crus I [92]. Reward-related direct cerebellar projections to the ventral tegmental area seem to be of a behaviorally highly relevant modulating nature [93], possibly on the basis of intracerebellar model generation and prediction error correction [94].

In addition, experimental evidence suggests a role of lobule VI and crus I in prediction error handling of emotional stimuli [95]. Overeating and obesity are intricately linked to negative affective states [96, 97]. Drawing conclusions from these associations is not straightforward since negative social bias toward obesity may contribute to affective disorders. Yet, cerebellar processing of predicted emotional consequences of food intake may contribute to overeating [98].

As described above, we noted a per-network lateralization of cerebellar nodes, with the positive predictive network being characterized by weightier left cerebellar nodes and the negative predictive network by weightier right cerebellar nodes. Several functional neuroimaging and lesion studies speak to a lateralization of higher cognitive cerebellar function. Broadly, the right cerebellum is associated with language and more general cognition, although this could well be secondary to its function in language. Of interest to our study, D’Mello et al. [99] found concrete executive control to be lateralized to the right cerebellum. The left cerebellum’s role is less elucidated (it also plays only a minor role in transcranial stimulation of the cerebellum; see [100]), but a meta-analysis of functional neuroimaging data reveals a strong association with executive function [29], while others found it to be involved in attention and visuospatial processing (for an overview, see [101]). It is unclear at this point how our results fit into this picture.

#### Task-general positive predictive network

We set out to investigate cerebellocerebral connectivity on the grounds of advanced theories of the cerebellum's function, insofar it contributes to higher cognitive function, within cerebral networks. Accordingly, we were able to identify established neural networks within our positive and negative predictive task-general networks. Habas et al. [24] analyzed cerebellar contributions to cerebral networks, including the central executive network. The central executive or frontoparietal network (for a discussion of nomenclature, see [75, 102]) consists of intercommunicating brain areas located primarily in the bilateral frontal and parietal lobes, where they form a mirror image of each other. This signature pattern of nodes reverberates in our positive predictive network’s cerebral components, albeit with preference of the right hemisphere; this is reflected in the cerebellar nodes’ left-hemispheric preference. Other than that, the cerebellar areas participating in the frontoparietal network found by Habas et al. could be replicated in our positive predictive network (see Fig. 5B).

The presence of frontoparietal networks (FPNs) in our positive predictive network offers an explanation of the task-general model’s superior predictive capabilities in comparison to single-task models. Averaging the latter’s connectivity matrices accentuates common predictive patterns (e.g., a meta-task network such as the FPN). Once termed the central executive or central control network, the FPNs are a set of networks for which no universally agreed-on terminology exists (for a valuable effort to resolve some of the confusion surrounding frontoparietal networks involved in executive function, see [75]). Yet, considerable agreement exists toward their function. They are attributed a key role in executing so-called executive functions (EF). The term EF denotes a set of cognitive functions underlying virtually all conscious behavior (for an overview, see [103]. One of the core components of executive function is working memory (the others being inhibitory control and cognitive flexibility; building on those are planning, reasoning, and problem-solving as higher-order executive functions). While the working memory task is therefore a direct measure of executive function performance, all tasks rely on higher cognitive function and thus on EF; their relation to overweight and the cerebellum therefore warrants a broader discussion. The bilateral entorhinal and pararhinal cortices contribute prominently to our positive predictive network; both areas are implicated in working memory and, likely therefore, executive functions’ performance (the latter might be secondary to the former [104–106]; for an integrative theory of working memory and executive function implying the perirhinal cortex, see [107]).

Several studies were able to demonstrate lower EF in overweight individuals (for reviews, see [8, 9, 108, 109]. Experimental evidence suggests impairments specifically in EF (as, e.g., working memory deficits can be elicited even though explicit learning is unaffected by obesity) [110]. Models of understanding the relationship between EF impairment and obesity point to the importance of EF in dietary intake control as they mediate the kind of goal-orientated behavior that is necessarily involved with delayed gratification [111, 112].

Lesion and neuroimaging studies, on the other hand, robustly show the cerebellum’s involvement in EF. The original lesion study leading to formulation of the CCAS [113] described EF deficits as one of its hallmarks (next to deficits in visuospatial cognition, language, and affection—for an updated description, see [114]).

Even better connected than areas implicated in the frontoparietal networks were nodes symmetrically located at the bilateral temporal poles, with ventral and apical temporopolar nodes being the best connected of all noncerebellar nodes. The temporal pole seems to be an integration center for a range of modalities (for an overview, see [115]), with the apex in particular functioning as a hub interconnecting other temporopolar functional divisions. The temporopolar apex mainly consists of area TG, being one of the best connected nodes of the positive predictive network, and is noted by Pascual et al. [116] for its strong functional connectivity to the posterior cerebellum. In addition to its role as a temporopolar hub, area TG’s functional connectivity suggests, alongside ventrolateral area TE’s, a role in semantic processing (note that left area TE1 is also among the highest-degree nodes of the positive predictive network). Integrative, amodal semantic processing is indeed thought to be central to temporopolar function [117], with a stimulation study elegantly confirming the semantic-notional (as opposed to verbal-linguistic) nature of this specific temporopolar function [118]. For neurodegenerative diseases like Alzheimer’s and frontotemporal dementia, temporopolar hypometabolism has been linked to deficits in executive function [119, 120]. Given this evidence, it does not seem too great a leap to assume the temporal poles’ meta-task relevance.

As a fourth component pattern, with the orbital frontal cortex, medial prefrontal cortex, and adjacent anterior cingulate cortex, as well as lateral temporal corices, inferior parietal lobes, posterior cingulate/retrosplenial cortex, and hippocampus/parahippocampal cortices, all key hubs of the default mode network (DMN) were present. The DMN is discussed in more detail in the context of the rsfMRI’s negative predictive network.

#### Task-general negative predictive network

While the positive network comprised cerebral nodes associated with the FPN and the temporal poles as supramodal hubs, the negative predictive network’s most relevant nodes (see Fig. 5C) are found in the bilateral primary sensory and motor cortices as well as auditory cortices. In terms of established cerebral networks, the pattern of (cerebral) nodes in the negative predictive network resembled most closely the sensorimotor, or pericentral, network (SMN), which centers on primary sensor and motor cortices. The SMN is primarily involved with integration of sensory inputs and coordination of motor outputs. As such, it is connected to numerous other networks and regions, including, unsurprisingly, the cerebellum. Not unlike the cerebellum, the SMN has recently been implicated in nonmotor functions, including executive function [121, 122] and task-independent temporal processing, which has been termed sensorimotor synchronization [123, 124].

In sensorimotor synchronization, the auditory system is thought to play a crucial role [125]. The relationship between SMN and auditory cortices is indeed close to a point where the latter have been considered part of the former [26, 102]. This relationship, like the SMN’s great interconnectedness in general, can be conceptualized within the SMN’s prominent role in internal modeling and model updating (for a review of evidence and theoretical constructs for the auditory cortices’ contribution to internal modeling, see [126]).

## Summary and Conclusions

Before providing a high-level summary of our study and exploring possible explanations of its findings, we think it helpful to offer 4 key points that can be distilled from our CPM results:

1. Cerebellocerebral connectivity predicts BMI.
2. Task-general cerebellocerebral connectivity most reliably predicts BMI.
3. Predictive networks derived this way overlap with established functional brain networks.
4. There is an inverse overlap between networks predictive of BMI and networks predictive of measures adversely affected by overweight/obesity (i.e., positive predictive networks overlapped with negative predictive networks and vice versa).

Applying connectome-based predictive modeling to functional MRI parcellated along functionally informed lines, we built general linear models to predict BMI. Each general linear model was based on 2 separate networks consisting of edges correlating positively or, respectively, negatively with BMI. In comparison, models built with task-based functional MRI predicted BMI with substantially higher accuracy than those built with resting-state functional MRI. Nevertheless, the respective predictive networks of both modalities shared common features.

Generally speaking, both positive and negative predictive networks featured multimodal integration hubs (temporal poles and ventromedial temporal lobes for the positive and auditory cortices for the negative predictive networks). Consistent with evidence positioning cerebellar functional connectivity within cerebral connectivity, we were able to identify established neural networks within positive and negative predictive networks. For the positive predictive networks, these were frontoparietal networks involved in EF, the default mode (tfMRI only) and the salience network (rsfMRI only). Within our negative predictive networks, we found the sensorimotor (both tfMRI and rsfMRI) and default mode network (rsfMRI only).

On the basis of its homogeneous cellular architecture, it has been proposed that the cerebellum is performing the same operation on the input it receives independent of the nature of the function it is involved in (e.g., basic motor or higher cognitive function), aptly called “universal cerebellar transform” (see, e.g., [127]). Cerebellar nodes can thus be thought of as processing hubs modulating cerebral function. Consistent with our results, the posterior cerebellum, specifically lobule VII, has been previously highlighted as the location of such hubs [128].

Lobule VII was also highlighted in a study finding gray-matter density in the cerebellum to be negatively correlated with neuron-specific enolase plasma levels, a marker of neuronal injury [129]. The authors interpret that finding as evidence for cerebellar vulnerability to obesity-related neuronal injury. A possible mechanistic link between altered cerebellar properties and obesity is provided by leptin, a hormone heavily involved in obesity [130]. Leptin receptors are most densely expressed in the cerebellum [131], and induction or withdrawal of leptin replacement therapy in leptin-deficient individuals has been shown to increase [132] or decrease [133] cerebellar gray-matter volume, respectively. Leptin replacement therapy also reproducibly altered the functional response of the cerebellum to food cues [89, 134].

The nature of the cerebellum’s influence on cerebral nodes and networks and why this would allow us to predict BMI in subjects is beyond the scope of our study. Pointers can be gleaned from studies measuring cerebrobehavioral effects of modulating cerebellar-to-cerebral output. This can be achieved through noninvasive (i.e., transcranial) stimulation, where modulation of the cerebellar neurons’ electrical activity has an impact on neuronal activity in connected cerebral areas. The most studied target area of this phenomenon is the primary motor cortex, where excitatory stimulation of the cerebellum has a depressive effect, which has been termed cerebellar brain inhibition [135]. Taking this robust effect as a starting point, efforts have been made to study the impact of transcranial cerebellar modulation on nonmotor domains (for an overview, see [100, 136]). The effect of cerebellar output is less clear, however, with evidence suggesting both inhibitory and excitatory roles (and sometimes both; see [137]).

If we accept the premises of the cerebellum’s role in cognition, affective regulation, and task execution, as well as an inverse correlation of BMI and performance in these domains, and suppose that the cerebellum’s altered functional status in overweight and obesity is an expression of these premises, our data would suggest an overall inhibitory role of the cerebellum. Cerebellar connectivity to EF-related networks was positively correlated with BMI, leading, in the proposed framework, to an inhibitory effect on task performance, while the salience network being part of rsfMRI’s positive predictive network and the DMN being part of rsfMRI’s negative predictive network would lead to less efficient task switching. The occurrence of an executive network in the rsfMRI’s negative predictive network and of the DMN in the tfMRI’s positive predictive network can be held against this hypothesis, although these networks do happen to be involved in their respectively “wrong” state of activeness (i.e., the executive network during rest and the DMN during task performance). A noninhibitory role of the cerebellum in these cases would be an alternative interpretation.

Whatever the correct mechanistic interpretation, empirical evidence does indeed implicate the cerebellum in feeding and appetite control. With its numerous reciprocal connections to key structures for homeostasis (including energy homeostasis and its circadian fluctuations), affective control and reward processing (where involvement in hedonic aspects of food intake has been shown), as well as motor aspects of eating, the cerebellum contributes to virtually every aspect of food intake (for a recent review, see [98]).

Rather excitingly, in a rodent model of Prader–Willi syndrome, a syndrome characterized, among other things, by insatiable appetite, a drastic reduction in food intake could be achieved by selective activation of a cerebellar satiation network. Associated cerebellar regions had been identified in humans employing functional MRI while presenting food-related cues to subjects, as they were the only brain regions with significantly different activations in subjects affected by Prader–Willi syndrome compared to control subjects [138]. In humans, appetite can be modulated via transcranial cerebellar stimulation [139].

One reading of the notion of an obese brain holds that brains of individuals with obesity are *a priori* (i.e., prior to being conditioned by experience) predisposed to obesogenic behavior, leading to obesity when exposed to an obesogenic environment. However, there are most likely adaptive neural processes at play, creating and shaping obesity-related interneuronal connections, possibly reinforcing obesogenic behavior. Persuasive evidence of obese experiences shaping the obese brain can be found when studying individuals undergoing substantial weight loss (e.g., patients undergoing bariatric surgery). Central nervous system targets for such research usually involve the dopaminergic system (for a review including methodological limitations, see [140]). While a recent study was negative with regard to changes in dopaminergic response to food intake, its postsurgery follow-up was rather short term [141]. Van der Zwaal et al. [142], on the other hand, were able to show increased dopamine receptor availability after long-term weight loss in bariatric surgery patients. It would be interesting to see if and how changes in BMI would change predictive networks in subjects.

## Limitations

The HCP provided us with high-quality imaging data for a large number of subjects, but basing our analyses on preexisting data had drawbacks, as we were inherently limited by the kind of data provided. While resting-state fMRI is inherently goal-agnostic and therefore not affected by this aspect, when designing an obesity study from scratch, a different selection of tasks might have been more appropriate to answer our questions. Yet, this fact is mitigated 2-fold. First, the HCP carefully chose their tasks to cover as much ground as possible and thus included tasks suited for our purposes. Second, task-general brain activity turned out to be more predictive, rendering specific task content less important.

We used BMI as our principal target measure as the biometric measures it is based on are available for virtually all HCP subjects. While being the most commonly used measure for obesity, arguably because it is easily determined, other measures may perform better in capturing obesity as a condition worthy of medical consideration (e.g., they are better predictors for obesity-related disorders) [143–147]. This is a concern for our study, as the biological basis for its assumption of altered cerebellocerebral connections is the existence of that very obesity condition.

On a final note, it is important to keep in mind that, due to the correlative nature of our study, causal relationships cannot be established.

## Availability of Source Code and Requirements

To obtain our results, a number of methodological-technical hurdles had to be overcome. One of these had to do with the HCP’s development of new imaging protocols and file formats to store imaging data. Thus, with regard to our purposes, no established analysis pipelines existed. In addition, to complete our analysis in a reasonable time frame, computing power not available in a single machine was required, which led to extensive use of cluster computing. Our efforts were helped intensively by using open-source software, which allowed us to inspect existing software, build upon existing solutions, and adapt code freely according to our needs on every level (from high-level scripting to writing custom functions altering low-level backend code).

Our newly developed software framework for CPM with a focus on HCP data is available under the GNU General Public License v3 in a Git repository at https://codeberg.org/tobac/hcp-suite. The repository also includes a detailed tutorial on how to use our software. The method was registered with the DOME-ML registry [148]. A Jupyter notebook is provided in the associated GigaDB dataset to reproduce the results reported in this article.

- Project name: HCP Suite
- Project homepage: https://codeberg.org/tobac/hcp-suite
- bio.tools Identifier: biotools:hcp_suite
- WorkflowHub DOI: 10.48546/WORKFLOWHUB.WORKFLOW.1234.1
- RRID: RRID:SCR_026222
- Latest Git commit this project is based on: 2677bbba7e
- Operating system(s): Platform independent
- Programming language: Python $\ge$ 3.7, R $\ge$ 4.0, GNU bash $\ge$ 4.4
- Other requirements: See the README in the repository for installation instructions
- License: GNU GPL v3

The specific software versions used to generate the results presented in this article were Python 3.11.5 [149], R 4.4.1 [150], and GNU bash 5.2.15.

Notable Python packages include NetworkX 3.3 [151], nibabel 5.2.1 [152], nilearn 0.10.4 [40], Pandas 2.2.2 [153], Pingouin 0.5.5 [154], and Ray 2.35.0 [64].

Notable R packages include ggplot2 3.5.1 [79], gtsummary 1.7.2 [155], and visNetwork 2.1.2 [66].

## Abbreviations

BMI: body mass index; CCAS: cerebellar cognitive affective syndrome; CPM: connectome-based predictive modeling; dmFPN: dorsomedial frontoparietal network; DMN: default mode network; EF: executive function; FPN: frontoparietal network; GLM: general linear model; HCP: Human Connectome Project; IQR: interquartile range; MNI: Montreal Neurological Institute; MRI: magnetic resonance imaging; rsfMRI: resting-state functional magnetic resonance imaging; SMN: somatomotor network; tfMRI: task-based functional magnetic resonance imaging; WM: working memory.

## Competing Interests

The authors declare that they have no competing interests.

## Supplementary Material

giaf010_Supplemental_File

giaf010_GIGA-D-24-00385_Original_Submission

giaf010_GIGA-D-24-00385_Revision_1

giaf010_Response_to_Reviewer_Comments_Original_Submission

giaf010_Reviewer_1_Report_Original_Submission Bo-yong Park -- 10/14/2024

giaf010_Reviewer_1_Report_Revision_1 Bo-yong Park -- 1/6/2025

giaf010_Reviewer_2_Report_Original_Submission Sandra Chanraud -- 10/30/2024

## Data Availability

Imaging and nonimaging data are available via the HCP after registering. Specifically, the following resources provide starting points to access and handle the public dataset this study is based on (”Human Connectome Project–Young Adult Study”). All supporting data and materials are available in the *GigaScience* repository, GigaDB [156]. Public data website: https://www.humanconnectome.org/study/hcp-young-adult Register for access: https://db.humanconnectome.org/app/template/Login.vm (simple sign-on) Access to restricted data (includes link to e-access application form): https://www.humanconnectome.org/study/hcp-young-adult/document/restricted-data-usage Data: WU-Minn HCP 1200 Subjects Data Release, for which the following reference manual applies: https://www.humanconnectome.org/storage/app/media/documentation/s1200/HCP_S1200_Release_Reference_Manual.pdf
