# Supplementary material for: Cerebellocerebral connectivity predicts body mass index: a new open-source Python-based framework for connectome-based predictive modeling
Source: Gigascience. 2025 Mar 12;14:giaf010. doi: 10.1093/gigascience/giaf010 (PMC11899596; doi:10.1093/gigascience/giaf010)
Supplement: giaf010_Supplemental_File [file giaf010_supplemental_file.docx]

# S1: Detailed description of individual-task tfMRI results (averaged over conditions)

### Emotion task

The emotion task contained two conditions: fear and neutral. Subjects were presented either pictures with fearful or angry faces (“fear” condition) or shapes (“neutral” condition) at the bottom of a screen and had to decide whether faces or shapes (respectively) at the top of the screen matched (Barch et al. 2013; Hariri et al. 2002).

Predictive power was very similar between the fear condition (r = 0.51) and the neutral condition (r = 0.50). Positive predictive networks bore more resemblance between conditions than negative predictive networks with positive predicitve edges connecting cerebellar nodes to mostly ipsilateral cerebellar and temporal, parietal and occipital (visual areas) nodes. Other than nodes in the mentioned areas, best-connected noncerebellar nodes in negative predictive networks of the *fear* condition notably included two subcortical nodes: subdivisons of the right caudate nucles and left thalamus. Negative predictive cerebellar connections in the fear conditions projected to more frontally located nodes (contralateral) featured a pattern of more laterally orientated cerebellar nodes in the *fear* condition on one hand (relative to positive predictive nodes) and quite centrally located in the *neutral* condition on the other hand (see supplemental figures and tables for more details regarding these results).

These latter differences were less pronounced for *averaged* task conditions (see fig. S[1](#fig%252525253Aresults_emotion_mean)), which had, as with the other tasks, better predictive powers than the conditions themselves (r = 0.59). As with task conditions, positive predictive networks featured more ipsilaterally orientated edges and less cerebral frontal nodes, but, in contrast, more parietal nodes. Best-connected noncerebellar nodes within the postive predictive network included bilateral temporal poles and perirhinal cortices as well as visual areas, while the auditory cortex featured quite heavily in the list of top negative nodes, which again included subcortical nodes (left thalamus and caudate nucleus).

| 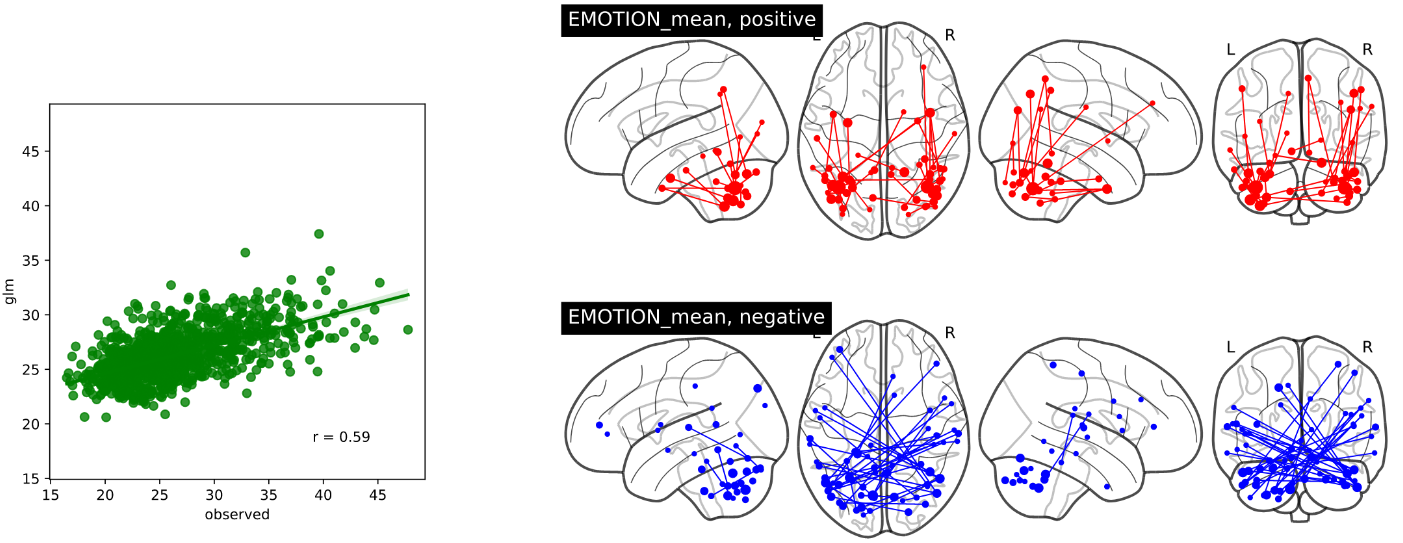Fig S1: Results of connectome‐based predictive modeling for averaged task conditions of the emotion task. The left panel plots observed body mass index vs. predicted body mass index; the right panel plots positive and negative predictive networks onto a glass brain |
| --- |

### Gambling task

The gambling task was designed to tap into incentive processing. Participants had to guess if the value of a card was less or more than five and would win one US dollar if correct and lose one if wrong. Accordingly, the gambling task was split into two conditions: loss and win. (Barch et al. 2013; Delgado et al. 2000)

Correlation of predicted with observed BMI was moderate for both conditions (r = 0.55; see supplemental figures and tables). For the *loss* condition, the positive predictive network was dominated by ipsilateral intracerebellar edges and edges connecting cerebellar nodes to ipsilateral temporal and parietal nodes, edges between contralateral cerebellar nodes and between cerebellar and contralateral temporal and parietal nodes govern the negative predictive network. The positive predictive network for the *win* condition featured left cerebellar nodes more heavily than right cerebellar nodes; connections were mainly ipsilaterally, while negative predictive networks were more contralaterally orientated. As for best-connected noncerebellar nodes, positively predicting nodes were evenly distributed between hemispheres with temporopolar nodes leading, followed by a range of parietal and occipital (including subdivisions of the visual cortex) nodes, while negatively predicting nodes were mostly located in the left hemisphere (loss > win).

By *averaging* task conditions, prediction improved (r = 0.59; see fig. S[2](#fig%252525253Aresults_gambling_mean)). Positive predictive edges connected left cerebellar nodes with ipsilateral temporal and parietal nodes and left cerebellar nodes with right temporal nodes with scarcely any intercerebellar edges. In contrast, the negative predictive network displayed intercerebellar connections, while edges connecting right cerebellar nodes to left cerebral (parietal, temporal, frontal) nodes had the most predictive power. Nodes with highest degrees within the positive predictive network included again bilateral temporopolar nodes and bilateral perirhinal cortices as well as visual areas, while within the negative predictive network, nodes in the left hemisphere were more numerous than those in the right and included temporal (excluding the poles), parietal, frontal (area 1 bilaterally), occipital and subcortical (left caudate nucleus and right putamen) nodes.

| 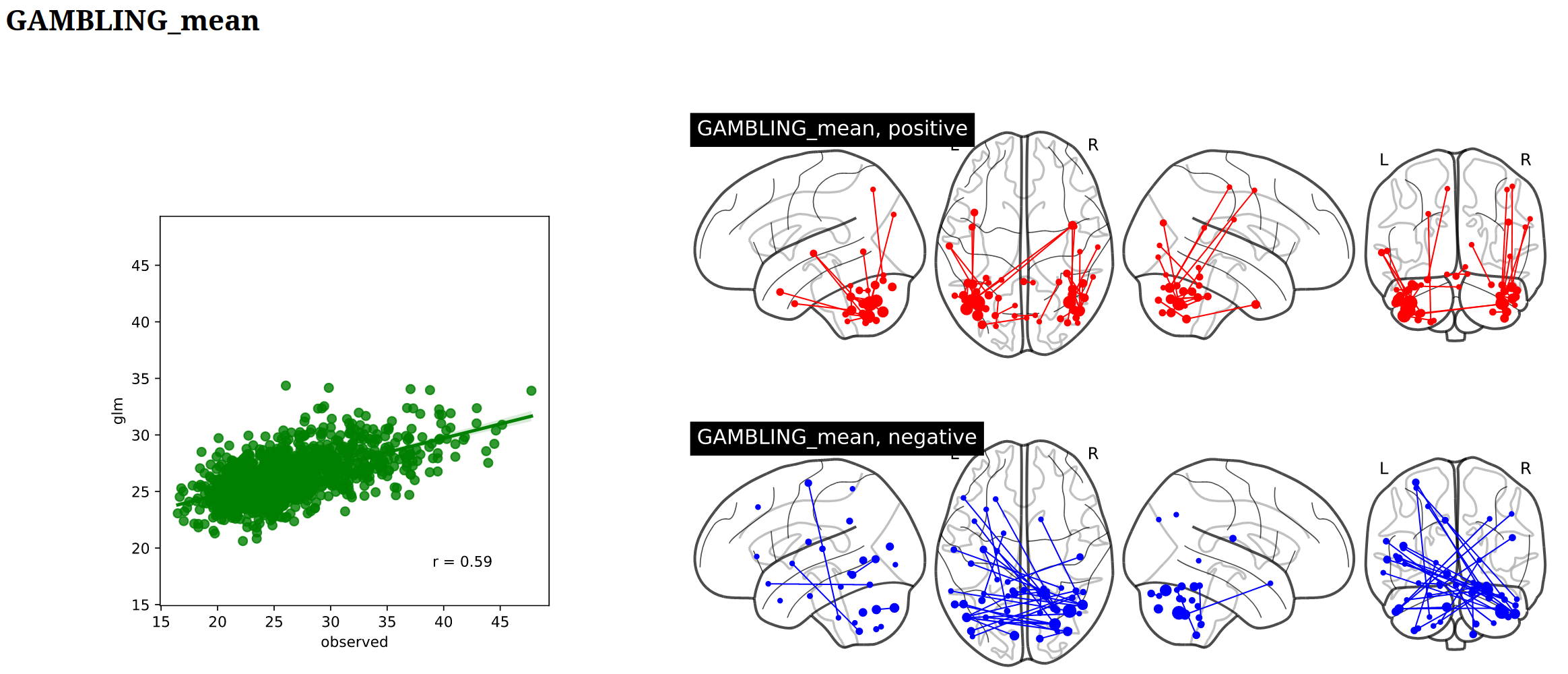Fig S2: Results of connectome‐based predictive modeling for averaged task conditions of the gambling task. The left panel plots observed body mass index vs. predicted body mass index; the right panel plots positive and negative predictive networks onto a glass brain |
| --- |

Language task

The language task consisted of the “story” condition testing semantic understanding and, for comparison, of the “math” condition where participants had to solve arithmetic tasks (Barch et al. 2013; Binder et al. 2011).

For the *math* condition, predictive power was better than for the story condition (r = 0.57 vs. r = 0.50); positive predictive networks involved cerebellar nodes in roughly equal numbers on both cerebellar hemispheres projecting mainly to bilateral (ipsilateral > contralateral) temporal (especially temporal poles, with bilateral cerebellar nodes projecting to the same nodes in the left temporal pole: L_TGd) nodes as well as to parietal (right > left hemisphere) and frontal (right hemisphere) nodes. In the negative predictive network, contralateral projections and the right cerebellum dominated, while right cerebellar nodes were also notably connected to ipsilateral frontal nodes. As with other tasks, leading best-connected nodes in the positive network were temporopolar nodes, followed by parietal nodes, although occipital nodes were absent. For the *story* condition, positive predictive networks consisted mainly of ipsilateral cerebellocerebal connections, while negative networks included more contralateral projections. Positive top nodes included again temporopolar nodes as well as nodes involved with auditory and visual processing. Negative top nodes of note were bilateral primary sensory cortices.

After *averaging*, correlation again improved (r = 0.62; see fig. S[3](#fig%252525253Aresults_language_mean)). The positive predictive network resembled the positive predictive network for the math condition, while the negative predictive network resembled the corresponding network for the story condition more closely. Bilateral temporopolar nodes topped the list of best-connected positive nodes, which also included temporal (left > right) nodes beyond the poles as well as left ventral and dorsal temporal bilateral ectorhinal cortices, and bilateral parietooccipital sulci. Part of the negative top nodes was a subdivision of the right thalamus and, as with the story condition, bilateral primary sensory cortices.

| 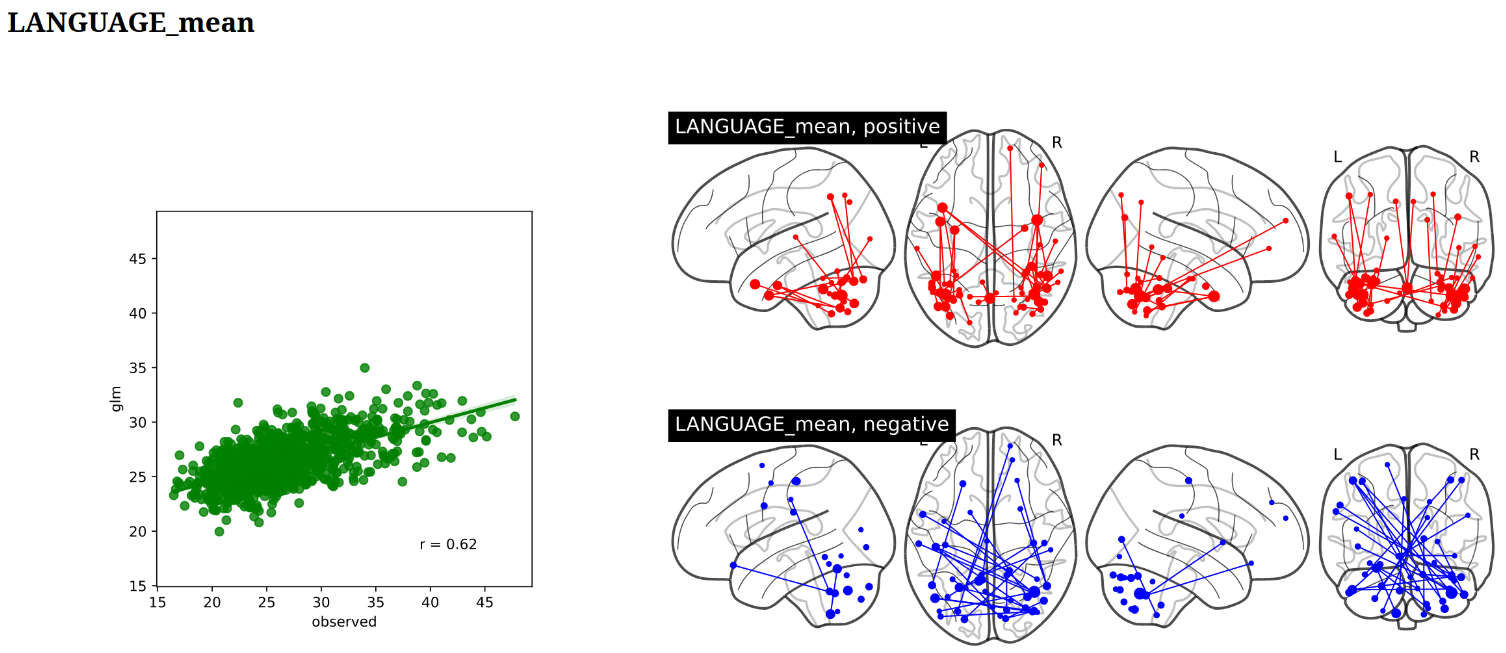Fig S3: Results of connectome‐based predictive modeling for averaged task conditions of the language task. The left panel plots observed body mass index vs. predicted body mass index; the right panel plots positive and negative predictive networks onto a glass brain |
| --- |

### Relational processing task

For the relational processing task, participants had to decide whether pairs of objects differed along the same dimensions (i.e. shape or texture) or if an object matched other objects with regard to a specified dimension; these different subtasks amounted to the “relational” and “match” condition, respectively (Barch et al. 2013; Smith, Keramatian, and Christoff 2007).

Overall predictive correlation was better for the *match* than for the *relational* condition (r = 0.54 vs. r = 0.47). Similar to other tasks, the positive predictive network tended to feature more ipsilateral connections with prominent projections to the temporal poles, while the negative predictive network connected cerebellar nodes to contralateral cerebellar and cerebral (predominantly parietal and frontal) nodes. Top negative nodes were accordingly located in temporal nodes but also included bilateral etorhinal cortices, while no temporopolar nodes made the list of top negative nodes, which contained nodes associated with auditory processing in a symmetrical manner. As for the positive predictie network in the *relational* condition, left cerebellar nodes tended to project ipsilaterally (especially temporal poles), while right cerebellar nodes were mostly (and unusually in relation to other tasks (?)) connected to contralateral cerebral nodes, while the negative predictive network predominantly was made up of contralateral cerebellocerebral and intercerebellar connections. Bilateral ectorhinal cortices were again part of the top positive node, while within the negative predictive network, left caudate nucleus and right putamen had a prominent role to play.

The *averaged* relational processing task yielded a better prediction than conditions on their own (r = 0.61, see fig. S[4](#fig%252525253Aresults_relational_mean)). Predictive networks showed a pattern of contralateral and ipsilateral projections to temporal and parietal nodes for the positive network, while the negative predictive network had contralateral cerebellocerebral connections as its backbone. Best-connected positive nodes were again temporopolar ones and bilateral ectorhinal cortices; best-connected negative nodes were a mixture of the corresponding list of the solitary conditions (auditory, parietal and frontal nodes).

| 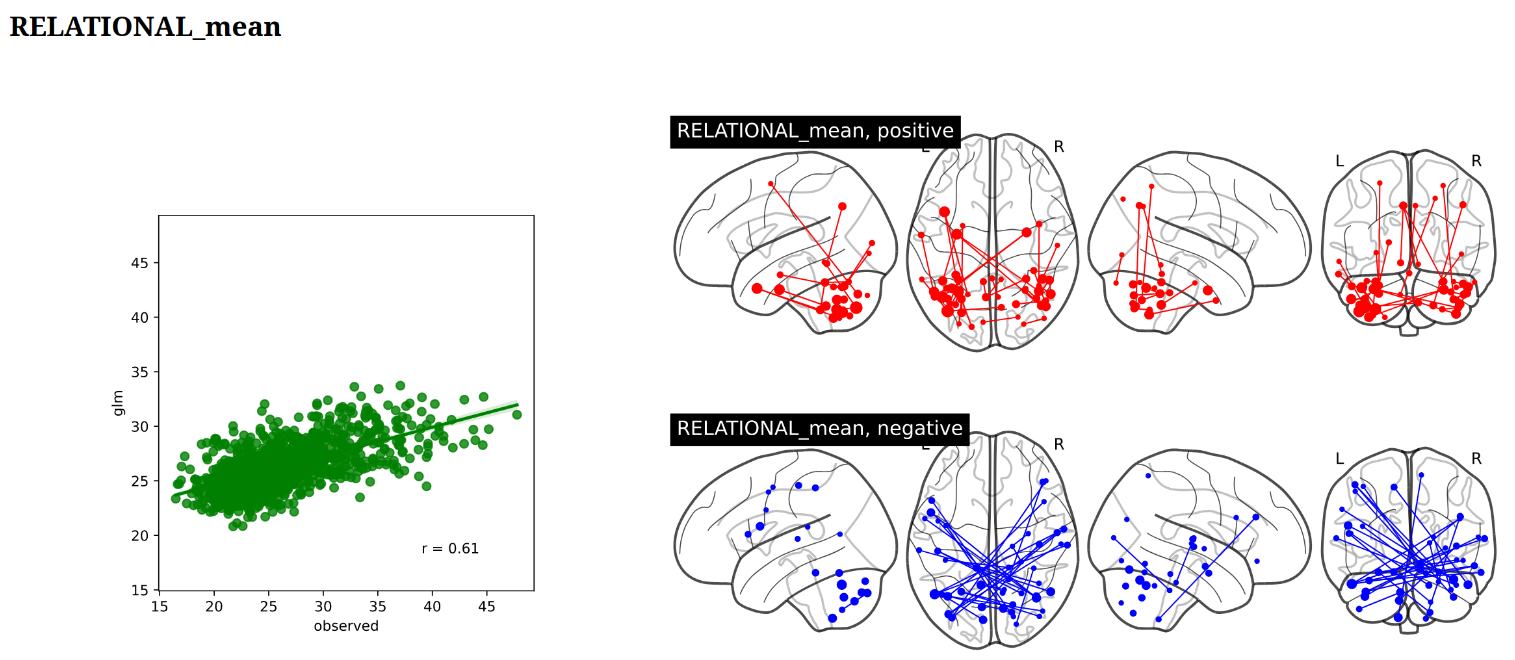Fig S4: Results of connectome‐based predictive modeling for averaged task conditions of the relational processing task. The left panel plots observed body mass index vs. predicted body mass index; the right panel plots positive and negative predictive networks onto a glass brain |
| --- |

### Social cognition task

During the social cognition task, designed to test participants’ theory of mind, geometrical objects were displayed interacting (“mental” condition) or moving randomly (“random” condition) and participants had to decide whether movement of those objects represented social interaction (Barch et al. 2013; Castelli et al. 2000; Wheatley, Milleville, and Martin 2007).

Predictive power for the mental and random condition were quite similar (r = 0.53 vs. r = 0.55, respectively). With regard to the *mental* condition, the positive predictive network was orientated towards ipsilateral connections between the cerebellum and temporal nodes, striking were edges connecting left cerebellar nodes to contralateral prefrontal nodes; intercerebellar edges (spanning cerebellar hemispheres) were sparse. Top positive nodes were again located in bilateral temporal poles, ectorhinal cortices and included the left hippocampus as a subcortical node. Negative predictive networks showed the usual pattern of contralateral cerebellocerebral edges, with top nodes showing a predominance of the left cerebral hemisphere. As for the *random* condition, positively predicting edges were mostly intracerebellar (i.e. within one cerebellar hemisphere), top nodes notably included, besides temporopolar nodes, the left primary sensory area, right visual area 2 and the left ventroposterior thalamus as well as bilateral nodes located in the parietoccipital sulcus.

After averaging conditions, predictive power improved (r = 0.62; see fig. S[5](#fig%252525253Aresults_social_mean)). The positive predictive network was mainly driven by intracerebellar edges on top of ipsilateral and right cerebellum to left cerebrum connections to temporal nodes with edges to frontal nodes, featuring prominently in the task conditions, being absent. The negative predictive network was again more contralaterally orientated. Best-connected cerebral nodes included the usual temporopolar and ectorhinal ones for the positive list, while the negative list lacked temporopolar nodes and featured nodes implicated in auditory pathways.

| 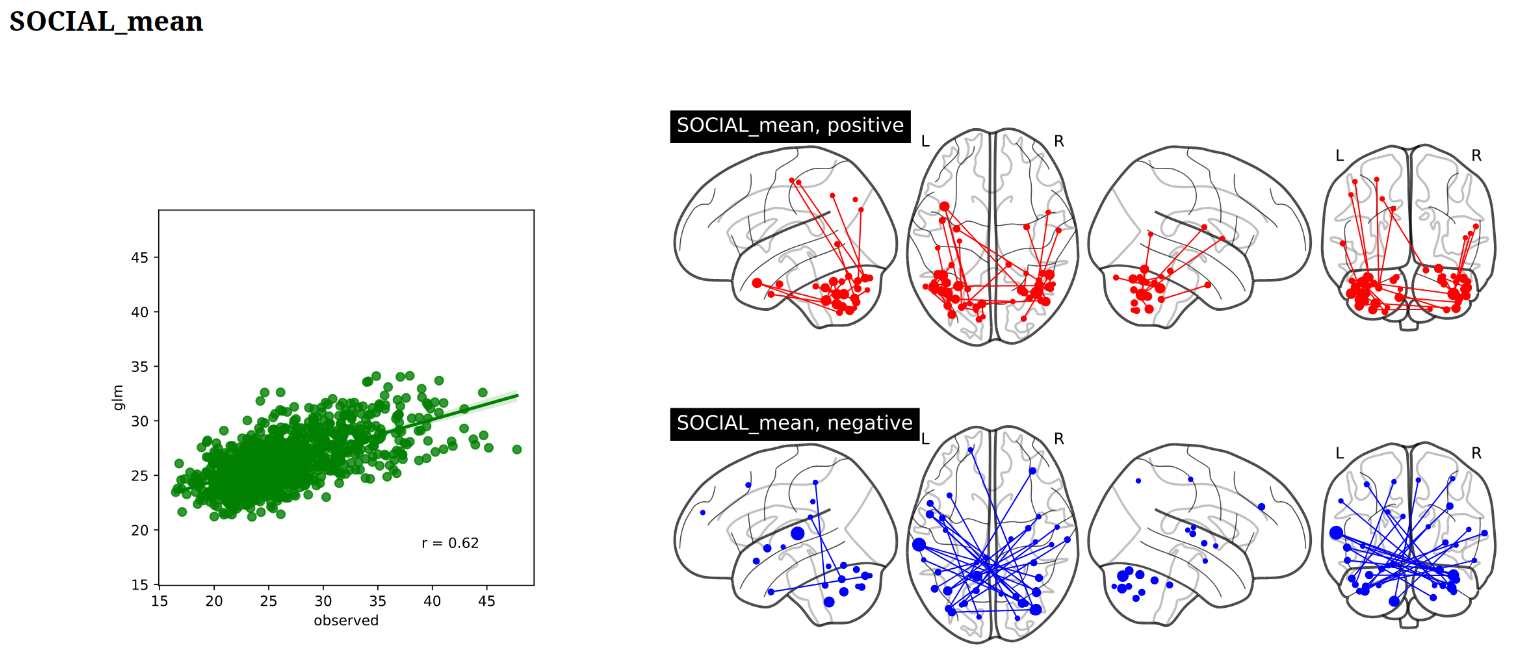Fig S5: Results of connectome‐based predictive modeling for averaged task conditions of the relational processing task. The left panel plots observed body mass index vs. predicted body mass index; the right panel plots positive and negative predictive networks onto a glass brain |
| --- |

### Working-memory task

The working-memory task was a variant of N-back tasks, subjects were presented with pictures of faces, places, body parts and tools in the 0-back and 2-back fashion (Barch et al. 2013). As we were more interested in working memory as an executive-function subdomain than in the localizing function of different picture categories (Downing et al. 2001), we combined all 0-back runs and all 2-back runs into two conditions.

Predictive power was the same for both conditions (r = 0.62). As with other tasks, intracerebellar and ipsilateral and contraleteral cerbellotemporal and cerebelloparietal edges could be observed for the positive predictive networks, with the *2-back* condition also showing ipsilateral and contralateral cerebellofrontal edges. In contrast, negative predictive networks displayed almost exclusively contralateral edges between cerebellar and temporal and parietal edges (*both* conditions) with projections to left (*2-back*) and right (*0-back*) frontal nodes. Besides the usual temporopolar and ectorhinal nodes, best-connected positive nodes also included bilateral (*0-back*) and right frontal areas (*2-back*) as well as right lateral parietal (*both conditions*), left lateral parietal (*2-back*) and left-medial parietal (*0-back*) nodes. Unusually, among the list of positives for the *2-back* condition, auditory complex 4 showed up.

*Averaging* the two task conditions into one single working-memory task produced better predictions (r = 0.66; see fig. S[6](#fig%252525253Aresults_wm_mean)). Predictive networks were similar to the 0-back condition’s, while the positive additionally included edges connecting cerebellar to prefrontal nodes. The negative predictive network was, with regard to cerebral projections of the cerebellum, right-dominant, while also including a wealth of intercerebellar edges. Best-connected noncerebellar nodes within the positive predictive network included bilateral temporopolar nodes, ectorhinal cortices, visual areas as well as lateral parietal (right hemisphere) and medial parietal (left hemisphere) nodes. Top nodes (by degree) within the negative predictive network were predominantly located in the left hemisphere and included primary sensory areas in both hemispheres as well as parietal (left > right) and prefrontal nodes.

| 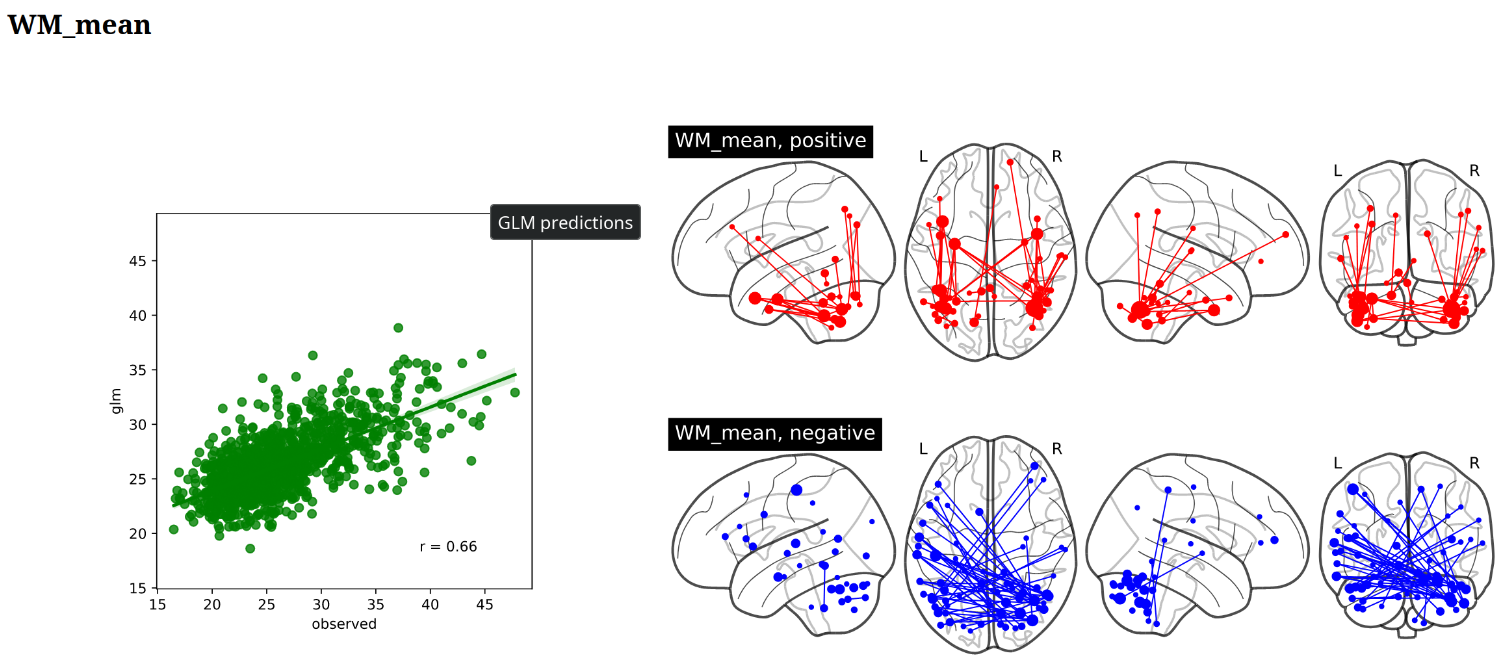Fig S6: Results of connectome‐based predictive modeling for averaged task conditions of the working-memory task. The left panel plots observed body mass index vs. predicted body mass index; the right panel plots positive and negative predictive networks onto a glass brain |
| --- |

# S2: Cerebellar nodes and histogram plots of their degrees


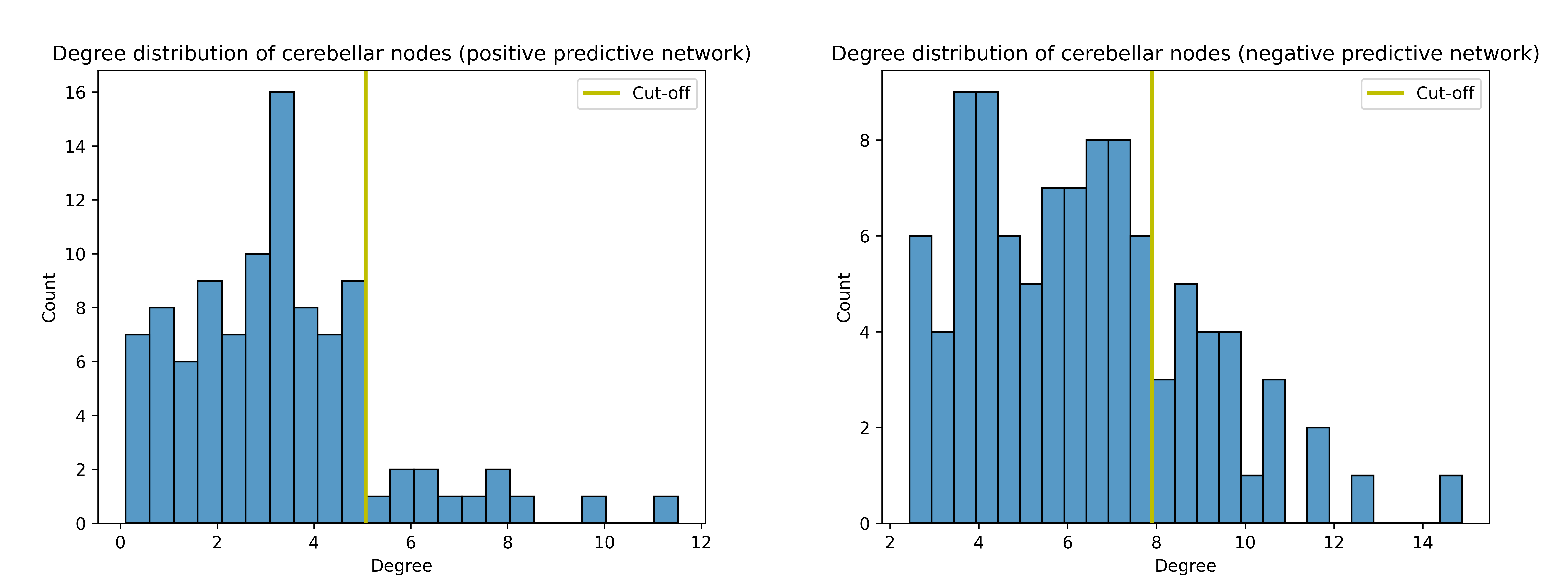
Fig S7: Histograms of cerebellar nodes' degree distribution in the positive (left figure) and negative (right figure) predictive task-general networks (all tasks averaged)f

# S3: Overlapping predictive networks

## Overlap with Wisconsin card sorting task


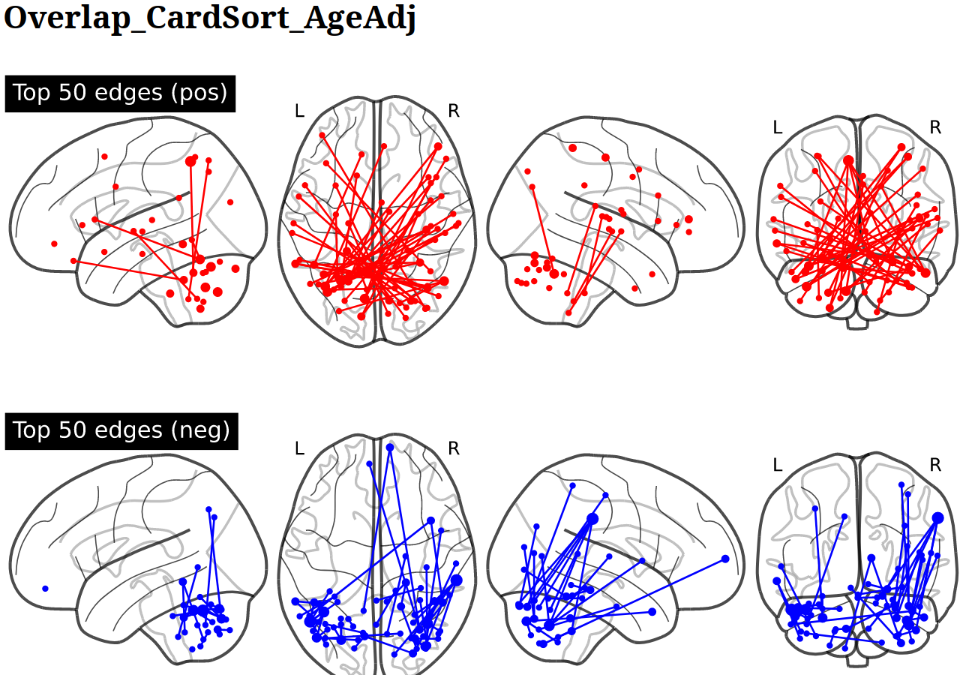
Fig S8: Positive (in red) and negative (in blue) predictive network overlap of connectome-based predictive modeling for body-mass index and Wisconsin card sorting task performance


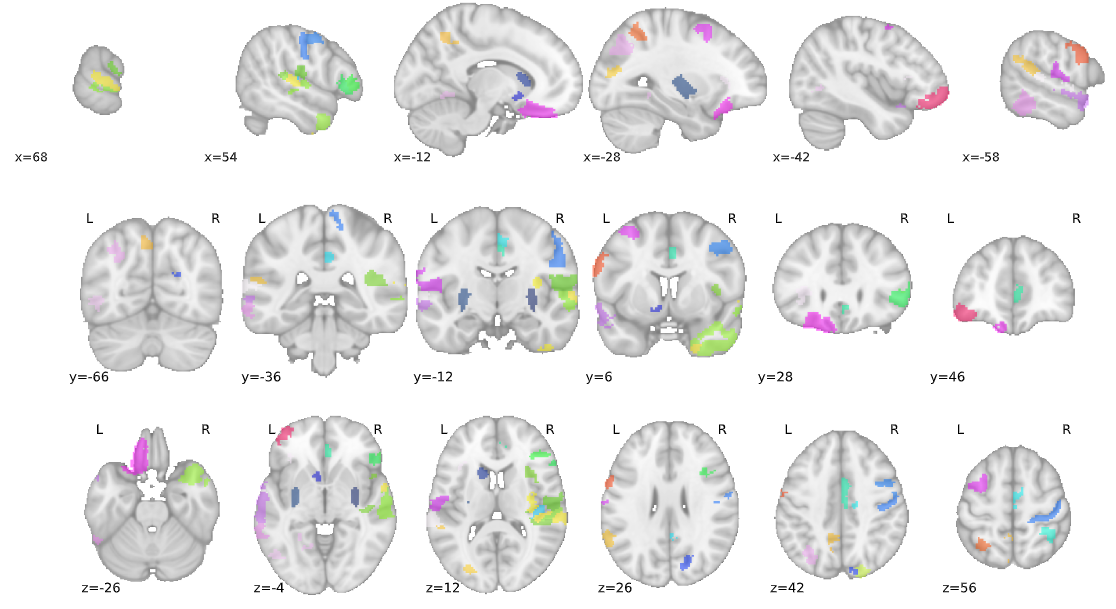


|  | Node | Degree | Label | Name | MNI coordinates | Color |
| --- | --- | --- | --- | --- | --- | --- |
| 5 | 360 | 0.419232 | L_PCV | Left PreCuneus Visual Area | -6.31 -52.4 50.04 |  |
| 11 | 466 | 0.361968 | L_TE1p | Left Area TE1 posterior | -58.29 -46.77 -9.99 |  |
| 14 | 162 | 0.332879 | R_3b | Right Primary Sensory Cortex | 36.81 -22.03 52.84 |  |
| 16 | 508 | 0.317496 | L_A4 | Left Auditory 4 Complex | -60.62 -24.47 7.55 |  |
| 20 | 433 | 0.268059 | L_OP4 | Left AreaOP4/PV | -55.9 -13.38 15.43 |  |
| 22 | 200 | 0.250557 | R_7PC | Right Area 7PC | 32.12 -45.87 59.78 |  |
| 24 | 235 | 0.234902 | R_IFSa | Right Area IFSa | 45.2 38.3 8.08 |  |
| 25 | 458 | 0.223784 | L_A5 | Left Auditory 5 Complex | -59.57 -17.43 -0.75 |  |
| 26 | 256 | 0.205245 | R_52 | Right Area 52 | 39.96 -19.49 0.99 |  |
| 27 | 486 | 0.202811 | L_VMV1 | Left VentroMedial Visual Area 1 | -19.57 -53.32 -6.83 |  |
| 28 | 381 | 0.201854 | L_LIPv | Left Area Lateral IntraParietal ventral | -27.97 -55.67 53.16 |  |
| 29 | 502 | 0.201687 | L_FOP5 | Left Area Frontal Opercular 5 | -35.99 26.1 3.9 |  |
| 30 | 277 | 0.200078 | R_PBelt | Right ParaBelt Complex | 58.2 -19.32 8.4 |  |
| 35 | 321 | 0.190783 | R_Ig | Right Insular Granular Complex | 37.54 -11.35 12.99 |  |
| 39 | 257 | 0.183503 | R_RI | Right RetroInsular Cortex | 43.04 -29.43 17.69 |  |
| 40 | 150 | 0.182448 | NAc-shell-lh | Left nucleus accumbens, shell | -9.07 10.87 -6.94 |  |
| 41 | 128 | 0.181045 | HIP-head-m2-lh | Left hippocampus head, medial division, subdivision 2 | -19.85 -17.46 -17.07 |  |
| 42 | 328 | 0.180179 | R_A4 | Right Auditory 4 Complex | 63.44 -20.25 7.24 |  |
| 48 | 425 | 0.167265 | L_13l | Left Area 13l | -22.3 27.63 -17.97 |  |
| 49 | 490 | 0.166495 | L_FST | Left Area FST | -45.82 -67.54 1.53 |  |
| 50 | 193 | 0.165448 | R_24dd | Right Dorsal Area 24d | 8.0 -16.1 50.36 |  |

## Overlap with Eriksen flanker task


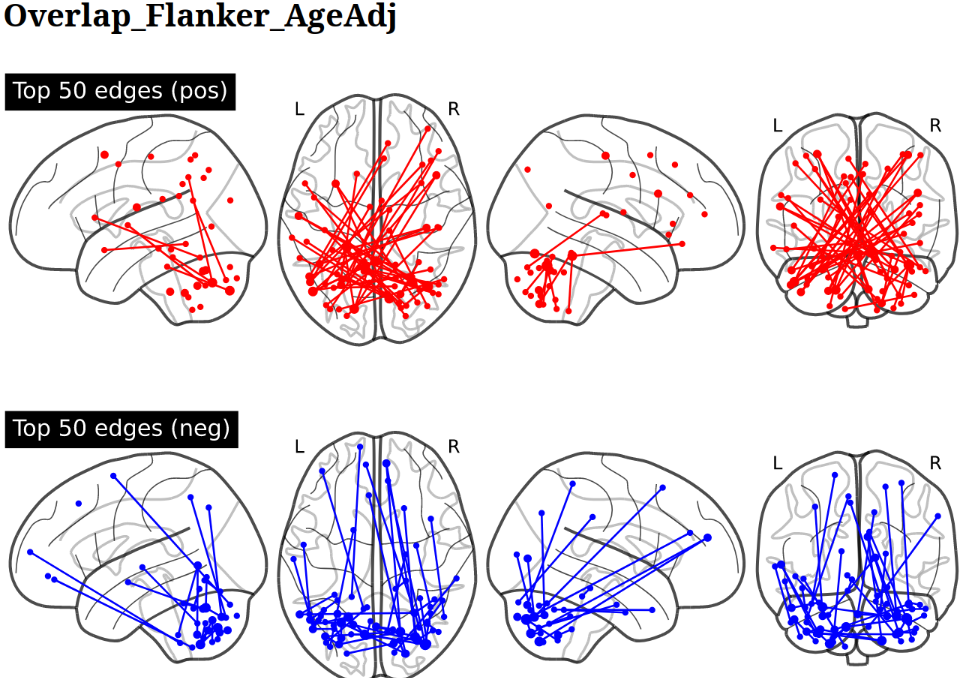
Fig S9: Positive (in red) and negative (in blue) predictive network overlap of connectome-based predictive modeling for body-mass index and Eriksen flanker task performance


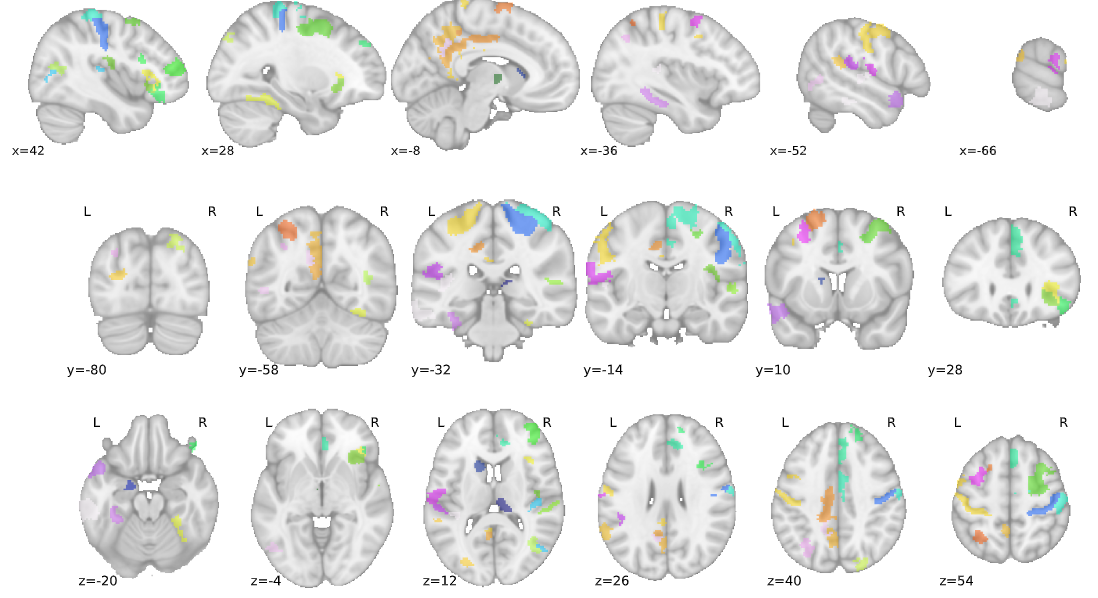


|  | Node | Degree | Label | Name | MNI coordinates | Color |
| --- | --- | --- | --- | --- | --- | --- |
| 9 | 162 | 0.236317 | R_3b | Right Primary Sensory Cortex | 36.81 -22.03 52.84 |  |
| 10 | 430 | 0.222305 | L_i6-8 | Left Inferior 6-8 Transitional Area | -29.08 9.98 53.54 |  |
| 17 | 232 | 0.185201 | R_IFJa | Right Area IFJa | 43.95 16.08 25.36 |  |
| 21 | 433 | 0.176892 | L_OP4 | Left AreaOP4/PV | -55.9 -13.38 15.43 |  |
| 22 | 204 | 0.174081 | R_1 | Right Area 1 | 45.89 -22.03 52.67 |  |
| 23 | 438 | 0.170763 | L_PFcm | Left Area PFcm | -50.13 -32.05 21.46 |  |
| 24 | 490 | 0.168354 | L_FST | Left Area FST | -45.82 -67.54 1.53 |  |
| 29 | 363 | 0.115504 | L_7m | Left Area 7m | -4.57 -61.87 36.73 |  |
| 30 | 238 | 0.113316 | R_a9-46v | Right Area anterior 9-46v | 37.71 49.79 10.41 |  |
| 31 | 360 | 0.112259 | L_PCV | Left PreCuneus Visual Area | -6.31 -52.4 50.04 |  |
| 32 | 345 | 0.112228 | L_55b | Left Area 55b | -44.73 -0.04 46.72 |  |
| 34 | 352 | 0.110966 | L_V3B | Left Area V3B | -26.64 -81.17 20.45 |  |
| 35 | 305 | 0.108754 | R_V6A | Right Area V6A | 22.11 -78.54 42.81 |  |
| 36 | 264 | 0.103059 | R_AVI | Right Anterior Ventral Insular Area | 33.85 23.73 -4.4 |  |
| 37 | 109 | 0.102963 | THA-VAs-rh | Right superior ventroanterior thalamus | 9.83 -9.05 11.9 |  |
| 38 | 494 | 0.101619 | L_31pd | Left Area 31pd | -9.58 -50.81 37.27 |  |
| 39 | 381 | 0.100786 | L_LIPv | Left Area Lateral IntraParietal ventral | -27.97 -55.67 53.16 |  |
| 40 | 229 | 0.100649 | R_47l | Right Area 47l (47 lateral) | 45.53 33.51 -11.07 |  |
| 43 | 144 | 0.099297 | CAU-DA-lh | Left dorsoanterior caudate | -13.7 17.14 8.0 |  |
| 44 | 210 | 0.098359 | R_p24pr | Right Area Posterior 24 prime | 4.25 -2.38 38.86 |  |

## Overlap with Penn Matrix Reasoning Test


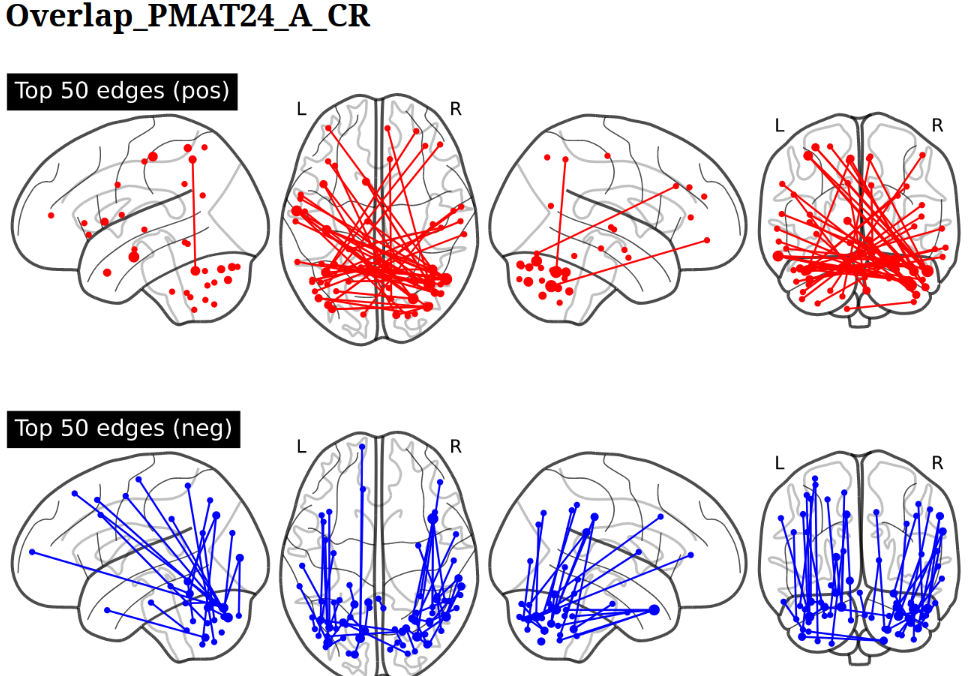
Fig S10: Positive (in red) and negative (in blue) predictive network overlap of connectome-based predictive modeling for body-mass index and Penn Matrix Reasoning Test performance

##
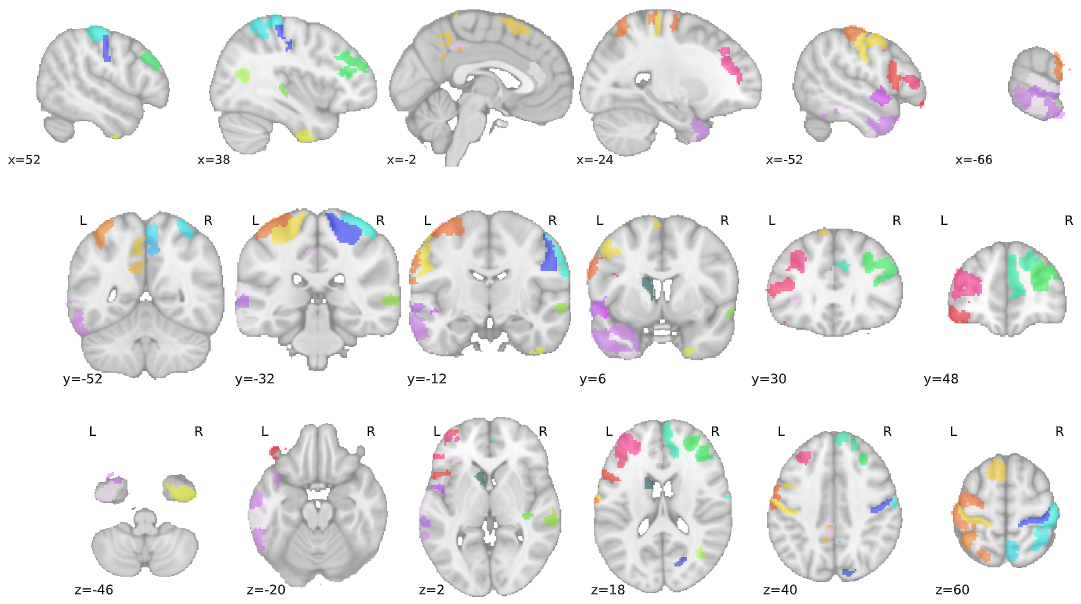


|  | Node | Degree | Label | Name | MNI coordinates | Color |
| --- | --- | --- | --- | --- | --- | --- |
| 28 | 180 | 0.859235 | R_PCV | Right PreCuneus Visual Area | 7.12 -52.51 50.13 |  |
| 31 | 236 | 0.824355 | R_p9-46v | Right Area posterior 9-46v | 43.02 32.55 25.82 |  |
| 39 | 382 | 0.704447 | L_VIP | Left Ventral IntraParietal Complex | -21.07 -61.03 58.84 |  |
| 42 | 465 | 0.671271 | L_TE1a | Left Area TE1 anterior | -58.78 -9.83 -20.57 |  |
| 47 | 204 | 0.634751 | R_1 | Right Area 1 | 45.89 -22.03 52.67 |  |
| 48 | 502 | 0.628718 | L_FOP5 | Left Area Frontal Opercular 5 | -35.99 26.1 3.9 |  |
| 53 | 342 | 0.601235 | L_3b | Left Primary Sensory Cortex | -37.01 -23.63 52.15 |  |
| 55 | 239 | 0.591863 | R_9-46d | Right Area 9-46d | 27.94 48.03 23.21 |  |
| 58 | 387 | 0.579439 | L_6d | Left Dorsal area 6 | -32.02 -13.25 62.94 |  |
| 60 | 360 | 0.574752 | L_PCV | Left PreCuneus Visual Area | -6.31 -52.4 50.04 |  |
| 62 | 464 | 0.568906 | L_TGd | Left Area TG dorsal | -39.32 9.55 -31.94 |  |
| 64 | 118 | 0.557294 | CAU-body-rh | Right caudate body | 14.08 5.65 15.52 |  |
| 65 | 380 | 0.546970 | L_7PC | Left Area 7PC | -31.68 -48.91 58.32 |  |
| 67 | 162 | 0.526964 | R_3b | Right Primary Sensory Cortex | 36.81 -22.03 52.84 |  |
| 68 | 222 | 0.523761 | R_9m | Right Area 9 Middle | 7.69 51.95 20.72 |  |
| 71 | 145 | 0.504866 | CAU-body-lh | Left caudate body | -12.25 5.47 15.33 |  |
| 73 | 143 | 0.501297 | CAU-VA-lh | Left ventroanterior caudate | -8.49 11.35 4.96 |  |
| 74 | 466 | 0.486625 | L_TE1p | Left Area TE1 posterior | -58.29 -46.77 -9.99 |  |
| 76 | 215 | 0.471993 | R_d32 | Right Area dorsal 32 | 8.81 39.27 24.24 |  |
| 77 | 412 | 0.466975 | L_IFJa | Left Area IFJa | -42.34 13.25 25.61 |  |
| 78 | 237 | 0.460214 | R_46 | Right Area 46 | 34.41 37.31 29.86 |  |
| 79 | 200 | 0.457193 | R_7PC | Right Area 7PC | 32.12 -45.87 59.78 |  |
| 83 | 169 | 0.444619 | R_V7 | Right Seventh Visual Area | 27.6 -79.1 33.5 |  |
| 85 | 505 | 0.441260 | L_TGv | Left Area TG Ventral | -41.2 -2.21 -41.38 |  |
| 86 | 389 | 0.434827 | L_6v | Left Ventral Area 6 | -55.79 1.93 31.74 |  |
| 87 | 508 | 0.412500 | L_A4 | Left Auditory 4 Complex | -60.62 -24.47 7.55 |  |

## Overlap with Delay Discounting task (200 condition)


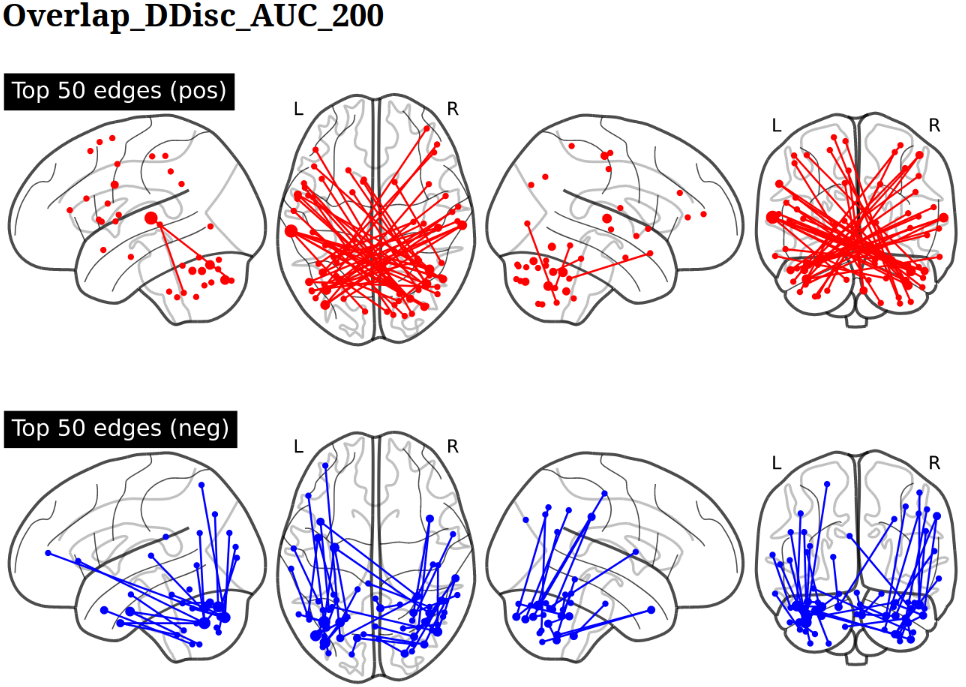
Fig S11: Positive (in red) and negative (in blue) predictive network overlap of connectome-based predictive modeling for body-mass index and Wisconsin card sorting ta (in blue) predictive network overlap of connectome-based predictive modeling for body-mass index and Delay Discounting task (200 condition) performance


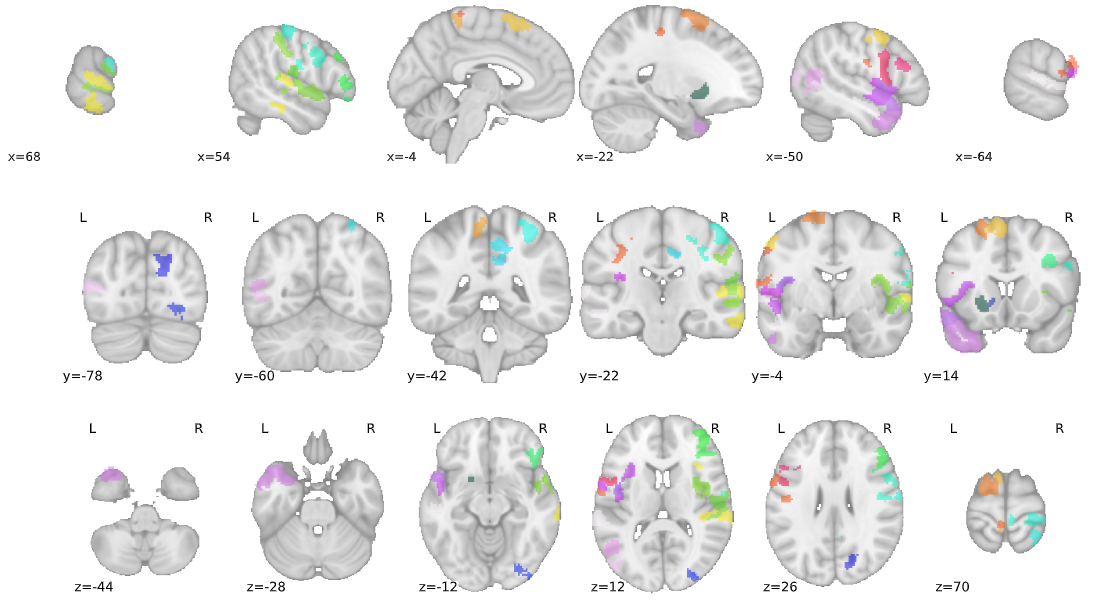


|  | Node | Degree | Label | Name | MNI coordinates | Color |
| --- | --- | --- | --- | --- | --- | --- |
| 0 | 508 | 1.806119 | L_A4 | Left Auditory 4 Complex | -60.62 -24.47 7.55 |  |
| 18 | 328 | 0.503248 | R_A4 | Right Auditory 4 Complex | 63.44 -20.25 7.24 |  |
| 25 | 204 | 0.454730 | R_1 | Right Area 1 | 45.89 -22.03 52.67 |  |
| 26 | 411 | 0.432918 | L_6r | Left Rostral Area 6 | -50.68 7.0 18.08 |  |
| 37 | 151 | 0.366658 | NAc-core-lh | Left nucleus accumbens, core | -12.73 18.59 -2.92 |  |
| 38 | 473 | 0.361003 | L_TPOJ2 | Left Area TemporoParietoOccipital Junction 2 | -48.42 -59.92 11.44 |  |
| 41 | 253 | 0.336497 | R_OP4 | Right AreaOP4/PV | 56.19 -10.6 14.84 |  |
| 45 | 432 | 0.323845 | L_43 | Left Area 43 | -56.12 -1.03 9.93 |  |
| 47 | 260 | 0.315065 | R_TA2 | Right Area TA2 | 51.39 1.0 -5.42 |  |
| 50 | 188 | 0.309597 | R_31pv | Right Area 31p ventral | 8.92 -46.27 32.54 |  |
| 52 | 414 | 0.300266 | L_IFSp | Left Area IFSp | -43.96 22.44 21.72 |  |
| 53 | 448 | 0.298464 | L_FOP2 | Left Frontal OPercular Area 2 | -40.48 -4.48 13.62 |  |
| 55 | 435 | 0.279348 | L_OP2-3 | Left AreaOP2-3/VS | -37.8 -17.8 17.54 |  |
| 58 | 464 | 0.267747 | L_TGd | Left Area TG dorsal | -39.32 9.55 -31.94 |  |
| 60 | 122 | 0.267692 | THA-DP-rh | Right dorsoposterior thalamus | 16.96 -30.83 2.88 |  |
| 63 | 389 | 0.259994 | L_6v | Left Ventral Area 6 | -55.79 1.93 31.74 |  |
| 66 | 235 | 0.235618 | R_IFSa | Right Area IFSa | 45.2 38.3 8.08 |  |
| 67 | 238 | 0.234420 | R_a9-46v | Right Area anterior 9-46v | 37.71 49.79 10.41 |  |
| 68 | 456 | 0.231834 | L_STGa | Left Area STGa | -51.29 10.13 -15.57 |  |
| 69 | 359 | 0.228295 | L_SFL | Left Superior Frontal Language Area | -7.79 12.8 62.7 |  |
| 70 | 156 | 0.228049 | R_V6 | Right Sixth Visual Area | 17.41 -75.2 31.57 |  |
| 72 | 206 | 0.225305 | R_3a | Right Area 3a | 35.5 -19.05 43.13 |  |
| 75 | 345 | 0.224447 | L_55b | Left Area 55b | -44.73 -0.04 46.72 |  |

## Overlap with Delay Discounting task (40k condition)

##
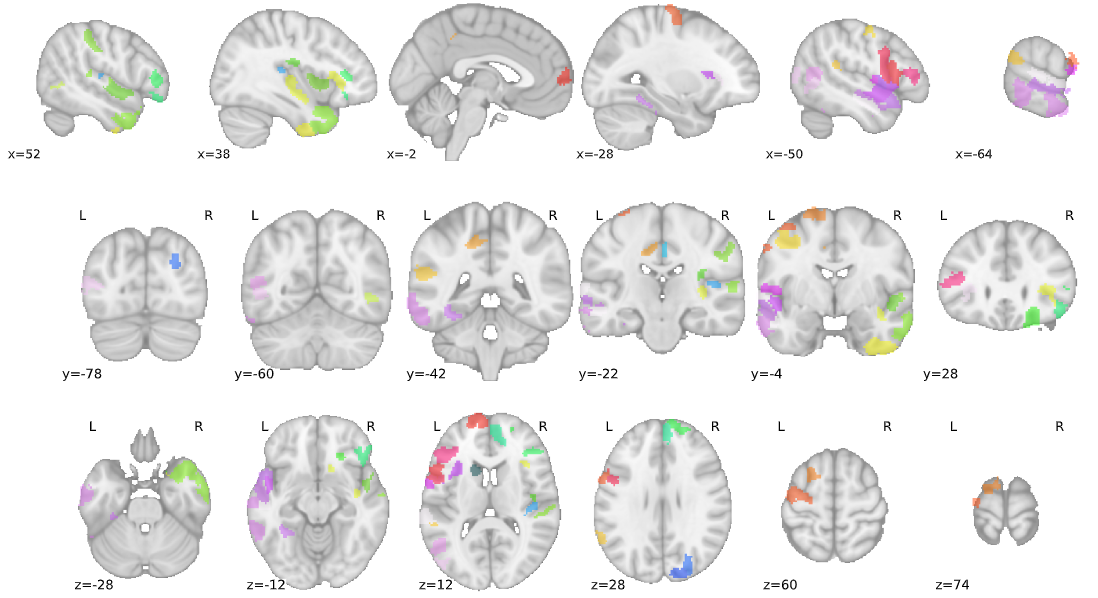


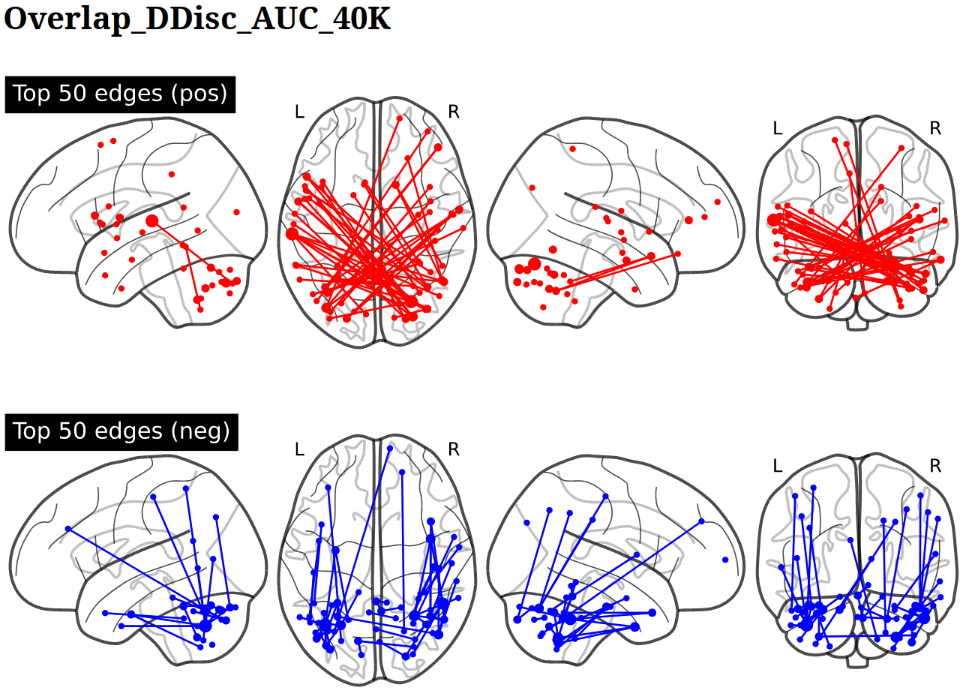
Fig S12: Positive (in red) and negative (in blue) predictive network overlap of connectome-based predictive modeling for body-mass index and Wisconsin card sorting ta (in blue) predictive network overlap of connectome-based predictive modeling for body-mass index and Delay Discounting task (40 k condition) performance

|  | Node | Degree | Label | Name | MNI coordinates | Color |
| --- | --- | --- | --- | --- | --- | --- |
| 2 | 508 | 1.131207 | L_A4 | Left Auditory 4 Complex | -60.62 -24.47 7.55 |  |
| 13 | 235 | 0.652132 | R_IFSa | Right Area IFSa | 45.2 38.3 8.08 |  |
| 22 | 285 | 0.445802 | R_TE1a | Right Area TE1 anterior | 60.49 -6.86 -21.14 |  |
| 23 | 441 | 0.436192 | L_FOP4 | Left Frontal OPercular Area 4 | -38.38 13.42 6.46 |  |
| 24 | 440 | 0.418784 | L_TA2 | Left Area TA2 | -51.57 0.9 -4.87 |  |
| 28 | 458 | 0.392345 | L_A5 | Left Auditory 5 Complex | -59.57 -17.43 -0.75 |  |
| 32 | 320 | 0.382112 | R_PoI1 | Right Area Posterior Insular 1 | 39.11 -8.73 -6.24 |  |
| 34 | 432 | 0.374901 | L_43 | Left Area 43 | -56.12 -1.03 9.93 |  |
| 36 | 319 | 0.357661 | R_pOFC | Right posterior OFC Complex | 14.39 11.06 -17.92 |  |
| 38 | 456 | 0.338836 | L_STGa | Left Area STGa | -51.29 10.13 -15.57 |  |
| 44 | 240 | 0.298148 | R_9a | Right Area 9 anterior | 17.26 59.18 21.32 |  |
| 45 | 169 | 0.296661 | R_V7 | Right Seventh Visual Area | 27.6 -79.1 33.5 |  |
| 46 | 260 | 0.293781 | R_TA2 | Right Area TA2 | 51.39 1.0 -5.42 |  |
| 47 | 490 | 0.293765 | L_FST | Left Area FST | -45.82 -67.54 1.53 |  |
| 48 | 166 | 0.292310 | R_V3A | Right Area V3A | 20.75 -85.77 28.21 |  |
| 49 | 377 | 0.291770 | L_6ma | Left Area 6m anterior | -16.17 3.62 65.51 |  |
| 50 | 277 | 0.287424 | R_PBelt | Right ParaBelt Complex | 58.2 -19.32 8.4 |  |
| 51 | 411 | 0.286785 | L_6r | Left Rostral Area 6 | -50.68 7.0 18.08 |  |
| 53 | 473 | 0.281641 | L_TPOJ2 | Left Area TemporoParietoOccipital Junction 2 | -48.42 -59.92 11.44 |  |
| 55 | 118 | 0.277624 | CAU-body-rh | Right caudate body | 14.08 5.65 15.52 |  |
| 56 | 284 | 0.274703 | R_TGd | Right Area TG dorsal | 39.95 11.8 -31.73 |  |
| 57 | 343 | 0.273109 | L_FEF | Left Frontal Eye Fields | -38.08 -3.84 50.85 |  |
| 59 | 407 | 0.257917 | L_44 | Left Area 44 | -50.01 17.01 11.43 |  |
| 60 | 465 | 0.249993 | L_TE1a | Left Area TE1 anterior | -58.78 -9.83 -20.57 |  |
| 61 | 245 | 0.236819 | R_13l | Right Area 13l | 23.11 30.35 -16.3 |  |
| 62 | 466 | 0.233539 | L_TE1p | Left Area TE1 posterior | -58.29 -46.77 -9.99 |  |
| 63 | 177 | 0.223064 | R_A1 | Right Primary Auditory Cortex | 44.82 -21.49 9.69 |  |
| 64 | 262 | 0.219502 | R_MI | Right Middle Insular Area | 39.2 9.55 -0.23 |  |
| 65 | 325 | 0.216341 | R_TGv | Right Area TG Ventral | 39.81 -0.81 -42.01 |  |
| 66 | 387 | 0.215669 | L_6d | Left Dorsal area 6 | -32.02 -13.25 62.94 |  |
| 68 | 358 | 0.214176 | L_PSL | Left PeriSylvian Language Area | -55.13 -43.95 23.61 |  |
| 72 | 446 | 0.207741 | L_FOP1 | Left Frontal OPercular Area 1 | -48.22 1.32 5.14 |  |
| 75 | 405 | 0.203921 | L_10d | Left Area 10d | -11.36 63.95 10.27 |  |
| 76 | 502 | 0.203713 | L_FOP5 | Left Area Frontal Opercular 5 | -35.99 26.1 3.9 |  |
| 78 | 389 | 0.201232 | L_6v | Left Ventral Area 6 | -55.79 1.93 31.74 |  |
| 79 | 492 | 0.199707 | L_LO3 | Left Area Lateral Occipital 3 | -42.01 -79.06 11.72 |  |
| 80 | 269 | 0.199360 | R_PFt | Right Area PFt | 55.2 -22.94 39.54 |  |
| 83 | 461 | 0.196626 | L_STSda | Left Area STSd anterior | -52.33 -7.04 -11.55 |  |
| 85 | 144 | 0.194980 | CAU-DA-lh | Left dorsoanterior caudate | -13.7 17.14 8.0 |  |

# S4: Results of permutation testing


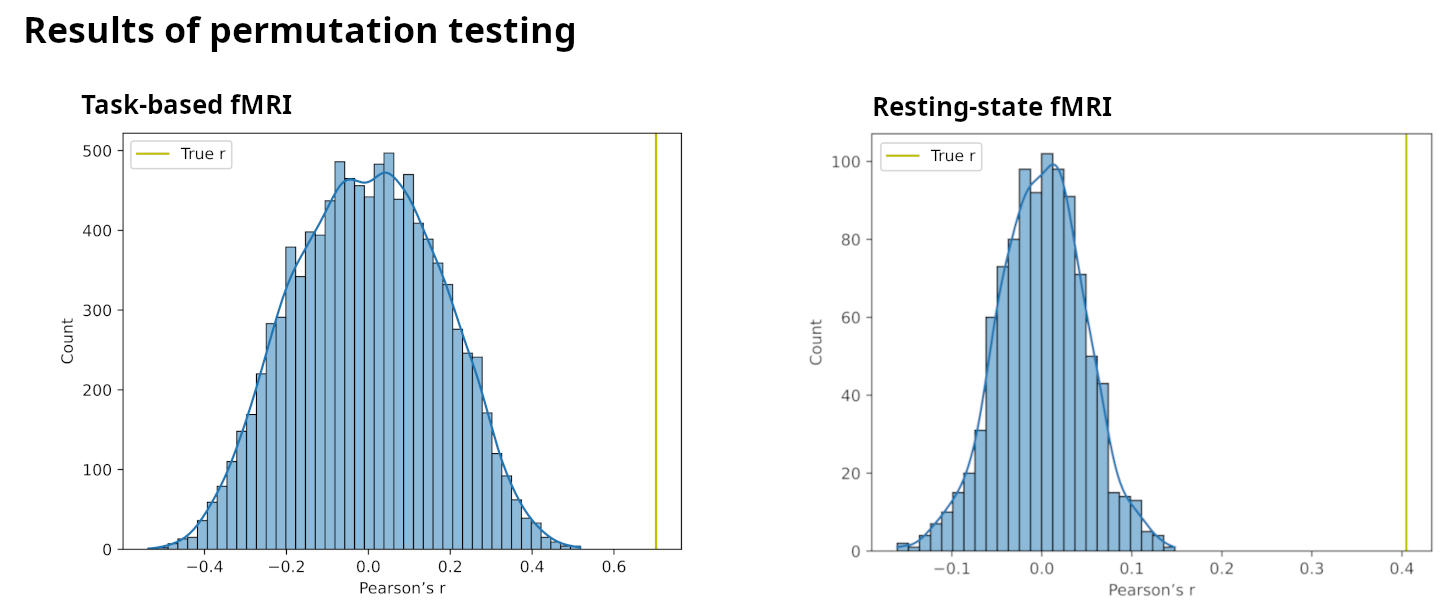
Fig S13: Bar plot of permutation results for all tasks combined (averaged); the vertical yellow line marks the actual correlation level achieved using non-permuted data.

# Literature

Barch, Deanna M., Gregory C. Burgess, Michael P. Harms, Steven E. Petersen, Bradley L. Schlaggar, Maurizio Corbetta, Matthew F. Glasser, et al. 2013. “Function in the Human Connectome: Task-fMRI and Individual Differences in Behavior.” *NeuroImage* 80 (October): 169–89. <https://doi.org/10.1016/j.neuroimage.2013.05.033>.

Binder, Jeffrey R., William L. Gross, Jane B. Allendorfer, Leonardo Bonilha, Jessica Chapin, Jonathan C. Edwards, Thomas J. Grabowski, et al. 2011. “Mapping Anterior Temporal Lobe Language Areas with fMRI: A Multicenter Normative Study.” *NeuroImage* 54 (2): 1465–75. <https://doi.org/10.1016/j.neuroimage.2010.09.048>.

Castelli, Fulvia, Francesca Happé, Uta Frith, and Chris Frith. 2000. “Movement and Mind: A Functional Imaging Study of Perception and Interpretation of Complex Intentional Movement Patterns.” *NeuroImage* 12 (3): 314–25. <https://doi.org/10.1006/nimg.2000.0612>.

Delgado, M. R., L. E. Nystrom, C. Fissell, D. C. Noll, and J. A. Fiez. 2000. “Tracking the Hemodynamic Responses to Reward and Punishment in the Striatum.” *Journal of Neurophysiology* 84 (6): 3072–77. <https://doi.org/10.1152/jn.2000.84.6.3072>.

Downing, P. E., Y. Jiang, M. Shuman, and N. Kanwisher. 2001. “A cortical area selective for visual processing of the human body.” *Science (New York, N.Y.)* 293 (5539): 2470–73. <https://doi.org/10.1126/science.1063414>.

Hariri, Ahmad R., Alessandro Tessitore, Venkata S. Mattay, Francesco Fera, and Daniel R. Weinberger. 2002. “The Amygdala Response to Emotional Stimuli: A Comparison of Faces and Scenes.” *NeuroImage* 17 (1): 317–23. <https://doi.org/10.1006/nimg.2002.1179>.

Smith, Rachelle, Kamyar Keramatian, and Kalina Christoff. 2007. “Localizing the Rostrolateral Prefrontal Cortex at the Individual Level.” *NeuroImage* 36 (4): 1387–96. <https://doi.org/10.1016/j.neuroimage.2007.04.032>.

Wheatley, Thalia, Shawn C. Milleville, and Alex Martin. 2007. “Understanding Animate Agents: Distinct Roles for the Social Network and Mirror System.” *Psychological Science* 18 (6): 469–74. <https://doi.org/10.1111/j.1467-9280.2007.01923.x>.
