# Supplementary material for: Cerebellocerebral connectivity predicts body mass index: a new open-source Python-based framework for connectome-based predictive modeling
Source: Gigascience. 2025 Mar 12;14:giaf010. doi: 10.1093/gigascience/giaf010 (PMC11899596; doi:10.1093/gigascience/giaf010)
Supplement: giaf010_GIGA-D-24-00385_Revision_1 [file giaf010_giga-d-24-00385_revision_1.pdf]

GigaScience

Cerebellocerebral Connectivity Predicts Body Mass Index: a New Open-Source Python-based Framework for Connectome-based Predictive Modeling

--Manuscript Draft--

**Manuscript Number:**	GIGA-D-24-00385R1
**Full Title:**	Cerebellocerebral Connectivity Predicts Body Mass Index: a New Open-Source Python-based Framework for Connectome-based Predictive Modeling
**Article Type:**	Research
**Funding Information:**	
**Abstract:**	Background: The cerebellum is one of the major central-nervous structures consistently altered in obesity. Its role in higher-cognitive function, parts of which are affected by obesity, is mediated through projections to and from the cerebral cortex. We therefore investigated the relationship between body mass index (BMI) and cerebellocerebral connectivity. Methods: We utilized the Human Connectome Project's Young Adults dataset including functional MRI (fMRI) and behavioral data, to perform connectome-based predictive modeling (CPM) restricted to cerebellocerebral connectivity of resting-state fMRI and task-based fMRI. We developed a Python-based open-source framework to perform CPM, a data-driven technique with built-in cross validation to establish brain-behavior relationships. Significance was assessed with permutation analysis. Results: We found that 1. cerebellocerebral connectivity predicted BMI, 2. task-general cerebellocerebral connectivity predicted BMI more reliably than resting-state fMRI and individual task-based fMRI separetely, 3. predictive networks derived this way overlapped with established functional brain networks (namely frontoparietal networks, the somatomotor network, the salience network, and the default mode network), and 4. we found there was an inverse overlap between networks predictive of BMI and networks predictive of cognitive measures aversely affected by overweight/obesity. Conclusions: Our results suggest obesity-specific alterations in cerebellocerebral connectivity, specifically with regard to task execution. With brain areas and brain networks relevant to task performance implicated, these alterations seem to reflect a neurobiological substrate for task performance aversely affected by obesity.
**Corresponding Author:**	Tobias Bachmann<br>University Hospital Leipzig: Universitatsklinikum Leipzig<br>Leipzig, GERMANY
**Corresponding Author Secondary Information:**	
**Corresponding Author's Institution:**	University Hospital Leipzig: Universitatsklinikum Leipzig
**Corresponding Author's Secondary Institution:**	
**First Author:**	Tobias Bachmann
**First Author Secondary Information:**	
**Order of Authors:**	Tobias Bachmann
	Karsten Mueller
	Matthias Schroeter
	Paolo Piaggi
	Christopher Weise
**Order of Authors Secondary Information:**	
**Response to Reviewers:**	Dear editorial board of GigaScience,<br><br>thank you for giving us the opportunity to submit a revised version of our manuscript. Since we have included some figures in our response, we have addressed the

Powered by Editorial Manager® and ProduXion Manager® from Aries Systems Corporation

	reviewers' points in a separate file (see uploaded Response_to_Reviewers.pdf). In addition, as requested, we registered our software and workflow with bio.tools, workflowhub.eu and as an RRID. The relevant references and IDs have been included in the revised manuscript.<br><br>Best regards,<br>The authors
Additional Information:	
Question	Response
Are you submitting this manuscript to a special series or article collection?	No
**Experimental design and statistics**<br><br>Full details of the experimental design and statistical methods used should be given in the Methods section, as detailed in our Minimum Standards Reporting Checklist. Information essential to interpreting the data presented should be made available in the figure legends.<br><br>Have you included all the information requested in your manuscript?	Yes
**Resources**<br><br>A description of all resources used, including antibodies, cell lines, animals and software tools, with enough information to allow them to be uniquely identified, should be included in the Methods section. Authors are strongly encouraged to cite Research Resource Identifiers (RRIDs) for antibodies, model organisms and tools, where possible.<br><br>Have you included the information requested as detailed in our Minimum Standards Reporting Checklist?	Yes
**Availability of data and materials**<br><br>All datasets and code on which the conclusions of the paper rely must be either included in your submission or deposited in publicly available repositories	Yes

(where available and ethically appropriate), referencing such data using a unique identifier in the references and in the "Availability of Data and Materials" section of your manuscript.<br><br>Have you have met the above requirement as detailed in our Minimum Standards Reporting Checklist?	

Click here to access/download;LaTeX - Main Document (TeX file);main.tex

```
This is pdfTeX, Version 3.141592653-2.6-1.40.26 (TeX Live 2024)
(preloaded format=pdflatex 2024.8.2)  23 JAN 2025 03:01
entering extended mode
 restricted \write18 enabled.
 %&-line parsing enabled.
**main.tex
(./main.tex
LaTeX2e <2024-06-01> patch level 2
L3 programming layer <2024-05-27>
(./oup-contemporary.cls
Document Class: oup-contemporary 2023/06/12, v1.2
(c:/texlive/2024/texmf-dist/tex/latex/base/article.cls
Document Class: article 2024/02/08 v1.4n Standard LaTeX document class
(c:/texlive/2024/texmf-dist/tex/latex/base/size10.clo
File: size10.clo 2024/02/08 v1.4n Standard LaTeX file (size option)
)
\c@part=\count194
\c@section=\count195
\c@subsection=\count196
\c@subsubsection=\count197
\c@paragraph=\count198
\c@subparagraph=\count199
\c@figure=\count266
\c@table=\count267
\abovecaptionskip=\skip49
\belowcaptionskip=\skip50
\bibindent=\dimen141
) (c:/texlive/2024/texmf-dist/tex/latex/base/inputenc.sty
Package: inputenc 2024/02/08 v1.3d Input encoding file
\inpenc@prehook=\toks17
\inpenc@posthook=\toks18
) (c:/texlive/2024/texmf-dist/tex/latex/base/fontenc.sty
Package: fontenc 2021/04/29 v2.0v Standard LaTeX package
) (c:/texlive/2024/texmf-dist/tex/generic/iftex/ifpdf.sty
Package: ifpdf 2019/10/25 v3.4 ifpdf legacy package. Use iftex instead.
(c:/texlive/2024/texmf-dist/tex/generic/iftex/iftex.sty
Package: iftex 2022/02/03 v1.0f TeX engine tests
)) (c:/texlive/2024/texmf-dist/tex/latex/microtype/microtype.sty
Package: microtype 2024/03/29 v3.1b Micro-typographical refinements (RS)
(c:/texlive/2024/texmf-dist/tex/latex/graphics/keyval.sty
Package: keyval 2022/05/29 v1.15 key=value parser (DPC)
\KV@toks@=\toks19
) (c:/texlive/2024/texmf-dist/tex/latex/etoolbox/etoolbox.sty
Package: etoolbox 2020/10/05 v2.5k e-TeX tools for LaTeX (JAW)
\etb@tempcnta=\count268
)
\MT@toks=\toks20
\MT@tempbox=\box52
\MT@count=\count269
LaTeX Info: Redefining \noprotrusionifhmode on input line 1061.
LaTeX Info: Redefining \leftprotrusion on input line 1062.
\MT@prot@toks=\toks21
LaTeX Info: Redefining \rightprotrusion on input line 1081.
LaTeX Info: Redefining \textls on input line 1392.
```

```
\MT@outer@kern=\dimen142
LaTeX Info: Redefining \textmicrotypecontext on input line 2013.
\MT@listname@count=\count270
(c:/texlive/2024/texmf-dist/tex/latex/microtype/microtype-pdftex.def
File: microtype-pdftex.def 2024/03/29 v3.1b Definitions specific to
pdftex (RS)

LaTeX Info: Redefining \lsstyle on input line 902.
LaTeX Info: Redefining \lslig on input line 902.
\MT@outer@space=\skip51
)
Package microtype Info: Loading configuration file microtype.cfg.
(c:/texlive/2024/texmf-dist/tex/latex/microtype/microtype.cfg
File: microtype.cfg 2024/03/29 v3.1b microtype main configuration file
(RS)
)) (c:/texlive/2024/texmf-dist/tex/latex/euler/euler.sty
Package: euler 1995/03/05 v2.5
Package: `euler' v2.5 <1995/03/05> (FJ and FMi)
LaTeX Font Info:    Redeclaring symbol font `letters' on input line 35.
LaTeX Font Info:    Encoding `OML' has changed to `U' for symbol font
(Font)              `letters' in the math version `normal' on input line
35.
LaTeX Font Info:    Overwriting symbol font `letters' in version `normal'
(Font)                  OML/cmm/m/it --> U/eur/m/n on input line 35.
LaTeX Font Info:    Encoding `OML' has changed to `U' for symbol font
(Font)              `letters' in the math version `bold' on input line
35.
LaTeX Font Info:    Overwriting symbol font `letters' in version `bold'
(Font)                  OML/cmm/b/it --> U/eur/m/n on input line 35.
LaTeX Font Info:    Overwriting symbol font `letters' in version `bold'
(Font)                  U/eur/m/n --> U/eur/b/n on input line 36.
LaTeX Font Info:    Redeclaring math symbol \Gamma on input line 47.
LaTeX Font Info:    Redeclaring math symbol \Delta on input line 48.
LaTeX Font Info:    Redeclaring math symbol \Theta on input line 49.
LaTeX Font Info:    Redeclaring math symbol \Lambda on input line 50.
LaTeX Font Info:    Redeclaring math symbol \Xi on input line 51.
LaTeX Font Info:    Redeclaring math symbol \Pi on input line 52.
LaTeX Font Info:    Redeclaring math symbol \Sigma on input line 53.
LaTeX Font Info:    Redeclaring math symbol \Upsilon on input line 54.
LaTeX Font Info:    Redeclaring math symbol \Phi on input line 55.
LaTeX Font Info:    Redeclaring math symbol \Psi on input line 56.
LaTeX Font Info:    Redeclaring math symbol \Omega on input line 57.
\symEulerFraktur=\mathgroup4
LaTeX Font Info:    Overwriting symbol font `EulerFraktur' in version
`bold'
(Font)                  U/euf/m/n --> U/euf/b/n on input line 63.
LaTeX Info: Redefining \oldstylenums on input line 85.
\symEulerScript=\mathgroup5
LaTeX Font Info:    Overwriting symbol font `EulerScript' in version
`bold'
(Font)                  U/eus/m/n --> U/eus/b/n on input line 93.
LaTeX Font Info:    Redeclaring math symbol \aleph on input line 97.
LaTeX Font Info:    Redeclaring math symbol \Re on input line 98.
LaTeX Font Info:    Redeclaring math symbol \Im on input line 99.
```

```
LaTeX Font Info:    Redeclaring math delimiter \vert on input line 101.
LaTeX Font Info:    Redeclaring math delimiter \backslash on input line
103.
LaTeX Font Info:    Redeclaring math symbol \neg on input line 106.
LaTeX Font Info:    Redeclaring math symbol \wedge on input line 108.
LaTeX Font Info:    Redeclaring math symbol \vee on input line 110.
LaTeX Font Info:    Redeclaring math symbol \setminus on input line 112.
LaTeX Font Info:    Redeclaring math symbol \sim on input line 113.
LaTeX Font Info:    Redeclaring math symbol \mid on input line 114.
LaTeX Font Info:    Redeclaring math delimiter \arrowvert on input line
116.
LaTeX Font Info:    Redeclaring math symbol \mathsection on input line
117.
\symEulerExtension=\mathgroup6
LaTeX Font Info:    Redeclaring math symbol \coprod on input line 125.
LaTeX Font Info:    Redeclaring math symbol \prod on input line 125.
LaTeX Font Info:    Redeclaring math symbol \sum on input line 125.
LaTeX Font Info:    Redeclaring math symbol \intop on input line 130.
LaTeX Font Info:    Redeclaring math symbol \ointop on input line 131.
LaTeX Font Info:    Redeclaring math symbol \braceld on input line 132.
LaTeX Font Info:    Redeclaring math symbol \bracerd on input line 133.
LaTeX Font Info:    Redeclaring math symbol \bracelu on input line 134.
LaTeX Font Info:    Redeclaring math symbol \braceru on input line 135.
LaTeX Font Info:    Redeclaring math symbol \infty on input line 136.
LaTeX Font Info:    Redeclaring math symbol \nearrow on input line 153.
LaTeX Font Info:    Redeclaring math symbol \searrow on input line 154.
LaTeX Font Info:    Redeclaring math symbol \nwarrow on input line 155.
LaTeX Font Info:    Redeclaring math symbol \swarrow on input line 156.
LaTeX Font Info:    Redeclaring math symbol \Leftrightarrow on input line
157.
LaTeX Font Info:    Redeclaring math symbol \Leftarrow on input line 158.
LaTeX Font Info:    Redeclaring math symbol \Rightarrow on input line
159.
LaTeX Font Info:    Redeclaring math symbol \leftrightarrow on input line
160.
LaTeX Font Info:    Redeclaring math symbol \leftarrow on input line 161.
LaTeX Font Info:    Redeclaring math symbol \rightarrow on input line
163.
LaTeX Font Info:    Redeclaring math delimiter \uparrow on input line
166.
LaTeX Font Info:    Redeclaring math delimiter \downarrow on input line
168.
LaTeX Font Info:    Redeclaring math delimiter \updownarrow on input line
170.
LaTeX Font Info:    Redeclaring math delimiter \Uparrow on input line
172.
LaTeX Font Info:    Redeclaring math delimiter \Downarrow on input line
174.
LaTeX Font Info:    Redeclaring math delimiter \Updownarrow on input line
176.
LaTeX Font Info:    Redeclaring math symbol \leftharpoonup on input line
177.
LaTeX Font Info:    Redeclaring math symbol \leftharpoondown on input
line 178.
```

```
LaTeX Font Info:    Redeclaring math symbol \rightharpoonup on input line
179.
LaTeX Font Info:    Redeclaring math symbol \rightharpoondown on input
line 180
.
LaTeX Font Info:    Redeclaring math delimiter \lbrace on input line 182.
LaTeX Font Info:    Redeclaring math delimiter \rbrace on input line 184.
\symcmmigroup=\mathgroup7
LaTeX Font Info:    Overwriting symbol font `cmmigroup' in version `bold'
(Font)                  OML/cmm/m/it --> OML/cmm/b/it on input line 200.
LaTeX Font Info:    Redeclaring math accent \vec on input line 201.
LaTeX Font Info:    Redeclaring math symbol \triangleleft on input line
202.
LaTeX Font Info:    Redeclaring math symbol \triangleright on input line
203.
LaTeX Font Info:    Redeclaring math symbol \star on input line 204.
LaTeX Font Info:    Redeclaring math symbol \lhook on input line 205.
LaTeX Font Info:    Redeclaring math symbol \rhook on input line 206.
LaTeX Font Info:    Redeclaring math symbol \flat on input line 207.
LaTeX Font Info:    Redeclaring math symbol \natural on input line 208.
LaTeX Font Info:    Redeclaring math symbol \sharp on input line 209.
LaTeX Font Info:    Redeclaring math symbol \smile on input line 210.
LaTeX Font Info:    Redeclaring math symbol \frown on input line 211.
LaTeX Font Info:    Redeclaring math accent \grave on input line 245.
LaTeX Font Info:    Redeclaring math accent \acute on input line 246.
LaTeX Font Info:    Redeclaring math accent \tilde on input line 247.
LaTeX Font Info:    Redeclaring math accent \ddot on input line 248.
LaTeX Font Info:    Redeclaring math accent \check on input line 249.
LaTeX Font Info:    Redeclaring math accent \breve on input line 250.
LaTeX Font Info:    Redeclaring math accent \bar on input line 251.
LaTeX Font Info:    Redeclaring math accent \dot on input line 252.
LaTeX Font Info:    Redeclaring math accent \hat on input line 254.
) (c:/texlive/2024/texmf-dist/tex/latex/merriweather/merriweather.sty
Package: merriweather 2022/09/20 (Bob Tennent) Supports
Merriweather(Sans) font
s for all LaTeX engines.
(c:/texlive/2024/texmf-dist/tex/generic/iftex/ifxetex.sty
Package: ifxetex 2019/10/25 v0.7 ifxetex legacy package. Use iftex
instead.
) (c:/texlive/2024/texmf-dist/tex/generic/iftex/ifluatex.sty
Package: ifluatex 2019/10/25 v1.5 ifluatex legacy package. Use iftex
instead.
) (c:/texlive/2024/texmf-dist/tex/latex/base/textcomp.sty
Package: textcomp 2024/04/24 v2.1b Standard LaTeX package
) (c:/texlive/2024/texmf-dist/tex/latex/xkeyval/xkeyval.sty
Package: xkeyval 2022/06/16 v2.9 package option processing (HA)
(c:/texlive/2024/texmf-dist/tex/generic/xkeyval/xkeyval.tex
(c:/texlive/2024/te
xmf-dist/tex/generic/xkeyval/xkvutils.tex
\XKV@toks=\toks22
\XKV@tempa@toks=\toks23
)
\XKV@depth=\count271
```

```
File: xkeyval.tex 2014/12/03 v2.7a key=value parser (HA)
)) (c:/texlive/2024/texmf-dist/tex/latex/base/fontenc.sty
Package: fontenc 2021/04/29 v2.0v Standard LaTeX package
) (c:/texlive/2024/texmf-dist/tex/latex/fontaxes/fontaxes.sty
Package: fontaxes 2020/07/21 v1.0e Font selection axes
LaTeX Info: Redefining \upshape on input line 29.
LaTeX Info: Redefining \itshape on input line 31.
LaTeX Info: Redefining \slshape on input line 33.
LaTeX Info: Redefining \swshape on input line 35.
LaTeX Info: Redefining \scshape on input line 37.
LaTeX Info: Redefining \sscshape on input line 39.
LaTeX Info: Redefining \ulcshape on input line 41.
LaTeX Info: Redefining \textsw on input line 47.
LaTeX Info: Redefining \textssc on input line 48.
LaTeX Info: Redefining \textulc on input line 49.
)) (c:/texlive/2024/texmf-dist/tex/latex/mathastext/mathastext.sty
Package: mathastext 2024/07/27 v1.4b Use the text font in math mode (JFB)

Package mathastext Info: Starting the math mode configuration.
\mst@exists@muskip=\muskip17
\mst@forall@muskip=\muskip18
\mst@prime@muskip=\muskip19
\mst@do@nonletters=\toks24
\mst@undo@nonletters=\toks25
\mst@do@easynonletters=\toks26
\mst@undo@easynonletters=\toks27
\symmtoperatorfont=\mathgroup8
\symmtletterfont=\mathgroup9
(   mathastext:   ) ! and ?
(   mathastext:   ) punctuation: , . : ; and \colon
LaTeX Info: Redefining \relbar on input line 1201.
LaTeX Info: Redefining \rightarrowfill on input line 1202.
LaTeX Info: Redefining \leftarrowfill on input line 1205.
(   mathastext:   ) + and =
LaTeX Info: Redefining \Relbar on input line 1298.
(   mathastext:   ) adding = ; and + to \nfss@catcodes
(   mathastext:   ) parentheses ( ) [ ] and slash /
(   mathastext:   ) alldelims: < > \backslash \setminus | \vert \mid \{
\}
LaTeX Font Info:    Redeclaring math symbol \setminus on input line 1364.
LaTeX Info: Redefining \models on input line 1383.
(   mathastext:   ) \# \mathdollar \% \&
(   mathastext:   ) \imath and \jmath
LaTeX Font Info:    Overwriting math alphabet `\Mathnormalbold' in
version `nor
mal'
(Font)                  T1/Merriwthr-OsF/b/it --> T1/Merriwthr-OsF/b/it
on inpu
t line 2863.
LaTeX Font Info:    Overwriting math alphabet `\Mathnormalbold' in
version `bol
d'
(Font)                  T1/Merriwthr-OsF/b/it --> T1/Merriwthr-OsF/b/it
on inpu
```

```
t line 2863.
LaTeX Font Info:    Overwriting symbol font `mtletterfont' in version
`normal'
(Font)                  T1/Merriwthr-OsF/m/it --> T1/Merriwthr-OsF/m/it
on inpu
t line 2863.
LaTeX Font Info:    Overwriting symbol font `mtletterfont' in version
`bold'
(Font)                  T1/Merriwthr-OsF/m/it --> T1/Merriwthr-OsF/b/it
on inpu
t line 2863.
LaTeX Font Info:    Overwriting symbol font `mtoperatorfont' in version
`normal
'
(Font)                  T1/Merriwthr-OsF/m/n --> T1/Merriwthr-OsF/m/n on
input
line 2863.
LaTeX Font Info:    Overwriting symbol font `mtoperatorfont' in version
`bold'
(Font)                  T1/Merriwthr-OsF/m/n --> T1/Merriwthr-OsF/b/n on
input
line 2863.
LaTeX Font Info:    Overwriting math alphabet `\Mathbf' in version
`normal'
(Font)                  T1/Merriwthr-OsF/b/n --> T1/Merriwthr-OsF/b/n on
input
line 2863.
LaTeX Font Info:    Overwriting math alphabet `\Mathbf' in version `bold'
(Font)                  T1/Merriwthr-OsF/b/n --> T1/Merriwthr-OsF/b/n on
input
line 2863.
LaTeX Font Info:    Overwriting math alphabet `\Mathit' in version
`normal'
(Font)                  T1/Merriwthr-OsF/m/it --> T1/Merriwthr-OsF/m/it
on inpu
t line 2863.
LaTeX Font Info:    Overwriting math alphabet `\Mathit' in version `bold'
(Font)                  T1/Merriwthr-OsF/m/it --> T1/Merriwthr-OsF/b/it
on inpu
t line 2863.
LaTeX Font Info:    Overwriting math alphabet `\Mathsf' in version
`normal'
(Font)                  T1/MerriwthrSans-OsF/m/n --> T1/MerriwthrSans-
OsF/m/n o
n input line 2863.
LaTeX Font Info:    Overwriting math alphabet `\Mathsf' in version `bold'
(Font)                  T1/MerriwthrSans-OsF/m/n --> T1/MerriwthrSans-
OsF/b/n o
n input line 2863.
LaTeX Font Info:    Overwriting math alphabet `\Mathtt' in version
`normal'
(Font)                  T1/lmtt/m/n --> T1/lmtt/m/n on input line 2863.
LaTeX Font Info:    Overwriting math alphabet `\Mathtt' in version `bold'
(Font)                  T1/lmtt/m/n --> T1/lmtt/b/n on input line 2863.
```

```
(   mathastext:   ) Latin letters in the `normal', resp. `bold',
(   mathastext:   ) math versions are now set up to use the fonts
(   mathastext:   ) T1/Merriwthr-OsF/m/it, resp. T1/Merriwthr-OsF/b/it.
(   mathastext:   ) Other characters (digits, ...) and \log-like names
will be
(   mathastext:   ) typeset with the n shape.
(   mathastext:   ) \hbar
(   mathastext:   ) minus as endash
(   mathastext:   ) The italic option is in effect.
(   mathastext:   ) \HUGE has been (re)-defined.
(   mathastext:   ) mathastext has declared larger sizes for subscripts.
(   mathastext:   ) To keep LaTeX defaults, use option
`defaultmathsizes'.

Package mathastext Info: Loading is complete.  You can now use
\Mathastext to
(mathastext)             modify the normal and bold math versions.  Use
it
(mathastext)             with optional argument or use \MTDeclareVersion
to
(mathastext)             declare additional math versions.
) (c:/texlive/2024/texmf-dist/tex/latex/relsize/relsize.sty
Package: relsize 2013/03/29 ver 4.1
) (c:/texlive/2024/texmf-dist/tex/latex/ragged2e/ragged2e.sty
Package: ragged2e 2023/06/22 v3.6 ragged2e Package
\CenteringLeftskip=\skip52
\RaggedLeftLeftskip=\skip53
\RaggedRightLeftskip=\skip54
\CenteringRightskip=\skip55
\RaggedLeftRightskip=\skip56
\RaggedRightRightskip=\skip57
\CenteringParfillskip=\skip58
\RaggedLeftParfillskip=\skip59
\RaggedRightParfillskip=\skip60
\JustifyingParfillskip=\skip61
\CenteringParindent=\skip62
\RaggedLeftParindent=\skip63
\RaggedRightParindent=\skip64
\JustifyingParindent=\skip65
) (c:/texlive/2024/texmf-dist/tex/latex/xcolor/xcolor.sty
Package: xcolor 2023/11/15 v3.01 LaTeX color extensions (UK)
(c:/texlive/2024/texmf-dist/tex/latex/graphics-cfg/color.cfg
File: color.cfg 2016/01/02 v1.6 sample color configuration
)
Package xcolor Info: Driver file: pdftex.def on input line 274.
(c:/texlive/2024/texmf-dist/tex/latex/graphics-def/pdftex.def
File: pdftex.def 2024/04/13 v1.2c Graphics/color driver for pdftex
) (c:/texlive/2024/texmf-dist/tex/latex/graphics/mathcolor.ltx)
Package xcolor Info: Model `cmy' substituted by `cmy0' on input line
1350.
Package xcolor Info: Model `hsb' substituted by `rgb' on input line 1354.
Package xcolor Info: Model `RGB' extended on input line 1366.
Package xcolor Info: Model `HTML' substituted by `rgb' on input line
1368.
```

```
Package xcolor Info: Model `Hsb' substituted by `hsb' on input line 1369.
Package xcolor Info: Model `tHsb' substituted by `hsb' on input line
1370.
Package xcolor Info: Model `HSB' substituted by `hsb' on input line 1371.
Package xcolor Info: Model `Gray' substituted by `gray' on input line
1372.
Package xcolor Info: Model `wave' substituted by `hsb' on input line
1373.
) (c:/texlive/2024/texmf-dist/tex/latex/colortbl/colortbl.sty
Package: colortbl 2024/07/06 v1.0i Color table columns (DPC)
(c:/texlive/2024/texmf-dist/tex/latex/tools/array.sty
Package: array 2024/06/14 v2.6d Tabular extension package (FMi)
\col@sep=\dimen143
\ar@mcellbox=\box53
\extrarowheight=\dimen144
\NC@list=\toks28
\extratabsurround=\skip66
\backup@length=\skip67
\ar@cellbox=\box54
)
\everycr=\toks29
\minrowclearance=\skip68
\rownum=\count272
) (c:/texlive/2024/texmf-dist/tex/latex/graphics/graphicx.sty
Package: graphicx 2021/09/16 v1.2d Enhanced LaTeX Graphics (DPC,SPQR)
(c:/texlive/2024/texmf-dist/tex/latex/graphics/graphics.sty
Package: graphics 2024/05/23 v1.4g Standard LaTeX Graphics (DPC,SPQR)
(c:/texlive/2024/texmf-dist/tex/latex/graphics/trig.sty
Package: trig 2023/12/02 v1.11 sin cos tan (DPC)
) (c:/texlive/2024/texmf-dist/tex/latex/graphics-cfg/graphics.cfg
File: graphics.cfg 2016/06/04 v1.11 sample graphics configuration
)
Package graphics Info: Driver file: pdftex.def on input line 106.
)
\Gin@req@height=\dimen145
\Gin@req@width=\dimen146
) (c:/texlive/2024/texmf-dist/tex/latex/xpatch/xpatch.sty
(c:/texlive/2024/texm
f-dist/tex/latex/l3kernel/expl3.sty
Package: expl3 2024-05-27 L3 programming layer (loader)
(c:/texlive/2024/texmf-dist/tex/latex/l3backend/l3backend-pdftex.def
File: l3backend-pdftex.def 2024-05-08 L3 backend support: PDF output
(pdfTeX)
\l__color_backend_stack_int=\count273
\l__pdf_internal_box=\box55
))
Package: xpatch 2020/03/25 v0.3a Extending etoolbox patching commands
(c:/texlive/2024/texmf-dist/tex/latex/l3packages/xparse/xparse.sty
Package: xparse 2024-05-08 L3 Experimental document command parser
)) (c:/texlive/2024/texmf-dist/tex/latex/environ/environ.sty
Package: environ 2014/05/04 v0.3 A new way to define environments
(c:/texlive/2024/texmf-dist/tex/latex/trimspaces/trimspaces.sty
Package: trimspaces 2009/09/17 v1.1 Trim spaces around a token list
)
```

```
\@envbody=\toks30
) (c:/texlive/2024/texmf-dist/tex/latex/lastpage/lastpage.sty
Package: lastpage 2024/07/07 v2.1c lastpage: 2.09 or 2e? (HMM)
(c:/texlive/2024/texmf-dist/tex/latex/lastpage/lastpage2e.sty
Package: lastpage2e 2024/07/07 v2.1c Decide which 2e lastpage version to
use (H
MM)
(c:/texlive/2024/texmf-dist/tex/latex/lastpage/lastpagemodern.sty
Package: lastpagemodern 2024-07-07 v2.1c Refers to last page's name (HMM;
JPG)
\c@lastpagecount=\count274
)
)) (c:/texlive/2024/texmf-dist/tex/latex/graphics/rotating.sty
Package: rotating 2016/08/11 v2.16d rotated objects in LaTeX
(c:/texlive/2024/texmf-dist/tex/latex/base/ifthen.sty
Package: ifthen 2024/03/16 v1.1e Standard LaTeX ifthen package (DPC)
)
\c@r@tfl@t=\count275
\rotFPtop=\skip69
\rotFPbot=\skip70
\rot@float@box=\box56
\rot@mess@toks=\toks31
) (c:/texlive/2024/texmf-dist/tex/latex/graphics/lscape.sty
Package: lscape 2020/05/28 v3.02 Landscape Pages (DPC)
) (c:/texlive/2024/texmf-dist/tex/latex/tools/afterpage.sty
Package: afterpage 2023/07/04 v1.08 After-Page Package (DPC)
\AP@output=\toks32
\AP@partial=\box57
\AP@footins=\box58
) (c:/texlive/2024/texmf-dist/tex/latex/textpos/textpos.sty
Package: textpos 2022/07/23 v1.10.1
Package textpos Info: choosing support for LaTeX3 on input line 60.
\TP@textbox=\box59
\TP@holdbox=\box60
\TPHorizModule=\dimen147
\TPVertModule=\dimen148
\TP@margin=\dimen149
\TP@absmargin=\dimen150
Grid set 16 x 16 = 37.34424pt x 52.81541pt
\TPboxrulesize=\dimen151
\TP@ox=\dimen152
\TP@oy=\dimen153
\TP@tbargs=\toks33
TextBlockOrigin set to 0pt x 0pt
) (c:/texlive/2024/texmf-dist/tex/latex/url/url.sty
\Urlmuskip=\muskip20
Package: url 2013/09/16  ver 3.4  Verb mode for urls, etc.
) (c:/texlive/2024/texmf-dist/tex/latex/newfloat/newfloat.sty
Package: newfloat 2023/10/01 v1.2 Defining new floating environments (AR)
Package newfloat Info: `rotating' package detected.
) (c:/texlive/2024/texmf-dist/tex/latex/mdframed/mdframed.sty
Package: mdframed 2013/07/01 1.9b: mdframed
(c:/texlive/2024/texmf-dist/tex/latex/kvoptions/kvoptions.sty
```

```
Package: kvoptions 2022-06-15 v3.15 Key value format for package options
(HO)
(c:/texlive/2024/texmf-dist/tex/generic/ltxcmds/ltxcmds.sty
Package: ltxcmds 2023-12-04 v1.26 LaTeX kernel commands for general use
(HO)
) (c:/texlive/2024/texmf-dist/tex/latex/kvsetkeys/kvsetkeys.sty
Package: kvsetkeys 2022-10-05 v1.19 Key value parser (HO)
)) (c:/texlive/2024/texmf-dist/tex/latex/zref/zref-abspage.sty
Package: zref-abspage 2023-09-14 v2.35 Module abspage for zref (HO)
(c:/texlive/2024/texmf-dist/tex/latex/zref/zref-base.sty
Package: zref-base 2023-09-14 v2.35 Module base for zref (HO)
(c:/texlive/2024/texmf-dist/tex/generic/infwarerr/infwarerr.sty
Package: infwarerr 2019/12/03 v1.5 Providing info/warning/error messages
(HO)
) (c:/texlive/2024/texmf-dist/tex/generic/kvdefinekeys/kvdefinekeys.sty
Package: kvdefinekeys 2019-12-19 v1.6 Define keys (HO)
) (c:/texlive/2024/texmf-dist/tex/generic/pdftexcmds/pdftexcmds.sty
Package: pdftexcmds 2020-06-27 v0.33 Utility functions of pdfTeX for
LuaTeX (HO
)
Package pdftexcmds Info: \pdf@primitive is available.
Package pdftexcmds Info: \pdf@ifprimitive is available.
Package pdftexcmds Info: \pdfdraftmode found.
) (c:/texlive/2024/texmf-dist/tex/generic/etexcmds/etexcmds.sty
Package: etexcmds 2019/12/15 v1.7 Avoid name clashes with e-TeX commands
(HO)
) (c:/texlive/2024/texmf-dist/tex/latex/auxhook/auxhook.sty
Package: auxhook 2019-12-17 v1.6 Hooks for auxiliary files (HO)
)
Package zref Info: New property list: main on input line 767.
Package zref Info: New property: default on input line 768.
Package zref Info: New property: page on input line 769.
)
\c@abspage=\count276
Package zref Info: New property: abspage on input line 67.
) (c:/texlive/2024/texmf-dist/tex/latex/needspace/needspace.sty
Package: needspace 2010/09/12 v1.3d reserve vertical space
)
\mdf@templength=\skip71
\c@mdf@globalstyle@cnt=\count277
\mdf@skipabove@length=\skip72
\mdf@skipbelow@length=\skip73
\mdf@leftmargin@length=\skip74
\mdf@rightmargin@length=\skip75
\mdf@innerleftmargin@length=\skip76
\mdf@innerrightmargin@length=\skip77
\mdf@innertopmargin@length=\skip78
\mdf@innerbottommargin@length=\skip79
\mdf@splittopskip@length=\skip80
\mdf@splitbottomskip@length=\skip81
\mdf@outermargin@length=\skip82
\mdf@innermargin@length=\skip83
\mdf@linewidth@length=\skip84
\mdf@innerlinewidth@length=\skip85
```

```
\mdf@middlelinewidth@length=\skip86
\mdf@outerlinewidth@length=\skip87
\mdf@roundcorner@length=\skip88
\mdf@footenotedistance@length=\skip89
\mdf@userdefinedwidth@length=\skip90
\mdf@needspace@length=\skip91
\mdf@frametitleaboveskip@length=\skip92
\mdf@frametitlebelowskip@length=\skip93
\mdf@frametitlerulewidth@length=\skip94
\mdf@frametitleleftmargin@length=\skip95
\mdf@frametitlerightmargin@length=\skip96
\mdf@shadowsize@length=\skip97
\mdf@extratopheight@length=\skip98
\mdf@subtitleabovelinewidth@length=\skip99
\mdf@subtitlebelowlinewidth@length=\skip100
\mdf@subtitleaboveskip@length=\skip101
\mdf@subtitlebelowskip@length=\skip102
\mdf@subtitleinneraboveskip@length=\skip103
\mdf@subtitleinnerbelowskip@length=\skip104
\mdf@subsubtitleabovelinewidth@length=\skip105
\mdf@subsubtitlebelowlinewidth@length=\skip106
\mdf@subsubtitleaboveskip@length=\skip107
\mdf@subsubtitlebelowskip@length=\skip108
\mdf@subsubtitleinneraboveskip@length=\skip109
\mdf@subsubtitleinnerbelowskip@length=\skip110
(c:/texlive/2024/texmf-dist/tex/latex/mdframed/md-frame-0.mdf
File: md-frame-0.mdf 2013/07/01\ 1.9b: md-frame-0
)
\mdf@frametitlebox=\box61
\mdf@footnotebox=\box62
\mdf@splitbox@one=\box63
\mdf@splitbox@two=\box64
\mdf@splitbox@save=\box65
\mdfsplitboxwidth=\skip111
\mdfsplitboxtotalwidth=\skip112
\mdfsplitboxheight=\skip113
\mdfsplitboxdepth=\skip114
\mdfsplitboxtotalheight=\skip115
\mdfframetitleboxwidth=\skip116
\mdfframetitleboxtotalwidth=\skip117
\mdfframetitleboxheight=\skip118
\mdfframetitleboxdepth=\skip119
\mdfframetitleboxtotalheight=\skip120
\mdffootnoteboxwidth=\skip121
\mdffootnoteboxtotalwidth=\skip122
\mdffootnoteboxheight=\skip123
\mdffootnoteboxdepth=\skip124
\mdffootnoteboxtotalheight=\skip125
\mdftotallinewidth=\skip126
\mdfboundingboxwidth=\skip127
\mdfboundingboxtotalwidth=\skip128
\mdfboundingboxheight=\skip129
\mdfboundingboxdepth=\skip130
\mdfboundingboxtotalheight=\skip131
```

```
\mdf@freevspace@length=\skip132
\mdf@horizontalwidthofbox@length=\skip133
\mdf@verticalmarginwhole@length=\skip134
\mdf@horizontalspaceofbox=\skip135
\mdfsubtitleheight=\skip136
\mdfsubsubtitleheight=\skip137
\c@mdfcountframes=\count278

****** mdframed patching \endmdf@trivlist

****** -- success******

\mdf@envdepth=\count279
\c@mdf@env@i=\count280
\c@mdf@env@ii=\count281
\c@mdf@zref@counter=\count282
Package zref Info: New property: mdf@pagevalue on input line 895.
) (c:/texlive/2024/texmf-dist/tex/latex/titlesec/titlesec.sty
Package: titlesec 2023/10/27 v2.16 Sectioning titles
\ttl@box=\box66
\beforetitleunit=\skip138
\aftertitleunit=\skip139
\ttl@plus=\dimen154
\ttl@minus=\dimen155
\ttl@toksa=\toks34
\titlewidth=\dimen156
\titlewidthlast=\dimen157
\titlewidthfirst=\dimen158
) (c:/texlive/2024/texmf-dist/tex/latex/koma-script/scrextend.sty
Package: scrextend 2023/07/07 v3.41 KOMA-Script package (extend other
classes w
ith features of KOMA-Script classes)
(c:/texlive/2024/texmf-dist/tex/latex/koma-script/scrkbase.sty
Package: scrkbase 2023/07/07 v3.41 KOMA-Script package (KOMA-Script-
dependent b
asics and keyval usage)
(c:/texlive/2024/texmf-dist/tex/latex/koma-script/scrbase.sty
Package: scrbase 2023/07/07 v3.41 KOMA-Script package (KOMA-Script-
independent
basics and keyval usage)
(c:/texlive/2024/texmf-dist/tex/latex/koma-script/scrlfile.sty
Package: scrlfile 2023/07/07 v3.41 KOMA-Script package (file load hooks)
(c:/texlive/2024/texmf-dist/tex/latex/koma-script/scrlfile-hook.sty
Package: scrlfile-hook 2023/07/07 v3.41 KOMA-Script package (using LaTeX
hooks)

(c:/texlive/2024/texmf-dist/tex/latex/koma-script/scrlogo.sty
Package: scrlogo 2023/07/07 v3.41 KOMA-Script package (logo)
)))
Applying: [2021/05/01] Usage of raw or classic option list on input line
252.
Already applied: [0000/00/00] Usage of raw or classic option list on
input line
 368.
```

```
))
Package scrextend Info: unexpected definition of `\@makefnmark'.
(scrextend)             Trying to patch it on input line 1762.
Package scrextend Info: patch seems to be successfull on input line 1762.
)

LaTeX Font Warning: Font shape `T1/cmr/m/n' in size <7.5> not available
(Font)              size <7> substituted on input line 69.

(c:/texlive/2024/texmf-dist/tex/latex/tools/calc.sty
Package: calc 2023/07/08 v4.3 Infix arithmetic (KKT,FJ)
\calc@Acount=\count283
\calc@Bcount=\count284
\calc@Adimen=\dimen159
\calc@Bdimen=\dimen160
\calc@Askip=\skip140
\calc@Bskip=\skip141
LaTeX Info: Redefining \setlength on input line 80.
LaTeX Info: Redefining \addtolength on input line 81.
\calc@Ccount=\count285
\calc@Cskip=\skip142
) (c:/texlive/2024/texmf-dist/tex/latex/geometry/geometry.sty
Package: geometry 2020/01/02 v5.9 Page Geometry
(c:/texlive/2024/texmf-dist/tex/generic/iftex/ifvtex.sty
Package: ifvtex 2019/10/25 v1.7 ifvtex legacy package. Use iftex instead.
)
\Gm@cnth=\count286
\Gm@cntv=\count287
\c@Gm@tempcnt=\count288
\Gm@bindingoffset=\dimen161
\Gm@wd@mp=\dimen162
\Gm@odd@mp=\dimen163
\Gm@even@mp=\dimen164
\Gm@layoutwidth=\dimen165
\Gm@layoutheight=\dimen166
\Gm@layouthoffset=\dimen167
\Gm@layoutvoffset=\dimen168
\Gm@dimlist=\toks35
) (c:/texlive/2024/texmf-dist/tex/latex/preprint/authblk.sty
Package: authblk 2001/02/27 1.3 (PWD)
\affilsep=\skip143
\@affilsep=\skip144
\c@Maxaffil=\count289
\c@authors=\count290
\c@affil=\count291
) (c:/texlive/2024/texmf-dist/tex/latex/footmisc/footmisc.sty
Package: footmisc 2023/07/05 v6.0f a miscellany of footnote facilities
\FN@temptoken=\toks36
\footnotemargin=\dimen169
\@outputbox@depth=\dimen170
Package footmisc Info: Declaring symbol style bringhurst on input line
696.
Package footmisc Info: Declaring symbol style chicago on input line 704.
Package footmisc Info: Declaring symbol style wiley on input line 713.
```

```
Package footmisc Info: Declaring symbol style lamport-robust on input
line 724.

Package footmisc Info: Declaring symbol style lamport* on input line 744.
Package footmisc Info: Declaring symbol style lamport*-robust on input
line 765
.
) (c:/texlive/2024/texmf-dist/tex/latex/fancyhdr/fancyhdr.sty
Package: fancyhdr 2024/07/23 v4.3.1 Extensive control of page headers and
foote
rs
\f@nch@headwidth=\skip145
\f@nch@O@elh=\skip146
\f@nch@O@erh=\skip147
\f@nch@O@olh=\skip148
\f@nch@O@orh=\skip149
\f@nch@O@elf=\skip150
\f@nch@O@erf=\skip151
\f@nch@O@olf=\skip152
\f@nch@O@orf=\skip153
) (c:/texlive/2024/texmf-dist/tex/generic/alphalph/alphalph.sty
Package: alphalph 2019/12/09 v2.6 Convert numbers to letters (HO)
(c:/texlive/2024/texmf-dist/tex/generic/intcalc/intcalc.sty
Package: intcalc 2019/12/15 v1.3 Expandable calculations with integers
(HO)
))
\c@authorfn=\count292
(c:/texlive/2024/texmf-dist/tex/latex/abstract/abstract.sty
Package: abstract 2009/06/08 v1.2a configurable abstracts
\abstitleskip=\skip154
\absleftindent=\skip155
\absrightindent=\skip156
\absparindent=\skip157
\absparsep=\skip158
)
Package newfloat Info: New float `keypoints' with options
`placement=t!,name=kp
t' on input line 291.
\c@keypoints=\count293
\newfloat@ftype=\count294
Package newfloat Info: float type `keypoints'=8 on input line 291.
(c:/texlive/2024/texmf-dist/tex/latex/enumitem/enumitem.sty
Package: enumitem 2019/06/20 v3.9 Customized lists
\labelindent=\skip159
\enit@outerparindent=\dimen171
\enit@toks=\toks37
\enit@inbox=\box67
\enit@count@id=\count295
\enitdp@description=\count296
) (c:/texlive/2024/texmf-dist/tex/latex/quoting/quoting.sty
Package: quoting 2014/01/28 v0.1c Consolidated environment for displayed
text
\quo@toppartop=\skip160
) (c:/texlive/2024/texmf-dist/tex/latex/sttools/stfloats.sty
```

```
Package: stfloats 2017/03/27 v3.3 Improve float mechanism and
baselineskip sett
ings
\@dblbotnum=\count297
\c@dblbotnumber=\count298
) (c:/texlive/2024/texmf-dist/tex/latex/booktabs/booktabs.sty
Package: booktabs 2020/01/12 v1.61803398 Publication quality tables
\heavyrulewidth=\dimen172
\lightrulewidth=\dimen173
\cmidrulewidth=\dimen174
\belowrulesep=\dimen175
\belowbottomsep=\dimen176
\aboverulesep=\dimen177
\abovetopsep=\dimen178
\cmidrulesep=\dimen179
\cmidrulekern=\dimen180
\defaultaddspace=\dimen181
\@cmidla=\count299
\@cmidlb=\count300
\@aboverulesep=\dimen182
\@belowrulesep=\dimen183
\@thisruleclass=\count301
\@lastruleclass=\count302
\@thisrulewidth=\dimen184
) (c:/texlive/2024/texmf-dist/tex/latex/tools/tabularx.sty
Package: tabularx 2023/12/11 v2.12a `tabularx' package (DPC)
\TX@col@width=\dimen185
\TX@old@table=\dimen186
\TX@old@col=\dimen187
\TX@target=\dimen188
\TX@delta=\dimen189
\TX@cols=\count303
\TX@ftn=\toks38
)
\enitdp@tablenotes=\count304
(c:/texlive/2024/texmf-dist/tex/latex/caption/caption.sty
Package: caption 2023/08/05 v3.6o Customizing captions (AR)
(c:/texlive/2024/texmf-dist/tex/latex/caption/caption3.sty
Package: caption3 2023/07/31 v2.4d caption3 kernel (AR)
\caption@tempdima=\dimen190
\captionmargin=\dimen191
\caption@leftmargin=\dimen192
\caption@rightmargin=\dimen193
\caption@width=\dimen194
\caption@indent=\dimen195
\caption@parindent=\dimen196
\caption@hangindent=\dimen197
Package caption Info: Standard document class detected.
)
\c@caption@flags=\count305
\c@continuedfloat=\count306
Package caption Info: rotating package is loaded.
Package caption Info: scrextend package is loaded.
\caption@addmargin@hsize=\dimen198
```

```
\caption@addmargin@linewidth=\dimen199
) (c:/texlive/2024/texmf-dist/tex/latex/natbib/natbib.sty
Package: natbib 2010/09/13 8.31b (PWD, AO)
\bibhang=\skip161
\bibsep=\skip162
LaTeX Info: Redefining \cite on input line 694.
\c@NAT@ctr=\count307
)) (c:/texlive/2024/texmf-dist/tex/latex/siunitx/siunitx.sty
Package: siunitx 2024-06-24 v3.3.19 A comprehensive (SI) units package
\l__siunitx_number_uncert_offset_int=\count308
\l__siunitx_number_exponent_fixed_int=\count309
\l__siunitx_number_min_decimal_int=\count310
\l__siunitx_number_min_integer_int=\count311
\l__siunitx_number_round_precision_int=\count312
\l__siunitx_number_lower_threshold_int=\count313
\l__siunitx_number_upper_threshold_int=\count314
\l__siunitx_number_group_first_int=\count315
\l__siunitx_number_group_size_int=\count316
\l__siunitx_number_group_minimum_int=\count317
\l__siunitx_angle_tmp_dim=\dimen256
\l__siunitx_angle_marker_box=\box68
\l__siunitx_angle_unit_box=\box69
\l__siunitx_compound_count_int=\count318
(c:/texlive/2024/texmf-dist/tex/latex/translations/translations.sty
Package: translations 2022/02/05 v1.12 internationalization of LaTeX2e
packages
 (CN)
) (c:/texlive/2024/texmf-dist/tex/latex/amsmath/amstext.sty
Package: amstext 2021/08/26 v2.01 AMS text
(c:/texlive/2024/texmf-dist/tex/latex/amsmath/amsgen.sty
File: amsgen.sty 1999/11/30 v2.0 generic functions
\@emptytoks=\toks39
\ex@=\dimen257
))
\l__siunitx_table_tmp_box=\box70
\l__siunitx_table_tmp_dim=\dimen258
\l__siunitx_table_column_width_dim=\dimen259
\l__siunitx_table_integer_box=\box71
\l__siunitx_table_decimal_box=\box72
\l__siunitx_table_uncert_box=\box73
\l__siunitx_table_before_box=\box74
\l__siunitx_table_after_box=\box75
\l__siunitx_table_before_dim=\dimen260
\l__siunitx_table_carry_dim=\dimen261
\l__siunitx_unit_tmp_int=\count319
\l__siunitx_unit_position_int=\count320
\l__siunitx_unit_total_int=\count321
) (c:/texlive/2024/texmf-dist/tex/latex/tools/longtable.sty
Package: longtable 2024-04-26 v4.20 Multi-page Table package (DPC)
\LTleft=\skip163
\LTright=\skip164
\LTpre=\skip165
\LTpost=\skip166
\LTchunksize=\count322
```

```
\LTcapwidth=\dimen262
\LT@head=\box76
\LT@firsthead=\box77
\LT@foot=\box78
\LT@lastfoot=\box79
\LT@gbox=\box80
\LT@cols=\count323
\LT@rows=\count324
\c@LT@tables=\count325
\c@LT@chunks=\count326
\LT@p@ftn=\toks40
) (c:/texlive/2024/texmf-dist/tex/latex/hyperref/hyperref.sty
Package: hyperref 2024-07-10 v7.01j Hypertext links for LaTeX
(c:/texlive/2024/texmf-dist/tex/generic/pdfescape/pdfescape.sty
Package: pdfescape 2019/12/09 v1.15 Implements pdfTeX's escape features
(HO)
) (c:/texlive/2024/texmf-dist/tex/latex/hycolor/hycolor.sty
Package: hycolor 2020-01-27 v1.10 Color options for hyperref/bookmark
(HO)
) (c:/texlive/2024/texmf-dist/tex/latex/hyperref/nameref.sty
Package: nameref 2023-11-26 v2.56 Cross-referencing by name of section
(c:/texlive/2024/texmf-dist/tex/latex/refcount/refcount.sty
Package: refcount 2019/12/15 v3.6 Data extraction from label references
(HO)
) (c:/texlive/2024/texmf-
dist/tex/generic/gettitlestring/gettitlestring.sty
Package: gettitlestring 2019/12/15 v1.6 Cleanup title references (HO)
)
\c@section@level=\count327
) (c:/texlive/2024/texmf-dist/tex/generic/stringenc/stringenc.sty
Package: stringenc 2019/11/29 v1.12 Convert strings between diff.
encodings (HO
)
)
\@linkdim=\dimen263
\Hy@linkcounter=\count328
\Hy@pagecounter=\count329
(c:/texlive/2024/texmf-dist/tex/latex/hyperref/pd1enc.def
File: pd1enc.def 2024-07-10 v7.01j Hyperref: PDFDocEncoding definition
(HO)
Now handling font encoding PD1 ...
... no UTF-8 mapping file for font encoding PD1
)
\Hy@SavedSpaceFactor=\count330
(c:/texlive/2024/texmf-dist/tex/latex/hyperref/puenc.def
File: puenc.def 2024-07-10 v7.01j Hyperref: PDF Unicode definition (HO)
Now handling font encoding PU ...
... no UTF-8 mapping file for font encoding PU
)
Package hyperref Info: Option `colorlinks' set `true' on input line 4040.
Package hyperref Info: Hyper figures OFF on input line 4157.
Package hyperref Info: Link nesting OFF on input line 4162.
Package hyperref Info: Hyper index ON on input line 4165.
Package hyperref Info: Plain pages OFF on input line 4172.
```

```
Package hyperref Info: Backreferencing OFF on input line 4177.
Package hyperref Info: Implicit mode ON; LaTeX internals redefined.
Package hyperref Info: Bookmarks ON on input line 4424.
\c@Hy@tempcnt=\count331
LaTeX Info: Redefining \url on input line 4763.
\XeTeXLinkMargin=\dimen264
(c:/texlive/2024/texmf-dist/tex/generic/bitset/bitset.sty
Package: bitset 2019/12/09 v1.3 Handle bit-vector datatype (HO)
(c:/texlive/2024/texmf-dist/tex/generic/bigintcalc/bigintcalc.sty
Package: bigintcalc 2019/12/15 v1.5 Expandable calculations on big
integers (HO
)
))
\Fld@menulength=\count332
\Field@Width=\dimen265
\Fld@charsize=\dimen266
Package hyperref Info: Hyper figures OFF on input line 6042.
Package hyperref Info: Link nesting OFF on input line 6047.
Package hyperref Info: Hyper index ON on input line 6050.
Package hyperref Info: backreferencing OFF on input line 6057.
Package hyperref Info: Link coloring ON on input line 6060.
Package hyperref Info: Link coloring with OCG OFF on input line 6067.
Package hyperref Info: PDF/A mode OFF on input line 6072.
(c:/texlive/2024/texmf-dist/tex/latex/base/atbegshi-ltx.sty
Package: atbegshi-ltx 2021/01/10 v1.0c Emulation of the original atbegshi
package with kernel methods
)
\Hy@abspage=\count333
\c@Item=\count334
\c@Hfootnote=\count335
)
Package hyperref Info: Driver (autodetected): hpdftex.
(c:/texlive/2024/texmf-dist/tex/latex/hyperref/hpdftex.def
File: hpdftex.def 2024-07-10 v7.01j Hyperref driver for pdfTeX
(c:/texlive/2024/texmf-dist/tex/latex/base/atveryend-ltx.sty
Package: atveryend-ltx 2020/08/19 v1.0a Emulation of the original
atveryend pac
kage
with kernel methods
)
\HyAnn@Count=\count336
\Fld@listcount=\count337
\c@bookmark@seq@number=\count338
(c:/texlive/2024/texmf-dist/tex/latex/rerunfilecheck/rerunfilecheck.sty
Package: rerunfilecheck 2022-07-10 v1.10 Rerun checks for auxiliary files
(HO)
(c:/texlive/2024/texmf-dist/tex/generic/uniquecounter/uniquecounter.sty
Package: uniquecounter 2019/12/15 v1.4 Provide unlimited unique counter
(HO)
)
Package uniquecounter Info: New unique counter `rerunfilecheck' on input
line 2
85.
)
```

```
\Hy@SectionHShift=\skip167
)
Package translations Info: No language package found. I am going to use
`englis
h' as default language. on input line 59.
LaTeX Font Info:    Trying to load font information for T1+Merriwthr-OsF
on inp
ut line 59.
(c:/texlive/2024/texmf-dist/tex/latex/merriweather/T1Merriwthr-OsF.fd
File: T1Merriwthr-OsF.fd 2020/08/30 (autoinst) Font definitions for
T1/Merriwth
r-OsF.
)
LaTeX Font Info:    Font shape `T1/Merriwthr-OsF/m/n' will be
(Font)              scaled to size 7.5pt on input line 59.
(./main.aux)
\openout1 = `main.aux'.

LaTeX Font Info:    Checking defaults for OML/cmm/m/it on input line 59.
LaTeX Font Info:    ... okay on input line 59.
LaTeX Font Info:    Checking defaults for OMS/cmsy/m/n on input line 59.
LaTeX Font Info:    ... okay on input line 59.
LaTeX Font Info:    Checking defaults for OT1/cmr/m/n on input line 59.
LaTeX Font Info:    ... okay on input line 59.
LaTeX Font Info:    Checking defaults for T1/cmr/m/n on input line 59.
LaTeX Font Info:    ... okay on input line 59.
LaTeX Font Info:    Checking defaults for TS1/cmr/m/n on input line 59.
LaTeX Font Info:    ... okay on input line 59.
LaTeX Font Info:    Checking defaults for OMX/cmex/m/n on input line 59.
LaTeX Font Info:    ... okay on input line 59.
LaTeX Font Info:    Checking defaults for U/cmr/m/n on input line 59.
LaTeX Font Info:    ... okay on input line 59.
LaTeX Font Info:    Checking defaults for PD1/pdf/m/n on input line 59.
LaTeX Font Info:    ... okay on input line 59.
LaTeX Font Info:    Checking defaults for PU/pdf/m/n on input line 59.
LaTeX Font Info:    ... okay on input line 59.
LaTeX Info: Redefining \microtypecontext on input line 59.
Package microtype Info: Applying patch `item' on input line 59.
Package microtype Info: Applying patch `toc' on input line 59.
Package microtype Info: Applying patch `eqnum' on input line 59.
Package microtype Info: Applying patch `footnote' on input line 59.
Package microtype Info: Applying patch `verbatim' on input line 59.
Package microtype Info: Generating PDF output.
Package microtype Info: Character protrusion enabled (level 2).
Package microtype Info: Using default protrusion set `alltext'.
Package microtype Info: Automatic font expansion enabled (level 2),
(microtype)             stretch: 20, shrink: 20, step: 1, non-selected.
Package microtype Info: Using default expansion set `alltext-nott'.
LaTeX Info: Redefining \showhyphens on input line 59.
Package microtype Info: No adjustment of tracking.
Package microtype Info: No adjustment of interword spacing.
Package microtype Info: No adjustment of character kerning.
Package microtype Info: Loading generic protrusion settings for font
family
```

```
(microtype)             `Merriwthr-OsF' (encoding: T1).
(microtype)             For optimal results, create family-specific
settings.
(microtype)             See the microtype manual for details.
LaTeX Font Info:    Redeclaring symbol font `operators' on input line 59.
LaTeX Font Info:    Encoding `OT1' has changed to `T1' for symbol font
(Font)              `operators' in the math version `normal' on input
line 59.
LaTeX Font Info:    Overwriting symbol font `operators' in version
`normal'
(Font)                  OT1/cmr/m/n --> T1/Merriwthr-OsF/m/up on input
line 59.

LaTeX Font Info:    Encoding `OT1' has changed to `T1' for symbol font
(Font)              `operators' in the math version `bold' on input line
59.
LaTeX Font Info:    Overwriting symbol font `operators' in version `bold'
(Font)                  OT1/cmr/bx/n --> T1/Merriwthr-OsF/m/up on input
line 59
.
LaTeX Font Info:    Overwriting symbol font `operators' in version `bold'
(Font)                  T1/Merriwthr-OsF/m/up --> T1/Merriwthr-OsF/b/up
on inpu
t line 59.
LaTeX Font Info:    Redeclaring math alphabet \mathbf on input line 59.
LaTeX Font Info:    Overwriting math alphabet `\mathbf' in version
`normal'
(Font)                  OT1/cmr/bx/n --> T1/Merriwthr-OsF/b/up on input
line 59
.
LaTeX Font Info:    Overwriting math alphabet `\mathbf' in version `bold'
(Font)                  OT1/cmr/bx/n --> T1/Merriwthr-OsF/b/up on input
line 59
.
LaTeX Font Info:    Redeclaring math alphabet \mathsf on input line 59.
LaTeX Font Info:    Overwriting math alphabet `\mathsf' in version
`normal'
(Font)                  OT1/cmss/m/n --> T1/MerriwthrSans-OsF/m/up on
input lin
e 59.
LaTeX Font Info:    Overwriting math alphabet `\mathsf' in version `bold'
(Font)                  OT1/cmss/bx/n --> T1/MerriwthrSans-OsF/m/up on
input li
ne 59.
LaTeX Font Info:    Redeclaring math alphabet \mathit on input line 59.
LaTeX Font Info:    Overwriting math alphabet `\mathit' in version
`normal'
(Font)                  OT1/cmr/m/it --> T1/Merriwthr-OsF/m/it on input
line 59
.
LaTeX Font Info:    Overwriting math alphabet `\mathit' in version `bold'
(Font)                  OT1/cmr/bx/it --> T1/Merriwthr-OsF/m/it on input
line 5
9.
```

```
LaTeX Font Info:    Redeclaring math alphabet \mathtt on input line 59.
LaTeX Font Info:    Overwriting math alphabet `\mathtt' in version
`normal'
(Font)                  OT1/cmtt/m/n --> T1/lmtt/m/up on input line 59.
LaTeX Font Info:    Overwriting math alphabet `\mathtt' in version `bold'
(Font)                  OT1/cmtt/m/n --> T1/lmtt/m/up on input line 59.
LaTeX Font Info:    Overwriting math alphabet `\mathsf' in version `bold'
(Font)                  T1/MerriwthrSans-OsF/m/up --> T1/MerriwthrSans-
OsF/b/up
 on input line 59.
LaTeX Font Info:    Overwriting math alphabet `\mathit' in version `bold'
(Font)                  T1/Merriwthr-OsF/m/it --> T1/Merriwthr-OsF/b/it
on inpu
t line 59.
\c@mv@tabular=\count339
\c@mv@boldtabular=\count340
(c:/texlive/2024/texmf-dist/tex/context/base/mkii/supp-pdf.mkii
[Loading MPS to PDF converter (version 2006.09.02).]
\scratchcounter=\count341
\scratchdimen=\dimen267
\scratchbox=\box81
\nofMPsegments=\count342
\nofMParguments=\count343
\everyMPshowfont=\toks41
\MPscratchCnt=\count344
\MPscratchDim=\dimen268
\MPnumerator=\count345
\makeMPintoPDFobject=\count346
\everyMPtoPDFconversion=\toks42
) (c:/texlive/2024/texmf-dist/tex/latex/epstopdf-pkg/epstopdf-base.sty
Package: epstopdf-base 2020-01-24 v2.11 Base part for package epstopdf
Package epstopdf-base Info: Redefining graphics rule for `.eps' on input
line 4
85.
(c:/texlive/2024/texmf-dist/tex/latex/latexconfig/epstopdf-sys.cfg
File: epstopdf-sys.cfg 2010/07/13 v1.3 Configuration of (r)epstopdf for
TeX Liv
e
))
*geometry* driver: auto-detecting
*geometry* detected driver: pdftex
*geometry* verbose mode - [ preamble ] result:
* driver: pdftex
* paper: a4paper
* layout: <same size as paper>
* layoutoffset:(h,v)=(0.0pt,0.0pt)
* modes: includefoot twoside
* h-part:(L,W,R)=(54.64pt, 488.22787pt, 54.64pt)
* v-part:(T,H,B)=(66.0pt, 745.04684pt, 34.0pt)
* \paperwidth=597.50787pt
* \paperheight=845.04684pt
* \textwidth=488.22787pt
* \textheight=715.04684pt
* \oddsidemargin=-17.62999pt
```

```
* \evensidemargin=-17.62999pt
* \topmargin=-47.76999pt
* \headheight=17.5pt
* \headsep=24.0pt
* \topskip=10.0pt
* \footskip=30.0pt
* \marginparwidth=48.0pt
* \marginparsep=10.0pt
* \columnsep=18.0pt
* \skip\footins=22.0pt plus 2.0pt
* \hoffset=0.0pt
* \voffset=0.0pt
* \mag=1000
* \@twocolumntrue
* \@twosidetrue
* \@mparswitchtrue
* \@reversemarginfalse
* (1in=72.27pt=25.4mm, 1cm=28.453pt)

Package caption Info: Begin \AtBeginDocument code.
Package caption Info: hyperref package is loaded.
Package caption Info: longtable package is loaded.
(c:/texlive/2024/texmf-dist/tex/latex/caption/ltcaption.sty
Package: ltcaption 2021/01/08 v1.4c longtable captions (AR)
)
Package caption Info: End \AtBeginDocument code.

(c:/texlive/2024/texmf-dist/tex/latex/translations/translations-basic-
dictionar
y-english.trsl
File: translations-basic-dictionary-english.trsl (english translation
file `tra
nslations-basic-dictionary')
)
Package translations Info: loading dictionary `translations-basic-
dictionary' f
or `english'. on input line 59.
Package hyperref Info: Link coloring ON on input line 59.
(./main.out) (./main.out)
\@outlinefile=\write3
\openout3 = `main.out'.

\@gscitedetails=\box82
\@gscitedetailsheight=\skip168
\@gsheadbox=\box83
\@gsheadboxheight=\skip169
LaTeX Font Info:    Font shape `T1/Merriwthr-OsF/b/n' will be
(Font)              scaled to size 6.5pt on input line 59.
LaTeX Font Info:    Calculating math sizes for size <7.5> on input line
59.

LaTeX Font Warning: Font shape `T1/Merriwthr-OsF/m/up' undefined
(Font)              using `T1/Merriwthr-OsF/m/n' instead on input line
59.
```

```
LaTeX Font Info:    Font shape `T1/Merriwthr-OsF/m/up' will be
(Font)              scaled to size 6.24973pt on input line 59.
LaTeX Font Info:    Font shape `T1/Merriwthr-OsF/m/up' will be
(Font)              scaled to size 5.24997pt on input line 59.
LaTeX Font Info:    Trying to load font information for U+eur on input
line 59.

(c:/texlive/2024/texmf-dist/tex/latex/amsfonts/ueur.fd
File: ueur.fd 2013/01/14 v3.01 Euler Roman
) (c:/texlive/2024/texmf-dist/tex/latex/microtype/mt-eur.cfg
File: mt-eur.cfg 2006/07/31 v1.1 microtype config. file: AMS Euler Roman
(RS)
)

LaTeX Font Warning: Font shape `OMS/cmsy/m/n' in size <7.5> not available
(Font)              size <7> substituted on input line 59.

LaTeX Font Info:    External font `cmex10' loaded for size
(Font)              <7.5> on input line 59.
LaTeX Font Info:    External font `cmex10' loaded for size
(Font)              <6.24973> on input line 59.
LaTeX Font Info:    External font `cmex10' loaded for size
(Font)              <5.24997> on input line 59.
LaTeX Font Info:    Trying to load font information for U+euf on input
line 59.

(c:/texlive/2024/texmf-dist/tex/latex/amsfonts/ueuf.fd
File: ueuf.fd 2013/01/14 v3.01 Euler Fraktur
) (c:/texlive/2024/texmf-dist/tex/latex/microtype/mt-euf.cfg
File: mt-euf.cfg 2006/07/03 v1.1 microtype config. file: AMS Euler
Fraktur (RS)

)
LaTeX Font Info:    Trying to load font information for U+eus on input
line 59.

(c:/texlive/2024/texmf-dist/tex/latex/amsfonts/ueus.fd
File: ueus.fd 2013/01/14 v3.01 Euler Script
) (c:/texlive/2024/texmf-dist/tex/latex/microtype/mt-eus.cfg
File: mt-eus.cfg 2006/07/28 v1.2 microtype config. file: AMS Euler Script
(RS)
)
LaTeX Font Info:    Trying to load font information for U+euex on input
line 59
.
(c:/texlive/2024/texmf-dist/tex/latex/amsfonts/ueuex.fd
File: ueuex.fd 2013/01/14 v3.01 Euler extra symbols
)

LaTeX Font Warning: Font shape `OML/cmm/m/it' in size <7.5> not available
(Font)              size <7> substituted on input line 59.

LaTeX Font Info:    Font shape `T1/Merriwthr-OsF/m/n' will be
```

```
(Font)              scaled to size 6.24973pt on input line 59.
LaTeX Font Info:    Font shape `T1/Merriwthr-OsF/m/n' will be
(Font)              scaled to size 5.24997pt on input line 59.
LaTeX Font Info:    Font shape `T1/Merriwthr-OsF/m/it' will be
(Font)              scaled to size 7.5pt on input line 59.
LaTeX Font Info:    Font shape `T1/Merriwthr-OsF/m/it' will be
(Font)              scaled to size 6.24973pt on input line 59.
LaTeX Font Info:    Font shape `T1/Merriwthr-OsF/m/it' will be
(Font)              scaled to size 5.24997pt on input line 59.
LaTeX Font Info:    Font shape `T1/Merriwthr-OsF/m/n' will be
(Font)              scaled to size 8.0pt on input line 59.
LaTeX Font Info:    Font shape `T1/Merriwthr-OsF/m/it' will be
(Font)              scaled to size 8.0pt on input line 59.
LaTeX Font Info:    Font shape `T1/Merriwthr-OsF/b/it' will be
(Font)              scaled to size 8.0pt on input line 59.
TextBlockOrigin set to 4pc+6.64pt x 4pc+6pt
<gigascience-logo.pdf, id=141, 99.37125pt x 33.12375pt>
File: gigascience-logo.pdf Graphic file (type pdf)
<use gigascience-logo.pdf>
Package pdftex.def Info: gigascience-logo.pdf  used on input line 94.
(pdftex.def)             Requested size: 126.00902pt x 42.0pt.

Overfull \hbox (54.64pt too wide) in paragraph at lines 94--94
[][]
 []

LaTeX Font Info:    Font shape `T1/Merriwthr-OsF/m/n' will be
(Font)              scaled to size 14.0pt on input line 94.
LaTeX Font Info:    Font shape `T1/Merriwthr-OsF/m/n' will be
(Font)              scaled to size 8.99997pt on input line 94.
LaTeX Font Info:    Calculating math sizes for size <14> on input line
94.
LaTeX Font Info:    Font shape `T1/Merriwthr-OsF/m/up' will be
(Font)              scaled to size 14.0pt on input line 94.
LaTeX Font Info:    Font shape `T1/Merriwthr-OsF/m/up' will be
(Font)              scaled to size 11.66617pt on input line 94.
LaTeX Font Info:    Font shape `T1/Merriwthr-OsF/m/up' will be
(Font)              scaled to size 9.79996pt on input line 94.
LaTeX Font Info:    External font `cmex10' loaded for size
(Font)              <14> on input line 94.
LaTeX Font Info:    External font `cmex10' loaded for size
(Font)              <11.66617> on input line 94.
LaTeX Font Info:    External font `cmex10' loaded for size
(Font)              <9.79996> on input line 94.
LaTeX Font Info:    Font shape `T1/Merriwthr-OsF/m/n' will be
(Font)              scaled to size 11.66617pt on input line 94.
LaTeX Font Info:    Font shape `T1/Merriwthr-OsF/m/n' will be
(Font)              scaled to size 9.79996pt on input line 94.
LaTeX Font Info:    Font shape `T1/Merriwthr-OsF/m/it' will be
(Font)              scaled to size 14.0pt on input line 94.
LaTeX Font Info:    Font shape `T1/Merriwthr-OsF/m/it' will be
(Font)              scaled to size 11.66617pt on input line 94.
LaTeX Font Info:    Font shape `T1/Merriwthr-OsF/m/it' will be
(Font)              scaled to size 9.79996pt on input line 94.
```

```
LaTeX Font Info:    Font shape `T1/Merriwthr-OsF/b/n' will be
(Font)              scaled to size 18.0pt on input line 94.
LaTeX Font Info:    Font shape `T1/Merriwthr-OsF/m/n' will be
(Font)              scaled to size 13.0pt on input line 94.
LaTeX Font Info:    Calculating math sizes for size <13> on input line
94.
LaTeX Font Info:    Font shape `T1/Merriwthr-OsF/m/up' will be
(Font)              scaled to size 13.0pt on input line 94.
LaTeX Font Info:    Font shape `T1/Merriwthr-OsF/m/up' will be
(Font)              scaled to size 10.83287pt on input line 94.
LaTeX Font Info:    Font shape `T1/Merriwthr-OsF/m/up' will be
(Font)              scaled to size 9.09996pt on input line 94.

LaTeX Font Warning: Font shape `OMS/cmsy/m/n' in size <13> not available
(Font)              size <12> substituted on input line 94.

LaTeX Font Info:    External font `cmex10' loaded for size
(Font)              <13> on input line 94.
LaTeX Font Info:    External font `cmex10' loaded for size
(Font)              <10.83287> on input line 94.
LaTeX Font Info:    External font `cmex10' loaded for size
(Font)              <9.09996> on input line 94.

LaTeX Font Warning: Font shape `OML/cmm/m/it' in size <13> not available
(Font)              size <12> substituted on input line 94.

LaTeX Font Info:    Font shape `T1/Merriwthr-OsF/m/n' will be
(Font)              scaled to size 10.83287pt on input line 94.
LaTeX Font Info:    Font shape `T1/Merriwthr-OsF/m/n' will be
(Font)              scaled to size 9.09996pt on input line 94.
LaTeX Font Info:    Font shape `T1/Merriwthr-OsF/m/it' will be
(Font)              scaled to size 13.0pt on input line 94.
LaTeX Font Info:    Font shape `T1/Merriwthr-OsF/m/it' will be
(Font)              scaled to size 10.83287pt on input line 94.
LaTeX Font Info:    Font shape `T1/Merriwthr-OsF/m/it' will be
(Font)              scaled to size 9.09996pt on input line 94.
LaTeX Font Info:    Trying to load font information for TS1+Merriwthr-OsF
on in
put line 94.
(c:/texlive/2024/texmf-dist/tex/latex/merriweather/TS1Merriwthr-OsF.fd
File: TS1Merriwthr-OsF.fd 2020/08/30 (autoinst) Font definitions for
TS1/Merriw
thr-OsF.
)
LaTeX Font Info:    Font shape `TS1/Merriwthr-OsF/m/n' will be
(Font)              scaled to size 10.83287pt on input line 94.
Package microtype Info: Loading generic protrusion settings for font
family
(microtype)             `Merriwthr-OsF' (encoding: TS1).
(microtype)             For optimal results, create family-specific
settings.
(microtype)             See the microtype manual for details.
LaTeX Font Info:    Font shape `T1/Merriwthr-OsF/m/n' will be
(Font)              scaled to size 9.0pt on input line 94.
```

```
LaTeX Font Info:    Font shape `T1/Merriwthr-OsF/m/up' will be
(Font)              scaled to size 9.0pt on input line 94.
LaTeX Font Info:    Font shape `T1/Merriwthr-OsF/m/up' will be
(Font)              scaled to size 7.0pt on input line 94.
LaTeX Font Info:    Font shape `T1/Merriwthr-OsF/m/up' will be
(Font)              scaled to size 5.0pt on input line 94.
LaTeX Font Info:    External font `cmex10' loaded for size
(Font)              <9> on input line 94.
LaTeX Font Info:    External font `cmex10' loaded for size
(Font)              <7> on input line 94.
LaTeX Font Info:    External font `cmex10' loaded for size
(Font)              <5> on input line 94.
LaTeX Font Info:    Font shape `T1/Merriwthr-OsF/m/n' will be
(Font)              scaled to size 7.0pt on input line 94.
LaTeX Font Info:    Font shape `T1/Merriwthr-OsF/m/n' will be
(Font)              scaled to size 5.0pt on input line 94.
LaTeX Font Info:    Font shape `T1/Merriwthr-OsF/m/it' will be
(Font)              scaled to size 9.0pt on input line 94.
LaTeX Font Info:    Font shape `T1/Merriwthr-OsF/m/it' will be
(Font)              scaled to size 7.0pt on input line 94.
LaTeX Font Info:    Font shape `T1/Merriwthr-OsF/m/it' will be
(Font)              scaled to size 5.0pt on input line 94.
LaTeX Font Info:    Font shape `T1/Merriwthr-OsF/m/n' will be
(Font)              scaled to size 6.5pt on input line 94.
LaTeX Font Info:    Calculating math sizes for size <6.5> on input line
94.
LaTeX Font Info:    Font shape `T1/Merriwthr-OsF/m/up' will be
(Font)              scaled to size 6.5pt on input line 94.
LaTeX Font Info:    Font shape `T1/Merriwthr-OsF/m/up' will be
(Font)              scaled to size 5.41643pt on input line 94.
LaTeX Font Info:    Font shape `T1/Merriwthr-OsF/m/up' will be
(Font)              scaled to size 4.54997pt on input line 94.

LaTeX Font Warning: Font shape `OMS/cmsy/m/n' in size <6.5> not available
(Font)              size <6> substituted on input line 94.


LaTeX Font Warning: Font shape `OMS/cmsy/m/n' in size <5.41643> not
available
(Font)              size <5> substituted on input line 94.


LaTeX Font Warning: Font shape `OMS/cmsy/m/n' in size <4.54997> not
available
(Font)              size <5> substituted on input line 94.

LaTeX Font Info:    External font `cmex10' loaded for size
(Font)              <6.5> on input line 94.
LaTeX Font Info:    External font `cmex10' loaded for size
(Font)              <5.41643> on input line 94.
LaTeX Font Info:    External font `cmex10' loaded for size
(Font)              <4.54997> on input line 94.

LaTeX Font Warning: Font shape `OML/cmm/m/it' in size <6.5> not available
```

```
(Font)              size <6> substituted on input line 94.


LaTeX Font Warning: Font shape `OML/cmm/m/it' in size <5.41643> not 
available
(Font)              size <5> substituted on input line 94.


LaTeX Font Warning: Font shape `OML/cmm/m/it' in size <4.54997> not 
available
(Font)              size <5> substituted on input line 94.

LaTeX Font Info:    Font shape `T1/Merriwthr-OsF/m/n' will be
(Font)              scaled to size 5.41643pt on input line 94.
LaTeX Font Info:    Font shape `T1/Merriwthr-OsF/m/n' will be
(Font)              scaled to size 4.54997pt on input line 94.
LaTeX Font Info:    Font shape `T1/Merriwthr-OsF/m/it' will be
(Font)              scaled to size 6.5pt on input line 94.
LaTeX Font Info:    Font shape `T1/Merriwthr-OsF/m/it' will be
(Font)              scaled to size 5.41643pt on input line 94.
LaTeX Font Info:    Font shape `T1/Merriwthr-OsF/m/it' will be
(Font)              scaled to size 4.54997pt on input line 94.
LaTeX Font Info:    Font shape `TS1/Merriwthr-OsF/m/n' will be
(Font)              scaled to size 5.41643pt on input line 94.

Overfull \hbox (54.64pt too wide) in paragraph at lines 94--94
[][][]
 []

LaTeX Font Info:    Font shape `T1/Merriwthr-OsF/b/n' will be
(Font)              scaled to size 10.0pt on input line 94.
LaTeX Font Info:    Font shape `T1/Merriwthr-OsF/b/n' will be
(Font)              scaled to size 8.0pt on input line 94.

Overfull \hbox (54.64pt too wide) in paragraph at lines 94--94
[][][]
 []

Package mdframed Info: mdframed works in twoside mode on input line 97.
LaTeX Font Info:    Font shape `T1/Merriwthr-OsF/b/n' will be
(Font)              scaled to size 8.2pt on input line 97.
LaTeX Font Info:    Font shape `TS1/Merriwthr-OsF/m/n' will be
(Font)              scaled to size 7.5pt on input line 99.
Package mdframed Info: mdframed inside float
 mdframed uses option nobreak mdframed on input line 109.
Package mdframed Info: mdframed inside a box
 mdframed uses option nobreak mdframed on input line 109.
LaTeX Font Info:    Font shape `T1/Merriwthr-OsF/m/up' will be
(Font)              scaled to size 7.5pt on input line 113.
LaTeX Font Info:    Font shape `T1/Merriwthr-OsF/b/n' will be
(Font)              scaled to size 7.5pt on input line 113.

Package natbib Warning: Citation `ncdriskfactorcollaborationncd-
riscWorldwideTr
```

```
endsBodymass2017' on page 1 undefined on input line 113.


Package natbib Warning: Citation
`worldhealthorganizationwhoFactSheetsObesity20
21' on page 1 undefined on input line 113.


Package natbib Warning: Citation `bluherObesityGlobalEpidemiology2019' on
page
1 undefined on input line 113.


Underfull \vbox (badness 10000) has occurred while \output is active []




Overfull \vbox (3.93274pt too high) has occurred while \output is active
[]



LaTeX Font Info:    Font shape `T1/Merriwthr-OsF/m/n' will be
(Font)              scaled to size 7.8pt on input line 114.
LaTeX Font Info:    Font shape `T1/Merriwthr-OsF/b/n' will be
(Font)              scaled to size 7.8pt on input line 114.
[1{c:/texlive/2024/texmf-
var/fonts/map/pdftex/updmap/pdftex.map}{c:/texlive/202
4/texmf-
dist/fonts/enc/dvips/merriweather/merriwthr_posqbl.enc}{c:/texlive/2024
/texmf-dist/fonts/enc/dvips/merriweather/merriwthr_owzwzj.enc}


 <./gigascience-logo.pdf>]

Package natbib Warning: Citation `barbosaAberrantImpulseControl2022' on
page 2
undefined on input line 115.


Package natbib Warning: Citation `leighRoleRewardCircuitry2018' on page 2
undef
ined on input line 115.


Package natbib Warning: Citation
`ziauddeenObesityNeurocognitiveBasis2015' on p
age 2 undefined on input line 115.


Package natbib Warning: Citation `verdejo-romanBrainRewardSystem2017' on
page 2
 undefined on input line 115.
```

Package natbib Warning: Citation `yangExecutiveFunctionPerformance2018b' on pag
e 2 undefined on input line 115.

Package natbib Warning: Citation `fitzpatrickSystematicReviewAre2013b' on page
2 undefined on input line 115.

Package natbib Warning: Citation `olivoLowNeuroticismCognitive2019' on page 2 u
ndefined on input line 115.

Package natbib Warning: Citation `garcia-garciaNeuroanatomicalDifferencesObesit
y2019a' on page 2 undefined on input line 117.

Package natbib Warning: Citation `gomez-apoStructuralBrainChanges2021' on page
2 undefined on input line 117.

Package natbib Warning: Citation `glicksteinCerebellumHistory2009' on page 2 un
defined on input line 119.

Package natbib Warning: Citation `guellMetalinguisticDeficitsPatients2015' on p
age 2 undefined on input line 119.

Package natbib Warning: Citation `hocheCerebellarCognitiveAffective2018' on pag
e 2 undefined on input line 119.

Package natbib Warning: Citation `hocheCerebellarContributionSocial2016' on pag
e 2 undefined on input line 119.

Package natbib Warning: Citation `koziolMovementThoughtExecutive2012' on page 2
undefined on input line 119.

Package natbib Warning: Citation `mantoSchmahmannSyndromeIdentification2015' on

page 2 undefined on input line 119.

Package natbib Warning: Citation `marienLanguageCerebellum2018' on page 2
undef
ined on input line 119.

Package natbib Warning: Citation `schmahmannCerebellumCognition2019a' on
page 2
undefined on input line 119.

Package natbib Warning: Citation `guellFunctionalGradientsCerebellum2018'
on pa
ge 2 undefined on input line 121.

Package natbib Warning: Citation `guellTripleRepresentationLanguage2018'
on pag
e 2 undefined on input line 121.

Package natbib Warning: Citation
`habasDistinctCerebellarContributions2009' on
page 2 undefined on input line 121.

Package natbib Warning: Citation `marekSpatialTemporalOrganization2018'
on page
2 undefined on input line 121.

Package natbib Warning: Citation
`bucknerOrganizationHumanCerebellum2011a' on p
age 2 undefined on input line 121.

Package natbib Warning: Citation `keren-
happuchMetaanalysisCerebellarContributi
ons2014' on page 2 undefined on input line 121.

Package natbib Warning: Citation `stoodleyFunctionalTopographyHuman2022'
on pag
e 2 undefined on input line 121.

Package natbib Warning: Citation `stoodleyFunctionalTopographyHuman2009'
on pag
e 2 undefined on input line 121.

```
Package natbib Warning: Citation `shenUsingConnectomebasedPredictive2017'
on pa
ge 2 undefined on input line 124.


Package natbib Warning: Citation `glasserHumanConnectomeProject2016' on
page 2
undefined on input line 130.


Package natbib Warning: Citation
`glasserMinimalPreprocessingPipelines2013' on
page 2 undefined on input line 134.



LaTeX Font Info:    Font shape `T1/Merriwthr-OsF/m/it' will be
(Font)              scaled to size 7.8pt on input line 135.
[2]

Package natbib Warning: Citation `robinsonMSMNewFlexible2014' on page 3
undefin
ed on input line 136.


Package natbib Warning: Citation `glasserMultimodalParcellationHuman2016'
on pa
ge 3 undefined on input line 136.


Package natbib Warning: Citation `glasserMultimodalParcellationHuman2016'
on pa
ge 3 undefined on input line 136.


Package natbib Warning: Citation
`coalsonImpactTraditionalNeuroimaging2018' on
page 3 undefined on input line 136.


Package natbib Warning: Citation `coalsonCIFTI2ConnectivityFile2014' on
page 3
undefined on input line 138.


Package natbib Warning: Citation
`glasserMinimalPreprocessingPipelines2013' on
page 3 undefined on input line 138.


Package natbib Warning: Citation
`coalsonImpactTraditionalNeuroimaging2018' on
page 3 undefined on input line 142.
```

```
Package natbib Warning: Citation `glasserMultimodalParcellationHuman2016'
on pa
ge 3 undefined on input line 142.


Package natbib Warning: Citation
`karlfristonFunctionalIntegrationBrain2004' on
 page 3 undefined on input line 142.


Package natbib Warning: Citation `glasserMultimodalParcellationHuman2016'
on pa
ge 3 undefined on input line 142.


Package natbib Warning: Citation `tianTopographicOrganizationHuman2020'
on page
 3 undefined on input line 142.


Package natbib Warning: Citation
`renConnectivitybasedParcellationImproved2019'
 on page 3 undefined on input line 142.


LaTeX Warning: File `img/parcellated_correlation_explainer_v2.png' not
found on
 input line 146.


! Package pdftex.def Error: File
`img/parcellated_correlation_explainer_v2.png'
 not found: using draft setting.

See the pdftex.def package documentation for explanation.
Type  H <return>  for immediate help.
 ...

l.146 ...parcellated_correlation_explainer_v2.png}

Try typing  <return>  to proceed.
If that doesn't work, type  X <return>  to quit.

LaTeX Font Info:    Trying to load font information for T1+lmtt on input
line 1
46.
(c:/texlive/2024/texmf-dist/tex/latex/lm/t1lmtt.fd
File: t1lmtt.fd 2015/05/01 v1.6.1 Font defs for Latin Modern
)
Package microtype Info: Loading generic protrusion settings for font
family
(microtype)             `lmtt' (encoding: T1).
```

```
(microtype)             For optimal results, create family-specific
settings.
(microtype)             See the microtype manual for details.
LaTeX Font Info:    Font shape `T1/Merriwthr-OsF/m/n' will be
(Font)              scaled to size 6.0pt on input line 155.
LaTeX Font Info:    Font shape `T1/Merriwthr-OsF/b/n' will be
(Font)              scaled to size 6.0pt on input line 155.



Package natbib Warning: Citation `abrahamMachineLearningNeuroimaging2014'
on pa
ge 3 undefined on input line 158.


Package natbib Warning: Citation
`pedregosaScikitlearnMachineLearning2011' on p
age 3 undefined on input line 158.


Package natbib Warning: Citation `ngTransportRiemannianManifold2014' on
page 3
undefined on input line 158.


Package natbib Warning: Citation
`varoquauxDetectionBrainFunctionalConnectivity
2010' on page 3 undefined on input line 158.


Package natbib Warning: Citation
`dadiBenchmarkingFunctionalConnectomebased2019
' on page 3 undefined on input line 158.


Package natbib Warning: Citation `pervaizOptimisingNetworkModelling2020'
on pag
e 3 undefined on input line 158.


Package natbib Warning: Citation
`ledoitWellconditionedEstimatorLargedimensiona
l2004' on page 3 undefined on input line 158.

LaTeX Font Info:    Font shape `T1/Merriwthr-OsF/b/n' will be
(Font)              scaled to size 8.5pt on input line 162.

Package natbib Warning: Citation `rohdeGeneticsEpigeneticsObesity2019' on
page
3 undefined on input line 185.


Package natbib Warning: Citation `goodarziGeneticsObesityWhat2018' on
page 3 un
```

defined on input line 185.

Package natbib Warning: Citation `xuMetaconnectomicAnalysisMaps2022' on
page 3
undefined on input line 185.

Package natbib Warning: Citation
`fornitoGeneticInfluencesCostEfficient2011' on
page 3 undefined on input line 185.

Package natbib Warning: Citation `glahnGeneticControlResting2010' on page
3 und
efined on input line 185.

Package natbib Warning: Citation `thompsonGeneticsConnectome2013' on page
3 und
efined on input line 185.

Package natbib Warning: Citation
`vandenheuvelGeneticControlFunctional2013' on
page 3 undefined on input line 185.

[3]

LaTeX Warning: File `./img/CPM_explainer.png' not found on input line
202.

! Package pdftex.def Error: File `./img/CPM_explainer.png' not found:
using dra
ft setting.

See the pdftex.def package documentation for explanation.
Type H <return> for immediate help.
...

l.202 ...dth=1\textwidth]{./img/CPM_explainer.png}

Try typing <return> to proceed.
If that doesn't work, type X <return> to quit.

LaTeX Font Info: Font shape `T1/Merriwthr-OsF/m/it' will be
(Font)   scaled to size 6.0pt on input line 205.

Underfull \hbox (badness 3068) in paragraph at lines 209--209
|\T1/Merriwthr-OsF/b/n/8.5 (+20) Task-based func-tional mag-netic res-o-
nance i

```
mag-ing
 []


Package natbib Warning: Citation `barchFunctionHumanConnectome2013' on
page 4 u
ndefined on input line 211.


Package natbib Warning: Citation `barchFunctionHumanConnectome2013' on
page 4 u
ndefined on input line 213.


Package natbib Warning: Citation `haririAmygdalaResponseEmotional2002a'
on page
 4 undefined on input line 213.


Package natbib Warning: Citation `barchFunctionHumanConnectome2013' on
page 4 u
ndefined on input line 215.


Package natbib Warning: Citation
`delgadoTrackingHemodynamicResponses2000' on p
age 4 undefined on input line 215.


Package natbib Warning: Citation `barchFunctionHumanConnectome2013' on
page 4 u
ndefined on input line 217.


Package natbib Warning: Citation `binderMappingAnteriorTemporal2011' on
page 4
undefined on input line 217.


Package natbib Warning: Citation `barchFunctionHumanConnectome2013' on
page 4 u
ndefined on input line 219.


Package natbib Warning: Citation
`smithLocalizingRostrolateralPrefrontal2007a'
on page 4 undefined on input line 219.


Package natbib Warning: Citation `barchFunctionHumanConnectome2013' on
page 4 u
ndefined on input line 221.
```

```
Package natbib Warning: Citation `castelliMovementMindFunctional2000' on
page 4
 undefined on input line 221.


Package natbib Warning: Citation `wheatleyUnderstandingAnimateAgents2007'
on pa
ge 4 undefined on input line 221.




Package natbib Warning: Citation `barchFunctionHumanConnectome2013' on
page 4 u
ndefined on input line 223.


Package natbib Warning: Citation `downingCorticalAreaSelective2001' on
page 4 u
ndefined on input line 223.

LaTeX Font Info:    Font shape `T1/Merriwthr-OsF/m/n' will be
(Font)              scaled to size 6.25008pt on input line 231.

Package natbib Warning: Citation `eklundClusterFailureWhy2016' on page 4
undefi
ned on input line 237.


Package natbib Warning: Citation `moritzRayDistributedFramework2018' on
page 4
undefined on input line 237.



[4{c:/texlive/2024/texmf-dist/fonts/enc/dvips/lm/lm-ec.enc}] [5]

Package natbib Warning: Citation `barchFunctionHumanConnectome2013' on
page 6 u
ndefined on input line 241.


Package natbib Warning: Citation `wu-
minnhcpconsortium1200SubjectsData2017' on
page 6 undefined on input line 241.


Package natbib Warning: Citation `barchFunctionHumanConnectome2013' on
page 6 u
ndefined on input line 243.


Package natbib Warning: Citation `wu-
minnhcpconsortium1200SubjectsData2017' on
```

```
page 6 undefined on input line 243.

LaTeX Font Info:    Font shape `T1/Merriwthr-OsF/b/n' will be
(Font)              scaled to size 7.0pt on input line 267.

Underfull \hbox (badness 10000) in alignment at lines 292--292
[][][][][][][][]
 []

LaTeX Font Info:    Font shape `T1/Merriwthr-OsF/m/up' will be
(Font)              scaled to size 6.0pt on input line 294.
LaTeX Font Info:    External font `cmex10' loaded for size
(Font)              <6> on input line 294.

Package natbib Warning: Citation
`almendeb.v.andcontributorsVisNetworkNetworkVi
sualization2022' on page 6 undefined on input line 303.


Package natbib Warning: Citation
`rcoreteamLanguageEnvironmentStatistical2022'
on page 6 undefined on input line 303.





[6]

LaTeX Warning: File `./img/rsfmri_net_comb2.png' not found on input line
314.


! Package pdftex.def Error: File `./img/rsfmri_net_comb2.png' not found:
using
draft setting.

See the pdftex.def package documentation for explanation.
Type  H <return>  for immediate help.
 ...

l.314 ....7\textwidth]{./img/rsfmri_net_comb2.png}

Try typing  <return>  to proceed.
If that doesn't work, type  X <return>  to quit.

LaTeX Font Info:    Font shape `T1/Merriwthr-OsF/b/sl' in size <7.5> not
availa
ble
(Font)              Font shape `T1/Merriwthr-OsF/b/it' tried instead on
input l
ine 344.
LaTeX Font Info:    Font shape `T1/Merriwthr-OsF/b/it' will be
(Font)              scaled to size 7.5pt on input line 344.
```

```
Underfull \vbox (badness 10000) has occurred while \output is active []




[7]

LaTeX Warning: File `./img/figure-results_rsfmri.png' not found on input line 3
95.


! Package pdftex.def Error: File `./img/figure-results_rsfmri.png' not found: u
sing draft setting.

See the pdftex.def package documentation for explanation.
Type  H <return>  for immediate help.
 ...

l.395 ...xtwidth]{./img/figure-results_rsfmri.png}

Try typing  <return>  to proceed.
If that doesn't work, type  X <return>  to quit.

LaTeX Font Info:    Font shape `T1/Merriwthr-OsF/m/it' will be
(Font)              scaled to size 6.25008pt on input line 424.
LaTeX Font Info:    Calculating math sizes for size <6.25008> on input line 424
.
LaTeX Font Info:    Font shape `T1/Merriwthr-OsF/m/up' will be
(Font)              scaled to size 6.25008pt on input line 424.
LaTeX Font Info:    Font shape `T1/Merriwthr-OsF/m/up' will be
(Font)              scaled to size 5.20816pt on input line 424.
LaTeX Font Info:    Font shape `T1/Merriwthr-OsF/m/up' will be
(Font)              scaled to size 4.37503pt on input line 424.

LaTeX Font Warning: Font shape `OMS/cmsy/m/n' in size <4.37503> not available
(Font)              size <5> substituted on input line 424.

LaTeX Font Info:    External font `cmex10' loaded for size
(Font)              <6.25008> on input line 424.
LaTeX Font Info:    External font `cmex10' loaded for size
(Font)              <5.20816> on input line 424.
LaTeX Font Info:    External font `cmex10' loaded for size
(Font)              <4.37503> on input line 424.

LaTeX Font Warning: Font shape `OML/cmm/m/it' in size <4.37503> not available
(Font)              size <5> substituted on input line 424.
```

```
LaTeX Font Info:    Font shape `T1/Merriwthr-OsF/m/n' will be
(Font)              scaled to size 5.20816pt on input line 424.
LaTeX Font Info:    Font shape `T1/Merriwthr-OsF/m/n' will be
(Font)              scaled to size 4.37503pt on input line 424.
LaTeX Font Info:    Font shape `T1/Merriwthr-OsF/m/it' will be
(Font)              scaled to size 5.20816pt on input line 424.
LaTeX Font Info:    Font shape `T1/Merriwthr-OsF/m/it' will be
(Font)              scaled to size 4.37503pt on input line 424.

Underfull \hbox (badness 10000) in paragraph at lines 424--424
 [][]\T1/Merriwthr-OsF/m/up/6.25008 (+20) For the es-tab-lished brain
net-works
, data were down-loaded from [][]$\T1/lmtt/m/n/6.25008 https : / / surfer
. nmr
 . mgh . harvard . edu / fswiki / CorticalParcellation _ Yeo2011$[][]
\T1/Merri
wthr-OsF/m/up/6.25008 (+20) and
 []


Underfull \hbox (badness 10000) in paragraph at lines 424--424
 \T1/Merriwthr-OsF/m/up/6.25008 (+20) plot-ted with nilearn (file-name
was \T1/
lmtt/m/n/6.25008
Yeo2011_17Networks_MNI152_FreeSurferConformed1mm_LiberalMask.n
ii.gz \T1/Merriwthr-OsF/m/up/6.25008 (+20) for the 17 net-work par-cel-
la-tion
and
 []


LaTeX Warning: File `./img/figure-results_tfmri.png' not found on input
line 43
1.


! Package pdftex.def Error: File `./img/figure-results_tfmri.png' not
found: us
ing draft setting.

See the pdftex.def package documentation for explanation.
Type  H <return>  for immediate help.
 ...

l.431 ...extwidth]{./img/figure-results_tfmri.png}

Try typing  <return>  to proceed.
If that doesn't work, type  X <return>  to quit.


Package natbib Warning: Citation `triarhouProposedNumberSystem2007' on
page 8 u
ndefined on input line 479.
```

```
Package natbib Warning: Citation `glasserMultimodalParcellationHuman2016'
on pa
ge 8 undefined on input line 479.


Package natbib Warning: Citation `diannepattersonAtlases2023' on page 8
undefin
ed on input line 479.


Underfull \hbox (badness 10000) in alignment at lines 544--544
[][][][][][][][]
 []


Underfull \vbox (badness 3471) has occurred while \output is active []




Overfull \hbox (23.00003pt too wide) in alignment at lines 635--635
[][][][][][][][]
 []


LaTeX Warning: File `./img/figure-overlap.png' not found on input line
647.


! Package pdftex.def Error: File `./img/figure-overlap.png' not found:
using dr
aft setting.

See the pdftex.def package documentation for explanation.
Type  H <return>  for immediate help.
 ...

l.647 ...=0.6\textwidth]{./img/figure-overlap.png}

Try typing  <return>  to proceed.
If that doesn't work, type  X <return>  to quit.

LaTeX Font Info:    Font shape `T1/Merriwthr-OsF/b/n' will be
(Font)              scaled to size 6.25008pt on input line 669.

Package natbib Warning: Citation `wickhamGgplot2ElegantGraphics2016' on
page 8
undefined on input line 669.


LaTeX Warning: File `./img/figure-cerebellar_edges.png' not found on
input line
 675.
```

```
! Package pdftex.def Error: File `./img/figure-cerebellar_edges.png' not
found:
 using draft setting.

See the pdftex.def package documentation for explanation.
Type  H <return>  for immediate help.
 ...

l.675 ...width]{./img/figure-cerebellar_edges.png}

Try typing  <return>  to proceed.
If that doesn't work, type  X <return>  to quit.



[8] [9] [10]


Package natbib Warning: Citation `menonSalienceNetwork2015' on page 11
undefine
d on input line 750.


Package natbib Warning: Citation
`petersCorticoStriatalThalamicLoopCircuits2016
' on page 11 undefined on input line 750.


Package natbib Warning: Citation `tomiyamaDysfunctionDorsalCaudate2019'
on page
 11 undefined on input line 750.


Package natbib Warning: Citation `tanObeseIndividualsShow2021' on page 11
undef
ined on input line 750.


Package natbib Warning: Citation `garcia-
garciaAlterationsSalienceNetwork2013'
on page 11 undefined on input line 750.


Package natbib Warning: Citation `wittWhatExecutiveFunction2021' on page
11 und
efined on input line 752.



[11]
```

```
Package natbib Warning: Citation `raichleBrainDefaultMode2015' on page 12 undef
ined on input line 756.


Package natbib Warning: Citation `foxHumanBrainIntrinsically2005' on page 12 un
defined on input line 756.


Package natbib Warning: Citation `shannonPremotorFunctionalConnectivity2011' on
 page 12 undefined on input line 756.


Underfull \vbox (badness 10000) has occurred while \output is active []



[12]

Package natbib Warning: Citation `stoodleyLocationLesionDetermines2016a' on pag
e 13 undefined on input line 805.


Package natbib Warning: Citation `smaersBrainSizeExpansion2019' on page 13 unde
fined on input line 805.


Package natbib Warning: Citation `balstersEvolutionCerebellarCortex2010' on pag
e 13 undefined on input line 805.


Package natbib Warning: Citation `weaverReciprocalEvolutionCerebellum2005' on p
age 13 undefined on input line 805.


Package natbib Warning: Citation `whitingEvolutionCorticocerebellarComplex2003'
 on page 13 undefined on input line 805.


Package natbib Warning: Citation `kingFunctionalBoundariesHuman2019' on page 13
 undefined on input line 807.
```

Package natbib Warning: Citation
`stoodleyEvidenceTopographicOrganization2010'
on page 13 undefined on input line 807.

Package natbib Warning: Citation `stoodleyFunctionalTopographyHuman2009'
on pag
e 13 undefined on input line 807.

Underfull \vbox (badness 4181) has occurred while \output is active []

Package natbib Warning: Citation `guellFunctionalGradientsCerebellum2018'
on pa
ge 13 undefined on input line 809.

Package natbib Warning: Citation `guellCerebellarFunctionalAnatomy2020'
on page
13 undefined on input line 809.

Package natbib Warning: Citation `kingFunctionalBoundariesHuman2019' on
page 13
undefined on input line 809.

Underfull \hbox (badness 1221) in paragraph at lines 809--810
\T1/Merriwthr-OsF/m/up/7.5 (+20) motor func-tions (task-negative, i.e.
DMN-rela
ted, and task-
[]

Package natbib Warning: Citation `saderCerebellumPlaysMore2023' on page
13 unde
fined on input line 811.

Package natbib Warning: Citation `bermanEffectsLeptinDeficiency2013' on
page 13
undefined on input line 811.

Package natbib Warning: Citation
`volkowObesityAddictionNeurobiological2013' on
page 13 undefined on input line 813.

Package natbib Warning: Citation `carnellAmodalBrainActivation2014' on
page 13

```
undefined on input line 813.


Package natbib Warning: Citation `tomasiOverlappingPatternsBrain2015' on page 1
3 undefined on input line 813.


Package natbib Warning: Citation `cartaCerebellarModulationReward2019' on page
13 undefined on input line 813.


Package natbib Warning: Citation `kostadinovPredictiveReactiveReward2019' on pa
ge 13 undefined on input line 813.


Underfull \vbox (badness 1237) has occurred while \output is active []



[13]

Package natbib Warning: Citation `ernstCerebellumInvolvedProcessing2019' on pag
e 14 undefined on input line 815.


Package natbib Warning: Citation `milaneschiDepressionObesityEvidence2019' on p
age 14 undefined on input line 815.


Package natbib Warning: Citation `gariepyAssociationObesityAnxiety2010' on page
 14 undefined on input line 815.


Package natbib Warning: Citation `iosifCerebellarPredictionFeeding2023' on page
 14 undefined on input line 815.


Package natbib Warning: Citation `dmelloEvidenceHierarchicalCognitive2020' on p
age 14 undefined on input line 817.


Package natbib Warning: Citation `oldratiTargetingHumanCerebellum2018' on page
14 undefined on input line 817.
```

Package natbib Warning: Citation `stoodleyFunctionalTopographyHuman2009' on pag
e 14 undefined on input line 817.

Package natbib Warning: Citation `starowicz-filipCerebellarFunctionalLateraliza
tion2021' on page 14 undefined on input line 817.

Package natbib Warning: Citation `habasDistinctCerebellarContributions2009' on
page 14 undefined on input line 821.

Package natbib Warning: Citation `uddinUniversalTaxonomyMacroscale2019' on page
 14 undefined on input line 821.

Package natbib Warning: Citation `wittWhatExecutiveFunction2021' on page 14 und
efined on input line 821.

Package natbib Warning: Citation `wittWhatExecutiveFunction2021' on page 14 und
efined on input line 823.

Package natbib Warning: Citation `cristoforiExecutiveFunctions2019' on page 14
undefined on input line 823.

Package natbib Warning: Citation `hernandezMedialPrefrontalperirhinalCortical20
17' on page 14 undefined on input line 823.

Package natbib Warning: Citation `skranesEntorhinalCorticalThinning2012' on pag
e 14 undefined on input line 823.

Package natbib Warning: Citation `izenRestingStateConnectivity2018' on page 14
undefined on input line 823.

Package natbib Warning: Citation `friedmanRolePrefrontalCortex2022' on page 14
undefined on input line 823.

Package natbib Warning: Citation
`favieriExecutiveFunctionsOverweight2019' on p
age 14 undefined on input line 825.

Package natbib Warning: Citation `yangExecutiveFunctionPerformance2018b'
on pag
e 14 undefined on input line 825.

Package natbib Warning: Citation `fitzpatrickSystematicReviewAre2013b' on
page
14 undefined on input line 825.

Package natbib Warning: Citation `smithReviewAssociationObesity2011' on
page 14
undefined on input line 825.

Package natbib Warning: Citation `coppinWorkingMemoryReward2014' on page
14 und
efined on input line 825.

Package natbib Warning: Citation
`dohleExecutiveFunctionsSelfregulation2018' on
page 14 undefined on input line 825.

Package natbib Warning: Citation `eichenTargetingExecutiveFunction2021'
on page
14 undefined on input line 825.

Package natbib Warning: Citation
`schmahmannCerebellarCognitiveAffective1998a'
on page 14 undefined on input line 827.

Package natbib Warning: Citation
`argyropoulosCerebellarCognitiveAffective2020'
on page 14 undefined on input line 827.

Package natbib Warning: Citation `herlinTemporalPoleAnatomy2021' on page
14 und
efined on input line 829.

```
Package natbib Warning: Citation `pascualLargeScaleBrainNetworks2015' on
page 1
4 undefined on input line 829.


Package natbib Warning: Citation `pattersonWhereYouKnow2007' on page 14
undefin
ed on input line 829.


Package natbib Warning: Citation
`pobricAmodalSemanticRepresentations2010' on p
age 14 undefined on input line 829.


Package natbib Warning: Citation
`schroeterDissociatingBehavioralDisorders2011'
 on page 14 undefined on input line 829.


Package natbib Warning: Citation `schroeterExecutiveDeficitsAre2012' on
page 14
 undefined on input line 829.


Package natbib Warning: Citation `zhuAberrantFunctionalConnectivity2022'
on pag
e 14 undefined on input line 835.


Package natbib Warning: Citation
`kebetsSomatosensoryMotorDysconnectivitySpans2
019' on page 14 undefined on input line 835.


Package natbib Warning: Citation
`reppSensorimotorSynchronizationReview2013' on
 page 14 undefined on input line 835.


Package natbib Warning: Citation `wienerImageTimeVoxelwise2010' on page
14 unde
fined on input line 835.



[14]

Package natbib Warning: Citation
`comstockSensorimotorSynchronizationAuditory20
18' on page 15 undefined on input line 837.
```

```
Package natbib Warning: Citation `uddinUniversalTaxonomyMacroscale2019'
on page
 15 undefined on input line 837.


Package natbib Warning: Citation
`bucknerOrganizationHumanCerebellum2011a' on p
age 15 undefined on input line 837.


Package natbib Warning: Citation `burgessEchoesMotorNetwork2017' on page
15 und
efined on input line 837.


Underfull \hbox (badness 2980) in paragraph at lines 837--838
[]\T1/Merriwthr-OsF/m/up/7.5 (+20) In sen-so-ri-mo-tor syn-chro-niza-
tion, the
au-di-tory sys-tem is
 []


Package natbib Warning: Citation `guellEmbodiedCognitionCerebellum2018'
on page
 15 undefined on input line 884.


Package natbib Warning: Citation `kawabataFunctionalConnectorHubs2022' on
page
15 undefined on input line 884.


Package natbib Warning: Citation `muellerOverweightObesityAre2012a' on
page 15
undefined on input line 886.


Package natbib Warning: Citation `obradovicLeptinObesityRole2021' on page
15 un
defined on input line 886.


Package natbib Warning: Citation `burgueraLongFormLeptin2000' on page 15
undefi
ned on input line 886.


Package natbib Warning: Citation `matochikEffectLeptinReplacement2005' on
page
15 undefined on input line 886.


Package natbib Warning: Citation `londonShortTermPlasticityGray2011' on
page 15
```

undefined on input line 886.

Package natbib Warning: Citation `baicyLeptinReplacementAlters2007' on page 15
undefined on input line 886.

Package natbib Warning: Citation `bermanEffectsLeptinDeficiency2013' on page 15
undefined on input line 886.

Package natbib Warning: Citation `fernandezAssessingCerebellarBrain2018' on pag
e 15 undefined on input line 888.

Package natbib Warning: Citation `mantoConsensusPaperNovel2022' on page 15 unde
fined on input line 888.

Package natbib Warning: Citation `oldratiTargetingHumanCerebellum2018' on page
15 undefined on input line 888.

Package natbib Warning: Citation `sebastianCerebellarNeuromodulationImproves202
0' on page 15 undefined on input line 888.

Package natbib Warning: Citation `iosifCerebellarPredictionFeeding2023' on page
15 undefined on input line 892.

Package natbib Warning: Citation `lowReversetranslationalIdentificationCerebell
ar2021' on page 15 undefined on input line 894.

Package natbib Warning: Citation `marronPrefrontocerebellarNeuromodulationAffec
ts2019' on page 15 undefined on input line 894.

Package natbib Warning: Citation `vangalenRoleCentralDopamine2018' on page 15 u
ndefined on input line 896.

```
Package natbib Warning: Citation `vangalenBrainResponsesNutrients2023' on
page
15 undefined on input line 896.


Package natbib Warning: Citation `vanderzwaalStriatalDopamineD22016' on
page 15
 undefined on input line 896.



[15]

Package natbib Warning: Citation `zhangBodyRoundnessIndex2024' on page 16
undef
ined on input line 902.


Package natbib Warning: Citation
`suligaUsefulnessAnthropometricIndices2019' on
 page 16 undefined on input line 902.


Package natbib Warning: Citation
`amirabdollahianAnthropometricIndicatorsAdipos
ity2018' on page 16 undefined on input line 902.


Package natbib Warning: Citation `huxleyBodyMassIndex2010' on page 16
undefined
 on input line 902.


Package natbib Warning: Citation `oneillMeasuringObesityAbsence2015' on
page 16
 undefined on input line 902.


Underfull \hbox (badness 1776) in paragraph at lines 908--909
\T1/Merriwthr-OsF/m/up/7.5 (+20) In or-der to ob-tain our re-sults, a
num-ber o
f methodological-
 []


Package natbib Warning: Citation
`bachmannCerebellocerebralConnectivityPredicts
2025' on page 16 undefined on input line 910.
```

Package natbib Warning: Citation `10.5555/1593511' on page 16 undefined on inpu
t line 925.

Package natbib Warning: Citation `rcoreteamLanguageEnvironmentStatistical2024'
on page 16 undefined on input line 925.

Package natbib Warning: Citation `hagbergExploringNetworkStructure2008' on page
16 undefined on input line 927.

Package natbib Warning: Citation `brett_2024_10714563' on page 16 undefined on
input line 927.

Package natbib Warning: Citation `abrahamMachineLearningNeuroimaging2014' on pa
ge 16 undefined on input line 927.

Package natbib Warning: Citation `the_pandas_development_team_2024_10957263' on
page 16 undefined on input line 927.

Package natbib Warning: Citation `raphael_vallat_2024_13683424' on page 16 unde
fined on input line 927.

Package natbib Warning: Citation `moritzRayDistributedFramework2018a' on page 1
6 undefined on input line 927.

Package natbib Warning: Citation `wickhamGgplot2ElegantGraphics2016' on page 16
undefined on input line 929.

Package natbib Warning: Citation `gtsummary' on page 16 undefined on input line
929.

Package natbib Warning: Citation `almendeb.v.andcontributorsVisNetworkNetworkVi
sualization2022' on page 16 undefined on input line 929.

```
Package natbib Warning: Citation
`bachmannSupportingDataCerebellocerebral2025'
on page 16 undefined on input line 935.


Underfull \hbox (badness 3989) in paragraph at lines 939--940
[]\T1/Merriwthr-OsF/m/up/7.5 (+20) Register for ac-cess:
[][]$\T1/lmtt/m/n/7.5
https : / / db . humanconnectome . org / app /
 []


Underfull \hbox (badness 1838) in paragraph at lines 940--942
[]\T1/Merriwthr-OsF/m/up/7.5 (+20) Access to re-stricted data (in-cludes
link t
o e-access ap-pli-
 []


Underfull \hbox (badness 10000) in paragraph at lines 940--942
\T1/Merriwthr-OsF/m/up/7.5 (+20) ca-tion form): [][]$\T1/lmtt/m/n/7.5
https : /
 / www . humanconnectome . org / study /
 []


Underfull \hbox (badness 10000) in paragraph at lines 942--943
[]\T1/Merriwthr-OsF/m/up/7.5 (+20) Data: WU-Minn HCP 1200 Sub-jects Data
Re-lea
se, for
 []


Underfull \hbox (badness 10000) in paragraph at lines 942--943
\T1/lmtt/m/n/7.5 humanconnectome . org / storage / app / media /
documentation
/
 []



[16]
No file main.bbl.

Package natbib Warning: There were undefined citations.




[17

]
```

```
enddocument/afterlastpage: lastpage setting LastPage.
(./main.aux)
 ***********
LaTeX2e <2024-06-01> patch level 2
L3 programming layer <2020/03/25>
 ***********

LaTeX Font Warning: Size substitutions with differences
(Font)              up to 1.0pt have occurred.


LaTeX Font Warning: Some font shapes were not available, defaults
substituted.

Package rerunfilecheck Info: File `main.out' has not changed.
(rerunfilecheck)             Checksum:
2CFAB35ECCCCBF7DEAF586183CE200DC;6268.
 )
Here is how much of TeX's memory you used:
 24808 strings out of 473583
 489399 string characters out of 5732343
 2019908 words of memory out of 5000000
 46515 multiletter control sequences out of 15000+600000
 2058761 words of font info for 653 fonts, out of 8000000 for 9000
 1141 hyphenation exceptions out of 8191
 123i,15n,131p,2116b,957s stack positions out of
10000i,1000n,20000p,200000b,200000s
<c:/texlive/2024/texmf-dist/fonts/type1/sorkin/merriweather/Merriwthr-
Bold.pf
b><c:/texlive/2024/texmf-dist/fonts/type1/sorkin/merriweather/Merriwthr-
BoldIta
lic.pfb><c:/texlive/2024/texmf-
dist/fonts/type1/sorkin/merriweather/Merriwthr-I
talic.pfb><c:/texlive/2024/texmf-
dist/fonts/type1/sorkin/merriweather/Merriwthr
-Regular.pfb><c:/texlive/2024/texmf-
dist/fonts/type1/public/amsfonts/cm/cmsy6.p
fb><c:/texlive/2024/texmf-
dist/fonts/type1/public/amsfonts/cm/cmsy7.pfb><c:/tex
live/2024/texmf-dist/fonts/type1/public/lm/lmtt8.pfb>
Output written on main.pdf (17 pages, 409111 bytes).
PDF statistics:
 348 PDF objects out of 1000 (max. 8388607)
 308 compressed objects within 4 object streams
 70 named destinations out of 1000 (max. 500000)
 234774 words of extra memory for PDF output out of 266212 (max.
10000000)
```

Manuscript

Click here to access/download;Manuscript;BMI_CCC_GigaScience-

Placeholder for OUP logo oup.pdf

GigaScience, 2025, 1–20

doi: xx.xxxx/xxxx
Manuscript in Preparation
Paper

PAPER

Cerebellocerebral Connectivity Predicts Body Mass Index: a New Open-Source Python-based Framework for Connectome-based Predictive Modeling

Tobias Bachmann[1,*], Karsten Mueller[2,3], Simon N. A. Kusnezow[4], Matthias L. Schroeter[2], Paolo Piaggi[5] and Christopher M. Weise[4]

[1]University of Leipzig Medical Center, Department of Neurology, Leipzig, Germany and [2]Max Planck Institute for Human Cognitive and Brain Sciences, Leipzig, Germany and [3]First Faculty of Medicine and General University Hospital in Prague, Department of Neurology, Prague, Czech Republic and [4]University of Halle Medical Center, Department of Neurology, Halle, Germany and [5]National Institutes of Health, Phoenix, Arizona, United States of America

*tobias.bachmann@medizin.uni-leipzig.de

ORCIDs: Tobias Bachmann [0000-0003-1796-6015]; Karsten Mueller [0000-0001-9613-0552]; Matthias Schroeter [0000-0001-7977-1083]; Paolo Piaggi [0000-0003-2774-9161]

Abstract

Background: The cerebellum is one of the major central-nervous structures consistently altered in obesity. Its role in higher-cognitive function, parts of which are affected by obesity, is mediated through projections to and from the cerebral cortex. We therefore investigated the relationship between body mass index (BMI) and cerebellocerebral connectivity. **Methods**: We utilized the Human Connectome Project's Young Adults dataset including functional MRI (fMRI) and behavioral data, to perform connectome-based predictive modeling (CPM) restricted to cerebellocerebral connectivity of resting-state fMRI and task-based fMRI. We developed a Python-based open-source framework to perform CPM, a data-driven technique with built-in cross validation to establish brain-behavior relationships. Significance was assessed with permutation analysis. **Results**: We found that 1. cerebellocerebral connectivity predicted BMI, 2. task-general cerebellocerebral connectivity predicted BMI more reliably than resting-state fMRI and individual task-based fMRI separetely, 3. predictive networks derived this way overlapped with established functional brain networks (namely frontoparietal networks, the somatomotor network, the salience network, and the default mode network), and 4. we found there was an inverse overlap between networks predictive of BMI and networks predictive of cognitive measures aversely affected by overweight/obesity. **Conclusions**: Our results suggest obesity-specific alterations in cerebellocerebral connectivity, specifically with regard to task execution. With brain areas and brain networks relevant to task performance implicated, these alterations seem to reflect a neurobiological substrate for task performance aversely affected by obesity.

Key words: Connectome-based predictive modeling; functional magnetic resonance imaging (fMRI); Python; Human Connectome Project (HCP); Cerebellum; BMI

Background

The prevalence of overweight has increased substantially over the last decades. Globally, around 2 billion people can be classified as

Compiled on: January 23, 2025.
Draft manuscript prepared by the author.

Key Points

- Cerebellocerebral connectivity predicts BMI
- Task-general cerebellocerebral connectivity most reliably predicts BMI.
- Predictive networks derived this way overlap with established functional brain networks.
- There is an inverse overlap between networks predictive of BMI and networks predictive of measures aversely affected by overweight/obesity (i.e. positive predictive networks overlapped with negative predictive networks and vice versa).

individuals with overweight or obesity as defined by a body mass index (BMI) of >= 25 or >= 30 kg/m^2, respectively [1, 2]. Being not only numerous, but a major risk factor of non-communicable disease, a considerable amount of research on these conditions exists. Etiological considerations try to conceptualize overweight and obesity as a result of a behavior not adapted to fairly new obesogenic environments [3]. From a neuroscientific perspective, behavior is a manifestation of brain activity, it therefore stands to reason that obesity is not only associated with a) specific behavioral characteristics, but also b) specific neuroanatomical characteristics.

Research on behavior associated with overweight, especially in the field of neuroimaging, has prominently been focusing on impulse control and reward processing [4, 5, 6, 7]. Studies have also consistently demonstrated impairments in executive function in individuals with overweight and, more pronounced, individuals with obesity [8, 9, 10].

Following these concepts, the majority of research on the neurobiological substrate of overweight has been concerned with cerebral cortical and subcortical regions thought to be involved in impulse control and affective regulation. Less light has been shed on the contribution of the cerebellum, even though it has consistently been demonstrated as functionally altered in individuals with overweight or obesity compared to individuals of non-pathological weight. In fact, recent metaanalyses count the cerebellum among three structures most robustly associated with obesity-related measurements [11, 12].

The cerebellum is an organized collection of a vast number of neurons commonly thought to be involved primarily in voluntary motor control. This model of cerebellar function dates back to the 19th century, when observations in animals and humans suffering from structural deficits (i.e. lesions or dys-/agenesis) of the cerebellum led to the formulation of a cerebellar syndrome with a set of core (motor) features, which is still very much in clinical use today: a combination of ataxia (dysmetria of the extremities and disturbance of balance and gait), dysarthria and oculomotor abnormalities (most notably nystagmus). (For a comprehensive overview of the history of the study of the cerebellum see [13]). In the late 20th and more emphatically in the 21st century, it has been, on the grounds of consolidating evidence, argued that the cerebellum does, in fact, play a role in a number of non-motor functions, i.e. higher-cortical functions like cognition, executive function, language, but also emotion, affect and behavior [14, 15, 16, 17, 18, 19, 20]. Of clinical importance, the corresponding symptoms of disturbances of non-motor cerebellar function constitute a complementary cerebellar syndrome: the Cerebellar Cognitive Affective Syndrome (CCAS).

Like the cerebellum's motor functions, these non-motor functions are thought to be put into effect through modulation of information of cerebral origin (for a comprehensive discussion, see section Discussion. This mechanism is reflected in the cerebellum's involvement in cerebral networks and in the existence of cerebellocerebral networks [21, 22, 23, 24] as its neurobiological basis. Functional data suggests that the majority of functional cerebellar units are indeed connected to non-motor areas of the cerebral cortex, i.e. association areas [25], while on a regional level domain-specific activation of cerebellar regions justifies extending the functional topography of the cerebellum to non-motor domains (for a comprehensive overview see [26, 27, 28]).

Therefore, we aimed to investigate BMI-dependent cerebellocerebral networks with a focus on their role in behavioural function. To do so, we applied connectome-based predictive modeling (CPM; for the principal paper see [29]), a protocol for establishing relationships between brain functional connectivity and neuroimaging-independent measures (e.g. anthropometric or behavioral measures) and offers several advantages in comparison to more common approaches (e.g. seed-based methods). Constitutive advantages of CPM are (a) being data-driven and (b) applying cross-validation. Regarding (a), no assumptions other than restricting connections as informed by our hypothesis (predictability of BMI by cerebellocerebral networks) were imposed on the data. By separating training and test datasets, cross-validation mitigates the problem of overfitting data: The performance of the model is evaluated on how well it performs on unseen data (for details, see Methods).

Taking into account the capabilities of CPM, our basic proposition of overweight- and obesity-specific behavioral alterations being mediated by cerebellar dysfunction can then be qualified. Thus, we hypothesized that cerebellocerebral connectivity, determined by resting-state and task-based functional magnetic resonance imaging (rsfMRI and tfMRI, respectively), is predictive of BMI in the context of CPM. We further hypothesized that tfMRI is more predictive of BMI than rsfMRI, since executive function and therefore task performance in seems to be more tangibly affected by overweight/obesity. As executive function is a general prerequisite for task execution, we speculated that tfMRI can task-independently be used to predict BMI. Finally, we evaluate if there is an overlap of predictive networks for BMI, as determined by CPM, with established functional brain networks and/or with predictive networks for cognitive and behavioral measure altered in overweight/obesity.

Data Description

Regarding imaging data, for both rsfMRI and tfMRI (see Methods for details) we used the Human Connectome Project's 1200 Subjects preprocessed release. The HCP developed what became known as the HCP-style approach to neuroimaging data, an acquisition and processing protocol based on a set of principles (”tenets”) guided by new insights gleaned from technical and analytical progress [30].

Raw high-resolution imaging data was acquired using a customized MRI scanner with anatomically and physiologically informed parameters, which for fMRI at 3T (the field strength we used) translates to a relatively fine-grained 2 mm isotropic spatial resolution. Multiband pulse sequences were used with a multiband factor of 8 and a short repetition time TR) of 0.72 s. Echo time (TE) equalled 33 ms, the flip angle (FA) was calculated at 52° to match the Ernst angle. 72 slices per brain were acquired with left-right and right-left phase encoding directions and an asymmetric acquisition matrix to help with distortion-related losses. Also notewor-

thy is the long overall acquisition time (e.g. approximately 1 hour of combined rsfMRI data for each subject).

To preserve as much signal as possible and make use of the high-quality raw data in downstream analyses, only minimal preprocessing was applied, notably restraining from unnecessary spatial smoothing and temporal filtering. While a comprehensive and detailed description has been published elsewhere [31], we provide a concise description of the steps involved. In a first step, correction for distortions related to gradient nonlinearity (which is more pronounced in the HCP's scanner setup) was applied with a FreeSurfer software package. The FSL software's FLIRT method was then used to correct for head motion. Grand-mean intensity normalization was performed on the fMRI time series.

One of the keystones of fMRI studies is reliable intersubject comparability, which requires translating a subject's physical space into a common standard space (”registering”). The HCP addressed this fundamental issue via multimodal registering, i.e. using a variety of imaging modalities to reliably and automatically identify anatomical or functional landmarks in each subject's 3D data and align them accordingly in what they call grayordinate space, a derivate of Montreal Neurological Institute (MNI) space, in which only matter of interest (i.e. gray matter) is preserved. Building on work described in [32], Glasser et al. [33] developed a multi-modal and mapped areal-feature-based (dubbed ”MSMAll”) registration method, which uses myelin maps, resting-state brain networks, visuotopic maps, and a subcortical region of interest for inter-subject alignment (see the supplemental methods of [33] for implementation details; for a discussion of the merits of multi-modal registration in the context of high-resolution imaging data and why reliable registration is paramount to neuroimaging studies, see [34]).

Using the specially developed CIFTI file format [35, 31], the cortex is represented as a 2D surface and subcortical structures as 3D volumes, reflecting inherent geographical properties of the dualist nature of human gray matter. This has profound practical consequences. Firstly, unfolding the cortical surface improves spatial localization by avoiding bleeding of signal into geographically close but functionally distinct regions of neighboring sulci. Secondly, surface-based methods, again, aid intersubject comparability by abstracting from intersubject variability of cortical folding patterns. For our own analysis, individual subject's CIFTI files registered as discussed above (i.e. via ”MSMAll”) constituted our starting point.

Methods

We opted for parcellated analysis as opposed to voxel-based analysis. Parcels consist of a collection of geographically and ideally functionally related voxels. As such, parcels not only save (computing) time and (memory) space and improve intersubject comparability and statistical sensitivity [34, 33]. They also represent functional integration hubs upon which brain function is built [36]. Trying to be as data-driven as feasible and being interested in functional connectivity, our parcels are functionally informed demarcations within the HCP data set. By combining three separately published parcellations, we were able to create a detailed whole-brain functional parcellation model covering the cerebral cortex, subcortical structures and the cerebellum. For the cerebral cortex we used the HCP-MMP1.0 (Human Connectome Project Multi-Modal Parcellation version 1.0, [33]) by Glasser et al. They delineated 180 parcels per hemisphere (360 in total) by using the overlap of four areal feature maps, one for each modality (cortical thickness, relative myelin content, tfMRI, rsfMRI). Subcortical parcels were provided by Tian et al. [37]. They relied on subcortical-to-cortical connectivity derived from the HCP's rsfMRI data to delineate 27 subcortical parcels per hemisphere (54 in total) along connectivity gradients, i.e. sufficiently stark changes in functional connectivity. Finally, cerebellar parcels came from a HCP-based study which clustered neighboring cerebellar voxels into 100 parcels by means of similarity of their rsfMRI time-series [38]. Combining the aforementioned parcellations gave us 513 parcels in total, see Figure 1. Our combined parcellation along with other auxiliary data and the entire code used in our study is publicly available (see below).

Using our parcels as nodes, we first extracted time series from the MSMall CIFTI files. For resting state, we used HCP Connectome Workbench software‘s command-line application to average timeseries per parcel. Supplying the individual parcels as ROIs, tfMRI was prepared with Python code based on the HCP pipelines script collection. With these time series, we calculated connectivity matrices for each subject using the Python package nilearn's ([39]; nilearn is based heavily on scikit-learn [40]] ConnectivityMeasure class (see bottom-left panel of Figure 1). For tfMRI, as opposed to more conventional partial correlation, we opted for tangent-space based connectivity matrices, which use a Riemannian manifold transformation [41, 42] as they were shown to be more sensitive to inter-subject differences. Comparing different processing methods for CPM, Dadi et al. found tangent-based parametrization and parcellations based on functional connectivity data to perform best [43]. For a more general comparison, which also resulted in a recommendation for tangent space, see the work of Pervaiz et al. [44]. We were able to confirm these reports by producing better predictions following these recommendations. Nilearn's implementation of Ledoit-Wolf's [45] shrinkage estimator was used as a regularization technique.

As we are interested in cerebellocerebral connections only, we purged our matrices of connections of non-interest (see bottom-right panel of Figure 1). Aside from limiting our connections of interest as informed by our hypothesis, no further anatomical assumptions were imposed on the data. These correlations-of-interest (COI) matrices (in the parlance of graph theory, which will be used later on, correlations represent ”edges” between ”nodes”, i.e. parcels) served as the basis for computing predictive networks in a CPM analysis.

Connectome-based predictive modeling (CPM)

Connectome-based predictive modeling (CPM) consists of several steps (see Figure 2): **(1)** Subjects are randomly divided in a “train” and a “test” population, with their respective sizes being determined by the number of folds k, i.e. pairs of train and test populations. For illustration purposes: An extreme number of folds (n-1) would correspond to the leave-one-out method, where, for each fold, a single subject constitutes the test population. We opted for k = 128, as a middle ground is reported to provide the most solid results (Kohavi, 1995), which is consistent with our own experience.

The train population's connectivity matrices are then used to **(2)** correlate their edges' weight, i.e. the strength of connections between nodes/parcels as represented, with the respective subject's variable of interest (e. g. BMI). At this stage, nuisance variables (gender, age, ethnicity) were regressed out. This yielded two separate networks, one of positively correlated edges, one of negatively correlated edges. To improve signal-to-noise ratio, only those edges passing a p threshold of 0.05 were put through to the next stage, which is **(3)** model building (note that p thresholding is just a means to select for “meaningful” edges and has nothing to do with statistical significance of our results, which is established later through permutation analysis). The HCP included a high number of twins (and subjects with other forms of close biological relation) in their study population. As both BMI [46, 47] and functional connectivity [48, 49, 50, 51, 52] are influenced by genetics, we took care to remove siblings from each fold before model building, thus ensuring that closely related subjects did not predict each other's BMI.

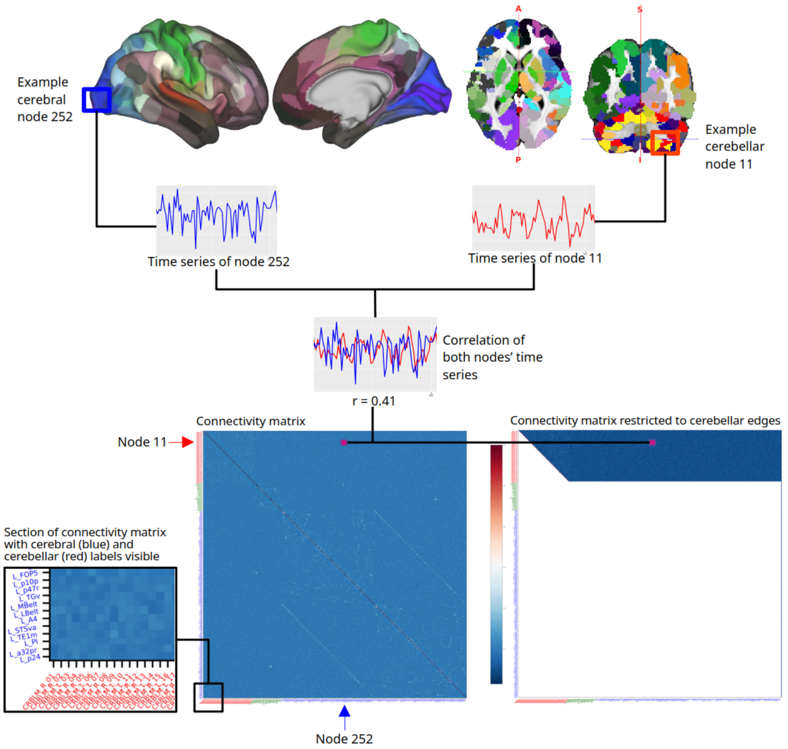

Figure 1. Flowchart depicting parcellated correlation analysis. The top row consists of our combined cerebrocortial-subcortical-cerebellar parcellation. A short time-series graph of exemplary nodes 11 and 247 are shown, the correlation of which, numerically represented by a value denoted with the letter r, makes up part of a connectivity matrix. The bottom-left panel shows an example connectivity matrix of a single subject. Cerebellar parcels are marked red, subcortical green and cortical blue. The bottom-right panel shows the same connectivity matrix reduced to connections of interest.

The positive and negative networks of the train population were fitted into linear models describing the relation of brain area connections to the brain-external measure (BMI) within that population; combining the negative and positive network models, a general linear model (GLM) was built. Finally, these models, trained on the train population, were used to (4) predict the test population's BMI. This process was repeated for k-fold times, so that all subjects were test subjects once and the entire population's BMI therefore predicted. Finally, Pearson's r for population-level correlation of predicted with observed values was calculated.

Task-based functional magnetic resonance imaging (tfMRI)

Besides task-free, i.e. resting-state fMRI (rsfMRI) data, the HCP offers task-based fMRI (tfMRI) session data. The tasks performed by subjects tap into different domains of cognitive and affective function. For a summary of tasks and their rationale see [53], concise explanations of the tasks are provided in the following paragraphs.

The emotion task contained two conditions: fear and neutral. Subjects were presented either pictures with fearful or angry faces (”fear” condition) or shapes (”neutral” condition) at the bottom of a screen and had to decide whether faces or shapes (respectively) at the top of the screen matched [53, 54].

The gambling task was designed to tap into incentive processing. Participants had to guess if the value of a card was less or more than five and would win one US dollar if correct and lose one if wrong [53, 55]. Accordingly, the gambling task was split into two conditions: loss and win.

The language task consisted of the ”story” condition testing semantic understanding and, for comparison, of the ”math” condition where participants had to solve arithmetic tasks [53, 56].

For the relational processing task, participants had to decide whether pairs of objects differed along the same dimensions (i.e. shape or texture) or if an object matched other objects with regard to a specified dimension; these different subtasks amounted to the ”relational” and ”match” condition, respectively [53, 57].

During the social cognition task, based on Frith-Happé animations designed to test participants' theory of mind, geometrical objects were displayed interacting (”mental” condition) or moving randomly (”random” condition) and participants had to decide whether movement of those objects represented social interaction [53, 58, 59].

The working-memory task is a variant of N-back tasks. Subjects were presented with pictures of faces, places, body parts and tools in the 0-back and 2-back fashion [53]. As we were more interested in working memory as an executive-function subdomain than in the localizing function of different picture categories [60], we combined all 0-back runs and all 2-back runs into two respective conditions.

To address our hypothesis of task-indendepent predictibility of BMI, we proceeded to (a) average connectivity matrices of task conditions resulting in a single connectivity matrix per task[1], and (b) to finally combine and average all tasks (i.e. adding all task-specific connectivity matrices per subject and dividing the resulting cumulative matrices by the number of tasks).

Statistical significance

To assess statistical significance of our results, i.e. the correlation of BMI as predicted by our GLM with observed BMI, whilst avoiding the pitfalls of parametric testing [61], we used permutation analysis, which consisted of repeatedly performing CPM after permuting BMI within the test population (if there is no true correla-

1 Averaging was done per subject, i.e. the connectivity matrix serving as input for CPM for subject X was calculated by averaging the connectivity matrices for e.g. the “loss” and “win” condition of the gambling task of that subject.

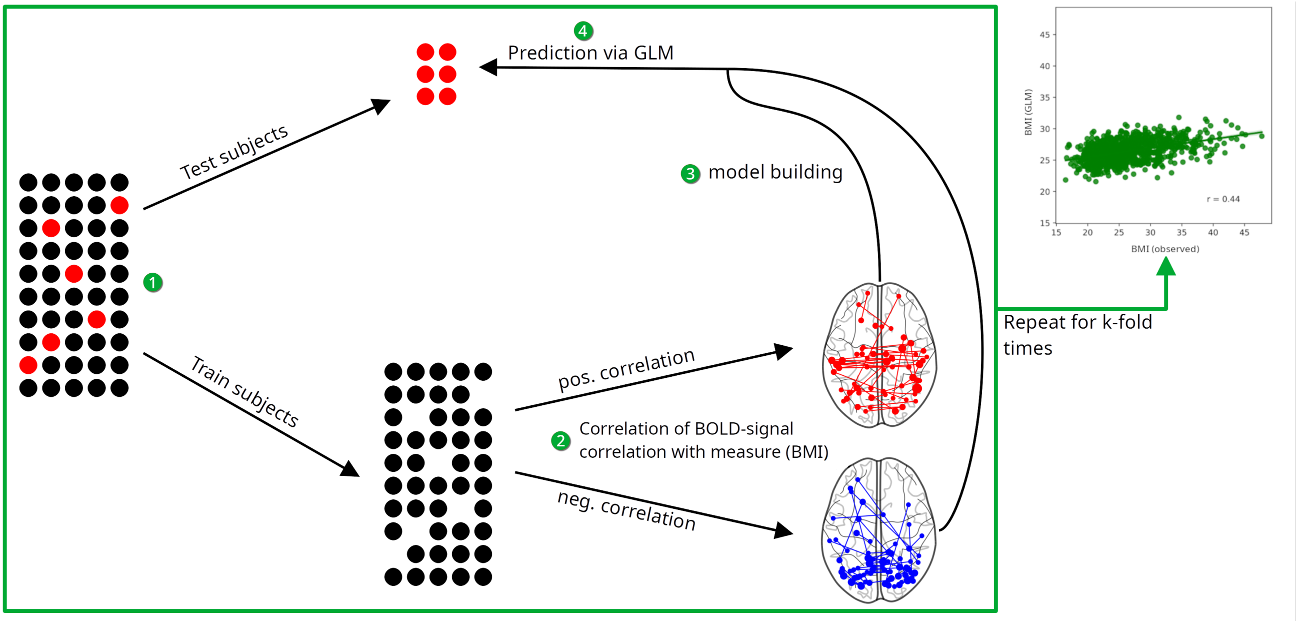

Figure 2. Flowchart depicting the steps involved in Connectome-based Predictive Modeling. *GLM*: General linear model; *BOLD*: blood-oxygen-level dependent

tion between BMI and cerebellocerebral connections, analysis after permuted BMI should yield no relevant results). The number of permutations was chosen as to establish significance at $p \leq 0.001$ (p equals the proportion of permutations equal or greater than our true prediction) after correcting for multiple comparisons[2]. For a graphical representation of the results see supplemental figure S13. Permutation analysis is a powerful, albeit computationally expensive method. As a means to deal with computational load, our analysis code is able to make use of parallel and distributed (as in multi-machine) computing, to which end we rely on the Ray framework [62] in its Python-based incarnation.

Overlap with networks predictive of related measures

To further explore our theory of a predictive-network-determining relationship of cerebellar non-motor function and BMI's negative association with the latter, we ventured to analyze the overlap between networks predictive of BMI on one hand and networks predictive of other measures on the other hand. We first calculated Pearson's r for BMI and measures of interest, i.e. measures of executive function (Wisconsin Card Sorting and Eriksen flanker task), general cognition (Penn Matrix Reasoning Test), and reward-related self-regulation [delay discounting; for a description of these measures, their acquisition and usage in context of the HCP, see [53, 63].

For exploratory purposes, we then performed CPM (with averaged tfMRI, as it yielded the most promising results in our primary analysis) on measures of interest, i.e. measures of executive function (Wisconsin Card Sorting and Eriksen flanker task), general cognition (Penn Matrix Reasoning Test), and reward-related self-regulation (delay discounting; for a description of these measures, their acquisition and usage in context of the HCP, see [53, 63]). We finally compared the resulting predictive networks with those of our primary analysis by multiplying masks of connectivity matrices of significant edges thus creating a connectivity matrix describing an overlap network. Ranking of nodes was based on their weighted degrees averaged over overlapping networks (see supplemental section S2 for details).

2 We took a conservative approach with 10000 permutations for the task-based analysis and 2000 permutations for the resting-state based analysis, which satisfies conservative correction methods like Bonferroni's.

Results

Population

Characteristics of our study population are summarized in Table 1. We only included subjects for whom all needed data was available, which included BMI for all subjects and respective neuroimaging data for each of the fMRI modalities. Therefore, the number of subjects differed between fMRI modalities and ranged from 999 (resting-state fMRI) to 1077 (gambling task). The number of subjects with complete data for all tasks, which could thus be included in our combined task analysis (see below) was 999. Demographic variables were very similar between groups, they all contained more female than male participants and were predominantly white (~ 75 %). Based on BMI, more than half of our subjects were individuals with overweight or obesity, a quarter were in the normal weight range and a small minority (~ 1,5 %) were individuals with underweight. Median BMI was in the lower overweight range.

Resting-state functional connectivity of parcels

Taking rsfMRI time-series from our whole-brain parcellation as a starting point and using visNetwork [64], an R [65] package, we are able to provide an interactive overview of functional connectivity between parcels (see Figure 3 for illustration; the interactive web page is available in GigaDB (see section *Data availability*) – note that no anatomical information was supplied to the algorithm seemingly "grouping" cerebral, cerebellar and subcortical parcels; the geographic proximity of the respective group's parcels to each other is solely a function of their greater interconnectedness).

CPM

Our primary measure was Pearson's r describing the relationship between observed (i.e. recorded by the HCP) and predicted (by our GLM) BMI. While this correlation is the most direct measure of predictive success, it is not informative with regard to the neu-

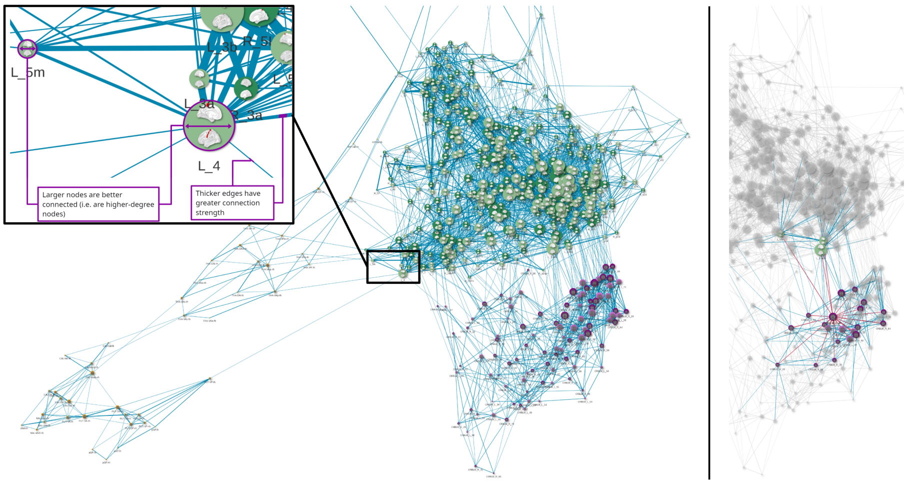

Figure 3. Screenshots of an interactive web-browser based visualization of resting-state functional MRI connectivity between all parcels; cerebral parcels are colored green, cerebellar parcels violet and subcortical parcels orange (brighter and darker hues denote left and right hemispheres, respectively). The right panel shows primary (red) and secondary (blue) connections of one selected cerebellar parcel.

robiological substrate of brain-behavior relationships. Of neuroscientific interest are specific predictive networks and the nodes and edges they consist of. (a) For visual inspection, we plotted networks on a schematic brain (see results figures) and provide interactive web-based plots for our main results (available in GigaDB, see section *Data availability*). (b) We plotted the most powerfully predictive edges one by one. (c) To reach a better understanding of cerebral regions involved, we sorted cerebral nodes according to their relevance in the respective networks; our concept of relevance here makes use of the graph-theoretical notion of a weighted degree, i.e. the sum of the weights of all edges a node is connected by. We finally compared these weightiest nodes to established brain networks.

The following presentation of our results is of a descriptive nature, we explore functional implications in the *Discussion* section.

rsfMRI-based CPM

Performing CPM with rsfMRI data yielded a modest correlation of observed with predicted BMI (r = 0.44; results were statistically significant, $p \leq 0.001$, see supplemental figure S13). Intracerebellar connections dominated both negative and positive networks, the former more than the latter. The positive predictive network's highest-degree nodes notably included bitemporal nodes (temporal poles and ventromedial areas of the temporal poles), the right insular cortex and the left cingulate cortex as well as biprefrontal and biparietal areas (see Figure 4). While the left V1 reached top spot in the negative predictive networks' list of best connected nodes, which also featured as the only subcortical region a thalamic subdivision.

As an interesting side note, whole-brain rsfMRI CPM, as opposed to rsfMRI CPM restricted to cerebellocerebral and cerebellocerebellar connections, performed only slightly better in predicting subjects' BMI (r = 0.44 vs. 0.40; see A. vs B. in Figure 4).

tfMRI-based CPM

tfMRI sessions consisted of several conditions per task group (e.g. zero-back and two-back conditions for the working memory task). Designed by the HCP for this purpose, we tried to utilize these constellations of conditions by subtracting connectivity matrices of more-general condition (e.g. zero-back) from more-specific conditions (e.g. two-back) to allow for capturing task-specific activations as opposed to overarching task-general activations. Besides two-back vs. zero-back conditions with working memory, we analysed story vs. math conditions in language, relational vs. match conditions in the relational task and the theory-of-mind condition vs. random conditions in the social task. Interestingly, predicted-observed correlations with task contrasts were generally low and not significant.

While specific predictive networks for individual tasks could be identified, correlation was thus better the more general the task data was prepared. Task groups (i.e. averaged task conditions) yielded markedly better results than individual tasks and task contrasts, while task-general activation (i.e. all task conditions averaged) outperformed both in predicting BMI (r = 0.70, $p \leq 0.001$; see Figure 5). A rather detailled description of individual task results and their respective plots can be found in supplementary section S1. In the following section we will concentrate on, i.e. plot, list, and discuss, the most relevant 5 % (by weighted degree) of cerebral nodes of the task-general predictive networks; the number of cerebellar nodes to concentrate on was decided by visual determination of a degree threshold via inspection of their degree distribution (see the bar plots in supplemental figure S7).

The *positive* predictive network was clearly dominated by ipsilateral and contralateral symmetric cerebellar projections to a just a few temporopolar nodes, namely (adopting the names provided by Glasser et al. (2016)) 1. dorsal and 2. ventral Area TG and 3. perirhinal cortex (see Table 2 for further details). Cerebellar nodes involved in the positive predictive network were also symmetrically located in the lateral posterior hemispheres, where they tended to be more ventrally located; only one intercerebellar edge was among the top 100 (ordered by edge strength), but a number of intracerebellar edges within the respective lateral posterior cerebellar hemispheres did (also symmetrical). The five most predictive cerebellocerebral edges connected left cerebellar node 86, its contralateral counterpart node 49 and adjacent node 4 to contralateral temporal nodes. Interestingly, these cerebellar nodes were all located in crus VIIb (see section *Discussion* below).

The *negative* predictive network, on the other hand, proved to be less concentrated and dominated by just a few nodes. Contralateral edges prevailed (not a single ipsilateral edge passed the threshold) with the right cerebellum contributing weightier (by weighted degree) nodes. Interestingly, the edge with the most predictive power connected a left cerebellar node to a subdivision of the right putamen. Other than that, the weightiest edges connected right cerebellar nodes with temporal nodes (but excluding the poles and including bilateral auditory areas 4), followed by parietal nodes and intercerebellar connections, which were far more numerous than in the positive predictive network (see Table 3 for further details). It is worth noting that the cerebellar constituents of the most predictive edges were again located in crus

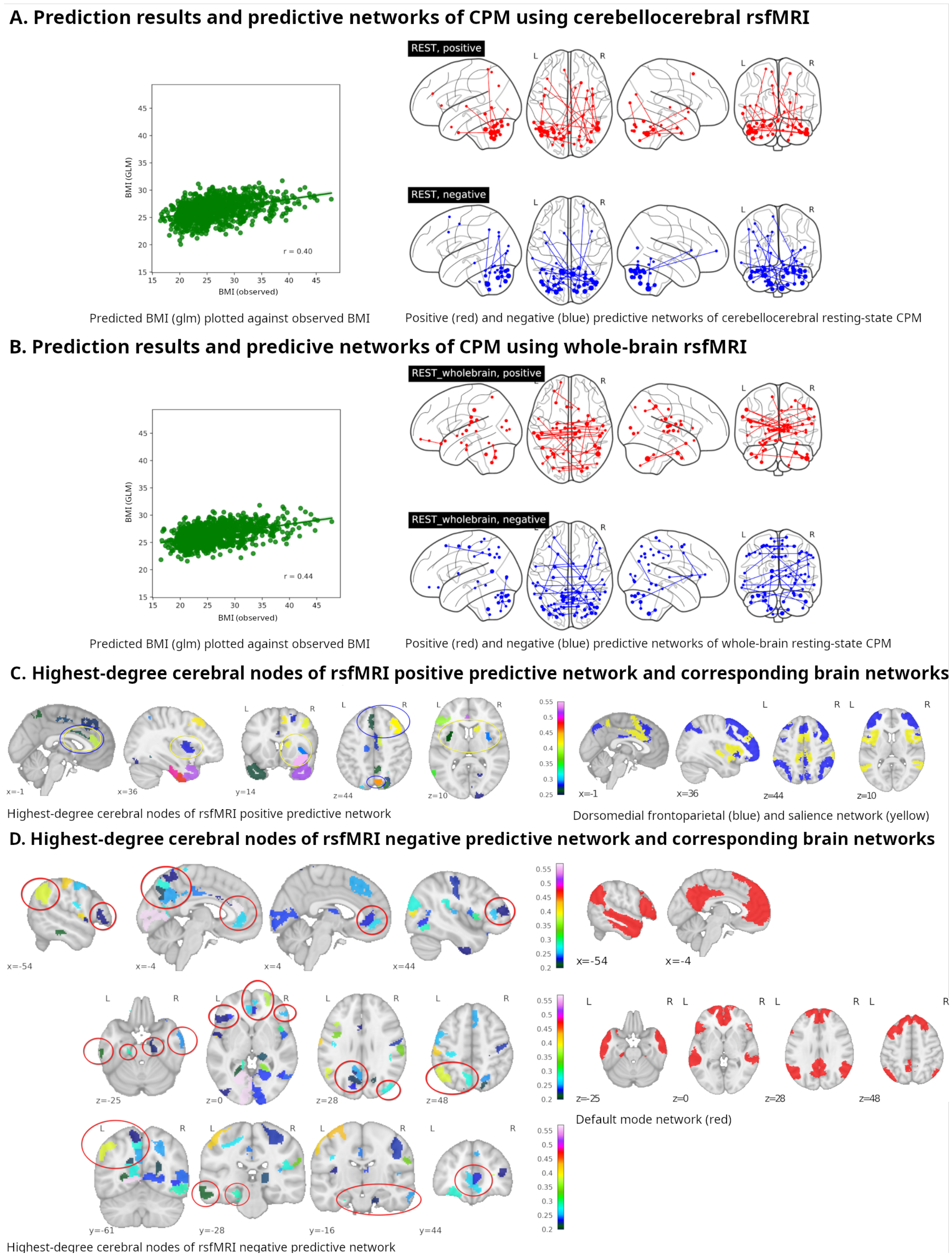

Figure 4. **A. and B.** Left panel: Plot of observed body mass index vs. predicted body mass index (p for Pearson's $r \leq 0.001$). Right panel: Plots of positive and negative predictive networks onto a glass brain. **C.** Left panel: Slices showing cerebral nodes of the resting-state *positive* predictive network with nodes participating in the dorsomedial frontoparietal network (dmFPN) and salience network (SN) encircled in blue and yellow, respectively. Right panel: Plots of established brain networks associated with our resting-state *positive* predictive network, slices at strategic locations show networks 6 and 4 of (Thomas Yeo et al. 2011)'s 7 network parcellation[a] (corresponding to the dmFPN, blue, and the SN, yellow). Coordinates are MNI coordinates. Colors and numbers on colorbars indicate weighted degrees. **D.** Left panel: Slices showing cerebral nodes of our resting-state *negative* predictive network. Coordinates are MNI coordinates. Colors and numbers on colorbars indicate weighted degrees. Right panel: Slices plotting network 7 of (Thomas Yeo et al. 2011)'s 7 network parcellation[a], which corresponds to the default mode network associated with our resting-state *negative* predictive network. *BMI*: Body mass index; *CPM*: Connectome-based predictive modeling; *L*: left; *R*: right; *rsfMRI*: resting-state functional magnetic resonance imaging.

a For the established brain networks, data were downloaded from `https://surfer.nmr.mgh.harvard.edu/fswiki/CorticalParcellation_Yeo2011` and plotted with nilearn (filename was `Yeo2011_17Networks_MNI152_FreeSurferConformed1mm_LiberalMask.nii.gz` for the 17 network parcellation and `Yeo2011_7Networks_MNI152_FreeSurferConformed1mm_LiberalMask.nii.gz` for the 7 network parcellation, both contained in `Yeo_JNeurophysiol11_MNI152.zip`.)

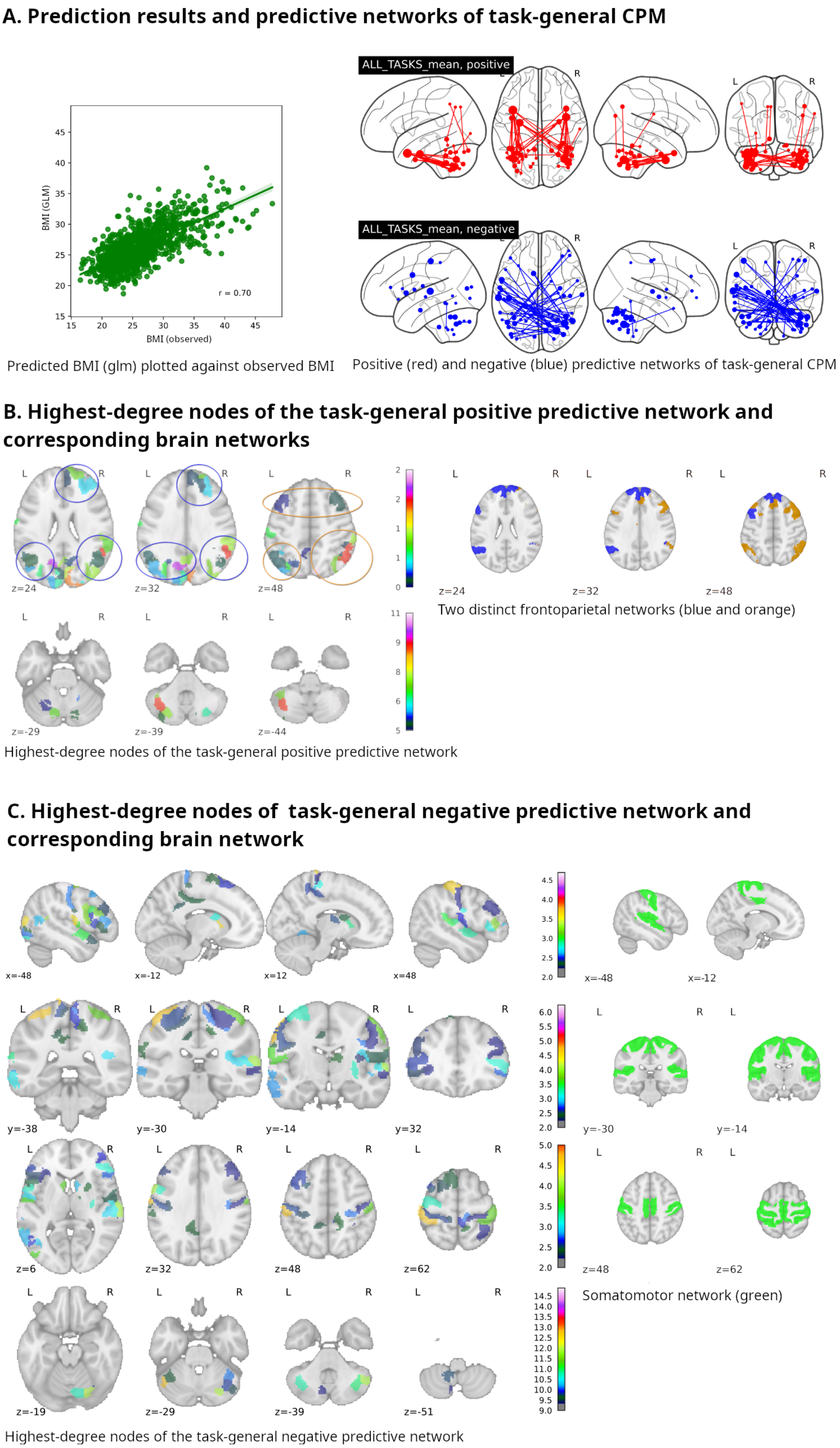

Figure 5. A. Left panel: Plot of observed body mass index vs. predicted body mass index (p for Pearson's r $\leq$ 0.001). Right panel: Plots of positive and negative predictive networks onto a glass brain. **B.** Left panel: Slices showing cerebral (top panel) and cerebellar (bottom panel) nodes of the task-general *positive* predictive network involved, among other things, in two established frontoparietal networks (encircled in blue and orange, respectively). Coordinates are MNI coordinates. Colors and numbers on colorbars indicate weighted degrees. Note that preferential distribution in the right cerebral hemisphere corresponds to a preferential distribution in the left cerebellar hemisphere. Right panel: Plots of aforementioned established frontoparietal networks associated with our task-general *positive* predictive network, slices at strategic locations show networks 13 and 17 of (Thomas Yeo et al. 2011)'s 17 network parcellation[a]. **C.** Left panel: Slices showing cerebral (top three panels) and cerebellar nodes (bottom panel) of the task-general *negative* predictive network. Coordinates are MNI coordinates. Colors and numbers on colorbars indicate weighted degrees. Right panel: Slices plotting the established somatomotor network (SMN) associated with our task-general *negative* predictive network at strategic locations, plotted with data from (Thomas Yeo et al. 2011)'s 7 network parcellation, where the SMN corresponds to network 2[a]. *BMI*: Body mass index; *CPM*: Connectome-based predictive modeling; *L*: left; *R*: right

Table 1. Table of summary population statistics.

**Characteristic**	Resting-state fMRI	Task-based fMRI					
	**rsfMRI**, n = 999[1]	**Emotion**, n = 1041[1]	**Gambling**, n = 1077[1]	**Language**, n = 1007[1]	**Relational**, n = 1034[1]	**Social**, n = 1042[1]	**WM**, n = 1074[1]
Age	29 (26, 32)	29 (26, 32)	29 (26, 32)	29 (26, 32)	29 (26, 32)	29 (26, 32)	29 (26, 32)
Gender							
Female	532 (53%)	558 (54%)	581 (54%)	540 (54%)	553 (53%)	559 (54%)	582 (54%)
Male	467 (47%)	483 (46%)	496 (46%)	467 (46%)	481 (47%)	483 (46%)	492 (46%)
Ethnicity							
Indigenous	2 (0.2%)	2 (0.2%)	2 (0.2%)	2 (0.2%)	2 (0.2%)	2 (0.2%)	2 (0.2%)
Asian/Pacific	63 (6.3%)	64 (6.1%)	64 (5.9%)	61 (6.1%)	64 (6.2%)	64 (6.1%)	64 (6.0%)
Black	139 (14%)	145 (14%)	155 (14%)	141 (14%)	144 (14%)	145 (14%)	154 (14%)
More than one	24 (2.4%)	24 (2.3%)	29 (2.7%)	23 (2.3%)	23 (2.2%)	23 (2.2%)	29 (2.7%)
Unknown	17 (1.7%)	16 (1.5%)	18 (1.7%)	13 (1.3%)	16 (1.5%)	16 (1.5%)	18 (1.7%)
White	754 (75%)	790 (76%)	809 (75%)	767 (76%)	785 (76%)	792 (76%)	807 (75%)
BMI	25.4 (22.8, 29.1)	25.4 (22.8, 29.1)	25.5 (22.8, 29.2)	25.4 (22.8, 29.2)	25.4 (22.8, 29.2)	25.4 (22.8, 29.1)	25.5 (22.9, 29.2)
Weight group							
Normal weight	426 (43%)	447 (43%)	454 (42%)	434 (43%)	442 (43%)	448 (43%)	450 (42%)
Obesity	233 (23%)	241 (23%)	255 (24%)	230 (23%)	242 (23%)	240 (23%)	256 (24%)
Overweight	326 (33%)	337 (32%)	351 (33%)	327 (32%)	334 (32%)	338 (32%)	351 (33%)
Underweight	14 (1.4%)	16 (1.5%)	17 (1.6%)	16 (1.6%)	16 (1.5%)	16 (1.5%)	17 (1.6%)

[1]Median (IQR); n (%)

WM: working memory. *BMI*: Body mass index. *fMRI*: Functional magnetic resonance imaging. *IQR*: Interquartile range. *rsfMRI*: Resting-state functional magnetic resonance imaging.

VII (left hemisphere: node 99, right hemisphere: nodes 20, 24, 70, and 96).

Statistical significance was assessed with permutation testing, for a graphical representation see supplemental figures S13.

Overlap analysis

As expected, BMI was negatively associated with measures of executive and cognitive function, with Penn Matrix Reasoning Test showing the strongest negative association and the flanker task the least (the latter is also the only measure where statistical significance was not reached; see Figure 6 for details).

Crucially, a significant overlap between networks predictive for BMI and other measures of interest was only observed when comparing positive predictive networks for BMI with negative predictive networks of the other measures (and vice versa, see Figure 6 for an example). This is in line with our theory, which predicts this kind of inverse relationship. An alternative explanation would be that this observation is simply a consequence of the inverse relationship of BMI with the other measures. However, correlations between BMI and the other measures are weak and significant only due to the large number of subjects, though. We find it therefore more plausible to attribute the inverse overlap to the predictive networks being functionally distinct. Consequently, positive predictive networks for BMI overlapping with negative predictive networks of the other measures had high-ranking frontoparietal nodes reminiscent of frontoparietal executive networks and negative predictive networks for BMI overlapping with positive predictive networks of the other measures tended to have high-degree somatomotor cortex nodes.

Discussion

Using CPM, we aimed to investigate whether and to which extent cerebellocerebral connectivity predicts BMI. Both tfMRI and rsfMRI yielded networks positively and negatively predictive of BMI. In the following sections, we will provide an in-depth discussion and interpretation of our results, i.e. of important nodes within the networks and of the networks as networks. We will start with the rsfMRI-based positive and negative predictive networks, followed by the tfMRI-based positive and negative predictive networks.

Resting-state CPM

CPM with cerebellocerebral connections based on rsfMRI data as opposed to tfMRI yielded markedly worse predictions of BMI. It is interesting to note, though, that whole-brain CPM with rsfMRI data performed not much better (Pearson's r = 0.40 vs. 0.44, respectively), which confirms the cerebellum's prominence with respect to obesity-related brain changes noted in literature.

We will first discuss cerebellar nodes, then move on to discussing cerebral nodes of the positive and negative predictive network separately, where we will again focus on established brain networks that were recognizable in our results.

While in the case of tfMRI, cerebellocerebral edges made up the vast majority of most predictive edges both in the positive and negative predictive networks with only a few intercerebellar connections making the list, this ratio was reversed for rsfMRI. On the other hand, cerebellar nodes in the rsfMRI predictive networks were geographically constrained to a much higher degree with most nodes being directly adjacent to each other (see image B in Figure 7).

Table 2. List of top 30 (by weighted degree) nodes in the positive predictive network for all tasks combined (averaged). Area names denote either Brodmann areas (numerical) or follow von Economo and Kosinas' letter system (for a concise overview see [66]). Cerebral divisions for cortical parcels by Glasser et al. [33]'s parcellation are based on its Neuroanatomical Supplementary Results as compiled by [67].

Node	Degree	Label	Name	Cerebral division	MNI coordinates
325	4.99839	R_TGv	Right Area TG Ventral	Lateral Temporal	39.81 -0.81 -42.01
455	4.9941	L_PeEc	Left Perirhinal Ectorhinal Cortex	Medial Temporal	-29.14 -9.22 -32.93
464	4.81841	L_TGd	Left Area TG dorsal	Lateral Temporal	-39.32 9.55 -31.94
275	4.31887	R_PeEc	Right Perirhinal Ectorhinal Cortex	Medial Temporal	29.4 -7.66 -33.54
505	3.90656	L_TGv	Left Area TG Ventral	Lateral Temporal	-41.2 -2.21 -41.38
284	3.00586	R_TGd	Right Area TG dorsal	Lateral Temporal	39.95 11.8 -31.73
304	2.63506	R_PGs	Right Area PGs	Inferior Parietal	45.35 -64.99 37.45
168	2.59669	R_POS2	Right Parieto-Occipital Sulcus Area 2	Posterior Cingulate	12.41 -68.35 38.55
348	2.32532	L_POS2	Left Parieto-Occipital Sulcus Area 2	Posterior Cingulate	-9.23 -69.57 36.83
302	2.17232	R_PFm	Right Area PFm Complex	Inferior Parietal	51.34 -47.96 40.5
158	2.06219	R_V3	Right Third Visual Area	Early Visual	20.69 -86.43 8.49
465	2.05809	L_TE1a	Left Area TE1 anterior	Lateral Temporal	-58.78 -9.83 -20.57
451	2.00351	L_EC	Left Entorhinal Cortex	Medial Temporal	-21.06 -14.52 -28.18
466	1.89598	L_TE1p	Left Area TE1 posterior	Lateral Temporal	-58.29 -46.77 -9.99
496	1.77366	L_VVC	Left Ventral Visual Complex	Ventral Stream Visual	-30.93 -52.31 -16.89
157	1.75576	R_V2	Right Second Visual Area	Early Visual	13.04 -78.5 5.59
225	1.72395	R_10d	Right Area 10d	Orbital and Polar Frontal	10.29 64.82 6.67
363	1.71058	L_7m	Left Area 7m	Posterior Cingulate	-4.57 -61.87 36.73
301	1.69895	R_PF	Right Area PF Complex	Inferior Parietal	58.54 -31.42 35.7
271	1.69591	R_EC	Right Entorhinal Cortex	Medial Temporal	21.61 -14.02 -27.89
296	1.69274	R_PGp	Right Area PGp	Inferior Parietal	42.77 -75.44 23.8
299	1.6117	R_IP0	Right Area IntraParietal 0	Inferior Parietal	34.4 -72.07 30.14
183	1.58747	R_7m	Right Area 7m	Posterior Cingulate	5.57 -61.75 36.66
298	1.56293	R_IP1	Right Area IntraParietal 1	Inferior Parietal	35.85 -62.69 42.61
508	1.55809	L_A4	Left Auditory 4 Complex	Auditory Association	-60.62 -24.47 7.55
240	1.54291	R_9a	Right Area 9 anterior	Dorsolateral Prefrontal	17.26 59.18 21.32
288	1.52408	R_TF	Right Area TF	Medial Temporal	42.23 -21.44 -27.25
178	1.51844	R_PSL	Right PeriSylvian Language Area	Temporo-Parieto-Occipital Junction	60.55 -37.3 24.57
478	1.46096	L_IP1	Left Area IntraParietal 1	Inferior Parietal	-30.49 -65.54 42.52
287	1.44772	R_TE2a	Right Area TE2 anterior	Lateral Temporal	55.13 -18.49 -27.3

MNI: Montreal Neurological Institute

Resting-state positive predictive network

The rsfMRI positive predictive network's best-connected cerebral nodes contained the dorsal-anterior portion of the right insula and the left anterior cingulate cortex (see Figure 4), corresponding to key nodes of the salience network (for an overview see [69]. Also included was the left caudate nucleus, which belongs to subcortical areas associated with the salience network. Interestingly, evidence in psychiatric disease implicates the caudate nucleus and its contribution to the SN in (impaired) cognitive flexibility and inhibitory control [70, 71], which, along with functional connectivity of the caudate nucleus, tends to be also impaired in individuals with obesity [72, 73].

Apart from the salience network, the rsfMRI positive predictive network's cerebral nodes are similar to the tfMRI one's, which is most conspicuous in the prominence of the temporal poles and ventromedial temporal lobes. Less pronounced, but nonetheless interesting is another executive network partially overlapping with the FPN (aka "central executive network") discussed above. Distinct from that network is the emergence of the anterior to middle cingulate cortex and the medial superior parietal cortex as hubs (the network we refer to here corresponds most closely to the green cluster [74] proposed the label "dorsomedial frontoparietal network", dM-FPN, for). Being part of the executive network family, this network too is implicated in EF.

Resting-state negative predictive network

Within the cerebral faction of the rsfMRI negative predictive network, we found a pattern of nodes reflecting virtually all key clusters of the default mode network (for an overview of the default mode network see [75]): bilateral orbital frontal cortices, medial prefrontal and adjacent anterior cingulate cortices, lateral temporal cortices, inferior parietal cortices, posterior cingulate, and parahippocampal cortices (see Figure4). Most famous for being active in rest and being downregulated when a task is performed [76], the default mode network has been implicated in various conditions. Of relevance to the individual-level neuroscientific view on the obesity complex, a major study on the topic of impulsitivity has shown the default mode network's cocorrelation with motor planning areas to be associated with impulsivity in juvenile offenders and developing youth [77].

Moving away from networks, one of the notable nodes being well-connected in the rsfMRI negative predictive network is the auditory cortex. We already discussed the auditory cortex above as a multimodal integration hub on occasion of its appearance in the tfMRI negative predictive network.

Task-based CPM

After averaging connectivity matrices for different task conditions, CPM yielded generally good results, differing only marginally in their range from r = 0.59 (emotion task) over r = 0.61 (relational task) to r = 0.62 (language, social and working-memory task). Improving prediction by averaging instead of just appending condition timeseries (thus keeping the number of data points the same while leveling out task-specific correlations) suggests that there are indeed task-general networks at play.

The discussion of the results of task-based CPM will therefore focus on averaged task data ("task general"). We will first discuss general aspects of our results, highlighting specific cerebellar nodes, whose connections were found to be most predictive of BMI and continue with a per-network discussion with an emphasis on

Table 3. List of top 30 (by weighted degree) nodes in the negative predictive network for all tasks combined (averaged). Area names denote either Brodmann areas (numerical) or follow von Economo and Kosinas' letter system (for a concise overview see Triarhou 2007).

Node	Degree	Label	Name	Cerebral division	MNI coordinates
508	6.24397	L_A4	Left Auditory 4 Complex	Auditory Association	-60.62 -24.47 7.55
384	4.66822	L_1	Left Area 1	Somatosensory and Motor	-44.99 -25.26 52.28
389	4.09919	L_6v	Left Ventral Area 6	Premotor	-55.79 1.93 31.74
328	4.00675	R_A4	Right Auditory 4 Complex	Auditory Association	63.44 -20.25 7.24
204	3.65353	R_1	Right Area 1	Somatosensory and Motor	45.89 -22.03 52.67
143	3.61732	CAU-VA-lh	Left ventroanterior caudate	Caudate nucleus	-8.49 11.35 4.96
492	3.58627	L_LO3	Left Area Lateral Occipital 3	MT+ Complex and Neighboring Visual Areas	-42.01 -79.06 11.72
433	3.57763	L_OP4	Left AreaOP4/PV	Posterior Opercular	-55.9 -13.38 15.43
361	3.32351	L_STV	Left Superior Temporal Visual Area	Temporo-Parieto-Occipital Junction	-57.46 -47.33 17.28
387	3.19566	L_6d	Left Dorsal area 6	Premotor	-32.02 -13.25 62.94
411	3.19286	L_6r	Left Rostral Area 6	Premotor	-50.68 7.0 18.08
114	3.17489	PUT-VP-rh	Right ventroposterior putamen	Putamen	30.73 -10.67 -0.64
235	3.14016	R_IFSa	Right Area IFSa	Inferior Frontal	45.2 38.3 8.08
414	3.10309	L_IFSp	Left Area IFSp	Inferior Frontal	-43.96 22.44 21.72
277	3.05388	R_PBelt	Right ParaBelt Complex	Early Auditory	58.2 -19.32 8.4
466	3.04505	L_TE1p	Left Area TE1 posterior	Lateral Temporal	-58.29 -46.77 -9.99
432	3.00455	L_43	Left Area 43	Posterior Opercular	-56.12 -1.03 9.93
255	2.97411	R_OP2-3	Right AreaOP2-3/VS	Posterior Opercular	38.28 -16.01 18.61
257	2.96739	R_RI	Right RetroInsular Cortex	Early Auditory	43.04 -29.43 17.69
440	2.96613	L_TA2	Left Area TA2	Auditory Association	-51.57 0.9 -4.87
412	2.96198	L_IFJa	Left Area IFJa	Inferior Frontal	-42.34 13.25 25.61
327	2.95945	R_LBelt	Right Lateral Belt Complex	Early Auditory	50.27 -24.46 10.32
177	2.89554	R_A1	Right Primary Auditory Cortex	Early Auditory	44.82 -21.49 9.69
335	2.88416	L_MST	Left Medial Superior Temporal Area	MT+ Complex and Neighboring Visual Areas	-43.71 -68.77 7.23
116	2.87957	CAU-VA-rh	Right ventroanterior caudate	Caudate nucleus	9.87 11.89 5.04
260	2.84924	R_TA2	Right Area TA2	Auditory Association	51.39 1.0 -5.42
470	2.83976	L_PHT	Left Area PHT	Lateral Temporal	-55.16 -57.57 0.6
458	2.81946	L_A5	Left Auditory 5 Complex	Auditory Association	-59.57 -17.43 -0.75
465	2.79938	L_TE1a	Left Area TE1 anterior	Lateral Temporal	-58.78 -9.83 -20.57
355	2.77208	L_PIT	Left Posterior InferoTemporal Complex	Ventral Stream Visual	-38.25 -81.59 -11.24

IFJ: Inferior frontal junction; *IFS*: Inferior frontal sulcus; *MNI*: Montreal Neurological Institute

cerebral nodes with and their participation in cerebral networks.

While we discovered distinct networks in each single-task CPM, when considering the analysis of combined tasks, a pattern was vindicated which had been indicated in the individual task analyses: The negative predictive network was considerably stronger overall (with regard to edge strength and weighted degree), with its cerebellocerebral and cerebellocerebellar connections overwhelmingly connecting contralateral nodes. In contrast, the positive predictive network had more and stronger ipsilateral connections. In the positive predictive network, distribution of nodes across cerebellar hemispheres was numerically balanced with one hemisphere mirroring the other; judging by weighted degree, though, left cerebellar nodes ruled superior. Cerebellar nodes in the negative predictive network, on the other hand, were predominantly (number-wise and degree-wise) found in the right hemisphere, which is also reflected in their comparative weighted degrees (see Table 3 and Table 2). With regard to anatomical distribution of cerebral nodes involved in our predictive networks, temporal poles and entorhinal and perirhinal cortices featured prominently in the positive predictive network, while they were conspicuously absent in the negative network. Other regions present in both networks notably included the lateral and medial prefrontal cortices as well as superior parietal areas. Interestingly, cerebellocerbral edges tended to rank higher than intracerebellar edges among those with highest predictive power (see Figure 7), supporting the notion of cerebellar function being mediated through its involvement in cerebral networks.

Suprathreshold cerebellar nodes in both networks were almost exclusively located in the posterior hemispheres. This can be interpreted in light of the cerebellum's functional division. A coarse division based on functional associations can delineate a motor cerebellum (anterior hemispheres), a limbic cerebellum (vermis and adjacent regions, the paravermis) and a cognitive cerebellum (posterior hemispheres). The posterior cerebellum's dominant role in cognitive processes was confirmed in lesion studies [78] and comparative anatomy lends support to the notion of the posterior hemispheres contributing to higher cognition, as they are the phylogenetically youngest part and seem to have expanded alongside cerebral regions relevant to cognitive functions associated with modern humans [79, 80, 81, 82].

In fact, most nodes of the 10 most predictive edges were located in lobule VII and all of them in lobules VI, VII and VIII (see Figure 7). Gross-anatomically, the cerebellum can be divided into two lobes (anterior and posterior) and 10 lobules I to X. While lobules I to IV correspond to the anterior lobe, lobule VI to IX are part of the posterior lobe; lobule VII is subdivided into crus I, crus II and lobule VIIb. In line with the general tendency of our results, lobule VII specifically (and to a lesser degree lateral lobule VI) is considered to be the principal lobule of the cognitive-affective cerebellum, with regions of lobule VIII also implicated [83, 84, 28]. Note that the only node from the positive predictive network not located in lobule VII was located in lobule VIIIa, which is adjacent to lobule VII; this might reflect a meaningful discrepancy between structural-anatomical and functional divisions. The bulk of lobule VIII, on the other hand, forms part of the motor cerebellum.

This notion of functional specialization of cerebellar regions is borne out by recent conceptualizations. Multiple lines of neuroimaging evidence converge on multiple representations of non-motor functions (task-negative, i.e. DMN-related, and task-positive, i.e. executive function-related and attention-related). Within this division, lobule VI and crus I form the first non-motor representation, crus II the second non-motor representation [21, 85]. As mentioned, crus I and crus II are part of lobule VII and most of our most relevant nodes are found in these cerebellar re-

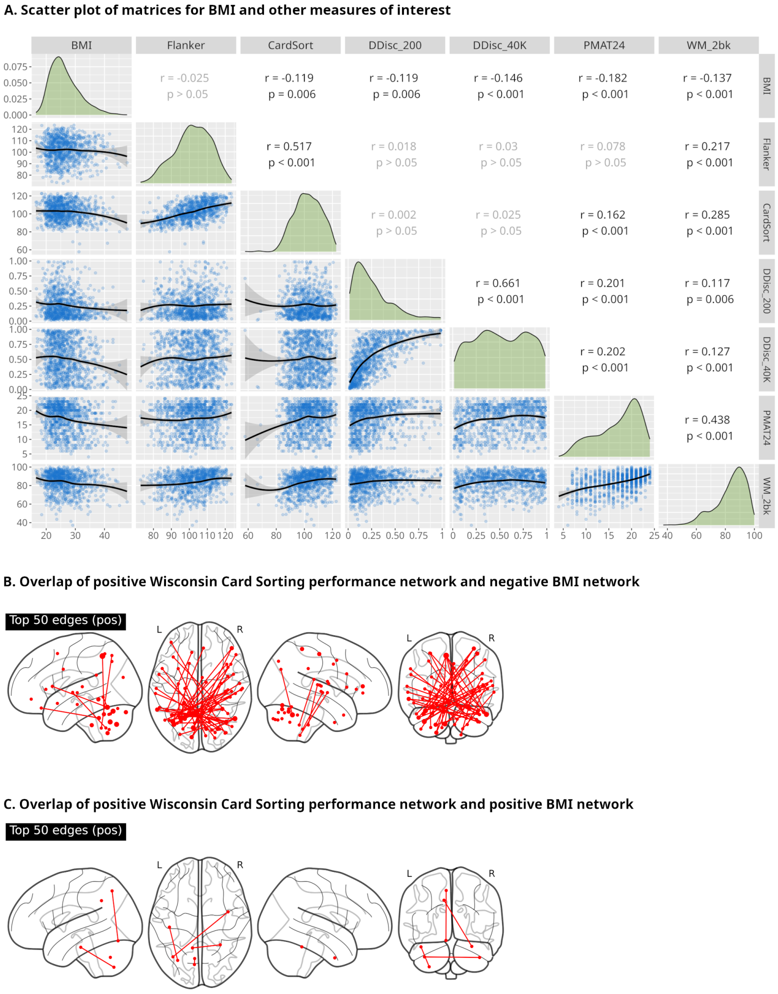

Figure 6. A. Scatter plot of matrices visualizing Pearson correlation for BMI and other measures of interest. Diagonally, a density plot of the measures are shown, the lower triangle depicts scatter plots of the respective measures, while the upper triangle denotes Pearson's r and Bonferroni-corrected p values as determined by permutation testing[b] as none of the measured showed a normal distribution in our sample (as seen in the density plots and verified with Shapiro-Wilk normality testing). *BMI*: Body mass index; *CardSort*: age-adjusted results for Wisconsin Card Sorting task; *DDisc_200/40K*: area under the curve for delay discounting (200 $ and 40.000 $ conditions, respectively); *Flanker*: age-adjusted results for flanker task; *PMAT24*: correct responses in the Penn Matrix Reasoning Test; *WM_2bk*: accuracy in the working memory two-back task. **B.** Example overlap between the network *positively* predicting Winsconsin card sorting performance and the network *negatively* predicting BMI. **C.** Example overlap (or lack thereof) between the network *positively* predicting Winsconsin card sorting performance and the network *positively* predicting BMI.

b The code used to replace ggplot2's [68] built-in parametric test function along with an example call used to generate image A of this figure is part of `hcp-suite` and can be found in our repository (see below) in `hcp-suite/utils/add_perm_P.R`.

gions. Crus I and crus II It is worth noting that functional divisions do not conform to structural divisions in lobes and lobules, which has been confirmed by another recent major study by King et al. [83]. It is not surprising, then, that the nodes found to be most relevant in our study do not strictly adhere to structural boundaries like lobules and crurae.

In a recent metareview concerned with the cerebellum's role in appetite control, crus I and lobule VI were highlighted as being consistently altered, a finding the authors explained by pointing out these cerebellar regions' association with executive and emotional control [86]. There is direct evidence for BMI-associated activation of crus I and lobule VI in response to food cues with the increased activation of these cerebellar areas being reversible after leptin-replacement associated BMI reduction in leptin-deficient individuals [87]. Both the latter study and the aforementioned metareview by Sader et al. recognized an asymmetry in crus I and lobule VI activation favoring the left cerebellum, which can also be seen in our results (for the positive predictive network, as expected; see image B in Figure 7).

Lobule VI and crus I are also implicated in reward processing, which in the context of our analysis resonates with the view of obesity as an addiction-like state with pathological food craving [88]. For instance, greater functional connectivity of bilateral lobule VI with the ventral tegmental area, an important reward-processing center, in obese women in response to energy-dense food cues was reported [89]. In a study on regular cocaine users, cocaine and food cues led to a similar pattern of activation predominantly in lobule VI and crus I [90]. Reward-related direct cerebellar projections to the ventral tegmental area seem to be of a behaviorally highly relevant modulating nature [91], possibly on the basis of intracerebellar model generation and prediction error correction [92].

In addition, experimental evidence suggests a role of Lobule VI and crus I in prediction error handling of emotional stimuli [93]. Overeating and obesity are intricately linked to negative affective states [94, 95]. Drawing conclusions from these associations is not straightforward since negative social bias towards obesity may contribute to affective disorders. Yet, cerebellar processing of predicted emotional consequences of food intake may contribute to overeating [96].

As described above, we noted a per-network lateralization of cerebellar nodes with the positive predictive network being characterized by weightier left cerebellar and the negative predictive

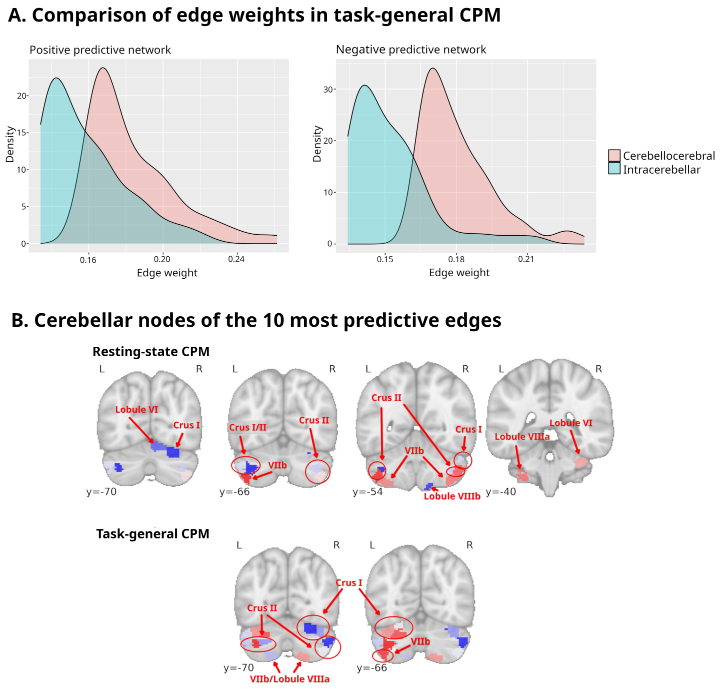

Figure 7. **A.** Comparison of weights of cerebellocerebral and intracerebellar edges of the positive (image on the left) and negative (image on the right) predictive network for task-general connectome-based predictive modeling. **B.** Cerebellar nodes of the 10 most predictive edges of connectome-based predictive modeling of task-based functional magnetic resonance imaging (fMRI) (top panel) or resting-state fMRI (bottom panel). Nodes of positive predictive networks are colored in red, nodes of negative predictive networks in blue. Location of nodes with regard to cerebellar lobules are annotated. Coordinates are MNI coordinates. *CPM*: connectome-based predictive modeling; *MNI*: Montreal Neurological Institue

network by weightier right cerebellar nodes. Several functional neuroimaging and lesion studies speak to a lateralization of higher cognitive cerebellar function. Broadly, the right cerebellum is associated with language and more general cognition, although this could well be secondary to its function in language. Of interest to our study, D'Mello et al. found concrete executive control to be lateralized to the right cerebellum [97]. The left cerebellum's role is less elucidated (it also plays only a minor role in tDCS studies, see [98]), but a metaanalysis of functional neuroimaging data reveals a strong association with executive function [28], while others found it to be involved in attention and visuospatial processing (for an overview see [99]). It is unclear at this point how our results fit into this picture.

Task-general positive predictive network

We set out to investigate cerebellocerebral connectivity on the grounds of advanced theories of cerebellar function positioning cerebellar function, insofar it contributes to higher cognitive function, within cerebral networks. Accordingly, we were able to identify established neural networks within our positive and negative predictive task-general networks. Habas et al. analysed cerebellar contributions to cerebral networks including the central executive network [23]. The central executive or frontoparietal network (for a discussion of nomenclature see [100, 74]) consists of intercommunicating brain areas locarsted primarily in the bilateral frontal and parietal lobes, where they form a mirror image of each other. This signature pattern of nodes reverberates in our positive predictive network's cerebral components, albeit with preference of the right hemisphere; this is reflected in the cerebellar nodes left-hemispheric preference. Other than that, the cerebellar areas participating in the frontoparietal network found by Habas et al. could be replicated in our positive predictive network (see image B in Figure 5).

The presence of frontoparietal networks (FPN) in our positive predictive network offers an explanation of the task-general model's superior predictive capabilities in comparison to single-task models. Averaging the latters' connectivity matrices accentuates common predictive patterns, e.g. a meta-task network such as the FPN. Early termed the central executive or central control network, the FPN are a set of networks for which no universally agreed-on terminology exists (for a valuable effort to resolve some of the confusion surrounding frontoparietal networks involved in executive function see [74]). Yet, considerable agreement exists toward their function. They are attributed a key role in executing so-called executive functions (EF). The term EF denotes a set of cognitive functions underlying virtually all conscious behavior (for an overview see [101]. One of the core components of executive function is working memory (the others being inhibitory control and cognitive flexibility; building on those are planning, reasoning and problem-solving as higher-order executive functions). While the working-memory task is therefore a direct measure of executive function performance, all tasks rely on higher cognitive function and thus on EF; their relation to overweight and the cerebellum therefore warrant a broader discussion. The bilateral entorhinal and pararhinal cortices contribute prominently to our positive predictive network; both areas are implicated in working memory and, likely therefore, executive functions performance (the latter might be secondary to the former [102, 103, 104]; for an integrative theory of working memory and executive function implying the perirhinal cortex see [105]).

Several studies were able to demonstrate lower EF in overweight individuals (for reviews see [106, 8, 9, 107]. Experimental evidence suggests impairments specifically in EF as e.g. working-memory deficits can be elicited even though explicit learning is unaffected by obesity [108]. Models of understanding the relationship between EF impairment and obesity point to the importance of EF in dietary intake control as they mediate the kind of goal-orientated behavior that is necessarily involved with delayed gratification [109, 110].

Lesion and neuroimaging studies, on the other hand, robustly show the cerebellum's involvement in EF. The original lesion study leading to formulation of the CCAS [111] described as one of its hall-

marks EF deficits (next to deficits in visuospatial cognition, language and affection – for an updated description see [112]).

Even better connected than areas implicated in the frontoparietal networks were nodes symmetrically located at the bilateral temporal poles, with ventral and apical temporopolar nodes being the best connected of all noncerebellar nodes. The temporal pole seems to be an integration center for a range of modalities (for an overview see [113]) with the apex in particular functioning as a hub interconnecting other temporopolar functional divisions. The temporopolar apex mainly consists of area TG, which ranges top of our best connected nodes of the positive predictive network, and is noted by Pascual et al. for its strong functional connectivity to the posterior cerebellum [114]. In addition to its role as a temporopolar hub, area TG's functional connectivity suggests, alongside ventrolateral area TE's, a role in semantic processing (note that left area TE1 is also among the highest-degree nodes of the positive predictive network). Integrative, amodal semantic processing is indeed thought to be central to temporopolar function [115] with a stimulation study elegantly confirming the semantic-notional (as opposed to verbal-linguistic) nature of this specific temporopolar function [116]. For neurodegenerative diseases like Alzheimer's and frontemporal dementia, temporopolar hypometabolism has been linked to deficits in executive function [117, 118]. Given this evidence, it does not seem too great a leap to assume the temporal poles' meta-task relevance.

As a fourth component pattern, with the orbital frontal cortex, the medial prefrontal cortex and adjacent anterior cingulate cortex as well as lateral temporal corices, inferior parietal lobes, posterior cingulate/retrosplenial cortex and hippocampus/parahippocampal cortices all key hubs of the default mode network (DMN) were present. The DMN is discussed in more detail in the context of the rsfMRI's negative predictive network.

Task-general negative predictive network

While the positive network comprised cerebral nodes associated with the FPN and the temporal poles as supramodal hubs, the negative predictive network's most relevant nodes (see image C in Figure 5) are found in the bilateral primary sensory and motor cortices as well as auditory cortices. In terms of established cerebral networks, the pattern of (cerebral) nodes in the negative predictive network resembled most closely the sensorimotor, or pericentral, network (SMN), which centers around primary sensor and motor cortices. The SMN is primarily involved with integration of sensory inputs and coordination of motor outputs. As such, it is connected to numerous other networks and regions including, unsurprisingly, the cerebellum. Not unlike the cerebellum, the SMN has recently been implicated in non-motor functions, including executive function [119, 120] and task-independent temporal processing, which has termed sensorimotor synchronization [121, 122].

In sensorimotor synchronization, the auditory system is thought to play a crucial role [123]. The relationship between SMN and auditory cortices is indeed close to a point where the latter have been considered to be part of the former [100, 25]. This relationship, like the SMN's great interconnectedness in general, can be conceptualized within the SMN's prominent role in internal modeling and model updating (for a review of evidence and theoretical constructs for the auditory cortices' contribution to internal modeling, see [124]).

Summary and conclusions

Before providing a high-level summary of our study and exploring possible explanations of its findings, we think it helpful to offer four key points that can be distilled from our CPM results:

1. Cerebellocerebral connectivity predicts BMI.
2. Task-general cerebellocerebral connectivity most reliably predicts BMI.
3. Predictive networks derived this way overlap with established functional brain networks.
4. There is an inverse overlap between networks predictive of BMI and networks predictive of measures aversely affected by overweight/obesity (i.e. positive predictive networks overlapped with negative predictive networks and vice versa).

Applying connectome-based predictive modeling to functional MRI parcellated along functionally informed lines, we built general linear models to predict BMI. Each general linear model was based on two separate networks consisting of edges correlating positively or, respectively, negatively with BMI. In comparison, models built with task-based functional MRI predicted BMI with substantially higher accuracy than those built with resting-state functional MRI. Nevertheless, the respective predictive networks of both modalities shared common features.

Generally speaking, both positive and negative predictive networks featured multimodal integration hubs (temporal poles and ventromedial temporal lobes for the positive and auditory cortices for the negative predictive networks). Consistent with evidence positioning cerebellar functional connectivity within cerebral connectivity, we were able to identify established neural networks within positive and negative predictive networks. For the positive predictive networks, these were frontoparietal networks involved in EF, the default mode (tfMRI only) and the salience network (rsfMRI only). Within our negative predictive networks, we found the sensorimotor (both tfMRI and rsfMRI) and default mode network (rsfMRI only).

On the basis of its homogenous cellular architecture, it has been proposed that the cerebellum is performing the same operation on the input it receives independent of the nature of the function it is involved in (e.g. basic motor or higher cognitive function), aptly called "universal cerebellar transform" (see e.g. [125]). Cerebellar nodes can thus be thought of as processing hubs modulating cerebral function. Consistent with our results, the posterior cerebellum, specifically lobule VII, has been previously highlighted as the location of such hubs [126].

Lobule VII was also highlighted in a study finding gray-matter density in the cerebellum to be negatively correlated with neuron-specific enolase plasma levels, a marker of neuronal injury [127]. The authors interpret that finding as evidence for cerebellar vulnerability to obesity-related neuronal injury. A possible mechanistic link between altered cerebellar properties and obesity is provided by leptin, a hormone heavily involved in obesity [128]. Leptin receptors are most densely expressed in the cerebellum [129] and induction or withdrawal of leptin replacement therapy in leptine-deficient individuals has been shown to increase [130] or decrease [131] cerebellar gray-matter volume, respectively. Leptine replacement therapy also reproducibly altered the functional response of the cerebellum to food cues [132, 87].

The nature of the cerebellum's influence on cerebral nodes and networks and why this would allow us to predict BMI in subjects, is beyond the scope of our study. Pointers can be gleaned from studies measuring cerebrobehavioral effects of modulating cerebellar-to-cerebral output. This can be achieved through non-invasive, i.e. transcranial, stimulation, where modulation of the cerebellar neuron's electrical activity has an impact on neuronal activity in connected cerebral areas. The most studied target area of this phenomenon is the primary motor cortex, where excitatory stimulation of the cerebellum has a depressive effect, which has been termed cerebellar brain inhibition [133]. Taking this robust effect as a starting point, efforts have been made to study the impact of transcranial cerebellar modulation on non-motor domains (for an overview see [134, 98]). The effect of cerebellar output is less clear, however, with evidence suggesting both inhibitory and excitatory roles (and sometimes both, see [135]).

If we accept the premises of (a) the cerebellum's role in cognition, affective regulation and task execution, (b) an inverse correlation of BMI and performance in these domains, and suppose that the cerebellum's altered functional status in overweight and obesity is an expression of these premises, our data would suggest an overall inhibitory role of the cerebellum. Cerebellar connectivity to EF-related networks was positively correlated with BMI, leading, in the proposed framework, to an inhibitory effect on task performance, while the salience network being part of rsfMRI's positive predictive network and the DMN being part of rsfMRI's negative predictive network would lead to less efficient task switching. The occurrence of an executive network in the rsfMRI's negative predictive network and of the DMN in the tfMRI's positive predictive network can be held against this hypothesis, although these networks do happen to be involved in their respectively "wrong" state of activeness, i.e. the executive network during rest and the DMN during task performance. A non-inhibitory role of the cerebellum in these cases would be an alternative interpretation.

Whatever the correct mechanistic interpretation, empirical evidence does indeed implicate the cerebellum in feeding and appetite control. With its numerous reciprocal connections to key structures for homeostasis (including energy homeostasis and its circadian fluctuations), affective control and reward processing (where involvement in hedonic aspects of food intake has been shown) as well as motor aspects of eating, the cerebellum contributes to virtually every aspect of food intake (for a recent review see [96]).

Rather excitingly, in a rodent model of Prader-Willi syndrome, a syndrome characterized, among other things, by insatiable appetite, a drastic reduction in food intake could be achieved by selective activation of a cerebellar satiation network. Associated cerebellar regions had been identified in humans employing functional MRI while presenting food-related cues to subjects, as they were the only brain regions with significantly different activations in subjects affected by Prader-Willi syndrome compared to control subjects [136]. In humans, appetite can be modulated via transcranial cerebellar stimulation [137].

One reading of the notion of an obese brain holds that brains of individuals with obesity are a priori (i.e. prior to being conditioned by experience) predisposed to obesogenic behavior, leading to obesity when exposed to an obesogenic environment. However, there are most likely adaptive neural processes at play, creating and shaping obesity-related interneuronal connections, possibly reinforcing obesogenic behavior. Persuasive evidence of obese experiences shaping the obese brain can be found when studying individuals undergoing substantial weight loss, e.g. patients undergoing bariatric surgery. Central-nervous-system targets for such research usually involve the dopaminergic system (for a review including methodological limitations see [138]). While a recent study was negative with regard to changes in dopaminergic response to food intake, its post-surgery follow-up was rather short-term [139]. Van der Zwaal et al., on the other hand, were able to show increased dopamine receptor availability after long-term weight-loss in bariatric surgery patients [140]. It would be interesting to see if and how changes in BMI would change predictive networks in subjects.

Limitations

The HCP provided us with high-quality imaging data for a large number of subjects. But basing our analyses on preexisting data had drawbacks, as we were principally limited by the kind of data provided. While resting-state fMRI is inherently goal-agnostic and therefore not affected by this aspect, when designing an obesity study from scratch, a different selection of tasks might have been more appropriate to answer our questions. Yet, this fact is mitigated twofold. Firstly, the HCP carefully chose their tasks to cover as much ground as possible and thus included tasks suited for our purposes. Secondly, task-general brain activity turned out to be more predictive rendering specific task content less important.

We used BMI as our principal target measure as the biometric measures it is based on is available for virtually all HCP subjects. While being the most commonly used measure for obesity, arguably because it is easily determined, other measures may perform better in capturing obesity as a condition worthy of medical consideration (e.g. they are better predictors for obesity-related disorders) [141, 142, 143, 144, 145]. This is a concern for our study, as the biological basis for its assumption of altered cerebellocerebral connections is the existence of that very obesity condition.

On a final note, it is important to keep in mind that, due to the correlative nature of our study, causal relationships cannot be established.

Availability of source code and requirements

In order to obtain our results, a number of methodological-technical hurdles had to be overcome. One of these had to do with the HCP's development of new imaging protocols and file formats store imaging data. Thus, with regard to our purposes, no established analysis pipelines existed. In addition, to complete our analysis in a reasonable timeframe, computing power not available in a single machine was required, which led to extensive use of cluster computing. Our efforts were helped intensively by using open-source software, which allowed us to inspect existing software, build upon existing solutions, and adapt code freely according to our needs on every level (from high-level scripting to writing custom functions altering low-level backend code).

Our newly-developed software framework for CPM with a focus on HCP data is available under the GNU General Public License v3 in a Git repository at `https://codeberg.org/tobac/hcp-suite`. The repository also includes a detailed tutorial on how to use our software. The method was registered with the DOME-ML registry [146]. A Jupyter notebook is provided in the associated GigaDB dataset to reproduce the results reported in this paper.

- Project name: HCP Suite
- Project home page: `https://codeberg.org/tobac/hcp-suite`
- bio.tools Identifier: `biotools:hcp_suite`
- WorkflowHub DOI: `10.48546/WORKFLOWHUB.WORKFLOW.1234.1`
- RRID: `RRID:SCR_026222`
- Latest Git commit this project is based on: `2677bbba7e`
- Operating system(s): Platform independent
- Programming language: Python $\geq$ 3.7, R $\geq$ 4.0, GNU bash $\geq$ 4.4
- Other requirements: See the README in the repository for installation instructions
- License: GNU GPL v3

The specific software versions used to generate the results presented in this article were Python 3.11.5 [147], R 4.4.1 [148] and GNU bash 5.2.15.

Notable Python packages include NetworkX 3.3 [149], nibabel 5.2.1 [150], nilearn 0.10.4 [39], Pandas 2.2.2 [151], Pingouin 0.5.5 [152], and Ray 2.35.0 [153].

Notable R packages include ggplot2 3.5.1 [68], gtsummary 1.7.2 [154], and visNetwork 2.1.2 [64].

Data availability

Imaging and non-imaging data are available via the HCP after registering. Specifically, the following resources provide starting points to access and handle the public data set this study is based on ("Human Connectome Project – Young Adult Study").

All supporting data and materials are available in the GigaScience repository, GigaDB [155].

- Public data website: https://www.humanconnectome.org/study/hcp-young-adult
- Register for access: https://db.humanconnectome.org/app/template/Login.vm(simplesign-on)
- Access to restricted data (includes link to e-access application form): https://www.humanconnectome.org/study/hcp-young-adult/document/restricted-data-usage
- Data: WU-Minn HCP 1200 Subjects Data Release, for which the following reference manual applies: https://www.humanconnectome.org/storage/app/media/documentation/s1200/HCP_S1200_Release_Reference_Manual.pdf

Declarations

List of abbreviations

- BMI: Body mass index
- CCAS: Cerebellar Cognitive Affective Syndrome
- CPM: Connectome-based predicitive modeling
- dmFPN: Dorsomedial frontoparietal network
- DMN: Default mode network
- EF: Executive functions
- fMRI: Functional magnetic resonance imaging
- FPN: Frontoparietal network
- GLM: General linear model
- HCP: Human Connectome Project
- IQR: Interquartile range
- MNI: Montreal Neurological Institute
- MRI: Magnetic resonance imaging
- rsfMRI: Resting-state functional magnetic resonance imaging
- SMN: Somatomotor network
- SN: Salience network
- tfMRI: Task-based functional magnetic resonance imaging
- WM: Working memory

Ethics approval

This study is based on data as provided by the Human Connectome Project, the HCP Data Use Terms have been accepted by all authors directly involved with the data's analysis. The need for an ethics approval was waived.

Consent for publication

Not applicable.

Competing Interests

The authors declare that they have no competing interests.

Funding

The authors declare that there is no funding associated with the work featured in this article.

Author's Contributions (CRediT roles)

- Tobias Bachmann: Data curation; formal analysis; methodology; software; writing – original draft; visualization; writing – review and editing
- Karsten Mueller: Writing – review and editing
- Simon N. A. Kusnezow: Writing – review and editing
- Matthias L. Schroeter: Writing – review and editing
- Paolo Piaggi: Writing – review and editing
- Christopher M. Weise: Conceptualization; methodology; supervision; writing – review and editing

Disclosure of use of AI-assisted tools including generative AI

The authors declare that no AI-assisted tools including generative AI have been used to write this article or generate its contents.

Acknowledgements

Data were provided in part by the Human Connectome Project, WU-Minn Consortium (Principal Investigators: David Van Essen and Kamil Ugurbil; 1U54MH091657) funded by the 16 NIH Institutes and Centers that support the NIH Blueprint for Neuroscience Research; and by the McDonnell Center for Systems Neuroscience at Washington University.

The authors gratefully acknowledge the generous computing time made available to them on the high-performance computing cluster provided by the Team Scientific Computing at the Leipzig University Computing Center.

References

1. NCD Risk Factor Collaboration (NCD-RisC). Worldwide Trends in Body-Mass Index, Underweight, Overweight, and Obesity from 1975 to 2016: A Pooled Analysis of 2416 Population-Based Measurement Studies in 128·9 Million Children, Adolescents, and Adults. Lancet (London, England) 2017 Dec;390(10113):2627–2642.
2. (WHO) WHO, Fact Sheets: Obesity and Overweight; 2021. https://www.who.int/news-room/fact-sheets/detail/obesity-and-overweight.
3. Blüher M. Obesity: Global Epidemiology and Pathogenesis. Nature Reviews Endocrinology 2019 May;15(5):288–298.
4. Barbosa DAN, Kuijper FM, Duda J, Wang AR, Cartmell SCD, Saluja S, et al. Aberrant Impulse Control Circuitry in Obesity. Molecular Psychiatry 2022 Aug;27(8):3374–3384.
5. Leigh SJ, Morris MJ. The Role of Reward Circuitry and Food Addiction in the Obesity Epidemic: An Update. Biological Psychology 2018 Jan;131:31–42.
6. Ziauddeen H, Alonso-Alonso M, Hill JO, Kelley M, Khan NA. Obesity and the Neurocognitive Basis of Food Reward and the Control of Intake. Advances in Nutrition 2015 Jul;6(4):474–486.
7. Verdejo-Román J, Vilar-López R, Navas JF, Soriano-Mas C, Verdejo-García A. Brain Reward System's Alterations in Response to Food and Monetary Stimuli in Overweight and Obese Individuals. Human Brain Mapping 2017 Feb;38(2):666–677.
8. Yang Y, Shields GS, Guo C, Liu Y. Executive Function Performance in Obesity and Overweight Individuals: A Meta-Analysis and Review. Neuroscience & Biobehavioral Reviews 2018 Jan;84:225–244.
9. Fitzpatrick S, Gilbert S, Serpell L. Systematic Review: Are Overweight and Obese Individuals Impaired on Behavioural Tasks of Executive Functioning? Neuropsychology Review 2013 Jun;23(2):138–156.
10. Olivo G, Gour S, Schiöth HB. Low Neuroticism and Cognitive Performance Are Differently Associated to Overweight and Obesity: A Cross-Sectional and Longitudinal UK Biobank Study. Psychoneuroendocrinology 2019 Mar;101:167–174.
11. García-García I, Michaud A, Dadar M, Zeighami Y, Neseliler

S, Collins DL, et al. Neuroanatomical Differences in Obesity: Meta-Analytic Findings and Their Validation in an Independent Dataset. International Journal of Obesity 2019 May;43(5):943–951.
12. Gómez-Apo E, Mondragón-Maya A, Ferrari-Díaz M, Silva-Pereyra J. Structural Brain Changes Associated with Overweight and Obesity. Journal of Obesity 2021;2021:6613385.
13. Glickstein M, Strata P, Voogd J. Cerebellum: History. Neuroscience 2009 Sep;162(3):549–559.
14. Guell X, Hoche F, Schmahmann JD. Metalinguistic Deficits in Patients with Cerebellar Dysfunction: Empirical Support for the Dysmetria of Thought Theory. The Cerebellum 2015 Feb;14(1):50–58.
15. Hoche F, Guell X, Vangel MG, Sherman JC, Schmahmann JD. The Cerebellar Cognitive Affective/Schmahmann Syndrome Scale. Brain: A Journal of Neurology 2018 Jan;141(1):248–270.
16. Hoche F, Guell X, Sherman JC, Vangel MG, Schmahmann JD. Cerebellar Contribution to Social Cognition. The Cerebellum 2016 Dec;15(6):732–743.
17. Koziol LF, Budding DE, Chidekel D. From Movement to Thought: Executive Function, Embodied Cognition, and the Cerebellum. Cerebellum (London, England) 2012 Jun;11(2):505–525.
18. Manto M, Mariën P. Schmahmann's Syndrome - Identification of the Third Cornerstone of Clinical Ataxiology. Cerebellum & Ataxias 2015 Feb;2(1):2.
19. Mariën, Borgatti. Language and the Cerebellum. Handbook of clinical neurology 2018;154.
20. Schmahmann JD. The Cerebellum and Cognition. Neuroscience Letters 2019 Jan;688:62–75.
21. Guell X, Schmahmann JD, Gabrieli JD, Ghosh SS. Functional Gradients of the Cerebellum. eLife 2018 Aug;7:e36652.
22. Guell X, Gabrieli JDE, Schmahmann JD. Triple Representation of Language, Working Memory, Social and Emotion Processing in the Cerebellum: Convergent Evidence from Task and Seed-Based Resting-State fMRI Analyses in a Single Large Cohort. NeuroImage 2018 May;172:437–449.
23. Habas C, Kamdar N, Nguyen D, Prater K, Beckmann CF, Menon V, et al. Distinct Cerebellar Contributions to Intrinsic Connectivity Networks. Journal of Neuroscience 2009 Jul;29(26):8586–8594.
24. Marek S, Siegel JS, Gordon EM, Raut RV, Gratton C, Newbold DJ, et al. Spatial and Temporal Organization of the Individual Human Cerebellum. Neuron 2018 Nov;100(4):977–993.e7.
25. Buckner RL, Krienen FM, Castellanos A, Diaz JC, Yeo BTT. The Organization of the Human Cerebellum Estimated by Intrinsic Functional Connectivity. Journal of Neurophysiology 2011 Nov;106(5):2322–2345.
26. Keren-Happuch, Shen-Hsing Annabel C, Moon-Ho Ringo H, John E D. A Meta-Analysis of Cerebellar Contributions to Higher Cognition from PET and fMRI Studies: A Meta-Analysis of Cerebellar Contributions. Human Brain Mapping 2014 Feb;35(2):593–615.
27. Stoodley CJ, Desmond JE, Guell X, Schmahmann JD. Functional Topography of the Human Cerebellum Revealed by Functional Neuroimaging Studies. In: Manto MU, Gruol DL, Schmahmann JD, Koibuchi N, Sillitoe RV, editors. Handbook of the Cerebellum and Cerebellar Disorders Cham: Springer International Publishing; 2022.p. 797–833.
28. Stoodley CJ, Schmahmann JD. Functional Topography in the Human Cerebellum: A Meta-Analysis of Neuroimaging Studies. NeuroImage 2009 Jan;44(2):489–501.
29. Shen X, Finn ES, Scheinost D, Rosenberg MD, Chun MM, Papademetris X, et al. Using Connectome-Based Predictive Modeling to Predict Individual Behavior from Brain Connectivity. Nature Protocols 2017 Mar;12(3):506–518.
30. Glasser MF, Smith SM, Marcus DS, Andersson JLR, Auerbach EJ, Behrens TEJ, et al. The Human Connectome Project's Neuroimaging Approach. Nature Neuroscience 2016 Sep;19(9):1175–1187.
31. Glasser MF, Sotiropoulos SN, Wilson JA, Coalson TS, Fischl B, Andersson JL, et al. The Minimal Preprocessing Pipelines for the Human Connectome Project. NeuroImage 2013 Oct;80:105–124.
32. Robinson EC, Jbabdi S, Glasser MF, Andersson J, Burgess GC, Harms MP, et al. MSM: A New Flexible Framework for Multimodal Surface Matching. NeuroImage 2014 Oct;100:414–426.
33. Glasser, Coalson TS, Robinson EC, Hacker CD, Harwell J, Yacoub E, et al. A Multi-Modal Parcellation of Human Cerebral Cortex. Nature 2016 Aug;536(7615):171–178.
34. Coalson TS, Van Essen DC, Glasser MF. The Impact of Traditional Neuroimaging Methods on the Spatial Localization of Cortical Areas. Proceedings of the National Academy of Sciences 2018 Jul;115(27):E6356–E6365.
35. Coalson T, Glasser M, Harwell J, Oostenveld R, CIFTI-2 Connectivity File Formats Documentation CIFTI Working Group; 2014.
36. Karl Friston. Functional Integration in the Brain. In: Human Brain Function Elsevier; 2004.p. 971–997.
37. Tian Y, Margulies DS, Breakspear M, Zalesky A. Topographic Organization of the Human Subcortex Unveiled with Functional Connectivity Gradients. Nature Neuroscience 2020 Nov;23(11):1421–1432.
38. Ren Y, Guo L, Guo CC. A Connectivity-Based Parcellation Improved Functional Representation of the Human Cerebellum. Scientific Reports 2019 Dec;9(1):9115.
39. Abraham A, Pedregosa F, Eickenberg M, Gervais P, Mueller A, Kossaifi J, et al. Machine Learning for Neuroimaging with Scikit-Learn. Frontiers in Neuroinformatics 2014;8.
40. Pedregosa F, Varoquaux G, Gramfort A, Michel V, Thirion B, Grisel O, et al. Scikit-Learn: Machine Learning in Python. Journal of Machine Learning Research 2011;12:2825–2830.
41. Ng B, Dressler M, Varoquaux G, Poline JB, Greicius M, Thirion B. Transport on Riemannian Manifold for Functional Connectivity-Based Classification. In: Golland P, Hata N, Barillot C, Hornegger J, Howe R, editors. Medical Image Computing and Computer-Assisted Intervention – MICCAI 2014 Lecture Notes in Computer Science, Cham: Springer International Publishing; 2014. p. 405–412.
42. Varoquaux G, Baronnet F, Kleinschmidt A, Fillard P, Thirion B. Detection of Brain Functional-Connectivity Difference in Post-stroke Patients Using Group-Level Covariance Modeling. In: Jiang T, Navab N, Pluim JPW, Viergever MA, editors. Medical Image Computing and Computer-Assisted Intervention – MICCAI 2010 Lecture Notes in Computer Science, Berlin, Heidelberg: Springer; 2010. p. 200–208.
43. Dadi K, Rahim M, Abraham A, Chyzhyk D, Milham M, Thirion B, et al. Benchmarking Functional Connectome-Based Predictive Models for Resting-State fMRI. NeuroImage 2019 May;192:115–134.
44. Pervaiz U, Vidaurre D, Woolrich MW, Smith SM. Optimising Network Modelling Methods for fMRI. NeuroImage 2020 May;211:116604.
45. Ledoit O, Wolf M. A Well-Conditioned Estimator for Large-Dimensional Covariance Matrices. Journal of Multivariate Analysis 2004 Feb;88(2):365–411.
46. Rohde K, Keller M, la Cour Poulsen L, Blüher M, Kovacs P, Böttcher Y. Genetics and Epigenetics in Obesity. Metabolism: Clinical and Experimental 2019 Mar;92:37–50.
47. Goodarzi MO. Genetics of Obesity: What Genetic Association Studies Have Taught Us about the Biology of Obesity and Its Complications. The Lancet Diabetes & Endocrinology 2018 Mar;6(3):223–236.
48. Xu Z, Xia M, Wang X, Liao X, Zhao T, He Y. Meta-Connectomic Analysis Maps Consistent, Reproducible, and Transcriptionally Relevant Functional Connectome Hubs

in the Human Brain. Communications Biology 2022 Oct;5(1):1056.
49. Fornito A, Zalesky A, Bassett DS, Meunier D, Ellison-Wright I, Yücel M, et al. Genetic Influences on Cost-Efficient Organization of Human Cortical Functional Networks. Journal of Neuroscience 2011 Mar;31(9):3261–3270.
50. Glahn DC, Winkler AM, Kochunov P, Almasy L, Duggirala R, Carless MA, et al. Genetic Control over the Resting Brain. Proceedings of the National Academy of Sciences of the United States of America 2010 Jan;107(3):1223–1228.
51. Thompson PM, Ge T, Glahn DC, Jahanshad N, Nichols TE. Genetics of the Connectome. NeuroImage 2013 Oct;80:475–488.
52. van den Heuvel MP, van Soelen ILC, Stam CJ, Kahn RS, Boomsma DI, Hulshoff Pol HE. Genetic Control of Functional Brain Network Efficiency in Children. European Neuropsychopharmacology 2013 Jan;23(1):19–23.
53. Barch DM, Burgess GC, Harms MP, Petersen SE, Schlaggar BL, Corbetta M, et al. Function in the Human Connectome: Task-fMRI and Individual Differences in Behavior. NeuroImage 2013 Oct;80:169–189.
54. Hariri AR, Tessitore A, Mattay VS, Fera F, Weinberger DR. The Amygdala Response to Emotional Stimuli: A Comparison of Faces and Scenes. NeuroImage 2002 Sep;17(1):317–323.
55. Delgado MR, Nystrom LE, Fissell C, Noll DC, Fiez JA. Tracking the Hemodynamic Responses to Reward and Punishment in the Striatum. Journal of Neurophysiology 2000 Dec;84(6):3072–3077.
56. Binder JR, Gross WL, Allendorfer JB, Bonilha L, Chapin J, Edwards JC, et al. Mapping Anterior Temporal Lobe Language Areas with fMRI: A Multicenter Normative Study. NeuroImage 2011 Jan;54(2):1465–1475.
57. Smith R, Keramatian K, Christoff K. Localizing the Rostrolateral Prefrontal Cortex at the Individual Level. NeuroImage 2007 Jul;36(4):1387–1396.
58. Castelli F, Happé F, Frith U, Frith C. Movement and Mind: A Functional Imaging Study of Perception and Interpretation of Complex Intentional Movement Patterns. NeuroImage 2000 Sep;12(3):314–325.
59. Wheatley T, Milleville SC, Martin A. Understanding Animate Agents: Distinct Roles for the Social Network and Mirror System. Psychological Science 2007 Jun;18(6):469–474.
60. Downing PE, Jiang Y, Shuman M, Kanwisher N. A Cortical Area Selective for Visual Processing of the Human Body. Science (New York, NY) 2001 Sep;293(5539):2470–2473.
61. Eklund A, Nichols TE, Knutsson H. Cluster Failure: Why fMRI Inferences for Spatial Extent Have Inflated False-Positive Rates. Proceedings of the National Academy of Sciences of the United States of America 2016 Dec;113(28):7900–7905.
62. Moritz P, Nishihara R, Wang S, Tumanov A, Liaw R, Liang E, et al., Ray: A Distributed Framework for Emerging AI Applications; 2018.
63. WU-Minn HCP consortium, editor, 1200 Subjects Data Release Reference Manual; 2017.
64. Almende B V and Contributors, Thieurmel B. visNetwork: Network Visualization Using 'vis.Js' Library; 2022.
65. R Core Team. R: A Language and Environment for Statistical Computing. Vienna, Austria: R Foundation for Statistical Computing; 2022.
66. Triarhou LC. A Proposed Number System for the 107 Cortical Areas of Economo and Koskinas, and Brodmann Area Correlations. Stereotactic and Functional Neurosurgery 2007;85(5):204–215.
67. Dianne Patterson, Atlases; 2023. https://neuroimaging-core-docs.readthedocs.io/en/latest/pages/atlases.html.
68. Wickham H. Ggplot2: Elegant Graphics for Data Analysis. Second edition ed. Use R!, Switzerland: Springer; 2016.
69. Menon V. Salience Network. In: Brain Mapping Elsevier; 2015.p. 597–611.
70. Peters SK, Dunlop K, Downar J. Cortico-Striatal-Thalamic Loop Circuits of the Salience Network: A Central Pathway in Psychiatric Disease and Treatment. Frontiers in Systems Neuroscience 2016;10.
71. Tomiyama H, Nakao T, Murayama K, Nemoto K, Ikari K, Yamada S, et al. Dysfunction between Dorsal Caudate and Salience Network Associated with Impaired Cognitive Flexibility in Obsessive-Compulsive Disorder: A Resting-State fMRI Study. NeuroImage Clinical 2019;24:102004.
72. Tan Z, Li G, Zhang W, Wang J, Hu Y, Li H, et al. Obese Individuals Show Disrupted Dynamic Functional Connectivity between Basal Ganglia and Salience Networks. Cerebral Cortex 2021 Dec;31(12):5676–5685.
73. García-García I, Jurado MÁ, Garolera M, Segura B, Sala-Llonch R, Marqués-Iturria I, et al. Alterations of the Salience Network in Obesity: A Resting-State fMRI Study. Human Brain Mapping 2013 Nov;34(11):2786–2797.
74. Witt ST, van Ettinger-Veenstra H, Salo T, Riedel MC, Laird AR. What Executive Function Network Is That? An Image-Based Meta-Analysis of Network Labels. Brain Topography 2021 Sep;34(5):598–607.
75. Raichle ME. The Brain's Default Mode Network. Annual Review of Neuroscience 2015 Jul;38(1):433–447.
76. Fox MD, Snyder AZ, Vincent JL, Corbetta M, Van Essen DC, Raichle ME. The Human Brain Is Intrinsically Organized into Dynamic, Anticorrelated Functional Networks. Proceedings of the National Academy of Sciences 2005 Jul;102(27):9673–9678.
77. Shannon BJ, Raichle ME, Snyder AZ, Fair DA, Mills KL, Zhang D, et al. Premotor Functional Connectivity Predicts Impulsivity in Juvenile Offenders. Proceedings of the National Academy of Sciences of the United States of America 2011 Jul;108(27):11241–11245.
78. Stoodley CJ, MacMore JP, Makris N, Sherman JC, Schmahmann JD. Location of Lesion Determines Motor vs. Cognitive Consequences in Patients with Cerebellar Stroke. NeuroImage Clinical 2016;12:765–775.
79. Smaers JB, Vanier DR. Brain Size Expansion in Primates and Humans Is Explained by a Selective Modular Expansion of the Cortico-Cerebellar System. Cortex; a Journal Devoted to the Study of the Nervous System and Behavior 2019 Sep;118:292–305.
80. Balsters JH, Cussans E, Diedrichsen J, Phillips KA, Preuss TM, Rilling JK, et al. Evolution of the Cerebellar Cortex: The Selective Expansion of Prefrontal-Projecting Cerebellar Lobules. NeuroImage 2010 Feb;49(3):2045–2052.
81. Weaver AH. Reciprocal Evolution of the Cerebellum and Neocortex in Fossil Humans. Proceedings of the National Academy of Sciences 2005 Mar;102(10):3576–3580.
82. Whiting BA, Barton RA. The Evolution of the Cortico-Cerebellar Complex in Primates: Anatomical Connections Predict Patterns of Correlated Evolution. Journal of Human Evolution 2003 Jan;44(1):3–10.
83. King M, Hernandez-Castillo CR, Poldrack RA, Ivry RB, Diedrichsen J. Functional Boundaries in the Human Cerebellum Revealed by a Multi-Domain Task Battery. Nature Neuroscience 2019 Aug;22(8):1371–1378.
84. Stoodley CJ, Schmahmann JD. Evidence for Topographic Organization in the Cerebellum of Motor Control versus Cognitive and Affective Processing. Cortex; a Journal Devoted to the Study of the Nervous System and Behavior 2010;46(7):831–844.
85. Guell X, Schmahmann J. Cerebellar Functional Anatomy: A Didactic Summary Based on Human fMRI Evidence. The Cerebellum 2020 Feb;19(1):1–5.
86. Sader M, Waiter GD, Williams JHG. The Cerebellum Plays More than One Role in the Dysregulation of Appetite: Review of Structural Evidence from Typical and Eating Disorder Pop-

ulations. Brain and Behavior 2023 Oct;p. e3286.
87. Berman SM, Paz-Filho G, Wong ML, Kohno M, Licinio J, London ED. Effects of Leptin Deficiency and Replacement on Cerebellar Response to Food-Related Cues. The Cerebellum 2013 Feb;12(1):59–67.
88. Volkow ND, Wang GJ, Tomasi D, Baler RD. Obesity and Addiction: Neurobiological Overlaps. Obesity Reviews 2013 Jan;14(1):2–18.
89. Carnell S, Benson L, Pantazatos SP, Hirsch J, Geliebter A. Amodal Brain Activation and Functional Connectivity in Response to High-Energy-Density Food Cues in Obesity. Obesity (Silver Spring, Md) 2014 Nov;22(11):2370–2378.
90. Tomasi D, Wang GJ, Wang R, Caparelli EC, Logan J, Volkow ND. Overlapping Patterns of Brain Activation to Food and Cocaine Cues in Cocaine Abusers: Association to Striatal D2/D3 Receptors. Human Brain Mapping 2015 Jan;36(1):120–136.
91. Carta I, Chen CH, Schott AL, Dorizan S, Khodakhah K. Cerebellar Modulation of the Reward Circuitry and Social Behavior. Science 2019 Jan;363(6424):eaav0581.
92. Kostadinov D, Beau M, Blanco-Pozo M, Häusser M. Predictive and Reactive Reward Signals Conveyed by Climbing Fiber Inputs to Cerebellar Purkinje Cells. Nature Neuroscience 2019 Jun;22(6):950–962.
93. Ernst TM, Brol AE, Gratz M, Ritter C, Bingel U, Schlamann M, et al. The Cerebellum Is Involved in Processing of Predictions and Prediction Errors in a Fear Conditioning Paradigm. eLife 2019 Aug;8:e46831.
94. Milaneschi Y, Simmons WK, van Rossum EFC, Penninx BW. Depression and Obesity: Evidence of Shared Biological Mechanisms. Molecular Psychiatry 2019 Jan;24(1):18–33.
95. Gariepy G, Nitka D, Schmitz N. The Association between Obesity and Anxiety Disorders in the Population: A Systematic Review and Meta-Analysis. International Journal of Obesity 2010 Mar;34(3):407–419.
96. Iosif CI, Bashir ZI, Apps R, Pickford J. Cerebellar Prediction and Feeding Behaviour. The Cerebellum 2023 Oct;22(5):1002–1019.
97. D'Mello AM, Gabrieli JDE, Nee DE. Evidence for Hierarchical Cognitive Control in the Human Cerebellum. Current biology: CB 2020 May;30(10):1881–1892.e3.
98. Oldrati V, Schutter DJLG. Targeting the Human Cerebellum with Transcranial Direct Current Stimulation to Modulate Behavior: A Meta-Analysis. Cerebellum (London, England) 2018 Apr;17(2):228–236.
99. Starowicz-Filip A, Prochwicz K, Kłosowska J, Chrobak AA, Myszka A, Bętkowska-Korpała B, et al. Cerebellar Functional Lateralization From the Perspective of Clinical Neuropsychology. Frontiers in Psychology 2021;12.
100. Uddin LQ, Yeo BTT, Spreng RN. Towards a Universal Taxonomy of Macro-Scale Functional Human Brain Networks. Brain topography 2019 Nov;32(6):926–942.
101. Cristofori I, Cohen-Zimerman S, Grafman J. Executive Functions. Handbook of Clinical Neurology 2019;163:197–219.
102. Hernandez AR, Reasor JE, Truckenbrod LM, Lubke KN, Johnson SA, Bizon JL, et al. Medial Prefrontal-Perirhinal Cortical Communication Is Necessary for Flexible Response Selection. Neurobiology of Learning and Memory 2017 Jan;137:36–47.
103. Skranes J, Løhaugen GCC, Evensen KAI, Indredavik MS, Haraldseth O, Dale AM, et al. Entorhinal Cortical Thinning Affects Perceptual and Cognitive Functions in Adolescents Born Preterm with Very Low Birth Weight (VLBW). Early Human Development 2012 Feb;88(2):103–109.
104. Izen SC, Chrastil ER, Stern CE. Resting State Connectivity Between Medial Temporal Lobe Regions and Intrinsic Cortical Networks Predicts Performance in a Path Integration Task. Frontiers in Human Neuroscience 2018 Oct;12:415.
105. Friedman NP, Robbins TW. The Role of Prefrontal Cortex in Cognitive Control and Executive Function. Neuropsychopharmacology 2022 Jan;47(1):72–89.
106. Favieri F, Forte G, Casagrande M. The Executive Functions in Overweight and Obesity: A Systematic Review of Neuropsychological Cross-Sectional and Longitudinal Studies. Frontiers in Psychology 2019;10.
107. Smith E, Hay P, Campbell L, Trollor JN. A Review of the Association between Obesity and Cognitive Function across the Lifespan: Implications for Novel Approaches to Prevention and Treatment. Obesity Reviews 2011;12(9):740–755.
108. Coppin G, Nolan-Poupart S, Jones-Gotman M, Small DM. Working Memory and Reward Association Learning Impairments in Obesity. Neuropsychologia 2014 Dec;65:146–155.
109. Dohle S, Diel K, Hofmann W. Executive Functions and the Self-Regulation of Eating Behavior: A Review. Appetite 2018 May;124:4–9.
110. Eichen DM, Pasquale EK, Twamley EW, Boutelle KN. Targeting Executive Function for Weight Loss in Adults with Overweight or Obesity. Physiology & Behavior 2021 Oct;240:113540.
111. Schmahmann JD, Sherman JC. The Cerebellar Cognitive Affective Syndrome. Brain: A Journal of Neurology 1998 Apr;121 (Pt 4):561–579.
112. Argyropoulos GPD, van Dun K, Adamaszek M, Leggio M, Manto M, Masciullo M, et al. The Cerebellar Cognitive Affective/Schmahmann Syndrome: A Task Force Paper. Cerebellum (London, England) 2020 Feb;19(1):102–125.
113. Herlin B, Navarro V, Dupont S. The Temporal Pole: From Anatomy to Function-A Literature Appraisal. Journal of Chemical Neuroanatomy 2021 Apr;113:101925.
114. Pascual B, Masdeu JC, Hollenbeck M, Makris N, Insausti R, Ding SL, et al. Large-Scale Brain Networks of the Human Left Temporal Pole: A Functional Connectivity MRI Study. Cerebral Cortex 2015 Mar;25(3):680–702.
115. Patterson K, Nestor PJ, Rogers TT. Where Do You Know What You Know? The Representation of Semantic Knowledge in the Human Brain. Nature Reviews Neuroscience 2007 Dec;8(12):976–987.
116. Pobric G, Jefferies E, Ralph MAL. Amodal Semantic Representations Depend on Both Anterior Temporal Lobes: Evidence from Repetitive Transcranial Magnetic Stimulation. Neuropsychologia 2010 Apr;48(5):1336–1342.
117. Schroeter ML, Vogt B, Frisch S, Becker G, Seese A, Barthel H, et al. Dissociating Behavioral Disorders in Early Dementia—An FDG-PET Study. Psychiatry Research: Neuroimaging 2011 Dec;194(3):235–244.
118. Schroeter ML, Vogt B, Frisch S, Becker G, Barthel H, Mueller K, et al. Executive Deficits Are Related to the Inferior Frontal Junction in Early Dementia. Brain 2012 Jan;135(1):201–215.
119. Zhu W, Tang W, Liang Y, Jiang X, Li Y, Chen Z, et al. Aberrant Functional Connectivity of Sensorimotor Network and Its Relationship With Executive Dysfunction in Bipolar Disorder Type I. Frontiers in Neuroscience 2022;15.
120. Kebets V, Holmes AJ, Orban C, Tang S, Li J, Sun N, et al. Somatosensory-Motor Dysconnectivity Spans Multiple Transdiagnostic Dimensions of Psychopathology. Biological Psychiatry 2019 Nov;86(10):779–791.
121. Repp BH, Su YH. Sensorimotor Synchronization: A Review of Recent Research (2006–2012). Psychonomic Bulletin & Review 2013 Jun;20(3):403–452.
122. Wiener M, Turkeltaub P, Coslett HB. The Image of Time: A Voxel-Wise Meta-Analysis. NeuroImage 2010 Jan;49(2):1728–1740.
123. Comstock DC, Hove MJ, Balasubramaniam R. Sensorimotor Synchronization With Auditory and Visual Modalities: Behavioral and Neural Differences. Frontiers in Computational Neuroscience 2018;12.
124. Burgess JD, Lum JAG, Hohwy J, Enticott PG. Echoes on the Motor Network: How Internal Motor Control Structures Af-

ford Sensory Experience. Brain Structure and Function 2017 Dec;222(9):3865–3888.
125. Guell X, Gabrieli JDE, Schmahmann JD. Embodied Cognition and the Cerebellum: Perspectives from the Dysmetria of Thought and the Universal Cerebellar Transform Theories. Cortex 2018 Mar;100:140–148.
126. Kawabata K, Bagarinao E, Watanabe H, Maesawa S, Mori D, Hara K, et al. Functional Connector Hubs in the Cerebellum. NeuroImage 2022 Aug;257:119263.
127. Mueller K, Sacher J, Arelin K, Holiga š, Kratzsch J, Villringer A, et al. Overweight and Obesity Are Associated with Neuronal Injury in the Human Cerebellum and Hippocampus in Young Adults: A Combined MRI, Serum Marker and Gene Expression Study. Translational Psychiatry 2012 Dec;2(12):e200–e200.
128. Obradovic M, Sudar-Milovanovic E, Soskic S, Essack M, Arya S, Stewart AJ, et al. Leptin and Obesity: Role and Clinical Implication. Frontiers in Endocrinology 2021 May;12.
129. Burguera B, Couce ME, Long J, Lamsam J, Laakso K, Jensen MD, et al. The Long Form of the Leptin Receptor (OB-Rb) Is Widely Expressed in the Human Brain. Neuroendocrinology 2000 Mar;71(3):187–195.
130. Matochik JA, London ED, Yildiz BO, Ozata M, Caglayan S, DePaoli AM, et al. Effect of Leptin Replacement on Brain Structure in Genetically Leptin-Deficient Adults. The Journal of Clinical Endocrinology and Metabolism 2005 May;90(5):2851–2854.
131. London ED, Berman SM, Chakrapani S, Delibasi T, Monterosso J, Erol HK, et al. Short-Term Plasticity of Gray Matter Associated with Leptin Deficiency and Replacement. The Journal of Clinical Endocrinology and Metabolism 2011 Aug;96(8):E1212–E1220.
132. Baicy K, London ED, Monterosso J, Wong ML, Delibasi T, Sharma A, et al. Leptin Replacement Alters Brain Response to Food Cues in Genetically Leptin-Deficient Adults. Proceedings of the National Academy of Sciences 2007 Nov;104(46):18276–18279.
133. Fernandez L, Major BP, Teo WP, Byrne LK, Enticott PG. Assessing Cerebellar Brain Inhibition (CBI) via Transcranial Magnetic Stimulation (TMS): A Systematic Review. Neuroscience and Biobehavioral Reviews 2018 Mar;86:176–206.
134. Manto M, Argyropoulos GPD, Bocci T, Celnik PA, Corben LA, Guidetti M, et al. Consensus Paper: Novel Directions and Next Steps of Non-invasive Brain Stimulation of the Cerebellum in Health and Disease. Cerebellum (London, England) 2022 Dec;21(6):1092–1122.
135. Sebastian R, Kim JH, Brenowitz R, Tippett DC, Desmond JE, Celnik PA, et al. Cerebellar Neuromodulation Improves Naming in Post-Stroke Aphasia. Brain Communications 2020;2(2):fcaa179.
136. Low AYT, Goldstein N, Gaunt JR, Huang KP, Zainolabidin N, Yip AKK, et al. Reverse-Translational Identification of a Cerebellar Satiation Network. Nature 2021 Dec;600(7888):269–273.
137. Marron EM, Viejo-Sobera R, Cuatrecasas G, Redolar-Ripoll D, Lorda PG, Datta A, et al. Prefronto-Cerebellar Neuromodulation Affects Appetite in Obesity. International Journal of Obesity 2019 Oct;43(10):2119–2124.
138. van Galen KA, Ter Horst KW, Booij J, la Fleur SE, Serlie MJ. The Role of Central Dopamine and Serotonin in Human Obesity: Lessons Learned from Molecular Neuroimaging Studies. Metabolism: Clinical and Experimental 2018 Aug;85:325–339.
139. van Galen KA, Schrantee A, ter Horst KW, la Fleur SE, Booij J, Constable RT, et al. Brain Responses to Nutrients Are Severely Impaired and Not Reversed by Weight Loss in Humans with Obesity: A Randomized Crossover Study. Nature Metabolism 2023 Jun;5(6):1059–1072.
140. van der Zwaal EM, de Weijer BA, van de Giessen EM, Janssen I, Berends FJ, van de Laar A, et al. Striatal Dopamine D2/3 Receptor Availability Increases after Long-Term Bariatric Surgery-Induced Weight Loss. European Neuropsychopharmacology: The Journal of the European College of Neuropsychopharmacology 2016 Jul;26(7):1190–1200.
141. Zhang X, Ma N, Lin Q, Chen K, Zheng F, Wu J, et al. Body Roundness Index and All-Cause Mortality Among US Adults. JAMA Network Open 2024 Jun;7(6):e2415051.
142. Suliga E, Ciesla E, Głuszek-Osuch M, Rogula T, Głuszek S, Kozieł D. The Usefulness of Anthropometric Indices to Identify the Risk of Metabolic Syndrome. Nutrients 2019 Oct;11(11):2598.
143. Amirabdollahian F, Haghighatdoost F. Anthropometric Indicators of Adiposity Related to Body Weight and Body Shape as Cardiometabolic Risk Predictors in British Young Adults: Superiority of Waist-to-Height Ratio. Journal of Obesity 2018;2018:8370304.
144. Huxley R, Mendis S, Zheleznyakov E, Reddy S, Chan J. Body Mass Index, Waist Circumference and Waist:Hip Ratio as Predictors of Cardiovascular Risk–a Review of the Literature. European Journal of Clinical Nutrition 2010 Jan;64(1):16–22.
145. O'Neill D. Measuring Obesity in the Absence of a Gold Standard. Economics and Human Biology 2015 Apr;17:116–128.
146. Bachmann T, Mueller K, Schroeter ML, Kusnezow SNA, Piaggi P, Weise CM. Cerebellocerebral Connectivity Predicts Body Mass Index: A New Open-Source Python-based Framework for Connectome-based Predictive Modeling. [DOME-ML Annotations]. DOME-ML Registry; 2025.
147. Python Software Foundation, Van Rossum G, Drake FL, The Python Language Reference; 2024.
148. R Core Team, R: A Language and Environment for Statistical Computing. Vienna, Austria; 2024. R Foundation for Statistical Computing.
149. Hagberg AA, Schult DA, Swart PJ. Exploring Network Structure, Dynamics, and Function Using NetworkX. In: Python in Science Conference Pasadena, California; 2008. p. 11–15.
150. Brett M, Markiewicz CJ, Hanke M, Côté MA, Cipollini B, McCarthy P, et al., Nipy/Nibabel: 5.2.1; 2024. Zenodo.
151. pandas development team T, Pandas-Dev/Pandas: Pandas; 2024. Zenodo.
152. Vallat R, Appelhoff S, Spaak E, JElfner, systole-docs, TAN G, et al., Raphaelvallat/Pingouin: V0.5.5; 2024. Zenodo.
153. Moritz P, Nishihara R, Wang S, Tumanov A, Liaw R, Liang E, et al. Ray: A Distributed Framework for Emerging AI Applications. In: Proceedings of the 13th USENIX Conference on Operating Systems Design and Implementation OSDI'18, USA: USENIX Association; 2018. p. 561–577.
154. Sjoberg DD, Whiting K, Curry M, Lavery JA, Larmarange J. Reproducible Summary Tables with the Gtsummary Package. The R Journal 2021;13(1):570–580.
155. Bachmann T, Mueller K, Kusnezow S, Schroeter M, Piaggi P, Weise C, Supporting Data for "Cerebellocerebral Connectivity Predicts Body Mass Index: A New Open-Source Python-based Framework for Connectome-based Predictive Modeling". GigaScience Database; 2025.

Click here to access/download;Figure;figure-cerebellar_edges.png

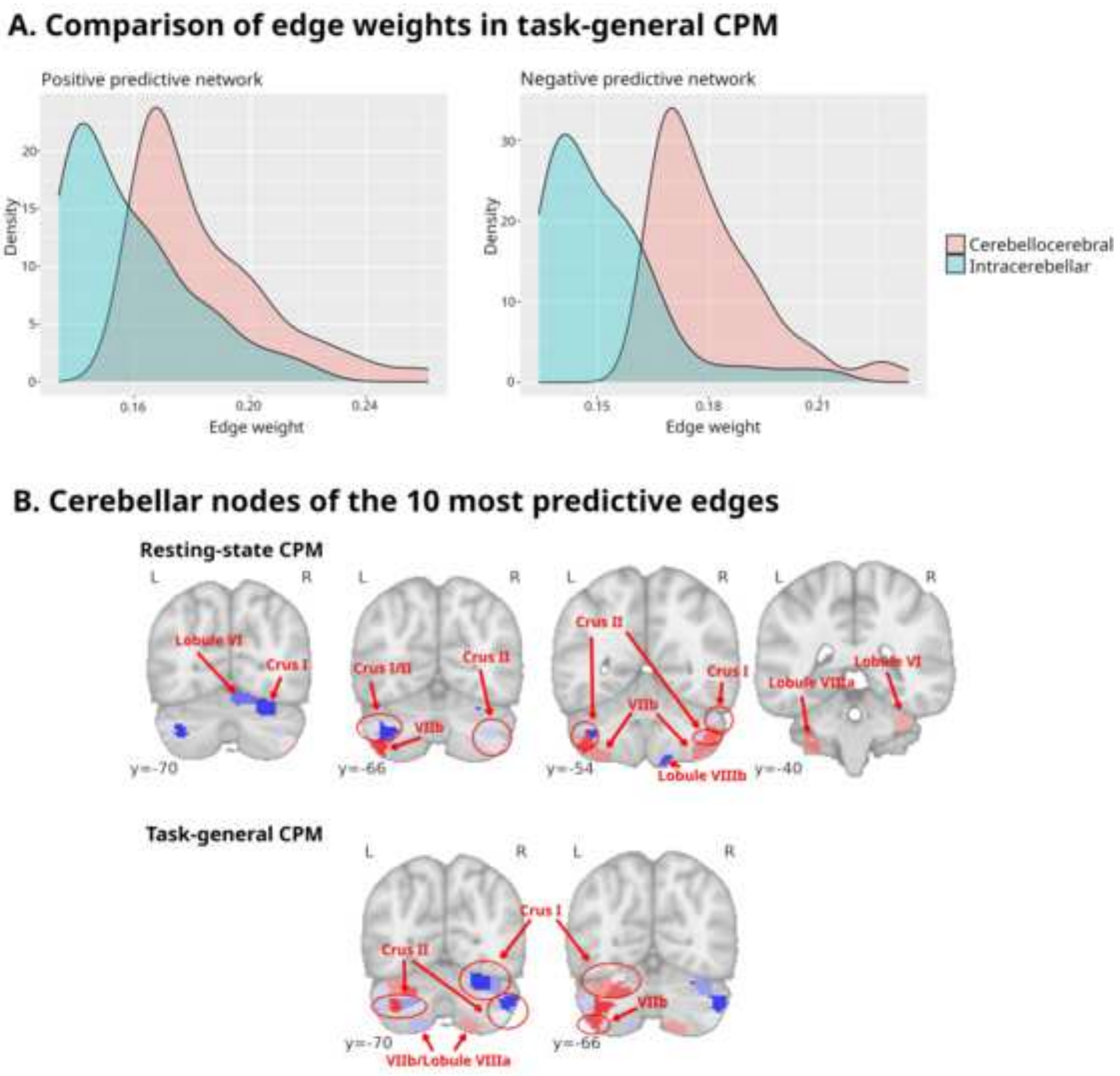

Click here to access/download;Figure;figure-overlap.png

A. Scatter plot of matrices for BMI and other measures of interest

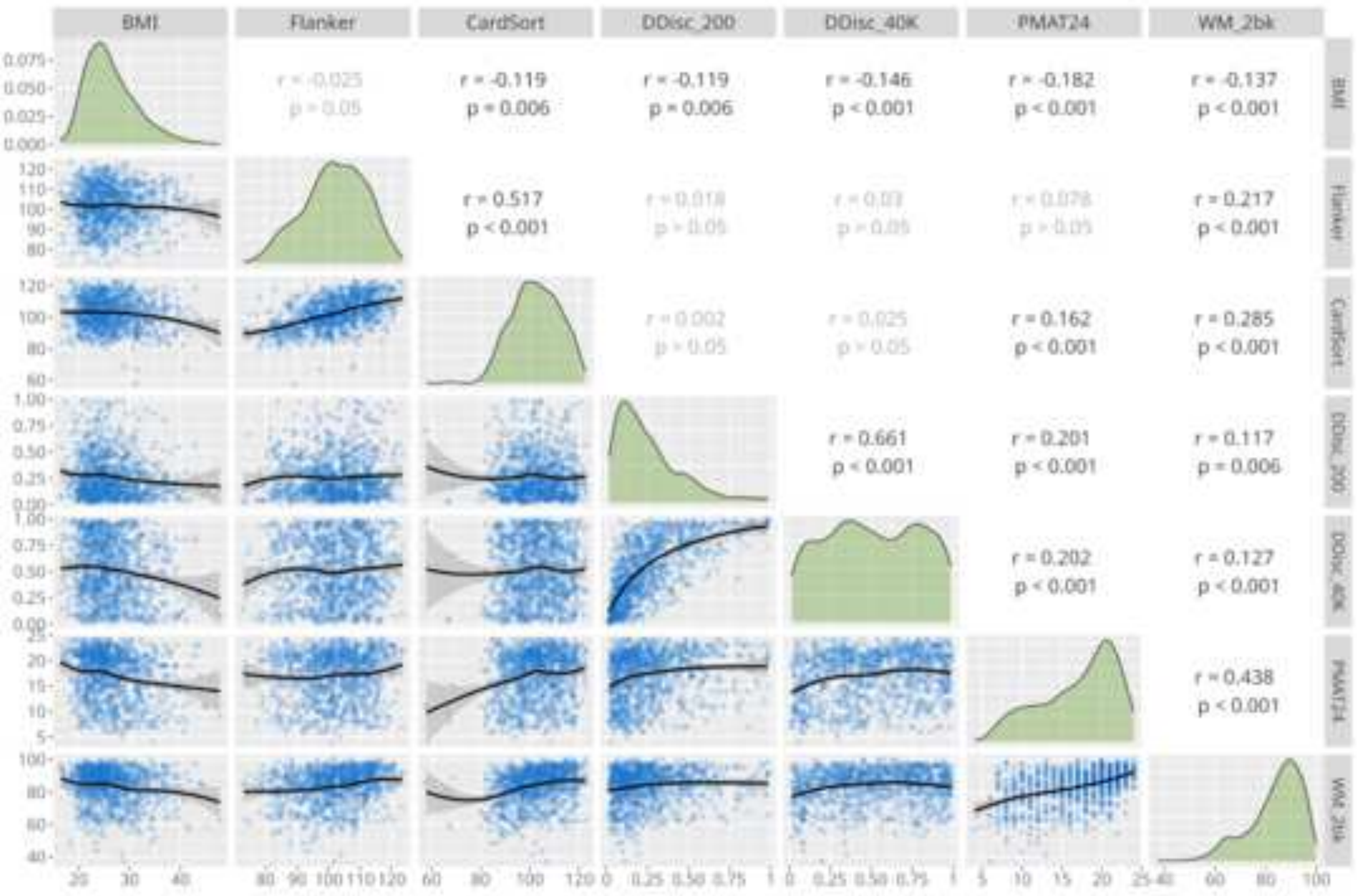

B. Overlap of positive Wisconsin Card Sorting performance network and negative BMI network

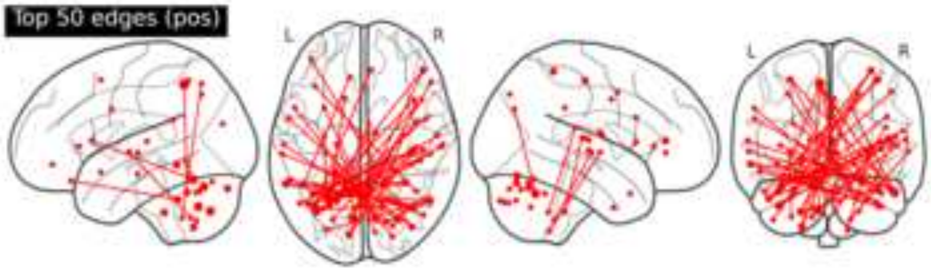

C. Overlap of positive Wisconsin Card Sorting performance network and positive BMI network

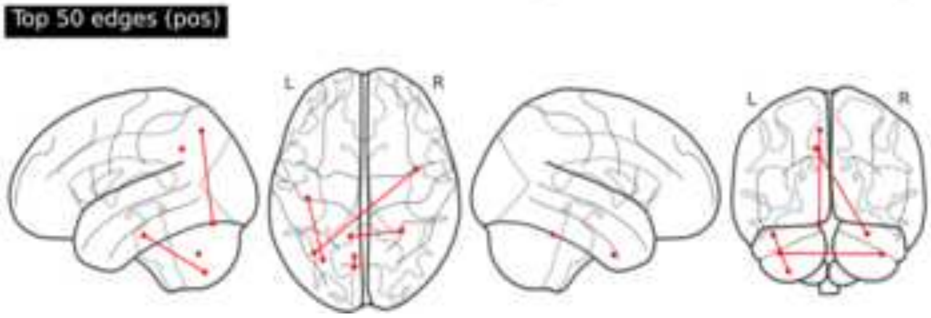

Click here to access/download;Figure;figure-results_tfmri.png

A. Prediction results and predictive networks of task-general CPM

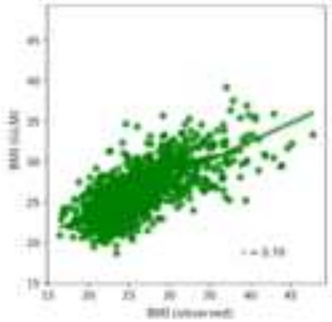

Predicted BMI (glm) plotted against observed BMI

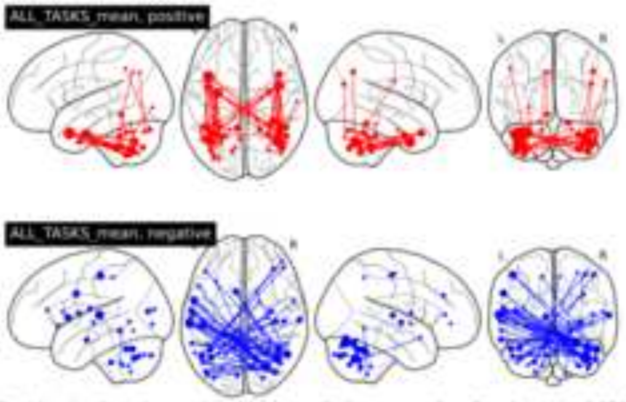

Positive (red) and negative (blue) predictive networks of task-general CPM

B. Highest-degree nodes of the task-general positive predictive network and corresponding brain networks

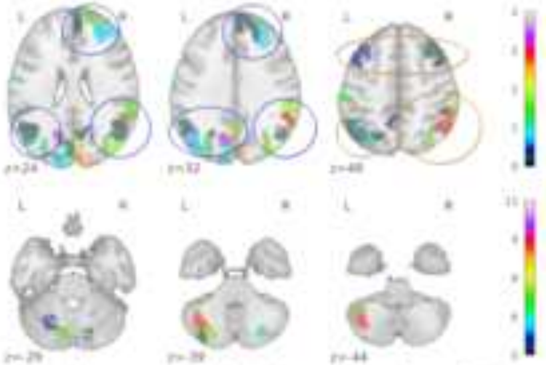

Highest-degree nodes of the task-general positive predictive network

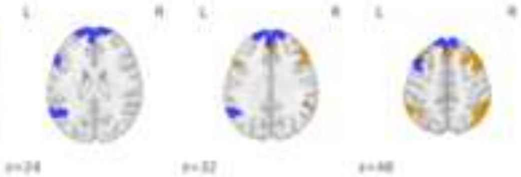

Two distinct frontoparietal networks (blue and orange)

C. Highest-degree nodes of task-general negative predictive network and corresponding brain network

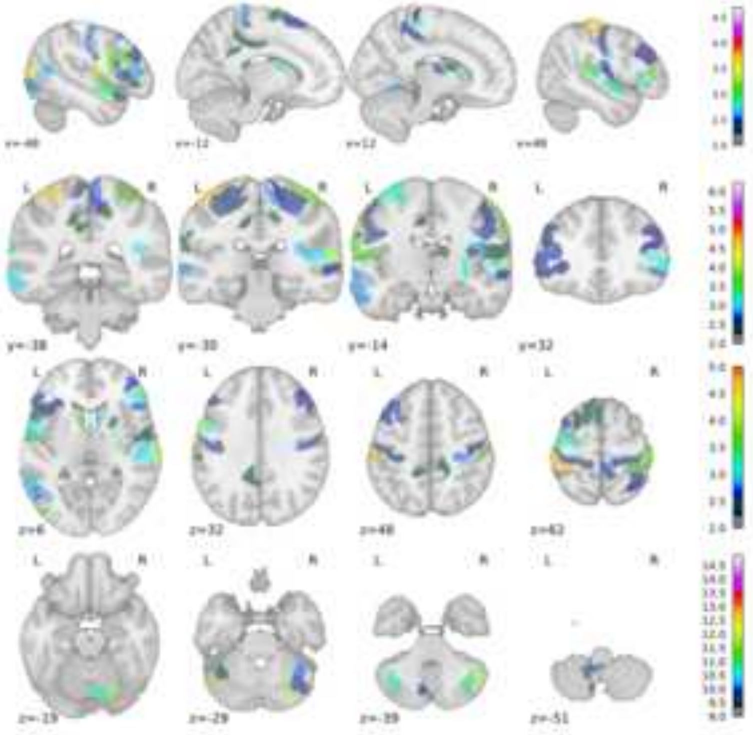

Highest-degree nodes of the task-general negative predictive network

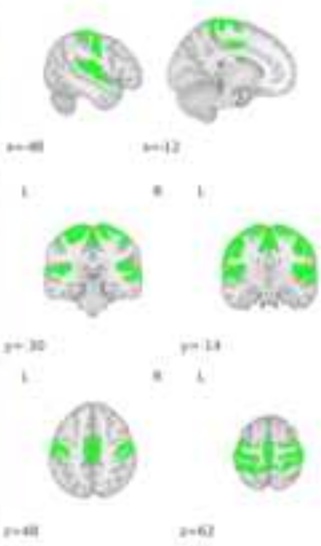

Somatomotor network (green)

Click here to access/download;Figure;rsfmri_net_comb2.png

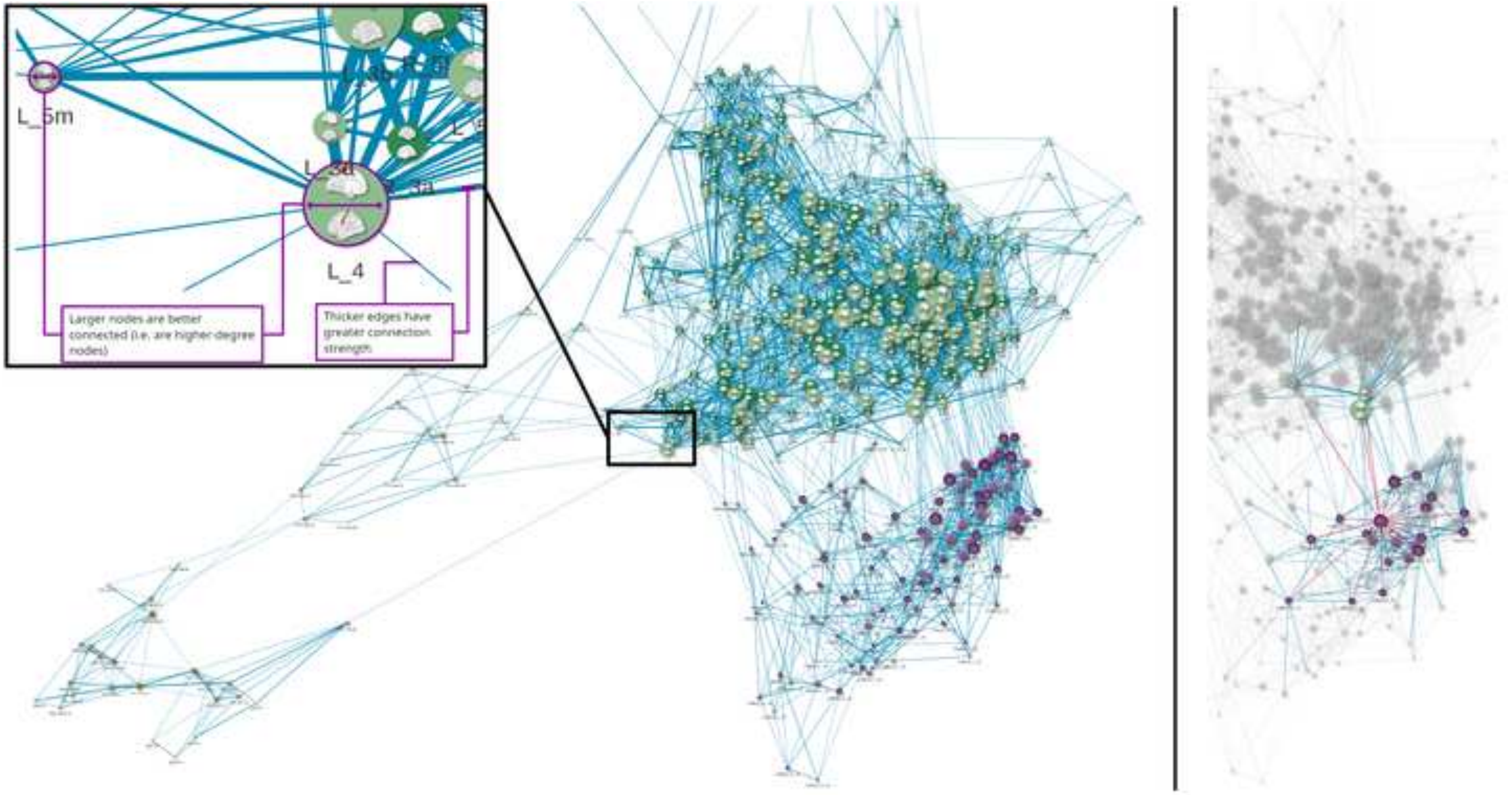

Click here to access/download;Figure;figure-results_rsfmri.png

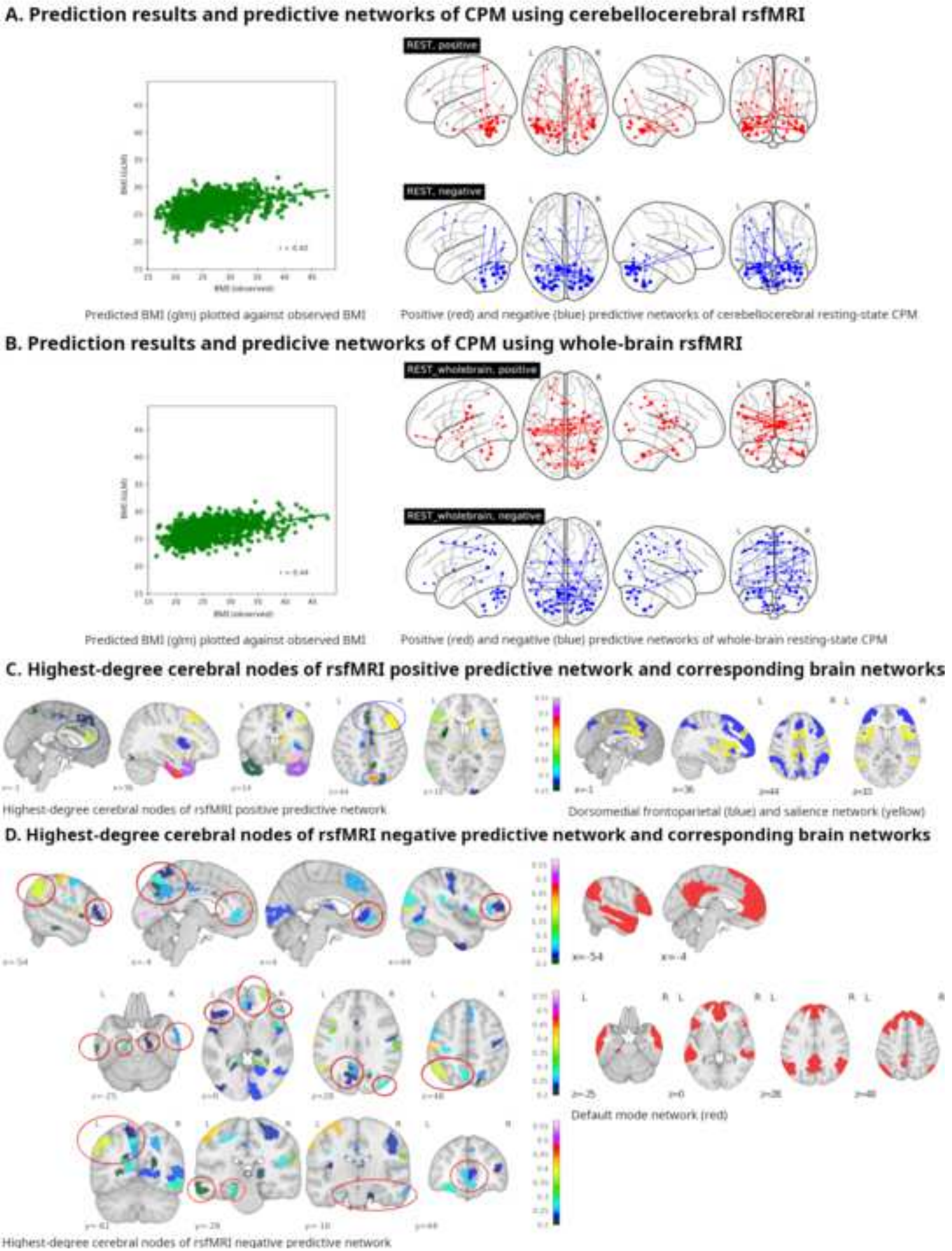

Click here to access/download;Figure;CPM_explainer.png

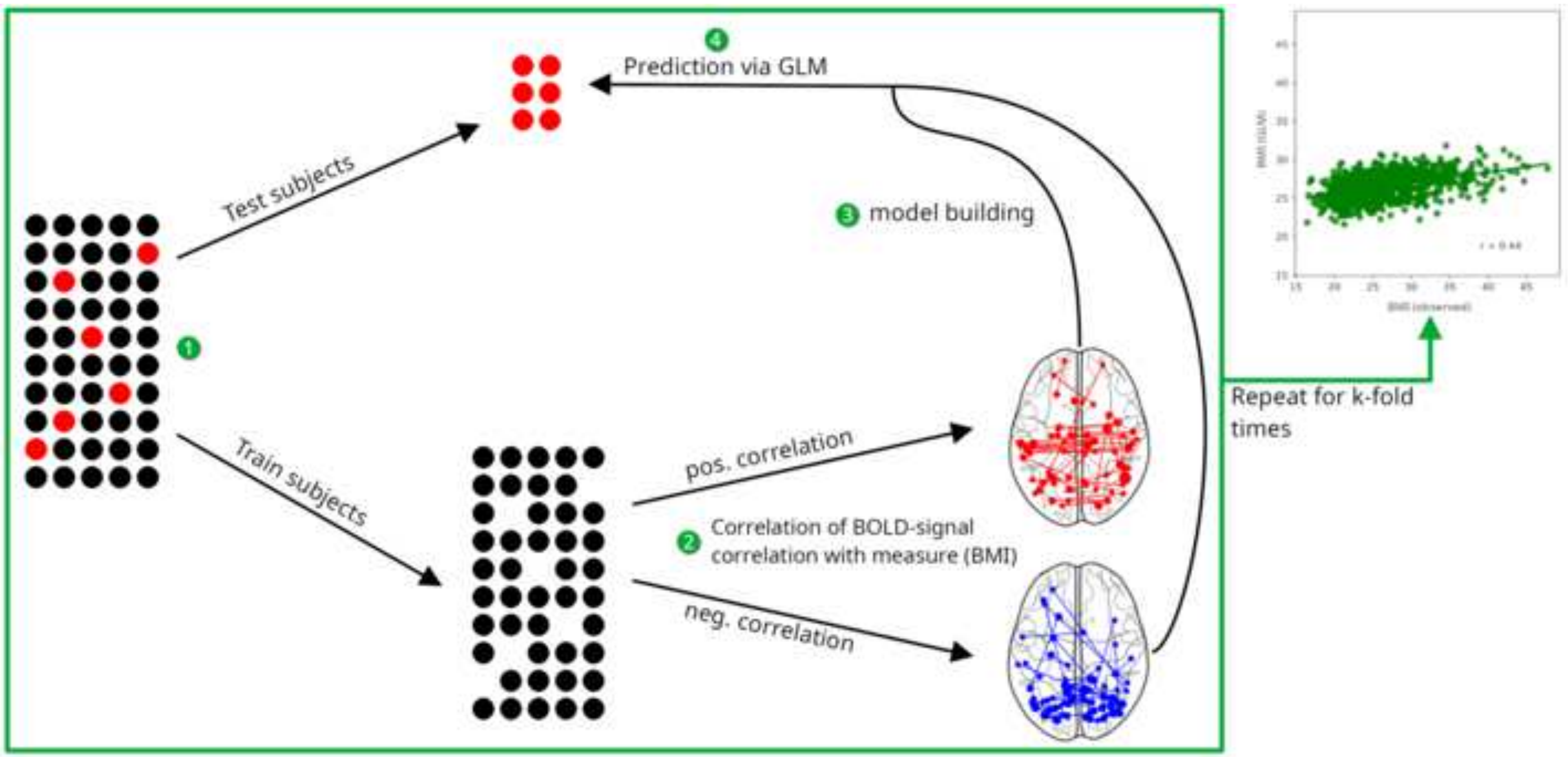

Click here to access/download;Figure;parcellated_correlation_explainer_v2.png

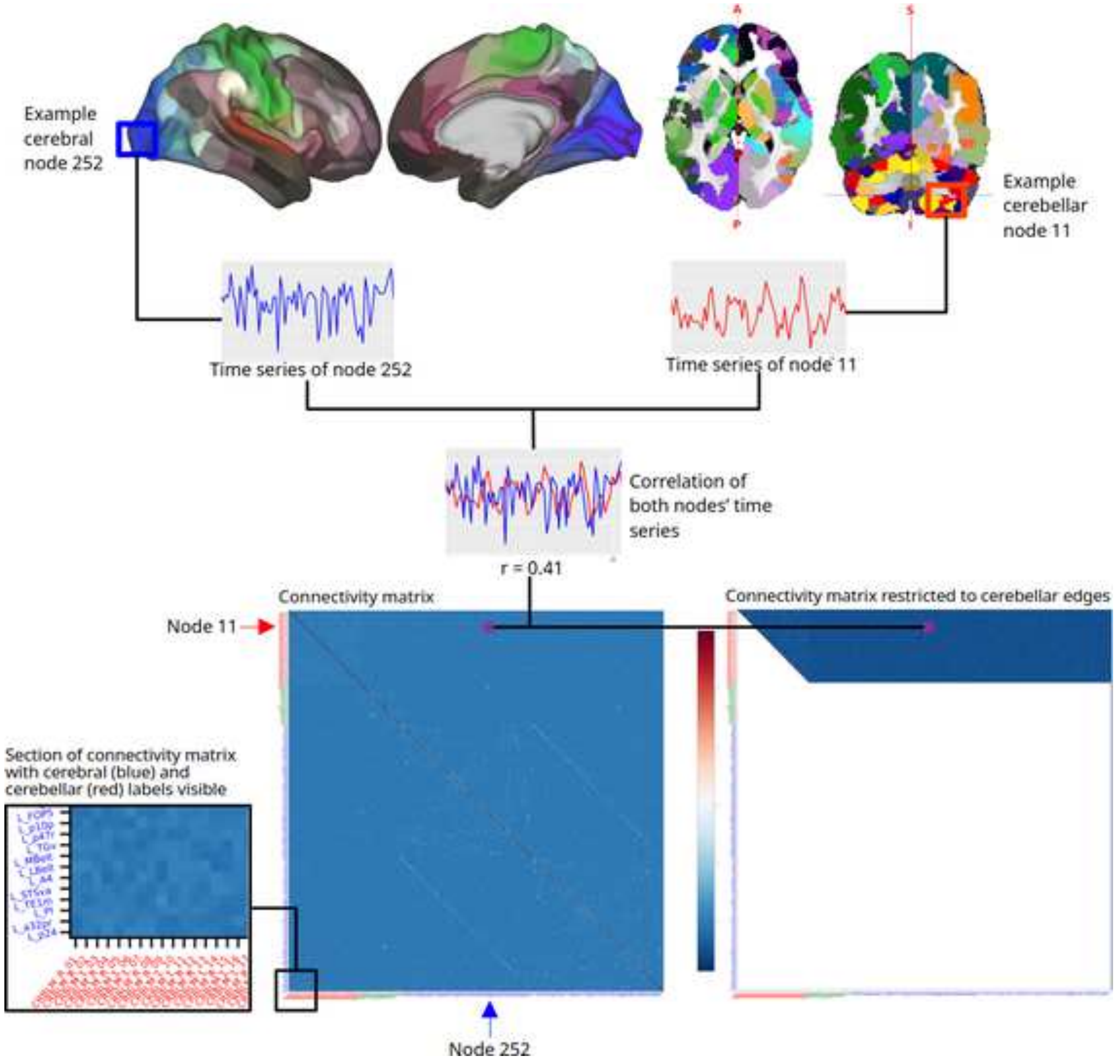

Click here to access/download
Supplementary Material
Supplementary_Material.docx

Response to Reviewers

Click here to access/download
Supplementary Material
Response_to_Reviewers.pdf
