# Supplementary material for: Cerebellocerebral connectivity predicts body mass index: a new open-source Python-based framework for connectome-based predictive modeling
Source: Gigascience. 2025 Mar 12;14:giaf010. doi: 10.1093/gigascience/giaf010 (PMC11899596; doi:10.1093/gigascience/giaf010)
Supplement: giaf010_Response_to_Reviewer_Comments_Original_Submission [file giaf010_response_to_reviewer_comments_original_submission.pdf]

Author's Response To Reviewer Comments

Dear editorial board of GigaScience,

thank you for giving us the opportunity to submit a revised version of our manuscript. Since we have included some figures in our response, we have addressed the reviewers' points in a separate file (see uploaded Response_to_Reviewers.pdf). In addition, as requested, we registered our software and workflow with bio.tools, workflowhub.eu and as an RRID. The relevant references and IDs have been included in the revised manuscript.

Best regards,
The authors
