# Supplementary material for: Cerebellocerebral connectivity predicts body mass index: a new open-source Python-based framework for connectome-based predictive modeling
Source: Gigascience. 2025 Mar 12;14:giaf010. doi: 10.1093/gigascience/giaf010 (PMC11899596; doi:10.1093/gigascience/giaf010)
Supplement: giaf010_Reviewer_1_Report_Original_Submission — Bo-yong Park -- 10/14/2024 [file giaf010_reviewer_1_report_original_submission.pdf]

Reviewer Report

Title: Cerebellocerebral Connectivity Predicts Body Mass Index: a New Open-Source Python-based Framework for Connectome-based Predictive Modeling

Version: Original Submission **Date:** 10/14/2024

Reviewer name: Bo-yong Park

Reviewer Comments to Author:

Many thanks for inviting me to review the manuscript by Bachmann and colleagues. In this work, the authors employed connectome-based predictive modeling to predict body mass index (BMI) using cerebellar-cerebral functional connectivity derived from resting-state and task fMRI. Exploring BMI-related cerebellar connectivity beyond the typical focus on cortical and subcortical regions is intriguing. However, there are critical methodological issues that undermine the reliability of the results, which need to be appropriately addressed. Additionally, some minor formatting issues and typos should be corrected.
["Data Description" section]
- This section could be integrated into the Methods section.
- In the first sentence, the authors mention that tfMRI had been used for the analysis. The specific type of tfMRI used should be stated here, even though further details are provided in the Methods section.
- The MRI acquisition parameters need to be provided. Currently, only spatial resolution and TR are mentioned, which is insufficient. Additional acquisition parameters should be included.
- The preprocessing steps should be described in greater detail. The current description primarily focuses only on the registration step.
["Methods" section]
- The description of the parcellation scheme is overly detailed and can be simplified into one or two sentences.
- The authors used a tangent-based approach to construct the correlation matrix. How does this approach differ from Fisher's r-to-z transformation, which is a standard method for normalizing skewed distributions?
["Methods - Connectome-based predictive modeling (CPM)" section]
- The k of 128 was used for k-fold cross-validation, and it seems too large. How many subjects were included in the training and testing phases? This setup may lead to overfitting.
- In the sentence "~connections between nodes/parcels 'als' represented by Pearson's r~" -> I believe 'als' is a typo.
- The authors controlled nuisance variables (gender, age, ethnicity) while assessing the correlation between connectivity strength and BMI. However, these variables were already removed when constructing the connectivity matrix. If this was intentional, please clarify the rationale.
- Only a p-value threshold of 0.05 was applied. Multiple comparison corrections should be considered to ensure the validity of the results.
- How many edges were entered into the GLM on average? If a large number of features were used to

construct the predictive model, overfitting may be a concern. Feature selection techniques, such as regularization, should be considered.
["Methods - Statistical significance" section]
- Similar to my previous comment, permutation testing does not inherently correct for multiple comparisons across different brain regions. It is simply a nonparametric test used when the data do not satisfy normality assumptions. Multiple comparison corrections should still be applied.
["Methods - Overlap with networks predictive of related measures" section]
- Multiple comparisons arising from calculating correlations with different cognitive measures should be corrected.
["Results - Population" section]
- In the Methods section, the authors stated that siblings were removed to account for potential genetic influences. However, based on Table 1, the number of participants suggests that siblings are included. If the siblings were removed when constructing the predictive models, were these models then applied to the testing phase with participants that included siblings? This approach seems inconsistent and requires clarification.
["Results - Resting-state functional connectivity of parcels" section]
- This section seems unnecessary.
- Fig. 5 seems to refer to Fig. 3.
["Results - CPM" section]
- The results are presented in a list-like format and are somewhat superficial. It is difficult to discern which connections are most important in explaining BMI. A clearer presentation of key findings would be helpful.
[Figures]
- The order of the figures is inconsistent and should be arranged according to the sequence in which they are first mentioned in the text.
- The texts in the figures are too small. It should be enlarged for better readability.
["Discussion" section]
- I expected a more detailed interpretation of the cerebellum's distinct functions in explaining their associations with obesity phenotypes. However, the current discussion primarily focuses on cortical and subcortical connectivity. This makes it difficult to understand the topological underpinnings of BMI-related cerebellar connectivity. A more in-depth regions-specific discussion of the cerebellum is needed.

Methods

Are the methods appropriate to the aims of the study, are they well described, and are necessary controls included? Choose an item.

Conclusions

Are the conclusions adequately supported by the data shown? Choose an item.

Reporting Standards

Does the manuscript adhere to the journal's guidelines on minimum standards of reporting? Choose an item.

Choose an item.

Statistics

Are you able to assess all statistics in the manuscript, including the appropriateness of statistical tests used? Choose an item.

Quality of Written English

Please indicate the quality of language in the manuscript: Choose an item.

Declaration of Competing Interests

Please complete a declaration of competing interests, considering the following questions:

- Have you in the past five years received reimbursements, fees, funding, or salary from an organisation that may in any way gain or lose financially from the publication of this manuscript, either now or in the future?
- Do you hold any stocks or shares in an organisation that may in any way gain or lose financially from the publication of this manuscript, either now or in the future?
- Do you hold or are you currently applying for any patents relating to the content of the manuscript?
- Have you received reimbursements, fees, funding, or salary from an organization that holds or has applied for patents relating to the content of the manuscript?
- Do you have any other financial competing interests?
- Do you have any non-financial competing interests in relation to this paper?

If you can answer no to all of the above, write 'I declare that I have no competing interests' below. If your reply is yes to any, please give details below.

I declare that I have no competing interests

I agree to the open peer review policy of the journal. I understand that my name will be included on my report to the authors and, if the manuscript is accepted for publication, my named report including any attachments I upload will be posted on the website along with the authors' responses. I agree for my report to be made available under an Open Access Creative Commons CC-BY license (http://creativecommons.org/licenses/by/4.0/). I understand that any comments which I do not wish to be included in my named report can be included as confidential comments to the editors, which will not be published.

Choose an item.

To further support our reviewers, we have joined with Publons, where you can gain additional credit to further highlight your hard work (see: https://publons.com/journal/530/gigascience). On publication of this paper, your review will be automatically added to Publons, you can then choose whether or not to claim your Publons credit. I understand this statement.

Yes Choose an item.
