# Supplementary material for: Cerebellocerebral connectivity predicts body mass index: a new open-source Python-based framework for connectome-based predictive modeling
Source: Gigascience. 2025 Mar 12;14:giaf010. doi: 10.1093/gigascience/giaf010 (PMC11899596; doi:10.1093/gigascience/giaf010)
Supplement: giaf010_Reviewer_2_Report_Original_Submission — Sandra Chanraud -- 10/30/2024 [file giaf010_reviewer_2_report_original_submission.pdf]

Reviewer Report

Title: Cerebellocerebral Connectivity Predicts Body Mass Index: a New Open-Source Python-based Framework for Connectome-based Predictive Modeling

Version: Original Submission **Date:** 10/30/2024

Reviewer name: Sandra Chanraud

Reviewer Comments to Author:

In the article "Cerebellocerebral Connectivity Predicts Body Mass Index: a New Open-Source Python-based Framework for Connectome-based Predictive Modeling", the authors have nicely revealed, using CPM in the CP cohort, brain areas and brain networks related to affected task in obesity.
I only have few comments:
1. The authors say, "As we are interested in cerebellocerebral connections only, we purged our matrices of connections of non-interest (see bottomright panel of Figure 1). Aside from limiting our connections of interest as informed by our hypothesis, no further anatomical assumptions were imposed on the data (Page 3, upper right paragraph)."
I am afraid it would overinflate the role of the cerebellum.
The authors addressed this by show that the whole-brain prediction r-square was 0.4, compared to 0.44 of using cerebrocerebellar (Figure 5A & 5B) and said "which confirms the cerebellum's prominence with respect to obesity-related brain."
But this kind of speaks to how CPM works - just using correlations for significance.
2. (i) On a related note, the k-fold was taken as 128. I am not sure I understand why - for the rsFMRI, that would mean the testing sample is around 10? I am not familiar with this testing, but I cannot see how such a small sample would validate the model, no matter how many permutations the authors took.
(ii) And for the test subjects, the correction for multiple comparisons was using permutations of BMI in the small test sample with a threshold of p<=0.001. I don't think this helps with the corrections for the significant edges selected through CPM.
(iii) The model was made by combining negative and positive correlative edges and taking edges with p<0.05. From my experience, I would say that the threshold is not stringent enough.
3. The authors report "We then performed CPM (with averaged tfMRI, as it yielded the most promising results in our primary analysis)" So did they just the average fMRI signal, because it gave good results?
4. The last paragraph before the discussion says "Crucially, a significant overlap between networks predictive for BMI and other measures of interest was only observed when comparing positive predictive networks for BMI with negative predictive networks of the other measures (and vice versa, see Figure 7 for an example). This is in line with our theory, which predicts this kind of inverse relationship." I wonder if rather than the brain networks, it could be because of the inverse relationship between BMI and obesity markers.
5. To finish, the authors explained the results by "we accept the premises of (a) the cerebellum's role in cognition, affective regulation and task execution, (b) an inverse correlation of BMI and performance in

these domains, and suppose
that the cerebellum's altered functional status in overweight and obesity is an expression of these premises, our data would suggest an overall inhibitory role of the cerebellum." So this just becomes a case of cause-and-effect, relying on correlation.

Methods

Are the methods appropriate to the aims of the study, are they well described, and are necessary controls included? Choose an item.

Conclusions

Are the conclusions adequately supported by the data shown? Choose an item.

Reporting Standards

Does the manuscript adhere to the journal's guidelines on minimum standards of reporting? Choose an item.

Choose an item.

Statistics

Are you able to assess all statistics in the manuscript, including the appropriateness of statistical tests used? Choose an item.

Quality of Written English

Please indicate the quality of language in the manuscript: Choose an item.

Declaration of Competing Interests

Please complete a declaration of competing interests, considering the following questions:

- Have you in the past five years received reimbursements, fees, funding, or salary from an organisation that may in any way gain or lose financially from the publication of this manuscript, either now or in the future?
- Do you hold any stocks or shares in an organisation that may in any way gain or lose financially from the publication of this manuscript, either now or in the future?
- Do you hold or are you currently applying for any patents relating to the content of the manuscript?
- Have you received reimbursements, fees, funding, or salary from an organization that holds or has applied for patents relating to the content of the manuscript?
- Do you have any other financial competing interests?

- Do you have any non-financial competing interests in relation to this paper?

If you can answer no to all of the above, write 'I declare that I have no competing interests' below. If your reply is yes to any, please give details below.

I declare that I have no competing interests' below.

I agree to the open peer review policy of the journal. I understand that my name will be included on my report to the authors and, if the manuscript is accepted for publication, my named report including any attachments I upload will be posted on the website along with the authors' responses. I agree for my report to be made available under an Open Access Creative Commons CC-BY license (http://creativecommons.org/licenses/by/4.0/). I understand that any comments which I do not wish to be included in my named report can be included as confidential comments to the editors, which will not be published.

Choose an item.

To further support our reviewers, we have joined with Publons, where you can gain additional credit to further highlight your hard work (see: https://publons.com/journal/530/gigascience). On publication of this paper, your review will be automatically added to Publons, you can then choose whether or not to claim your Publons credit. I understand this statement.

Yes Choose an item.
